# Nuclear and Mitochondrial SSU rRNA Genes Reveal Hidden Diversity of *Haptophrya* Endosymbionts in Freshwater Planarians and Challenge Their Traditional Classification in Astomatia

**DOI:** 10.3389/fmicb.2022.830951

**Published:** 2022-04-14

**Authors:** Matej Rataj, Tengyue Zhang, Peter Vd’ačný

**Affiliations:** Department of Zoology, Faculty of Natural Sciences, Comenius University in Bratislava, Bratislava, Slovakia

**Keywords:** 16S and 18S rRNA genes, compensatory base changes, cryptic species, endosymbionts, Scuticociliatia, secondary structure

## Abstract

Like many other aquatic animals, freshwater planarians have also become partners of symbiotic ciliates from the class Oligohymenophorea. In the present study, we explored the hidden diversity and addressed the questionable systematic position of mouthless obligatory gut endosymbionts of freshwater planarians, using the nuclear and mitochondrial SSU rRNA genes. Although all isolated ciliates morphologically corresponded to a single species, molecular analyses suggested the existence of three genetically distinct entities: *Haptophrya planariarum*, *Haptophrya dugesiarum* nov. spec., and *Haptophrya schmidtearum* nov. spec. The two former species share the same planarian host, which indicates a speciation model involving one duplication event without host switching. Such a diversification pattern was recognized also in astome ciliates inhabiting megascolecid and glossoscolecid earthworms. The present multi-gene phylogenies along with the secondary structure of the mitochondrial 16S rRNA molecule, however, challenge the traditional classification of *Haptophrya* within the subclass Astomatia. *Haptophrya* very likely evolved from an orphan scuticociliate lineage by the loss of oral apparatus and by the transformation of the thigmotactic field into an adhesive sucker. Since astomy evolved multiple times independently within the Oligohymenophorea, the loss of cell mouth cannot be used as a sole argument for the assignment of *Haptophrya* to the Astomatia anymore.

## Introduction

Freshwater triclad planarians (order Tricladida Lang, 1884) represent a relatively derived monophyletic lineage of free-living flatworms. The age of their origin was mostly inferred by biogeographical events, as the proper fossil record of triclads is extremely limited due to their fragile bodies. Diversification of the family Dugesiidae Ball, 1974 might date back even to the epoch of the supercontinent Pangaea (~300 Mya). Molecular clock estimates, however, suggest that triclads could be a way more ancient (for a review, see Vila-Farré and Rink, 2018 and references cited therein). Interestingly, their divergence time estimates meet those of their ciliate endosymbionts, which indicates that the ciliate-planarian associations could be very ancient (Rataj and Vďačný, 2018; Zhang and Vďačný, 2022). Despite the very long evolutionary history, the bauplan of these dorso-ventrally flattened worms remained highly uniform. The limbless narrowly leaf-shaped body covered with mucus and carrying a protrusive pharynx has ensured them a position of successful predators over millions of years. The conservativeness of the planarian bauplan might have also lowered the pressure on the morphological evolution of their ciliate symbionts, which in turn could lead to their cryptic speciation. As concerns ecological demands, planarians inhabit all kinds of freshwater habitats all over the globe. For instance, they live in fishponds, lakes of various sizes and depths, in temporary water bodies such as puddles, swamps, cave ponds, but also in springs, creeks, streams, and shallow sections of rivers (Reynoldson, 1981). All these environments are commonly inhabited by ciliates as well (Lynn, 2008), a fact enabling encounters of planarians with ciliates.

Like many other aquatic animals, freshwater planarians have also become partners of symbiotic ciliates. Even though only a small fraction of triclads has been properly examined for the presence of symbiotic ciliates, at least three different types of associations were detected. The character of these symbiotic systems and the impact on planarians differ significantly, however. All ciliates so far isolated either from the gut, the body cavity, or from the surface of planarians are members of the highly diverse class Oligohymenophorea de Puytorac et al., 1974. The first description of a symbiotic ciliate from freshwater planarians comes from von Siebold (1839). He allegedly found a harmless endocommensal carrying an adhesive sucker without any additional spine-like structures, currently known as *Haptophrya planariarum* (von Siebold, 1839) Stein, 1867, in the gut of *Planaria torva* Müller, 1773. The morpho-molecular characterization of *H. planariarum* and its host range, which accounts for seven triclad species living in the Holarctis and Palaearctic, were reviewed elsewhere (Rataj and Vďačný, 2018). Another endozoic ciliate described from freshwater triclads is *Annelophrya sphaeronucleata* (Georgévitch, 1950) Lom, 1959. It is easily distinguished from *H. planariarum* by the presence of numerous hooks in the thigmotactic area (Georgévitch, 1950; Lom, 1959). There are, unfortunately, no recent records of this species and no DNA samples are available from the initial discovery. *Haptophrya* Stein, 1867 and *Annelophrya* Lom, 1959 are traditionally classified in the subclass Astomatia Schewiakoff, 1896 in compendium of Lynn (2008).

The second group of ciliates associated with planarians includes surface-dwelling mobilid peritrichs (subclass Peritrichia Stein, 1859) belonging to the genera *Urceolaria* Stein, 1867 and *Trichodina* Ehrenberg, 1830. Mobilids thus entered into relationships with planarians at least two times independently. For a long time, only a single species from each genus was known to inhabit planarians (Haider, 1964). Recently, we have recognized that *Trichodina steinii* Claparède and Lachmann, 1859 is a complex of three morphologically cryptic species, whose occurrence perfectly correlates with that of their different planarian hosts. *Urceolaria mitra* (von Siebold, 1848) Stein, 1867 very likely also covers two cryptic but co-occurring species (Rataj and Vďačný, 2021). Interestingly, *Urceolaria* and *Trichodina* can either occupy their hosts separately or they can co-occur as well (Reynoldson, 1947). Both attach to their hosts with the aid of a skeletal ring, leaving a visible mark on the host surface (Bowen and Ryder, 1994). The extensive work on ecology, dispersal, and population dynamics of *U. mitra* showed that mobilids only use the host-produced water currents for feeding purposes and cause no direct harm to their hosts (Reynoldson, 1950, 1951, 1955, 1956).

Unlike mobilids, representatives of the third group of ciliates detected in freshwater triclads are histophagous hymenostomes (subclass Hymenostomatia Delage and Hérouard, 1896). They are capable of causing severe damage to their hosts by feeding on their parenchyma tissues, not seldomly leading even to death (Wright, 1981). Members of only two hymenostome genera have been recognized to infect planarians. More specifically, three *Ophryoglena* Ehrenberg, 1830 species (*O. intestinalis* Rossolimo, 1926, *O. parasitica* André, 1909 and *O. pyriformis* Rossolimo, 1926) and six *Tetrahymena* Furgason, 1940 species were isolated from the gut and body cavity of at least seven different freshwater triclads (André, 1909; Rossolimo, 1926; Wright, 1981; Rataj and Vďačný, 2020). However, only four *Tetrahymena* species were identified by a means of molecular methods (Rataj and Vďačný, 2020). The identity of the two remaining *Tetrahymena* species remains questionable due to their substantial morphological uniformity.

Symbiotic relationships are in the center of interest of a broad variety of ecological, physiological, parasitological, cell biological, and environmental studies (e.g., Bignell, 2000; Dimijian, 2000a,b; Sapp, 2004). The development of molecular methods enabled the examination of symbionts and their hosts also from the viewpoint of molecular taxonomy, phylogenetics, and biogeography. The recent multi-gene analyses brought serious concerns about the alleged omnipresence and non-specificity of ciliate symbionts (c.f. Lynn et al., 2014; Moon-van der Staay et al., 2014; Rataj and Vďačný, 2020, 2021; Obert et al., 2021). Although the species concept and approaches used to discriminate cryptic species have a paramount effect on the diversity recognized (Caron, 2013; Struck et al., 2018), the very deep divergences in mitochondrial genes of symbiotic ciliates isolated from different hosts cannot be ignored any more (e.g., Lynn et al., 2014; Obert et al., 2021; Rataj and Vďačný, 2021). In the present paper, we studied nuclear and mitochondrial genes encoding for the small subunit rRNA molecules, including their secondary and tertiary structures, to address the real diversity of the morphologically uniform, gut endosymbiont of freshwater planarians, *H. planariarum*. The integrative approach significantly confronted the current taxonomic concept of *H. planariarum* and challenged its traditional systematic position in the subclass Astomatia as well.

## Materials and Methods

### Material Collection and Processing

Planarians were collected from a variety of freshwater bodies at 28 localities in Slovakia (Central Europe). Detailed descriptions of collection sites were provided by Rataj and Vďačný (2020) and are summarized in Supplementary Table S1. Identification of planarians was based on morphological criteria, and the identity of some taxa was confirmed also by sequencing their cytochrome *c* oxidase subunit I (COI). Primers and PCR conditions used for amplification of the planarian COI gene are listed in Supplementary Tables S2, S3. The molecular assignment of the examined planarians to species is shown in Supplementary Figure S1. Planarians were processed and dissected as described by Rataj and Vďačný (2018, 2019). Haptophryans were isolated from the pharynx plicatus and gut content of their planarian hosts with Pasteur micropipettes. They were investigated at low (50–400×) and high (1,000×) magnifications with bright field and differential interference contrast under a Leica DM2500 optical microscope. Their ciliary pattern and nuclear apparatus were revealed with protargol impregnation (Wilbert, 1975). Habitus and taxonomically important features were captured on microphotographs by a Canon EOS 80D camera. Measurements were taken in ImageJ ver. 1.49.

Single cells of ciliates were collected for molecular analyses as described by Rataj and Vďačný (2018, 2019). More specifically, each specimen was separately placed in 180 μl of cell lysis buffer (Promega, Fitchburg, Wisconsin, United States) and its genomic DNA was extracted with the ReliaPrep™ Blood gDNA Miniprep System (Promega, Fitchburg, Wisconsin, United States). The mitochondrial 16S rRNA gene (16S henceforth) and the nuclear 18S rRNA gene (18S henceforth) were consequently PCR amplified. Primers and PCR conditions are provided in Supplementary Tables S2, S3. Unfortunately, all attempts to amplify the nuclear ITS1-5.8S-ITS2 region and the mitochondrial COI gene failed. The list of tested primers is provided in Supplementary Table S2. PCRs were carried out with the GoTaq® Long PCR Master Mix (Promega, Fitchburg, Wisconsin, United States), following the protocol described in Rataj and Vďačný (2021). Sequencing was conducted in Macrogen Europe B.V. (Amsterdam, The Netherlands) on an ABI 3730 automatic sequencer. Newly acquired sequences were examined in Chromas ver. 2.6.6 (Technelysium Pty Ltd., South Brisbane, Australia) and only high-quality sequence fragments were assembled into contigs in BioEdit ver. 7.2.5 (Hall, 1999). More specifically, the Phred score was ≥20 and the length of fragments typically exceeded 900 nt. The assemble parameters were as follows: a minimum of 150 base overlap and a minimum match of 95%.

### Predicting Secondary Structures of rRNA Molecules

The secondary structure of 16S and 18S rRNA molecules was predicted using R2DT (Sweeney et al., 2021). The 16S secondary structure model proposed for *Paramecium tetraurelia* and *Tetrahymena pyriformis* (Cannone et al., 2002)^1^ as well as the 18S model proposed for *Tetrahymena thermophila* by Lee and Gutell (2012) were also taken into account. These models were based on ribosomal crystal structures and hence are consistent with 3D ribosomal structures, accounting for non-canonical base pairs on parity with Watson-Crick base pairs. Since helices 39 and 40 of 16S are highly variable and much longer in *Haptophrya* than in other ciliates, the RNAstructure web server (Mathews, 2004)^2^ was employed to find their common secondary structure motif. The secondary structure of the divergent helices 21, 33, 39, 40, and 44 of 16S was also analyzed using the free-energy minimization approach on the Mfold web server ver. 3.0 (Zuker, 2003).^3^

The incorporation of non-canonical base pairs in 16S helices modeled on the RNAstructure and Mfold web servers followed the RC/Rp pipeline proposed by Rybarczyk et al. (2015). More specifically, RNA 3D structures were predicted from the putative secondary structures using RNAComposer (Popenda et al., 2012).^4^ Resulting PDB files with tertiary structure information served as input for RNApdbee (Antczak et al., 2014; Zok et al., 2018).^5^ Canonical and non-canonical base pairs were identified from the tertiary structures with the 3DNA/DSSR option, secondary structures were resolved using the hybrid algorithm and visualized using the VARNA-based procedure. Finally, the recognized non-canonical base pairs were included in the 16S rRNA models. Secondary structures were plotted either in Varna ver. 3.93 (Darty et al., 2009) or with the help of R2DT (Sweeney et al., 2021) and TRAVeLer (Elias and Hoksza, 2017). The helix number system of rRNA molecules was according to Lee and Gutell (2012) and Petrov et al. (2014).

### Genetic Distances, Networks, and Molecular Diagnostic Characters

16S and 18S sequences were aligned according to the primary and predicted secondary structures (see above) using the package 4SALE ver. 1.7.1 (Seibel et al., 2006). Subsequently, the number of compensatory base changes (CBC) was identified with the CBCAnalyzer option (Wolf et al., 2005) supplied with 4SALE. Pairwise *p*-distances were calculated for each rRNA gene separately with a custom Python script, using a pairwise deletion option to exclude alignment gaps. Parsimony networks were constructed from the secondary structure-based alignments in PopART v.1.7 (Leigh and Bryant, 2015) using the TCS method (Clement et al., 2000). Nucleotide positions suitable for species diagnoses (i.e., molecular autapomorphies) were identified within the secondary structure-based alignments, using a custom Python script.

### Phylogenetic Analyses

To determine the phylogenetic position of *Haptophrya* within the class Oligohymenophorea, two datasets were assembled following mostly our previous study (Zhang and Vďačný, 2022) and taking into account newly published sequences of oligohymenophorean ciliates isolated from a variety of invertebrates (Rataj and Vďačný, 2020, 2021; Obert et al., 2021). The first dataset contained 18S sequences sampled across almost the whole class Oligohymenophorea. However, only taxa having also 16S sequences were selected for this dataset. The single exceptions were *Conchophthirus* and *Dexiotricha*, which were included as they are the nearest relatives of *Haptophrya* according to the BLAST search and previous single-gene analyses (e.g., Rataj and Vďačný, 2018, 2019; Antipa et al., 2020; Obert et al., 2021; Zhang and Vďačný, 2022). The second dataset contained 18S and corresponding 16S sequences. Taxon sampling and GenBank accession numbers of both datasets are collated in Supplementary Table S4.

Individual molecular markers were aligned on the MAFFT ver. 7 web server,^6^ using the iterative G-INS-i method as well as the progressive G-INS-1 method with an accurate guide tree (Katoh et al., 2019). Both alignment strategies were coupled with a gap opening penalty of 1.53 and the 200PAM/κ = 2 scoring matrix. Ambiguous nucleotide alignment positions were masked on the Gblocks ver. 0.91b server (Talavera and Castresana, 2007)^7^ with a less stringent option, allowing smaller final blocks, gap positions within the final blocks, and less strict flanking positions. Phylogenetic trees were constructed with the maximum likelihood (ML) approach as implemented in the program IQTREE ver. 1.6.10 (Nguyen et al., 2015) on the IQTREE web server (Trifinopoulos et al., 2016)^8^ and with Bayesian (BI) inference as implemented in the program MrBayes ver. 3.2.7 (Ronquist et al., 2012). Each molecular partition was assigned the best evolutionary substitution model, as selected under the BI information criterion in IQTREE. The ML search started from a BioNJ tree and the branching pattern of ML trees was assessed with 1,000 ultrafast bootstrap pseudoreplicates. All other parameters were left default. Settings in Bayesian analyses and convergence diagnostics followed our previous protocols (Rataj and Vďačný, 2021). Prior parameters of evolutionary models in Bayesian analyses were estimated in IQTREE and implemented with the “prset” command. Trees were rooted in FigTree ver. 1.2.3^9^ by placing the root on a branch separating members of the class Oligohymenophorea from those of the class Colpodea, which served as an outgroup.

## Results

### Alpha-Diversity, Prevalence, and Intensity of Haptophryans in Planarians

Two ecological groups of planarians were examined for the presence of haptophryans during the years 2016 and 2019. The first group comprised stream- and river-dwelling planarians (169 specimens of *Dugesia gonocephala*, 166 individuals of *Polycelis felina*, and four exemplars of *Crenobia alpina*), while the second group contained pond planarians (426 specimens of *Girardia tigrina*, eight individuals of *Dendrocoelum lacteum*, six exemplars of *Schmidtea lugubris*, six specimens of *Schmidtea polychroa*, and a single individual of *Polycelis nigra*). *Haptophrya* was detected only in two out of the eight planarian species examined, namely, in *D. gonocephala* collected from a variety of running waters and in *S. polychroa* living in stagnant waters. Although the abundance of haptophryans was generally very low (one to dozen of specimens per host), these endosymbiotic ciliates were noted at 12 out of the 28 localities studied.

All isolated ciliates morphologically corresponded to *H. planariarum* (see below). However, according to the present molecular analyses, the morphospecies *H. planariarum* is a complex of three genetically distinct and morphologically cryptic species (see below). The most common species is assigned to *H. planariarum*, while the two rare species are endowed here with names: *H. dugesiarum* nov. spec. and *H. schmidtearum* nov. spec. The former species was detected exclusively in *D. gonocephala* at 11 localities and co-occurred with *H. dugesiarum* at two localities (in a shallow section of the river Váh near the village of Bystrá, Veľká Fatra Mts. and in a stream running through the Fončorda residential area, Banská Bystrica, Zvolenská kotlina basin). *Haptophrya dugesiarum* was recorded only at these two localities and was also restricted to *D. gonocephala*. Finally, *H. schmidtearum* was found only at a single locality (Jurské jazierko pond, an urban oak-hornbeam forest, district of the village of Svätý Jur, Malé Karpaty Mts.). Although two planarians co-occurred in the Jurské jazierko pond, *H. schmidtearum* was consistently and multiple times isolated only from *S. polychroa* and never from *P. nigra*.

The prevalence of *H. planariarum* and *H. dugesiarum* in *D. gonocephala* was 59.76 and 13.61%, respectively. However, the prevalence of *H. schmidtearum* in *S. polychroa* was 33.33%. The intensity of all three *Haptophrya* species was consistently very low. Namely, there were one to 14 specimens of *H. planariarum* per host, 1–7 individuals of *H. dugesiarum*, and only up to four exemplars of *H. schmidtearum* per host.

### Molecular Identification of Haptophryans

In total, 96 new sequences were obtained from three morphologically cryptic *Haptophrya* species isolated from freshwater planarians (Table 1). They can be unambiguously distinguished by rRNA gene sequences, whereby the nuclear 18S rRNA gene can serve as a pre-barcode and the mitochondrial 16S rRNA gene as a DNA barcode.

**Table 1:** Characterization and origin of nuclear and mitochondrial SSU rRNA gene sequences of *Haptophrya* species analyzed in the present study.

Species	Specimen^a^	Host species	Locality^b^	18S rRNA gene	16S rRNA gene
*Haptophrya planariarum*	KD 1 DG^c^	*Dugesia gonocephala*	1	OL752480	OL752528
*Haptophrya planariarum*	KD 2 DG	*D. gonocephala*	1	OL752481	OL752529
*Haptophrya planariarum*	KD 3 DG	*D. gonocephala*	1	OL752482	OL752530
*Haptophrya planariarum*	OL 4 DG	*D. gonocephala*	3	OL752483	OL752531
*Haptophrya planariarum*	OL 6 DG	*D. gonocephala*	3	OL752484	OL752532
*Haptophrya planariarum*	OL 7 DG	*D. gonocephala*	3	OL752485	OL752533
*Haptophrya planariarum*	OL 8 DG	*D. gonocephala*	3	OL752486	OL752534
*Haptophrya planariarum*	OL 9 DG	*D. gonocephala*	3	OL752487	OL752535
*Haptophrya planariarum*	OL 11 DG	*D. gonocephala*	3	OL752488	OL752536
*Haptophrya planariarum*	MI 12 DG	*D. gonocephala*	4	OL752489	OL752537
*Haptophrya planariarum*	MI 16 DG	*D. gonocephala*	4	OL752490	OL752538
*Haptophrya planariarum*	DL 19 DG	*D. gonocephala*	2	OL752491	OL752539
*Haptophrya planariarum*	DL 20 DG	*D. gonocephala*	2	OL752492	OL752540
*Haptophrya planariarum*	DL 21 DG	*D. gonocephala*	2	OL752493	OL752541
*Haptophrya planariarum*	DL 22 DG	*D. gonocephala*	2	OL752494	OL752542
*Haptophrya planariarum*	DL 23 DG	*D. gonocephala*	2	OL752495	OL752543
*Haptophrya planariarum*	DL 24 DG	*D. gonocephala*	2	OL752496	OL752544
*Haptophrya planariarum*	DL 25 DG	*D. gonocephala*	2	OL752497	OL752545
*Haptophrya planariarum*	KD 26 DG	*D. gonocephala*	1	OL752498	OL752546
*Haptophrya planariarum*	KD 27 DG	*D. gonocephala*	1	OL752499	OL752547
*Haptophrya planariarum*	DL 28 DG	*D. gonocephala*	2	OL752500	OL752548
*Haptophrya planariarum*	KD 29 DG	*D. gonocephala*	1	OL752501	OL752549
*Haptophrya planariarum*	KDo 33 DG	*D. gonocephala*	7	OL752502	OL752550
*Haptophrya planariarum*	MH 34 DG	*D. gonocephala*	8	OL752503	OL752551
*Haptophrya planariarum*	MH 35 DG	*D. gonocephala*	8	OL752504	OL752552
*Haptophrya planariarum*	MH 36 DG	*D. gonocephala*	8	OL752505	OL752553
*Haptophrya planariarum*	MH 37 DG	*D. gonocephala*	8	OL752506	OL752554
*Haptophrya planariarum*	RT 42 DG	*D. gonocephala*	10	OL752507	OL752555
*Haptophrya planariarum*	RT 43 DG	*D. gonocephala*	10	OL752508	OL752556
*Haptophrya planariarum*	RT 44 DG	*D. gonocephala*	10	OL752509	OL752557
*Haptophrya planariarum*	RT 47 DG	*D. gonocephala*	10	OL752510	OL752558
*Haptophrya planariarum*	RT 48 DG	*D. gonocephala*	10	OL752511	OL752559
*Haptophrya planariarum*	CA 50 DG	*D. gonocephala*	11	OL752512	OL752560
*Haptophrya planariarum*	CA 51 DG	*D. gonocephala*	11	OL752513	OL752561
*Haptophrya planariarum*	CA 52 DG	*D. gonocephala*	11	OL752514	OL752562
*Haptophrya planariarum*	DL 54 DG	*D. gonocephala*	2	OL752515	OL752563
*Haptophrya planariarum*	BB 85 DG	*D. gonocephala*	20	OL752516	OL752564
*Haptophrya planariarum*	BB 99 DG	*D. gonocephala*	20	OL752517	OL752565
*Haptophrya planariarum*	ST 132 DG	*D. gonocephala*	21	OL752518	OL752566
*Haptophrya planariarum*	BY 139 DG	*D. gonocephala*	22	OL752519	OL752567
*Haptophrya planariarum*	BY 149 DG	*D. gonocephala*	22	OL752520	OL752568
*Haptophrya dugesiarum*	BB 78 DG^d^	*D. gonocephala*	20	OL752521	OL752569
*Haptophrya dugesiarum*	BB 79 DG	*D. gonocephala*	20	OL752522	OL752570
*Haptophrya dugesiarum*	BB 87 DG	*D. gonocephala*	20	OL752523	OL752571
*Haptophrya dugesiarum*	BB 98 DG	*D. gonocephala*	20	OL752524	OL752572
*Haptophrya dugesiarum*	BY 142 DG	*D. gonocephala*	22	OL752525	OL752573
*Haptophrya schmidtearum*	JJ 110 SP^e^	*Schmidtea polychroa*	25	OL752526	OL752574
*Haptophrya schmidtearum*	JJ 117 SP	*S. polychroa*	25	OL752527	OL752575

a Specimen code consists of a locality code as specified in Supplementary Table S1, an isolate code, and an abbreviation of host species name (DG, *Dugesia gonocephala*, SP, *Schmidtea polychroa*).

b For locality codes and further details, see Supplementary Table S1.

c Genomic DNA of the voucher specimen of *H. planariarum* has been deposited in the Natural History Museum in Bratislava, Slovakia (ID Collection Code 01427588).

d Genomic DNA of the holotype specimen of *H. dugesiarum* has been deposited in the Natural History Museum in Bratislava, Slovakia (ID Collection Code 01427586).

e Genomic DNA of the holotype specimen of *H. schmidtearum* has been deposited in the Natural History Museum in Bratislava, Slovakia (ID Collection Code 01427587).

**Figure 1:**
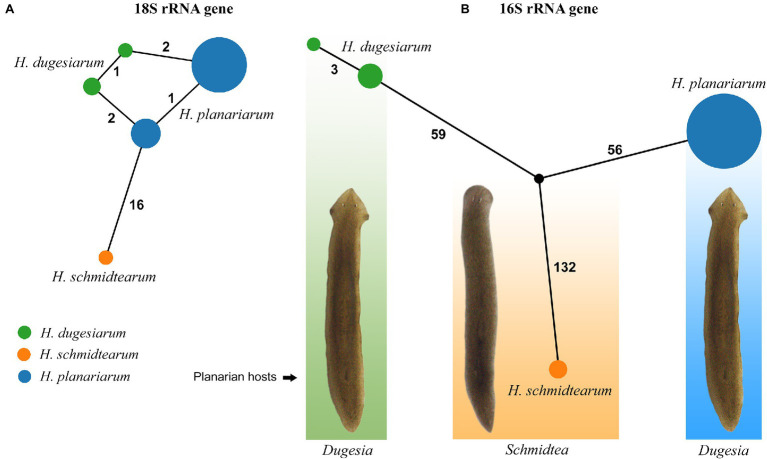
TCS networks of the nuclear 18S **(A)** and the mitochondrial 16S **(B)** rRNA gene sequences of three *Haptophrya* species isolated from freshwater planarians. Circle size corresponds to the haplotype frequency and color refers to individual *Haptophrya* species. Numbers along edges represent mutational steps.

**Figure 2:**
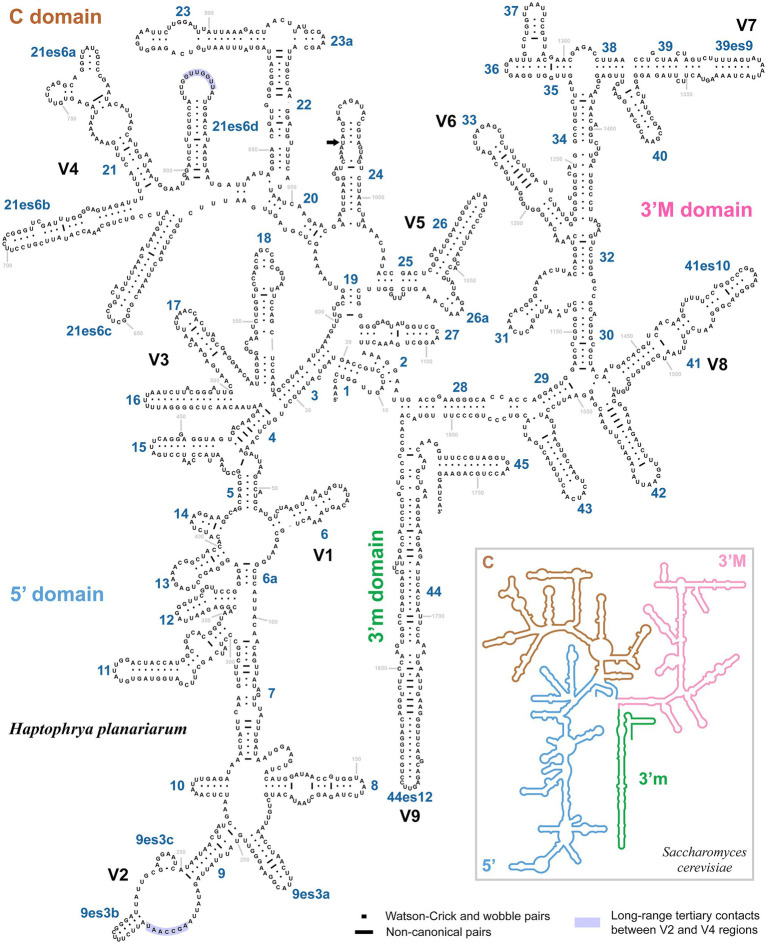
Secondary structure of the 18S rRNA molecule of *Haptophrya planariarum*, based on models taking into account 3D structures. 18S secondary structure map of *Saccharomyces cerevisiae* (inset) is from http://apollo.chemistry.gatech.edu/RibosomeGallery (Petrov et al., 2014). Arrow marks the ancestrally polymorphic position 975 in helix 24, where is either uracil or guanine. Both nucleotide states retain the RNA helical structure.

The 18S rRNA gene is 1761 nucleotides (nt) long in *H. planariarum* and *H. dugesiarum*, while 1760 nt long in *H. schmidtearum*. The guanosine–cytosine (GC) content ranges from 42.22% in *H. schmidtearum* to 42.48% in *H. planariarum*. The 18S sequences of *H. planariarum* and *H. dugesiarum* differ by only two or three nucleotide positions (Figure 1A), which corresponds to a *p*-distance of 0.11–0.17%. As evident from the parsimony network, there are two ribotypes in both *Haptophrya* species isolated from *D. gonocephala*. Interestingly, individual ribotypes of *H. planariarum* and *H. dugesiarum* are distinguished by the same site (position 975) in helix 24 where is either uracil or guanine. This nucleotide position is very likely ancestrally polymorphic and both nucleotide states retain the RNA helical structure. On the other hand, *H. schmidtearum* is fairly distant from *H. planariarum* and *H. dugesiarum*, as it differs from them by as many as 16–19 nucleotides (Figure 1A). The *p*-distance between *H. schmidtearum* and the two other *Haptophrya* species ranges from 0.97 to 1.14%, indicating a comparatively deep divergence. The 18S secondary structure models of the three *Haptophrya* species are shown in Figure 2 and Supplementary Figures S2, S3. The fraction of Watson–Crick pairs (AU and CG) ranges from 72.53% in *H. schmidtearum* to 73.12% in *H. planariarum*, wobble pairs (GU or UG) from 12.54% in *H. planariarum* to 12.93% in *H. schmidtearum*, and non-canonical pairs from 14.34% in *H. planariarum* to 14.54% in *H. schmidtearum*. The molecular diagnostic characters of these three species are accumulated in helices 21, 21es6b, and 21es6c of the V4 region, helices 38–40 of the V7 region, and helix 44 of the V9 region. The distinctness of *H. schmidtearum* from *H. planariarum* and *H. dugesiarum* is also strengthened by three CBCs.

**Figure 3:**
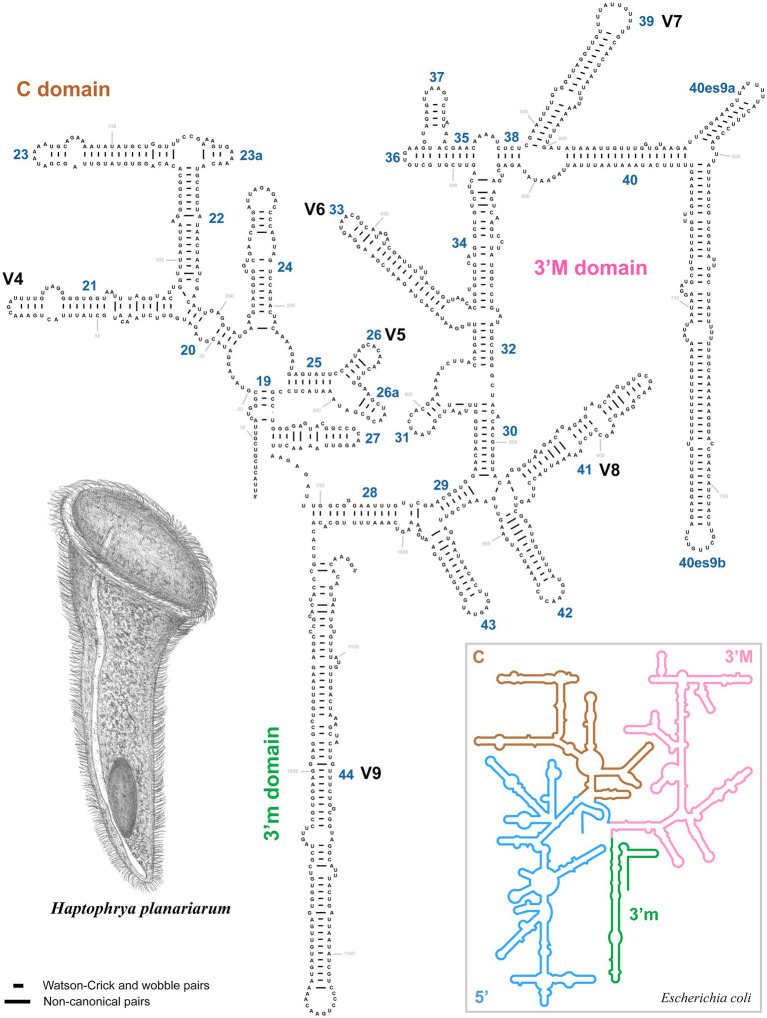
Secondary structure of the 16S rRNA molecule of *Haptophrya planariarum*, based on models taking into account 3D structures. 16S secondary structure map of *Escherichia coli* (inset) is from http://apollo.chemistry.gatech.edu/RibosomeGallery (Petrov et al., 2014).

**Figure 4:**
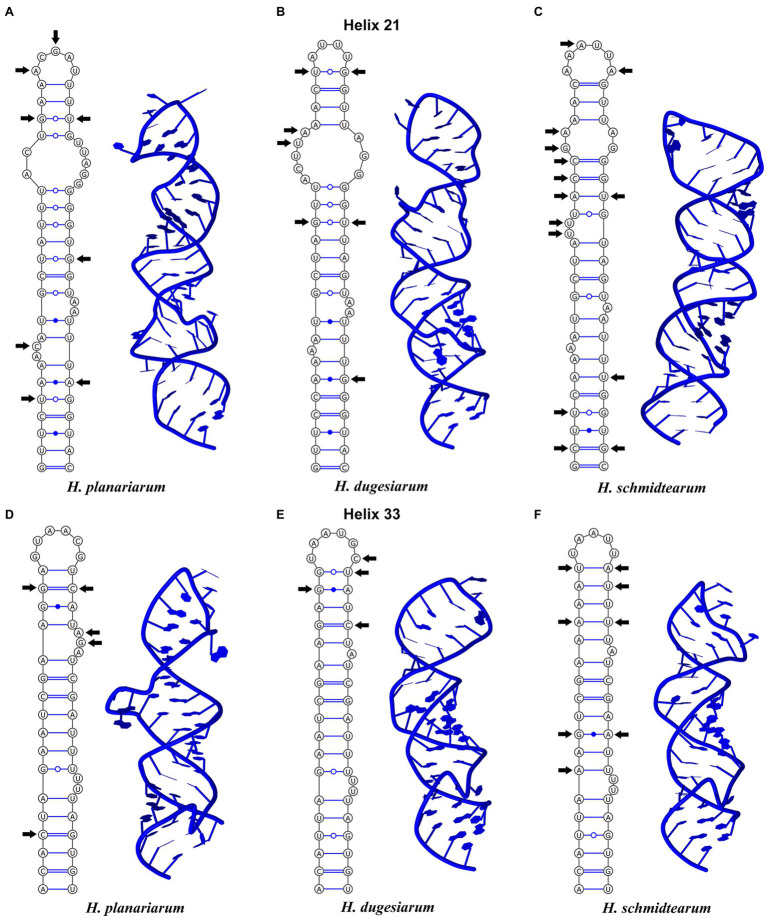
Secondary structure of the highly variable helices 21 (upper panel) and 33 (lower panel) from the C and 3’M domains of the 16S rRNA molecule of *Haptophrya planariarum* **(A,D)**, *Haptophrya dugesiarum* **(B,E)**, and *Haptophrya schmidtearum* **(C,F)**. Arrows denote the molecular diagnostic characters. Note that each species can be unambiguously distinguished by both the primary and the secondary structure of helices 21 and 33. The 16S rRNA gene can be thus used as an optimal DNA barcode for the three *Haptophrya* species.

The amplified region of the mitochondrial 16S rRNA gene covers the C, 3’M, and 3’m domains and is 1,170 nt long in *H. planariarum*, 1,172 nt long in *H. dugesiarum*, and 1,167 nt long in *H. schmidtearum*. The GC content is 37.86% in *H. planariarum*, 37.27–37.37% in *H. dugesiarum*, and 39.33% in *H. schmidtearum*. According to the network analyses, there is a single ribotype in *H. planariarum* and *H. schmidtearum*, while two ribotypes in *H. dugesiarum*. The two *Haptophrya* species isolated from *D. gonocephala* differ by as many as 115–118 mutational steps (Figure 1B), which corresponds to a *p*-distance of 11.03–11.21%. *Haptophrya schmidtearum* is separated from their common ancestor by as many as 132 mutational steps (Figure 1B). This deep divergence is reflected also by high *p*-distances, which range from 16.87 to 17.03% between *H. schmidtearum* and the two other species. The 16S secondary structure models of the three *Haptophrya* species are shown in Figure 3 and Supplementary Figures S4, S5. The fraction of Watson–Crick pairs (AU and CG) ranges from 70.89% in *H. planariarum* to 72.72% in *H. dugesiarum*, wobble pairs (GU or UG) from 14.50% in *H. dugesiarum* to 18.73% in *H. planariarum*, and non-canonical pairs from 10.38% in *H. planariarum* to 12.77% in *H. dugesiarum*. Species-specific mutations tend to accumulate in helix 21 of the C domain (Figures 4A–C), helix 33 (Figures 4D–F), helices 39 and 40 (Supplementary Figures S6A–C) of the 3’M domain, as well as in helix 44 of the 3’m domain (Supplementary Figures S7A–C). The distinctness of all three *Haptophrya* species is also strengthened by the presence of CBCs in the 16S rRNA molecule. Specifically, *H. schmidtearum* is separated from *H. planariarum* and *H. dugesiarum* by 9 and 12 CBCs, respectively. The two latter species are distinguished by 10 CBCs.

### Phylogenetic Position of *Haptophrya*

**Figure 5:**
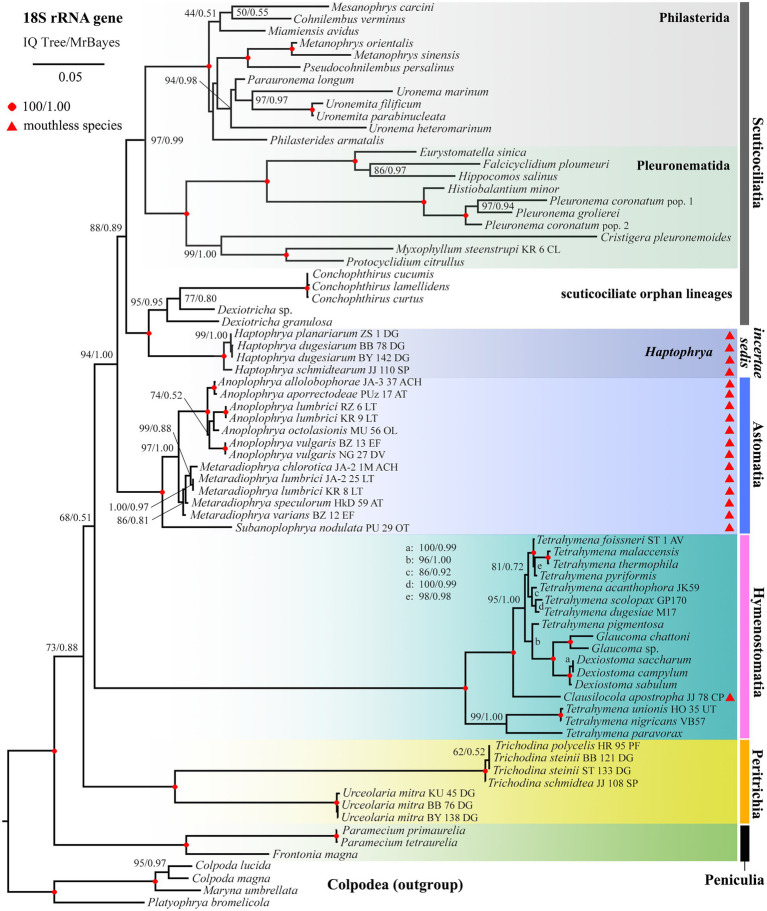
Phylogenetic tree based on the 18S rRNA gene, showing the systematic positions of *Haptophrya* and other mouthless species (marked by red triangles). Bootstrap values for maximum likelihood (ML) conducted in IQTrees as well as posterior probabilities for Bayesian inferences conducted in MrBayes were mapped onto the 50%-majority rule IQTree. Note that mouthless endosymbionts inhabiting planarians (*Haptophrya*), annelids (Astomatia), and mollusks (*Clausilocola*) do not cluster together. *Haptophrya* is most closely related to *Conchophthirus* and *Dexiotricha*, while *Clausilocola* is robustly nested within the subclass Hymenostomatia. The scale bar denotes five substitutions per one hundred nucleotide positions.

**Figure 6:**
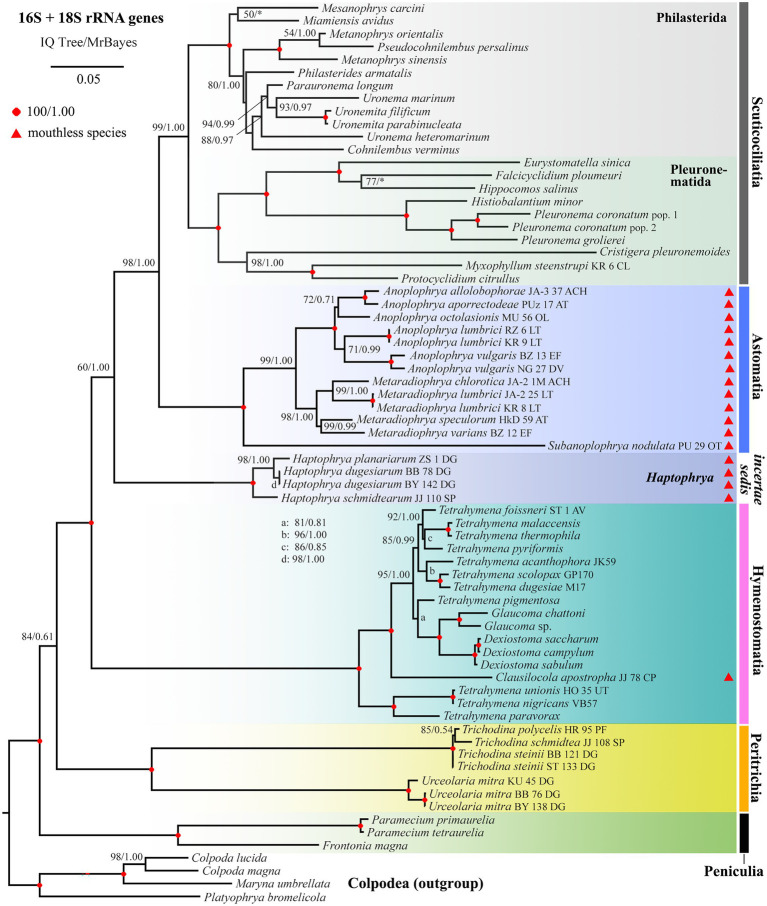
Phylogenetic tree based on the 16S and 18S rRNA genes, showing the systematic positions of *Haptophrya* and other mouthless species (marked by red triangles). Bootstrap values for ML conducted in IQTrees as well as posterior probabilities for Bayesian inferences conducted in MrBayes were mapped onto the 50%-majority rule IQTree. Note that mouthless endosymbionts inhabiting planarians (*Haptophrya*), annelids (Astomatia), and mollusks (*Clausilocola*) do not cluster together. *Haptophrya* represents an orphan lineage in the two-gene trees. Astomes group with relatives of the scuticociliate orders Philasterida and Pleuronematida, while *Clausilocola* is robustly nested within the subclass Hymenostomatia. The scale bar denotes five substitutions per one hundred nucleotide positions.

Phylogenetic positions of mouthless ciliates, represented by *Haptophrya*, *Clausilocola*, and “core” astomes, within the class Oligohymenophorea were determined using the nuclear 18S and the mitochondrial 16S rRNA gene in a ML and BI framework. Masked and unmasked alignments constructed with the iterative G-INS-i and the progressive G-INS-1 method brought highly similar results. Phylogenetic trees inferred from the masked G-INS-1 datasets are shown in Figures 5, 6, as they received the highest ML bootstrap values and posterior probabilities. The phylogenetic position of *Haptophrya* slightly differed between the single-gene (18S) and the two-gene (16S + 18S) dataset, very likely due to the limited taxon sampling in the latter one. Despite that, *Haptophrya* never grouped with astomes isolated from annelids or with *Clausilocola* isolated from gastropods. In 18S rRNA gene phylogenies, *Haptophrya* very robustly clustered with free-living members of the scuticociliate genus *Dexiotricha* and representatives of the symbiotic genus *Conchophthirus* inhabiting the mantle cavity of bivalve mussels (100% ML, 1.00 BI). This heterogeneous clade was depicted as sister to the “core” scuticociliates (Pleuronematida + Philasterida) though with very poor statistical support (88% ML, 0.89 BI). Astomes isolated from annelids formed a monophylum (100% ML, 1.00 BI) that branched off before *Haptophrya*, while *Clausilocola* was nested within the subclass Hymenostomatia (100% ML, 1.00 BI). In trees inferred from 16S + 18S rRNA gene sequences, *Haptophrya* was placed in a sister position to a highly diverse clade comprising astomes and the “core” scuticociliates. Astomes grouped with the “core” scuticociliates with strong support (98% ML, 1.00 BI) in the two-gene phylogenies. On the other hand, the grouping of haptophryans with the “core” scuticociliates was only very weakly supported in the single-gene phylogenies (see above) and hence should be taken with caution.

To summarize, mouthless endosymbionts inhabiting planarians (*Haptophrya*), annelids (“core” astomes), and mollusks (*Clausilocola*) never clustered together. *Haptophrya* was revealed to be most closely related to two scuticociliate orphan genera (*Conchophthirus* and *Dexiotricha*), “core” astomes are very likely relatives of the scuticociliate orders Philasterida and Pleuronematida, and *Clausilocola* is robustly nested within the hymenostome family Tetrahymenidae.

### Morphological Characterization and Identification of Haptophryans

**Figure 7:**
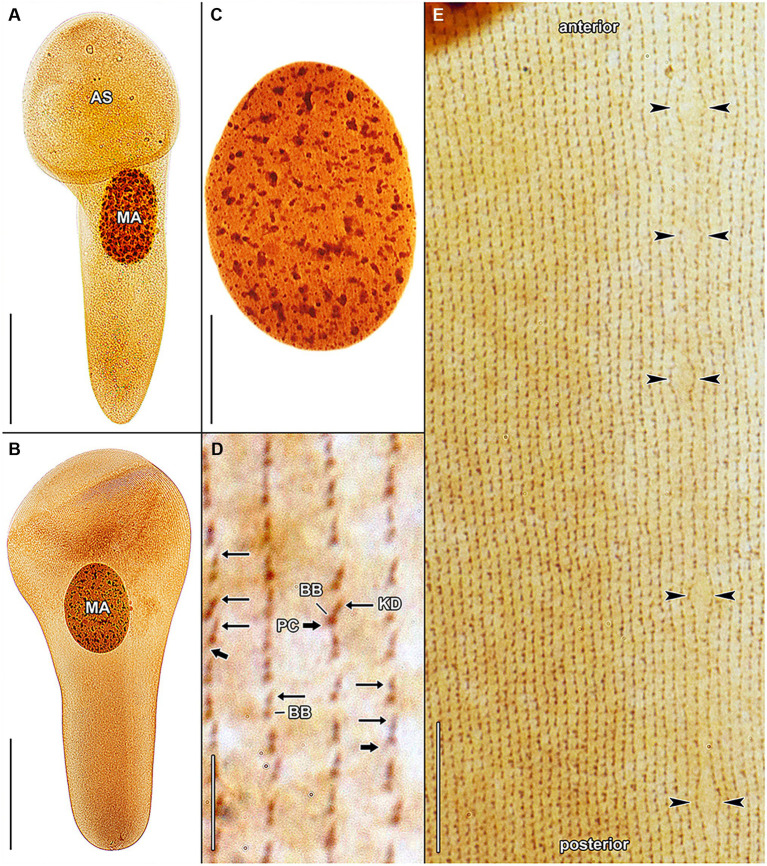
*Haptophrya planariarum*, protargol-impregnated specimens isolated from *Dugesia gonocephala* collected from the Malá Vydrica stream at the locality Železná studnička, Bratislava, Slovakia. **(A)** Ventral overview of a representative specimen. The body is campanulate and differentiated into a conspicuous anterior sucker, a more or less distinct neck-like constriction, and a cone-like trunk. **(B)** Ventral overview of a very late divider. Division occurs in freely motile conditions and is monotomic, i.e., yields two daughter cells. Axes of both daughter cells have the same orientation. **(C)** Ventral view, showing the very narrowly spaced somatic kineties composed of monokinetids. **(D)** The macronucleus is ellipsoidal and studded with globular to irregular nucleoli. **(E)** Detail of somatic kineties, showing the fibrillar associates of basal bodies. AS, adhesive sucker; BB, basal body; KD, kinetodesmal fiber (thin arrow); MA, macronucleus; and PC, postciliary microtubules (thick arrow). Scale bars = 5 μm **(E)**, 10 μm **(C)**, 30 μm **(D)**, and 100 μm **(A,B)**.

**Figure 8:**
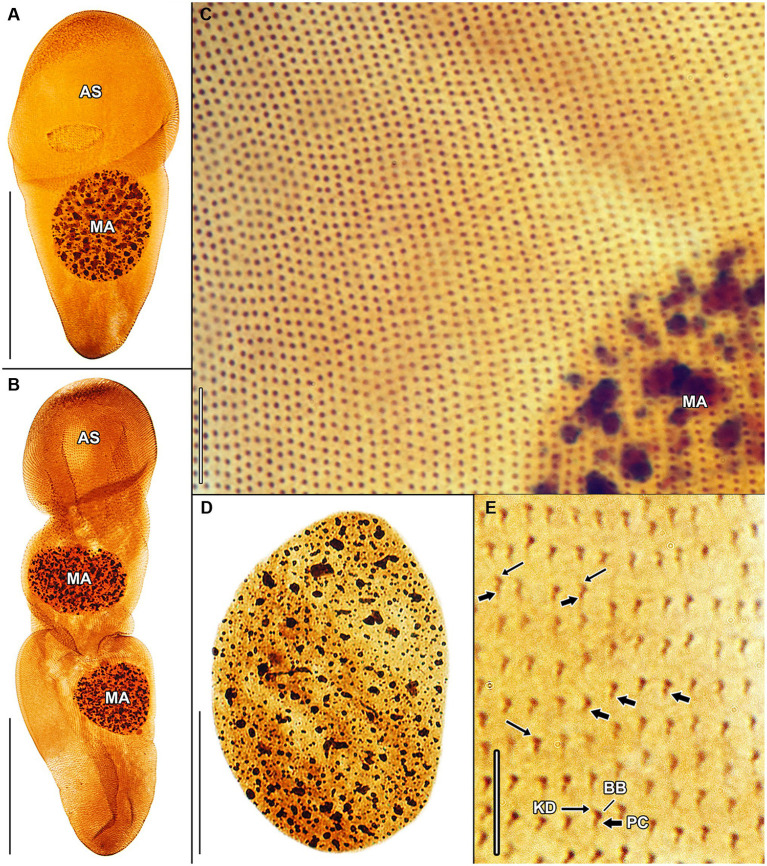
*Haptophrya planariarum*, protargol-impregnated specimens isolated from *Dugesia gonocephala* collected from the Malá Vydrica stream at the locality Železná studnička, Bratislava, Slovakia. **(A)** Ventral view of the adhesive sucker, showing the horseshoe-shaped suture (opposed arrowheads) that runs along the dorsal and lateral borders of the sucker. About 90 ventral kineties run onto the adhesive sucker to abut almost at right angle on the dorsal and lateral kineties along the whole horseshoe-shaped suture line. **(B)** Dorsal view of the adhesive sucker, showing the ciliary pattern. Somatic ciliature is holotrichous. Basal bodies are very densely spaced, i.e., the intrakinetidal distance is about 1.5 μm. Somatic kineties extend meridionally and they are also very narrowly spaced, i.e., the intrakinetal distance is approximately 1 μm. **(C)** Dorsal view of the trunk region, showing the ciliary pattern along the contractile canal. Opposed arrowheads denote the excretory pores of the canal. MA, macronucleus. Scale bars = 10 μm.

**Figure 9:**
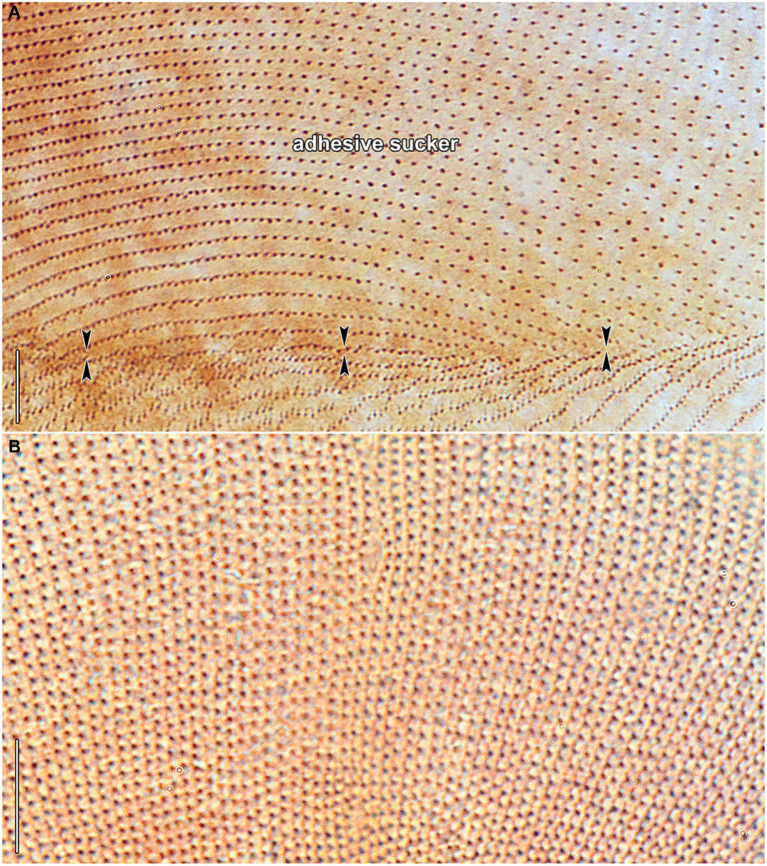
*Haptophrya schmidtearum* nov. spec., protargol-impregnated specimens isolated from *Schmidtea polychroa* collected from the Jurské jazierko pond in the district of the village of Svätý Jur, Malé Karpaty Mts., Slovakia. **(A,B)** Overviews of representative specimens. The body is campanulate to truncate claviform and differentiated into a conspicuous anterior sucker, a more or less distinct neck-like constriction, and a cone-like trunk. **(C)** The macronucleus is ellipsoidal and studded with globular to irregular nucleoli. **(D)** Detail of somatic kineties, showing the fibrillar associates of basal bodies. When kinetodesmal fibers are very deeply impregnated, somatic monokinetids might appear as dikinetids, especially when postciliary microtubules are only weakly impregnated. **(E)** Dorsal view of the trunk region, showing the ciliary pattern along the contractile canal. Opposed arrowheads denote the excretory pores of the canal. AS, adhesive sucker; BB, basal body; KD, kinetodesmal fiber (thin arrow); MA, macronucleus; and PC, postciliary microtubules (thick arrow). Scale bars = 5 μm **(D)**, 10 μm **(E)**, 30 μm **(C)**, and 100 μm **(A,B)**.

**Figure 10:**
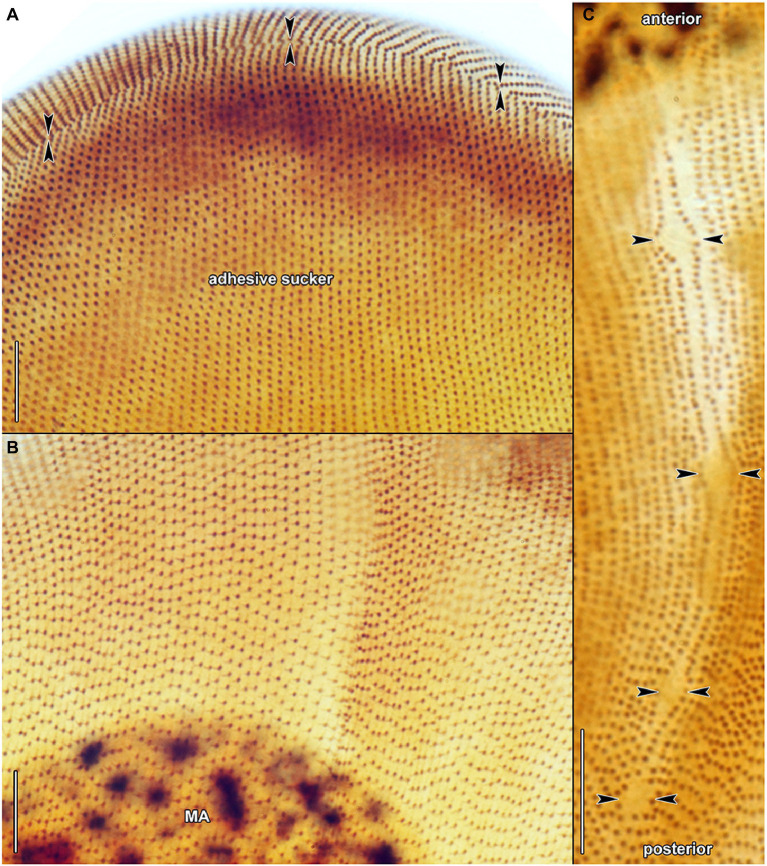
*Haptophrya schmidtearum* nov. spec., protargol-impregnated specimens isolated from *Schmidtea polychroa* collected from the Jurské jazierko pond in the district of the village of Svätý Jur, Malé Karpaty Mts., Slovakia. **(A)** Ventrolateral view, showing the secant system (opposed arrowheads) at the lateral margin of the adhesive sucker, whose ciliary rows abut on the anterior end of trunk ciliary rows. **(B)** Dorsal view of the adhesive sucker, showing the ciliary pattern. Somatic ciliature is holotrichous. Basal bodies are very densely spaced, i.e., the intrakinetidal distance is about 1.5 μm. Somatic kineties extend meridionally and they are also very narrowly spaced, i.e., the intrakinetal distance is approximately 1 μm. Note that kinetodesmal fibers are well-developed, directed anteriorly, and overlapping. They form kinetodesmal ribbons that extend close to the right of the somatic ciliary rows and appear as weakly impregnated lines, as typical of members of the class Oligohymenophorea. Scale bars = 10 μm.

**Figure 11:**
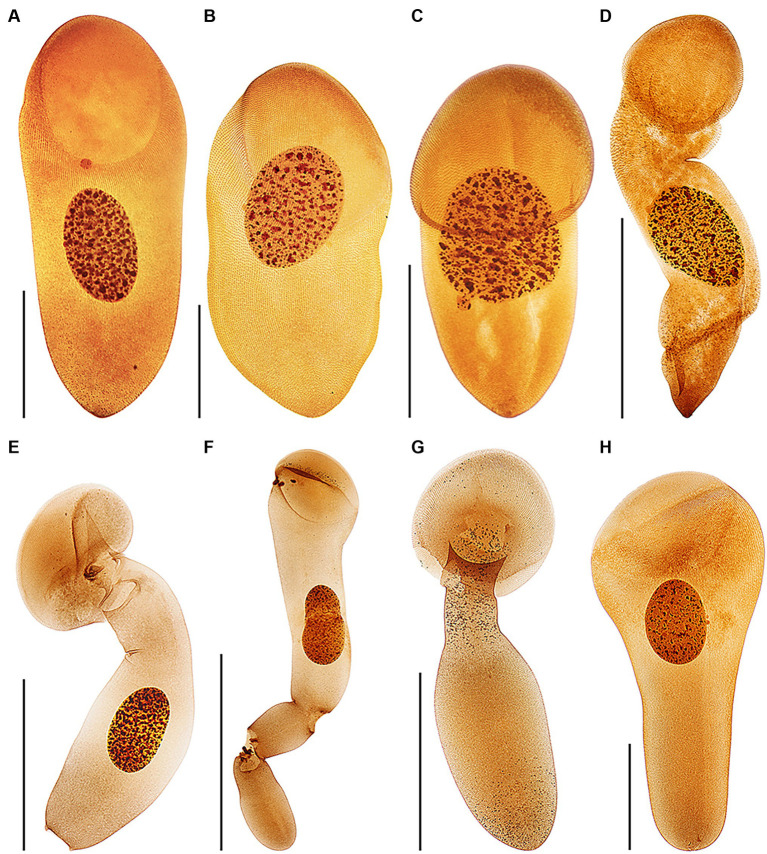
Protargol-impregnated specimens of *Haptophrya planariarum* **(A–D)** and *Haptophrya schmidtearum* nov. spec. **(E–H)**. The typical body shape of *Haptophrya* species is campanulate to truncate claviform and the body is differentiated into a conspicuous anterior sucker, a more or less distinct neck-like constriction, and a cone-like trunk **(A–C,H)**. The macronucleus is broadly to narrowly ellipsoidal and situated in the rear body end in living cells. Preparations artifacts and postmortal changes include elongation of the body, inflation of the anterior body third, and fragmentation of the posterior trunk region **(D,E–G)**. Note that the macronucleus is gradually displaced from the rear end to the anterior body region in prepared cells. Already Schultze (1851) figured these morphological changes in *H. planariarum* specimens isolated from *Planaria torva*. Due to the dramatic changes in the body shape and size as well as in the localization of the nuclear apparatus in some protargol-impregnated specimens, fresh and living cells need to be investigated. Scale bars = 100 μm **(A–C,H)** and 200 μm **(D–G)**.

Haptophryans were isolated from two planarian hosts (*D. gonocephala* and *S. polychroa*) collected at 12 localities. According to molecular data, they belong to three species (*H. planariarum*, *H. dugesiarum*, and *H. schmidtearum*; Figures 1A,B). Nonetheless, they share all taxonomically important qualitative morphological characters (Figures 7A–E, 8A–C, 9A–E, 10A,B, and 11A–H): (i) a campanulate to truncate claviform body differentiated into a conspicuous anterior, shallow sucker, a more or less distinct neck-like constriction, and a cone-like trunk; (ii) the broadly to narrowly ellipsoidal macronucleus is situated in the rear body end in living cells and is accompanied by 1–3 globular micronuclei; (iii) the contractile canal extends along the whole dorsal cell margin; (iv) somatic kineties are built from monokinetids throughout; (v) ciliary rows are meridional and very narrowly spaced, about half of them extends onto the anterior sucker; (vi) the horseshoe-shaped suture runs along the dorsal and lateral borders of the sucker (shown only for *H. planariarum* in Figure 8A, opposed arrowheads); and (vii) two inconspicuous secant systems at lateral ends of the horseshoe-shaped suture (left suture shown only for *H. schmidtearum* in Figure 10A, opposed arrowheads). The general body organization as well as the ciliary pattern of *Haptophrya* is illustrated in detail in our previous study (Rataj and Vďačný, 2018) as well as in Lom (1959) and Corliss et al. (1965). The seeming differences between kinetid structures (Figures 7E, 9E) are caused by different bleaching and impregnation intensities of kinetodesmal fibers and postciliary microtubules. Thus, very deeply impregnated kinetodesmal fibers might cause the somatic monokinetid to appear as dikinetids especially when postciliary microtubules are only weakly impregnated.

The quantitative characters significantly overlap and hence also do not allow discrimination of the three *Haptophrya* species. In mixed populations, the separation of *H. dugesiarum* from *H. planariarum* was impossible with morphological data and both species could be reliably discerned only after sequencing. Therefore, the following morphometric comparison is based only on a voucher population of *H. planariarum* (collected from the Malá Vydrica stream in the Kačínska dolina valley at the locality Železná studnička, Bratislava) and the type population of *H. schmidtearum* (collected from the Jurské jazierko pond in an urban oak-hornbeam forest, district of the village of Svätý Jur, Malé Karpaty Mts.). The “Malá Vydrica” population was selected to avoid mixing of *H. planariarum* and *H. dugesiarum*, as the latter species has been hitherto not detected in SW Slovakia. The body length *in vivo* spans a range of 205–335 μm in *H. planariarum*, while it is about 281 μm in *H. schmidtearum*. The maximum width of the adhesive sucker *in vivo* is 95 μm in *H. planariarum*, while the sucker is slightly wider (up to 131 μm) in *H. schmidtearum*. The maximum trunk width in living cells is 58 μm in *H. planariarum*, while *H. schmidtearum* is slightly broader (about 79 μm). The size of the macronucleus after protargol impregnation is 67–111 × 50–79 μm in *H. planariarum* and 81–104 × 53–63 μm in *H. schmidtearum*. Finally, the number of somatic kineties on one body side is 90–101 in *H. planariarum* and 98–108 in *H. schmidtearum*.

## Discussion

### Cryptic Speciation in *Haptophrya*

Since the times of Ehrenberg (1838) and Stein (1859), it has been assumed that ciliate taxa should differ morphologically. However, there is no law in nature that would require phylogenetically distinct species to be also divergent in phenotype. Phenotypic divergence may be also delayed relative to phylogenetic divergence for multiple reasons: (i) species may have diverged too recently to have also diverged in phenotype, (ii) selective forces at work relative to a particular phenotype, and (iii) simple body plans offer only a little evidence for phenotypic divergence except for difficult to perceive ultrastructural or physiological features (Lücking et al., 2021). With the application of multiple molecular markers (especially the mitochondrial 16S rRNA and COI genes), cryptic or near-cryptic speciation (subtle, easily overlooked differences, or only statistically supported differences) has been detected in a variety of symbiotic ciliates. For instance, in clevelandellids associated with wood-feeding cockroaches (Pecina and Vďačný, 2020, 2021), in histophagous tetrahymenids parasitizing mollusks (Zhang and Vďačný, 2021), in astomes occupying the digestive tube of lumbricid earthworms (Obert and Vďačný, 2019, 2020; Obert et al., 2021), and in mobilids living on the surface of freshwater planarians (Rataj and Vďačný, 2021). Cryptic or near-cryptic speciation has apparently occurred also in haptophryans associated with freshwater planarians, as documented by the huge differences in the primary structure (interspecies *p*-distances range from 11.03 to 17.03%, while maximum intraspecies distances are only 0.26%), the secondary structure of the V4, V7, and V9 regions (Figure 4; Supplementary Figures S6, S7) as well as by 9–12 CBCs in the 16S rRNA molecule. Although no distinct qualitative or quantitative morphological differences were detected, the molecular differences among the phylogenetically delimited *Haptophrya* species are so huge that they cannot be interpreted as intra-species variability of *H. planariarum*. *Haptophrya schmidtearum* might be considered as a near-cryptic species, as it can be separated from *H. planariarum* and *H. dugesiarum* by the planarian host (the pond-dwelling *S. polychroa* vs. the stream- and river-dwelling *D. gonocephala*). Interestingly, the two latter *Haptophrya* species share the same host, which indicates a duplication event without host switching. This diversification mode was suggested also for astomes inhabiting megascolecid and glossoscolecid earthworms (Obert et al., 2021). In the absence of phenotypic divergence and diversification driven by duplication events with or without host switching, we prefer to employ molecular data to distinguish the three *Haptophrya* species. We anticipate that the current reluctance against the acceptance of phenotypically cryptic species (Warren et al., 2017) will diminish, as molecular tools have become more easily accessible and molecular delimitation of ciliate species has been successfully applied to multiple free-living (e.g., Doerder, 2019; Greczek-Stachura et al., 2021 and references cited therein) and symbiotic ciliates (e.g., Obert et al., 2021; Pecina and Vďačný, 2021). Finally, it is important to mention that the identity of *H. planariarum* populations reported from the Nearctic and Paleotropis is highly questionable, as the species was discovered in the Palearctic (for a review, see Rataj and Vďačný, 2018) and planarian species have biogeographies. Likewise, the conspecificity of populations isolated from various planarian hosts, belonging to the families Planariidae Stimpson, 1857 (*Planaria* Müller, 1776), Dendrocoelidae Hallez, 1892 (*Dendrocoelum* Ørsted, 1844), and Dugesiidae Ball, 1974 (*Dugesia* Girard, 1850 and *Schmidtea* Ball, 1974), is very doubtful in light of the present findings. Molecular data, especially 16S sequences, are indispensable to revealing their true identities.

### Phylogenetic Relationships and Systematic Position of *Haptophrya*

*Haptophrya* was traditionally classified within the order Astomatida Schewiakoff, 1896 of the subclass Astomatia due to the complete lack of oral structures (Cépède, 1910; de Puytorac, 1957, 1963; Lom, 1959; Corliss et al., 1965). *Annelophrya* Lom, 1959, *Cepedietta* Kay, 1942, *Lachmanella*, Cépède, 1910, and *Steinella* Cépède, 1910 were considered the nearest relatives of *Haptophrya* and were assigned to the family Haptophryidae Cépède, 1923 in compendium of Lynn (2008). Since these genera differ by their attachment strategies (adhesive sucker vs. hooks), division mode (binary fission vs. chain formation), and host organisms (planarians vs. amphibians), they were divided into three subfamilies. The nominotypical subfamily Haptophryinae Cépède, 1923 (*Haptophrya*) occupies exclusively the digestive tract of freshwater planarians and is characterized by the absence of hooks and binary fission without chain formation. The subfamily Cepediettinae Corliss et al., 1965 unites endosymbionts of frogs and newts. They also attach to the host’s intestine employing an adhesive sucker but they form chains during reproduction. Finally, members of the subfamily Lachmannellinae Cépède, 1923 live in freshwater (*Annelophrya*) and marine (*Lachmannella* and *Steinella*) planarians. They attach to their digestive tract with a single hook (*Lachmannella*), two hooks of unequal size (*Steinella*), or with numerous, small spines surrounding the thigmotactic area (*Annelophrya*). No chain formation has been noted during the binary fission of lachmannellids. These profound morpho-ecological differences question the close relationship of cepediettids and lachmannellids as well as their assignment to the family Haptophryidae. Due to the lack of molecular data, their relatedness with *Haptophrya* and systematic positions remain unknown.

Lom (1959) suggested a close kinship of *Haptophrya* and *Clausilocola* Lom, 1959 due to their similarities in body shape and the presence of anterior adhesive sucker. Their monophyletic origin was, however, rejected in the light of both detailed morphological and molecular analyses (Zhang and Vďačný, 2022). Specifically, *Haptophrya* displays a secant system at each lateral end of the horseshoe-shaped suture and does not have dikinetids and unciliated apical field (Corliss et al., 1965; Rataj and Vďačný, 2018; present study). By contrast, *Clausilocola* possesses a single subapical secant system in the midline of the ventral side, an extensive unciliated apical area, and numerous dikinetids at the top of all ciliary rows. Unrelatedness of *Haptophrya* and *Clausilocola* was recognized also by the previous (Zhang and Vďačný, 2022) and present (Figures 5, 6) phylogenetic analyses, as both genera were nested in different oligohymenophorean subclasses: *Haptophrya* in the Scuticociliatia Small, 1967 and *Clausilocola* in the Hymenostomatia Delage and Hérouard, 1896.

The classification of *Haptophrya* in the subclass Astomatia was challenged already by the first 18S rRNA gene analyses (Rataj and Vďačný, 2018). *Haptophrya* was placed in a sister position to the free-living scuticociliate genus *Dexiotricha* Stokes, 1885, and this relationship was corroborated also by two shared unique nucleotide positions. Later on, the orphan scuticociliate genus *Conchophthirus* Stein, 1861, which inhabits the mantle cavity of freshwater bivalves, was added to this clade (Antipa et al., 2020). Phylogenetic relationships among *Haptophrya*, *Dexiotricha*, and *Conchophthirus* remained, however, unresolved in 18S phylogenies (Antipa et al., 2020; Zhang and Vďačný, 2022). Despite this, *Haptophrya* has been never found as a close relative of astomes isolated from the digestive tube of annelids (Obert and Vďačný, 2019, 2021; Obert et al., 2021). The primary and secondary structures of the mitochondrial 16S rRNA molecule also do not support their close relationships. The amplified part of the 16S rRNA gene is 864–888 nt long in the “core” astomes (Obert et al., 2021), while 1,167–1,172 nt long in haptophryans (present study). This conspicuous length difference is caused by deletions/insertions, especially in helices 21 and 23 from the V4 region, helix 33 from the V6 region, helices 39 and 40 from the V7 region, and helix 44 from the V9 region. Especially, the structure of helix 40 is quite different among astomes, “core” scuticociliates, and haptophryans. Helix 40 of *Haptophrya* carries two extra hairpins (40es9a and 40es9b), which are absent in astomes (Obert et al., 2021), “core” scuticociliates (Zhang et al., 2019), peniculines and hymenostomes (Cannone et al., 2002). Whether these insertions are specific for *Haptophrya* or shared with *Dexiotricha* and *Conchophthirus* needs to be analyzed in the future when 16S sequences become available for the two latter genera. On the other hand, the length and structure of the astome 16S rRNA molecules correspond rather well to those of the “core” scuticociliates (Zhang et al., 2019), peniculines, and hymenostomes (Cannone et al., 2002). Based on the multi-gene phylogenies as well as the primary and the secondary structure of the 16S rRNA molecules, we proclaim that the “core” astomes are more closely related to the “core” scuticociliates than to *Haptophrya*. Very likely, *Haptophrya* evolved from an orphan scuticociliate lineage comprising also *Dexiotricha* and *Conchophthirus* by the loss of oral apparatus and by the transformation of the thigmotactic field into an adhesive sucker. Since astomy evolved at least three times independently (in *Haptophrya*, *Clausilocola*, and “core” astomes) within the class Oligohymenophorea (Zhang and Vďačný, 2022; present study), the loss of cell mouth cannot be used as a sole argument for the assignment of haptophryans to the subclass Astomatia anymore.

## Taxonomic Summary

We use molecular data to diagnose the two new *Haptophrya* species because morphological data do not allow their unambiguous separation. We interpret the isolated DNA as type material of the new species, which is in accordance with Article 72.5.1 of the International Commission on Zoological Nomenclature (1999). The reference alignments are provided in Supplementary Material. The primary and secondary structures of the 16S and 18S molecules are shown in Figures 2–4 and Supplementary Figures S2–S7.

### Zoobank Registration Number of Work

urn:lsid:zoobank.org:pub:3949D77D-194C-42A7-8BBE-687C52451603.

Phylum Ciliophora Doflein, 1901
Class Oligohymenophorea de Puytorac et al., 1974
*Incertae sedis* in Subclass Scuticociliatia Small, 1967
Family Haptophryidae Cépède, 1923
Genus *Haptophrya* Stein, 1867 (type species: *Opalina planariarum* von Siebold, 1839)

#### *Haptophrya dugesiarum* Nov. Spec.

##### Zoobank Registration Number of New Species

urn:lsid:zoobank.org:act:1CAD60AE-A806-4C2B-9119-7AFBC27EACE5.

###### Diagnosis

16S rRNA gene: 55 G, 62 T, 63 A, 68 T, 73 G, 82 T, 92 G, 110 G, 118 G, 120 G, 167 A, 169 C, 180 C, 181 A, 183 A, 424 C, 426 T, 446 G, 453 C, 454 T, 457 C, 521 A, 535 G, 559 A, 568 A, 573 C, 581 C, 587 -, 597 C, 598 G, 617 G, 629 C, 630 C, 632 A, 637 -, 642 A, 643 A, 649 A, 651 T, 652 G, 663 T, 664 T, 665 A, 666 T, 667 T, 668 T, 673 G, 674 G, 683 T, 686 A, 688 T, 701 T, 702 A, 707 T, 711 A, 730 A, 731 T, 733 A, 738 A, 745 C, 746 C, 749 A, 751 A, 754 G, 755 G, 757 C, 762 T, 771 A, 772 A, 783 A, 790 A, 793 T, 796 C, 799 A, 800 A, 802 A, 805 C, 807 A, 808 A, 811 A, 817 G, 824 -, 825 -, 831 C, 837 G, 840 T, 925 A, 930 T, 1085 A, 1102 A, 1108 -, 1114 A, 1118 A, 1122 T, 1128 G, 1144 T, 1149 T, 1152 G, 1162 C, 1164 G, and 1175 A. 18S rRNA gene: 486 T, 1320 A.

###### Type Locality

An unnamed stream in the Fončorda residential area, Banská Bystrica, Zvolenská kotlina basin, Slovakia (48°43′21.4″N, 19°06′58.3″E).

###### Type Host

*Dugesia gonocephala* (Dugès, 1830) Girard, 1850.

###### Type Material

A DNA sample of holotype specimen has been deposited in Natural History Museum, Vajanského nábrežie 2, 810 06 Bratislava, Slovakia (ID Collection Code 01427586).

###### Gene Sequences

The 16S and 18S rRNA gene sequences of the holotype specimen have been deposited in GenBank under the following accession nos. OL752569 and OL752521, respectively.

###### Etymology

The specific epithet is a plural genitive case of the Neo-Latin noun *Dugesi*·*a*, *ae* [f] (generic name of freshwater planarians), meaning a *Haptophrya* from dugesians. The species-group name is to be treated as an adjective used as a substantive in the genitive case, because of its derivation from the host’s generic name (Article 11.9.1.4. of the International Commission on Zoological Nomenclature, 1999).

#### *Haptophrya schmidtearum* Nov. Spec.

##### Zoobank Registration Number of New Species

urn:lsid:zoobank.org:act:EF237EA8-DBC2-4E7C-94A7-00CCF191F284.

###### Diagnosis

16S rRNA gene: 39 C, 41 T, 53 T, 54 T, 56 A, 57 C, 58 C, 59 G, 60 A, 61 -, 62 -, 68 A, 70 A, 73 A, 81 T, 92 -, 96 G, 106 A, 108 C, 111 A, 116 A, 152 C, 157 A, 170 G, 182 T, 189 G, 191 T, 278 T, 282 G, 428 C, 436 A, 438 G, 444 A, 447 T, 452 -, 454 A, 455 T, 457 T, 464 A, 515 C, 532 C, 547 C, 552 G, 554 G, 555 G, 558 C, 559 G, 564 A, 565 C, 567 G, 570 C, 574 T, 577 A, 578 C, 591 G, 597 -, 598 -, 601 T, 604 G, 606 A, 609 C, 612 C, 617 T, 620 A, 622 A, 628 -, 631 -, 637 A, 638 A, 639 A, 644 T, 645 T, 650 G, 651 G, 659 A, 660 C, 661 C, 664 -, 669 A, 671 C, 678 G, 680 T, 682 A, 683 G, 684 C, 685 G, 686 G, 692 A, 695 C, 697 C, 698 A, 700 A, 704 C, 705 A, 706 A, 708 A, 709 C, 711 G, 712 C, 713 C, 714 C, 715 C, 716 C, 719 C, 720 A, 722 T, 724 C, 725 C, 730 -, 733 C, 734 C, 735 T, 736 T, 737 G, 738 G, 739 T, 743 C, 744 C, 751 -, 754 -, 755 -, 756 -, 761 -, 773 A, 774 A, 775 A, 776 G, 777 G, 780 T, 781 T, 782 T, 784 G, 785 G, 788 T, 790 G, 791 C, 792 G, 795 G, 797 A, 799 T, 800 T, 805 G, 808 T, 812 T, 814 A, 816 C, 819 A, 824 T, 825 T, 827 G, 829 T, 837 -, 839 A, 844 G, 908 A, 920 G, 928 C, 931 A, 933 G, 934 T, 943 T, 957 T, 979 G, 1021 T, 1087 T, 1098 A, 1100 A, 1103 T, 1106 -, 1112 -, 1113 C, 1114 -, 1115 -, 1120 G, 1122 A, 1125 -, 1127 A, 1129 -, 1133 G, 1134 T, 1135 T, 1141 A, 1142 A, 1144 A, 1145 C, 1148 C, 1149 G, 1150 G, 1151 T, 1154 C, 1155 -, 1157 G, 1160 C, 1164 -, 1168 G, 1178 G, and 1180 A. 18S rRNA gene: 639 A, 640 T, 645 T, 661 G, 666 A, 680 T, 691 T, 704 A, 723 A, 725 C, 726 C, 766 A, 1336 -, 1349 T, 1370 T, 1463 A, and 1660 G.

###### Type Locality

Jurské jazierko pond in an urban oak-hornbeam forest, district of the village of Svätý Jur, Malé Karpaty Mts. (Little Carpathians), Slovakia (48°15′28.0″N, 17°09′14.6″E).

###### Type Host

*Schmidtea polychroa* (Schmidt, 1861) Ball, 1974.

###### Type Material

A DNA sample of holotype specimen has been deposited in Natural History Museum, Vajanského nábrežie 2, 810 06 Bratislava, Slovakia (ID Collection Code 01427587).

###### Gene Sequences

The 16S and 18S rRNA gene sequences of the holotype specimen have been deposited in GenBank under the following accession nos. OL752574 and OL752526, respectively.

###### Etymology

The specific epithet is a plural genitive case of the Neo-Latin noun *Schmidte*·*a*, *ae* [f] (generic name of freshwater planarians), meaning a *Haptophrya* from schmidteans. The species-group name is to be treated as an adjective used as a substantive in the genitive case, because of its derivation from the host’s generic name (Article 11.9.1.4. of the International Commission on Zoological Nomenclature, 1999).

#### *Haptophrya planariarum* (von Siebold, 1839) Stein, 1867

Nomenclature and taxonomy of *H. planariarum* were reviewed by Rataj and Vďačný (2018). The species was neotypified by Corliss et al. (1965). However, at the present state of knowledge, the morphological data do not enable unambiguous identification of this species and gene sequences are not available from the neotype. To promote nomenclatural and taxonomic stability and correct usage of the name *H. planariarum*, we provide below a molecular diagnosis based on the barcoding 16S rRNA gene, voucher material, and gene sequences from the voucher specimen.

##### Molecular Diagnosis

16S rRNA gene: 36 T, 42 T, 46 C, 62 G, 66 A, 68 G, 73 T, 84 G, 92 A, 115 G, 129 T, 147 G, 153 A, 200 A, 279 G, 280 C, 286 -, 321 G, 433 C, 445 G, 454 C, 457 A, 458 G, 480 C, 485 T, 533 A, 559 -, 561 G, 563 G, 596 A, 597 T, 598 T, 599 T, 600 T, 605 A, 617 A, 625 G, 626 G, 627 T, 635 G, 637 T, 641 T, 647 G, 648 T, 651 A, 664 C, 681 G, 683 C, 686 -, 693 -, 699 -, 710 T, 711 -, 726 G, 727 G, 728 G, 730 C, 733 G, 738 T, 748 C, 751 G, 753 C, 754 T, 755 A, 759 G, 767 T, 768 T, 769 T, 770 T, 790 T, 794 G, 799 C, 800 G, 805 T, 808 G, 822 T, 824 C, 825 A, 834 T, 837 A, 841 G, 842 A, 923 A, 944 C, 960 T, 989 G, 1081 A, 1104 C, 1105 T, 1109 T, 1114 G, 1122 G, 1139 G, 1144 C, 1146 G, 1149 A, 1159 A, 1164 A, 1176 C, and 1190 G.

##### Voucher Material

A DNA sample of a voucher specimen has been deposited in Natural History Museum, Vajanského nábrežie 2, 810 06 Bratislava, Slovakia (ID Collection Code 01427588). The voucher originated from the Malá Vydrica stream in the Kačínska dolina valley at the locality Železná studnička, Bratislava (48°12′05.9″N, 17°04′34.7″E).

##### Gene Sequences

The 16S and 18S rRNA gene sequences of the voucher specimen have been deposited in GenBank under the following accession nos. OL752528 and OL752480, respectively.

## Data Availability Statement

The data presented in the study are deposited in the GenBank database (https://www.ncbi.nlm.nih.gov/nucleotide/), accession numbers OL752480 to OL752575. Results of all analyses are included in this published article and Supplementary Material. GenBank accession numbers of sequences used in phylogenetic analyses can be found in the Supplementary Material.

## Author Contributions

PV conceptualized the research. MR and TZ performed the laboratory work. PV and MR prepared and analyzed the data, created visualizations, and wrote the original draft of the manuscript. All authors contributed to the article and approved the submitted version.

## Funding

This work was supported by the Slovak Research and Development Agency under contract No. APVV-19-0076, by the Grant Agency of the Ministry of Education, Science, Research and Sport of the Slovak Republic and Slovak Academy of Sciences under the Grant VEGA 1/0013/21, as well as by the Comenius University in Bratislava under the Grants UK/28/2019 and UK/160/2020.

## Conflict of Interest

The authors declare that the research was conducted in the absence of any commercial or financial relationships that could be construed as a potential conflict of interest.

## Publisher’s Note

All claims expressed in this article are solely those of the authors and do not necessarily represent those of their affiliated organizations, or those of the publisher, the editors and the reviewers. Any product that may be evaluated in this article, or claim that may be made by its manufacturer, is not guaranteed or endorsed by the publisher.

## Footnotes

^1^ http://apollo.chemistry.gatech.edu/RibosomeGallery

^2^ http://rna.urmc.rochester.edu/RNAstructureWeb

^3^ http://www.unafold.org/

^4^ http://rnacomposer.cs.put.poznan.pl

^5^ http://rnapdbee.cs.put.poznan.pl

^6^ http://mafft.cbrc.jp/alignment/server/

^7^ http://molevol.cmima.csic.es/castresana/Gblocks_server.html

^8^ http://iqtree.cibiv.univie.ac.at/

^9^ http://tree.bio.ed.ac.uk/software/figtree/
