# Supplementary material for: Nuclear and Mitochondrial SSU rRNA Genes Reveal Hidden Diversity of Haptophrya Endosymbionts in Freshwater Planarians and Challenge Their Traditional Classification in Astomatia
Source: Front Microbiol. 2022 Apr 14;13:830951. doi: 10.3389/fmicb.2022.830951 (PMC9048206; doi:10.3389/fmicb.2022.830951)
Supplement: Supplementary file 1 [file Data_Sheet_1.PDF]

# SUPPLEMENTARY MATERIAL

# Nuclear and mitochondrial SSU rRNA genes reveal hidden diversity of *Haptophrya* endosymbionts in freshwater planarians and challenge their traditional classification in Astomatia

***Matej Rataj, Tengyue Zhang, Peter Vďačný****

*Department of Zoology, Faculty of Natural Sciences, Comenius University in Bratislava, Bratislava, Slovakia*

***Correspondence:**
*Peter Vďačný*
*peter.vdacny@uniba.sk*

---

**Pages:** **42**

**Tables:** **5**

**Figures:** **7**

**Alignments:** **2**

**References:** **12**

**SUPPLEMENTARY TABLE S1 |** Characterization of collection sites of planarian species examined for the presence of *Haptophrya*.

| Collection date (m/d/y) | Site code | Collection site | Water body | GPS coordinates | Host species | No. of speci-mens |
|---|---|---|---|---|---|---|
| 10/01/2016 | 1 | Municipal forest park, Kačínska dolina valley, Železná studnička, Bratislava, Malé Karpaty Mts. (Little Carpathians) | Malá Vydrica stream | 48°12'05.9"N, 17°04'34.7"E | *Dugesia gonocephala* | 22 |
| 10/01/2016 | 2 | Municipal forest park, Drieňovská lúka meadow, Železná studnička, Bratislava, Malé Karpaty Mts. (Little Carpathians) | Drieňovská dráha stream | 48°11'39.3"N, 17°05'50.8"E | *Dugesia gonocephala* | 28 |
| 10/08/2016 | 3 | Spruce forest, Olešná Potôčky, district of the village of Olešná, Turzovská vrchovina highlands | Olešňanský potok stream | 49°26'08.7"N, 18°38'39.6"E | *Dugesia gonocephala* | 11 |
| 10/08/2016 | 4 | Inundation area in the vicinity of the village of Milošová, Turzovská vrchovina highlands | Milošovanský potok stream | 49°28'17.1"N, 18°45'18.4"E | *Dugesia gonocephala* | 9 |
| 10/22/2016 | 5 | Drieňovecká mokraď swamp, Slovenský kras (Slovak Karst) National Park, Slovenské Rudohorie (Slovak Ore Mts.) | Bercz spring area | 48°36'54.7"N, 20°54'54.8"E | *Polycelis felina* | 74 |
| 10/29/2016 | 6 | Urban oak-hornbeam forest, Pekná cesta, Bratislava, Malé Karpaty Mts. (Little Carpathians) | Vajspeterský potok stream | 48°12'47.3"N, 17°07'21.1"E | *Dugesia gonocephala* | 11 |
| 10/29/2016 | 7 | Urban oak-hornbeam forest, Knižková dolina valley, Bratislava, Malé Karpaty Mts. (Little Carpathians) | Banský potok stream | 48°13'08.8"N, 17°07'58.8"E | *Dugesia gonocephala* | 10 |
| 11/05/2016 | 8 | Urban oak-hornbeam forest, Medené Hámre, district of the village of Borinka, Malé Karpaty Mts. (Little Carpathians) | Forest rainwater pool | 48°16'03.1"N, 17°07'16.0"E | *Dugesia gonocephala* | 11 |
| 11/05/2016 | 9 | Municipal park, Tovarníky, district of the town of Topoľčany, Nitrianska pahorkatina highlands | Tovarníky fishpond | 48°34'07.9"N, 18°08'45.9"E | *Girardia tigrina* | 28 |
| 11/12/2016 | 10 | Regulated stream in the town of Rajecké Teplice, Rajecká kotlina basin, Malá Fatra Mts. | Bystrička stream | 49°07'21.4"N, 18°41'16.9"E | *Dugesia gonocephala* | 8 |
| 11/19/2016 | 11 | Pine-spruce forest, district of the village of Čadečka, Kysucké Beskydy Mts. | Čadečanský potok stream | 49°27'54.9"N, 18°49'02.9"E | *Dugesia gonocephala* | 10 |
| 11/16/2018 | 12 | Urban oak-hornbeam forest, Pekná cesta, Bratislava, Malé Karpaty Mts. (Little Carpathians) | Vajspeterský rybník fishpond | 48°12'20.8"N, 17°07'42.4"E | *Girardia tigrina* | 10 |
| 11/25/2018 | 13 | Recreation area in the village of Veľký Biel, Podunajská rovina plain | Veľkobielske jazero lake | 48°12'26.4"N, 17°21'28.3"E | *Girardia tigrina* | 34 |
| 12/02/2018 | 14 | Recreation area in the village of Rovinka, Podunajská rovina plain | Rovinské štrkovisko lake | 48°05'57.2"N, 17°13'34.4"E | *Girardia tigrina* | 48 |
| 12/06/2018 | 15 | Eutrophic fishpond, Východné, Bratislava, Podunajská rovina plain | Kalné jazero fishpond | 48°11'36.7"N, 17°10'39.2"E | *Girardia tigrina* | 48 |
| 12/06/2018 | 16 | Municipal recreation area, Bratislava, Podunajská rovina plain | Zlaté Piesky lake | 48°11'04.0"N, 17°11'11.0"E | *Girardia tigrina* | 118 |
| 12/08/2018 | 17 | Alluvial forest, Rusovce, Bratislava, Podunajská rovina plain | Rusovecké jazero lake | 48°03'45.9"N, 17°09'16.5"E | *Girardia tigrina* | 70 |
| 12/16/2018 | 18 | Municipal recreation area, district of the village of Dunajská Lužná, | Malé Košariská lake | 48°05'50.5"N, 17°16'44.0"E | *Girardia tigrina* | 40 |

| | | | | | | |
|---|---|---|---|---|---|---|
| | | Podunajská rovina plain | | | | |
| 02/09/2019 | 19 | Artificial lake, Vajnory, Bratislava, Podunajská rovina plain | Vajnorské jazero lake | 48°11'35.3"N, 17°12'34.9"E | *Dendrocoelum lacteum/ Schmidtea lugubris* | 8/6 |
| 02/17/2019 | 20 | Residential area Fončorda, Banská Bystrica, Zvolenská kotlina basin | Unnamed stream | 48°43'21.4"N, 19°06'58.3"E | *Dugesia gonocephala* | 20 |
| 03/07/2019 | 21 | Shallow river section near the village of Stankovany, Veľká Fatra Mts. | Váh river | 49°08'26.3"N, 19°10'14.6"E | *Dugesia gonocephala* | 11 |
| 03/07/2019 | 22 | Shallow river section near the village of Bystrá, Veľká Fatra Mts. | Váh river | 49°05'04.8"N, 19°17'07.7"E | *Dugesia gonocephala* | 18 |
| 03/07/2019 | 23 | Pine-spruce forest, Hrabovská dolina valley, Veľká Fatra Mts. | Hrabovský potok stream | 49°04'15.0"N, 19°16'22.2"E | *Polycelis felina* | 61 |
| 03/07/2019 | 24 | Pine-spruce forest, Čutkovská dolina valley, Veľká Fatra Mts. | Čutkovský potok stream | 49°04'53.7"N, 19°15'19.6"E | *Polycelis felina* | 31 |
| 03/23/2019 | 25 | Urban oak-hornbeam forest, district of the village of Svätý Jur, Malé Karpaty Mts. (Little Carpathians) | Jurské jazierko pond | 48°15'28.0"N, 17°09'14.6"E | *Schmidtea polychroa/ Polycelis nigra* | 6/1 |
| 04/06/2019 | 26 | Artificial pond, Východné, Bratislava, Podunajská rovina plain | Unnamed pond | 48°12'08.7"N, 17°09'51.9"E | *Girardia tigrina* | 28 |
| 05/09/2019 | 27 | Bielovodská dolina valley, Vysoké Tatry Mts. (High Tatra) | Biela voda river | 49°14'29.9"N, 20°06'04.7"E | *Crenobia alpina* | 4 |
| 10/24/2019 | 28 | Very slowly running river section near the village of Brehy, Žarnovica district, Štiavnické vrchy Mts. | Hron river | 48°24'25.5"N, 18°38'47.2"E | *Girardia tigrina* | 2 |

**SUPPLEMENTARY TABLE S2 |** Primers used for amplification of molecular markers analyzed in *Haptophrya* and their planarian hosts.

| Molecular marker | Organism group | Primer name | Primer sequence (in 5' to 3' direction) | Reference |
|---|---|---|---|---|
| 18S rRNA gene | *Haptophrya* | Euk A | AAC CTG GTT GAT CCT GCC AGT | Medlin et al. (1988) |
| | | Euk B | TGA TCC TTC TGC AGG TTC AC | Medlin et al. (1988) |
| 16S rRNA gene | *Haptophrya* | 16S-mtSSU-F | TGT GCC AGC AGC CGC GGT AA | van Hoek et al. (2000) |
| | | 16S-mtSSU-R | CCC MTA CCR GTA CCT TGT GT | van Hoek et al. (2000) |
| ITS1-5.8S-ITS2 region | *Haptophrya* | ITS1 | TCC GTA GGT GAA CCT GCG G | White et al. (1990) |
| | | ITS4 | TCC TCC GCT TAT TGA TAT GC | White et al. (1990) |
| | | ITS-F | GTA GGT GAA CCT GCG GAA GGA TCA TTA | Miao et al. (2008) |
| | | LO-R | GCT ATC CTG AGR GAA ACT TCG | Pawlowski (2000) |
| | | 28S1R | GTG TTT CAA GAC GGG TCG | Wang et al. (2019) |
| Cytochrome *c* oxidase subunit I | *Haptophrya* | F199dT-A | TGT AAA ACG ACG GCC AGT TCA GGW GCT GCA HTA GC | Lynn and Strüder-Kypke (2006) |
| | | F298dT | TGT AAA ACG ACG GCC AGT GCN CAY GGT YTA ATN ATG GT | Strüder-Kypke and Lynn (2010) |
| | | F388dT | TGT AAA ACG ACG GCC AGT GGW KCB AAA GAT GTW GC | Strüder-Kypke and Lynn (2010) |
| | | R1143dT | CAG GAA ACA GCT ATG ACT ART ATA GGA TCM CCW CCA TAA GC | Strüder-Kypke and Lynn (2010) |
| | | R1184dT | CAG GAA ACA GCT ATG ACT ADA CYT C | Strüder-Kypke and Lynn (2010) |
| | Planarians | COIpr-a2 | AGC TGC AGT TTT GGT TTT TTG GA | Bessho et al. (1992) |
| | | COIpr-b2 | ATG AGC AAC AAC ATA ATA AGT ATC ATG | Bessho et al. (1992) |

**SUPPLEMENTARY TABLE S3 |** Conditions of PCR reactions used for amplification of five molecular markers analyzed in astome ciliates and their planarian hosts.

| Molecular marker | Organism group | PCR program | | | Reference |
|---|---|---|---|---|---|
| | | **Initial denaturation** | **Cycling (denaturation, annealing, extension)** | **Final extension** | |
| 18S rRNA gene | *Haptophrya* | 95 °C/15 min | 30 cycles: 95 °C/45 s, 55 °C/60 s, 72 °C/150 s | 72 °C/10 min | Vďačný et al. (2011) |
| 16S rRNA gene | *Haptophrya* | 94 °C/3 min | 5 cycles: 94 °C/30 s, 50 °C/60 s, 68 °C/75 s<br>35 cycles: 94 °C/30 s, 60 °C/60 s, 68 °C/75 s | 68 °C/10 min | Rataj and Vďačný (2020) |
| Cytochrome *c* oxidase subunit I (COI) | Planarians | 94 °C/4 min | 5 cycles: 94 °C/45 s, 45 °C/75 s, 72 °C/90 s<br>35 cycles: 94 °C/45 s, 55 °C/75 s, 72 °C/90 s | 72 °C/8 min | Rataj and Vďačný (2020) |

**SUPPLEMENTARY TABLE S4 |** List of taxa with corresponding GenBank accession numbers of 18S and 16S rRNA gene sequences included in phylogenetic analyses.

| Taxon | 18S rRNA gene | 16S rRNA gene |
|---|---|---|
| *Anoplophrya allolobophorae* JA3 37 ACH | MZ048824 | MZ048789 |
| *Anoplophrya aporrectodeae* PUz 17 AT | MZ048825 | MZ048790 |
| *Anoplophrya lumbrici* RZ 6 LT | MN121062 | MZ048798 |
| *Anoplophrya lumbrici* KR 9 LT | MZ048831 | MZ048796 |
| *Anoplophrya octolasioni* MU 56 OL | MZ048828 | MZ048793 |
| *Anoplophrya vulgaris* BZ 13 EF | MZ048834 | MZ048804 |
| *Anoplophrya vulgaris* NG 27 DV | MZ048833 | MZ048802 |
| *Clausilocola apostropha* JJ 78 CP | MZ825342 | MZ825327 |
| *Cohnilembus verminus* | HM236339 | MH577598 |
| *Colpoda lucida* | EU039895 | HM246409 |
| *Colpoda magna* | EU039896 | HM246410 |
| *Cristigera pleuronemoides* | KF256816 | MH577581 |
| *Dexiostoma campylum* | X56532 | KY218544 |
| *Dexiostoma sabulum* | KY218611 | KY218549 |
| *Dexiostoma saccharum* | KY218610 | KY218546 |
| *Eurystomatella sinica* | JX310021 | MH577601 |
| *Falcicyclidium ploumeuri* | KF256819 | MH577586 |
| *Frontonia magna* | FJ876953 | KX302681 |
| *Glaucoma chattoni* | X56533 | KY218552 |
| *Glaucoma sp.* | KY218621 | KY218553 |
| *Haptophrya planariarum* KD 1 DG | OL752480 | OL752528 |
| *Haptophrya planariarum* BB 78 DG | OL752521 | OL752569 |
| *Haptophrya planariarum* BY 142 DG | OL752525 | OL752573 |
| *Haptophrya planariarum* JJ 110 SP | OL752526 | OL752574 |
| *Hippocomos salinus* | JX310012 | MH577579 |
| *Histiobalantium minor* | JX310013 | MH577583 |
| *Maryna umbrellata* | JF747217 | JQ026523 |
| *Mesanophrys carcini* | JN885085 | MH577582 |
| *Metanophrys orientalis* | JN885084 | MH577589 |
| *Metanophrys sinensis* | HM236336 | MH577600 |
| *Metaradiophrya chlorotica* JA2 1M ACH | MZ048835 | MZ048805 |
| *Metaradiophrya lumbrici* JA2 25 LT | MN121068 | MZ048808 |
| *Metaradiophrya lumbrici* KR 8 LT | MN121070 | MZ048810 |
| *Metaradiophrya speculorum* HkD 60 AT | MW182013 | MZ048821 |
| *Metaradiophrya varians* BZ 12 EF | MN121076 | MZ048814 |
| *Miamiensis avidus* | JN885091 | MH577597 |
| *Myxophyllum steenstrupi* KR 11CL | MT649640 | MT649652 |
| *Paramecium primaurelia* | AF100315 | K01750 |
| *Paramecium tetraurelia* | X03772 | K01751 |
| *Parauronema longum* | HM236338 | MH577578 |
| *Philasterides armatalis* | FJ848877 | MH577575 |
| *Platyophrya bromelicola* | EU039906 | HM246415 |
| *Pleuronema coronatum* pop.1 | JX310014 | MH577574 |
| *Pleuronema coronatum* pop.2 | AY103188 | MH577594 |
| *Pleuronema grolierei* | KF840519 | MH577584 |
| *Protocyclidium citrullus* | KF256820 | MH577580 |
| *Pseudocohnilembus persalinus* | GQ265955 | MH577587 |
| *Subanoplophrya nodulata* PU 29 OT | MN121064 | MZ048822 |
| *Tetrahymena acanthophora* | MN994469 | MN994474 |
| *Tetrahymena dugesiae* | MK454732 | MN994480 |
| *Tetrahymena foissneri* ST 1 AV | MW827176 | MW827185 |
| *Tetrahymena malaccensis* | M26360 | DQ927303 |
| *Tetrahymena nigricans* | MN994472 | MN994483 |
| *Tetrahymena paravorax* | EF070253 | NC008338 |
| *Tetrahymena pigmentosa* | M26358 | NC008339 |
| *Tetrahymena pyriformis* | EF070254 | AF160864 |

| **Taxon** | **18S rRNA gene** | **16S rRNA gene** |
|---|---|---|
| *Tetrahymena scolopax* | KJ028504 | MN994484 |
| *Tetrahymena thermophila* | MH051926 | NC003029 |
| *Tetrahymena unionis* HO 35 UT | MW827181 | MW827190 |
| *Trichodina polycelis* HR 95 PF | MW759641 | MW768981 |
| *Trichodina schmidtea* JJ 108 SP | MW759648 | MW768988 |
| *Trichodina steinii* BB 121 DG | MW759654 | MW768994 |
| *Trichodina steinii* ST 133 DG | MW759658 | MW768998 |
| *Urceolaria mitra* KU 45 DG | MW759668 | MW769007 |
| *Urceolaria mitra* BB 76 DG | MW759660 | MW769000 |
| *Urceolaria mitra* BY 138 DG | MW759665 | MW769003 |
| *Uronema heteromarinum* | FJ870100 | MH577576 |
| *Uronema marinum* | GQ465466 | MH577577 |
| *Uronemita filificum* | MH574793 | MH577602 |
| *Uronemita parabinucleata* | KU199245 | MH577590 |

**SUPPLEMENTARY TABLE S5 |** Characterization and origin of the mitochondrial COI gene sequences of host planarians analyzed in the present study.

| Species | Specimen | Locality no. | Locality | GenBank no. |
|---|---|---|---|---|
| *Dendroceolum lacteum* | VA 64 | 19 | Vajnorské jazero lake, Vajnory, Bratislava, Podunajská rovina plain | OL716069 |
| *Dugesia gonocephala* | BB 86<br>BB 88 | 20 | Unnamed stream, residential area Fončorda, Banská Bystrica, Zvolenská kotlina basin | OL716070<br>OL716071 |
| | HR 90<br>HR 120 | 23 | Hrabovský potok stream, Hrabovská dolina valley, Veľká Fatra Mts. | OL716072<br>OL716073 |
| | ST 134<br>ST 135 | 21 | Shallow section of the Váh river near the village of Stankovany, Veľká Fatra Mts. | OL716074<br>OL716075 |
| | BY 140<br>BY 144 | 22 | Shallow section of the Váh river near the village of Bystrá, Veľká Fatra Mts. | OL716076<br>OL716077 |
| *Girardia tigrina* | ZP 80<br>ZP 81 | 16 | Zlaté Piesky lake, municipal recreation area, Bratislava, Podunajská rovina plain | OL716078<br>OL716079 |
| | VY 159<br>VY 160 | 26 | Artificial pond, Východné, Bratislava, Podunajská rovina plain | OL716080<br>OL716081 |
| *Polycelis felina* | HR 89<br>HR 91 | 23 | Hrabovský potok stream, Hrabovská dolina valley, Veľká Fatra Mts. | OL716082<br>OL716083 |
| | DR 164<br>DR 165 | 5 | Drieňovecká mokraď swamp, Slovenský kras (Slovak Karst) National Park, Slovenské Rudohorie (Slovak Ore Mts.) | OL716084<br>OL716085 |

*For locality codes and further details, see Supplementary Table S1.*

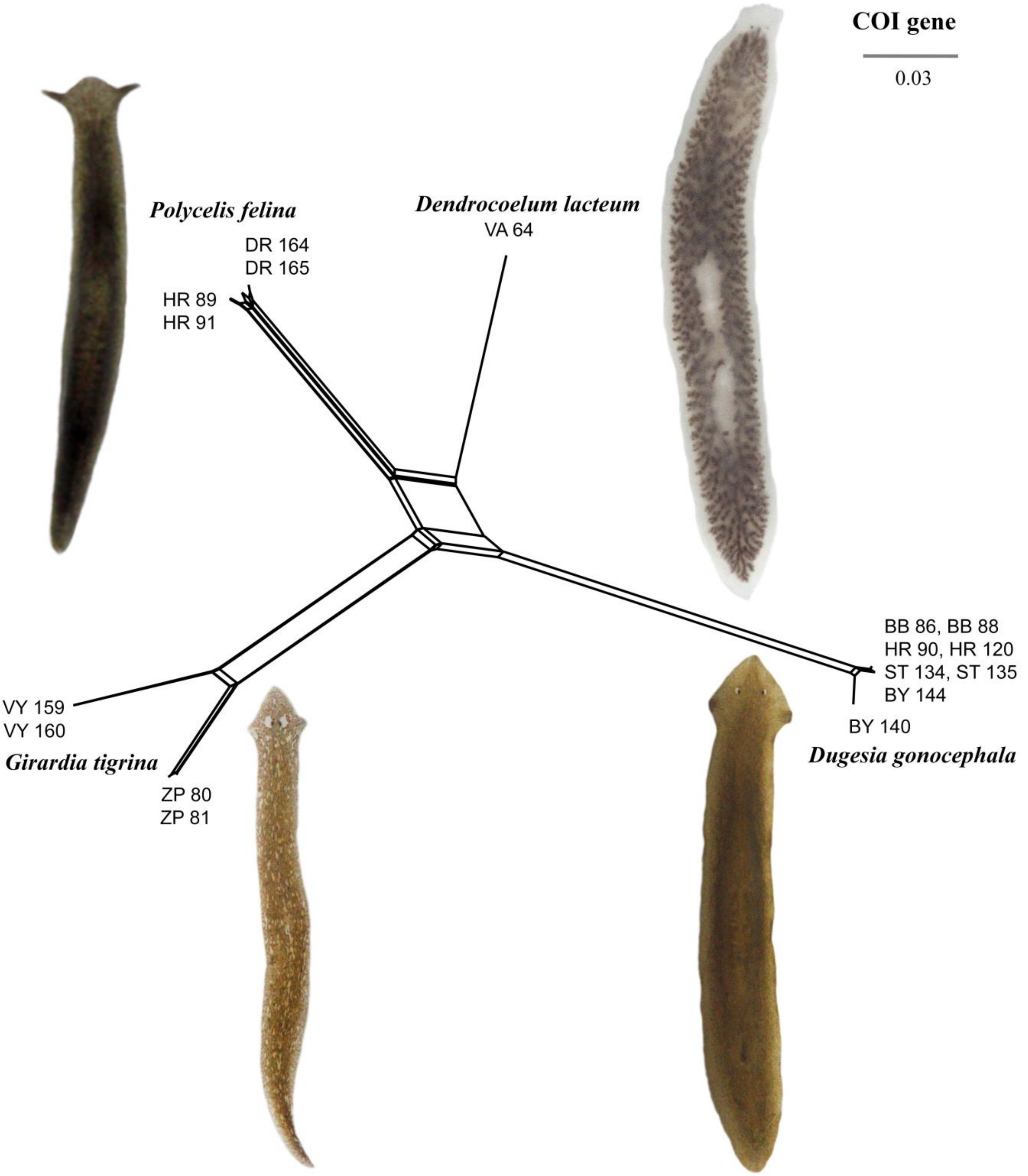


**SUPPLEMENTARY FIGURE S1 |** Phylogenetic network computed from cytochrome oxidase *c* subunit I (COI) sequences of some studied planarians, using the neighbornet algorithm and the uncorrected distances in SplitsTree ver. 4. For GenBank accession numbers of newly obtained sequences, see Supplementary Table S5. The scale bar indicates three substitutions per one hundred nucleotide positions.

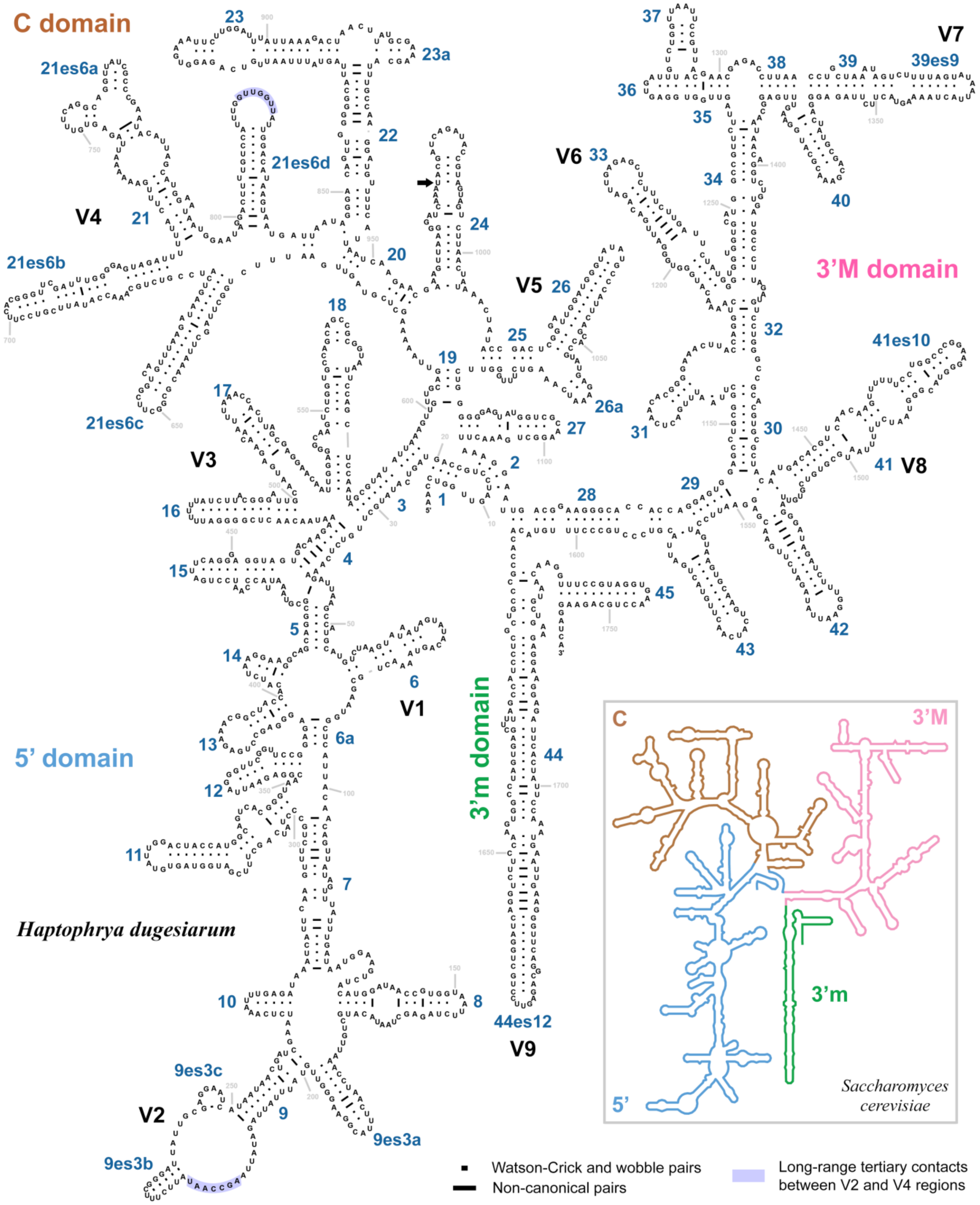


**SUPPLEMENTARY FIGURE S2 |** Secondary structure of the 18S rRNA molecule of *Haptophrya dugesiarum* nov. spec., based on models taking into account 3D structures. 18S secondary structure map of *Saccharomyces cerevisiae* (inset) is from http://apollo.chemistry.gatech.edu/RibosomeGallery (Petrov et al., 2014). Arrow marks the ancestrally polymorphic position 975 in helix 24, where is either uracil or guanine. Both nucleotide states retain the RNA helical structure.

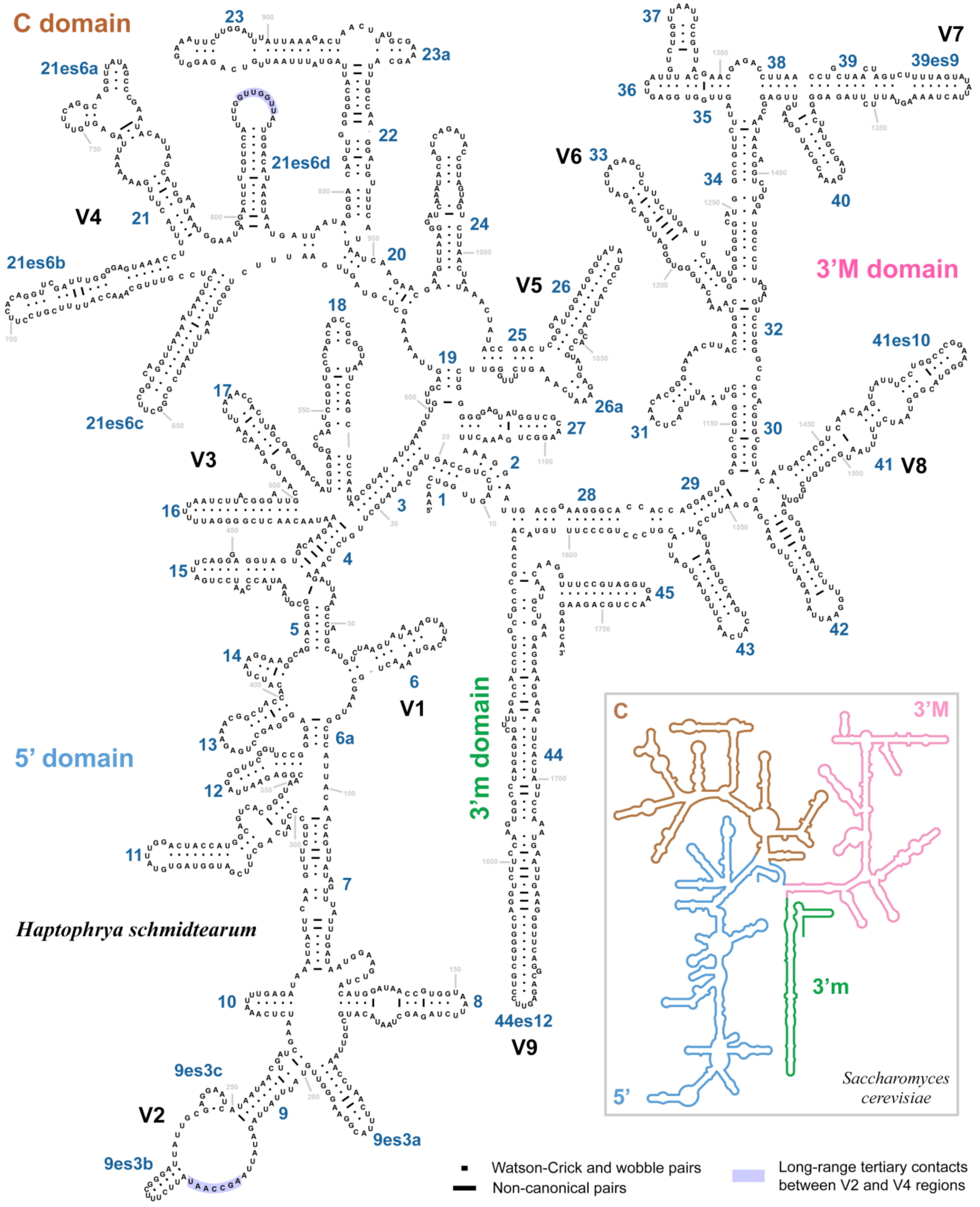


**SUPPLEMENTARY FIGURE S3 |** Secondary structure of the 18S rRNA molecule of *Haptophrya schmidtearum* nov. spec., based on models taking into account 3D structures. 18S secondary structure map of *Saccharomyces cerevisiae* (inset) is from http://apollo.chemistry.gatech.edu/RibosomeGallery (Petrov et al., 2014).

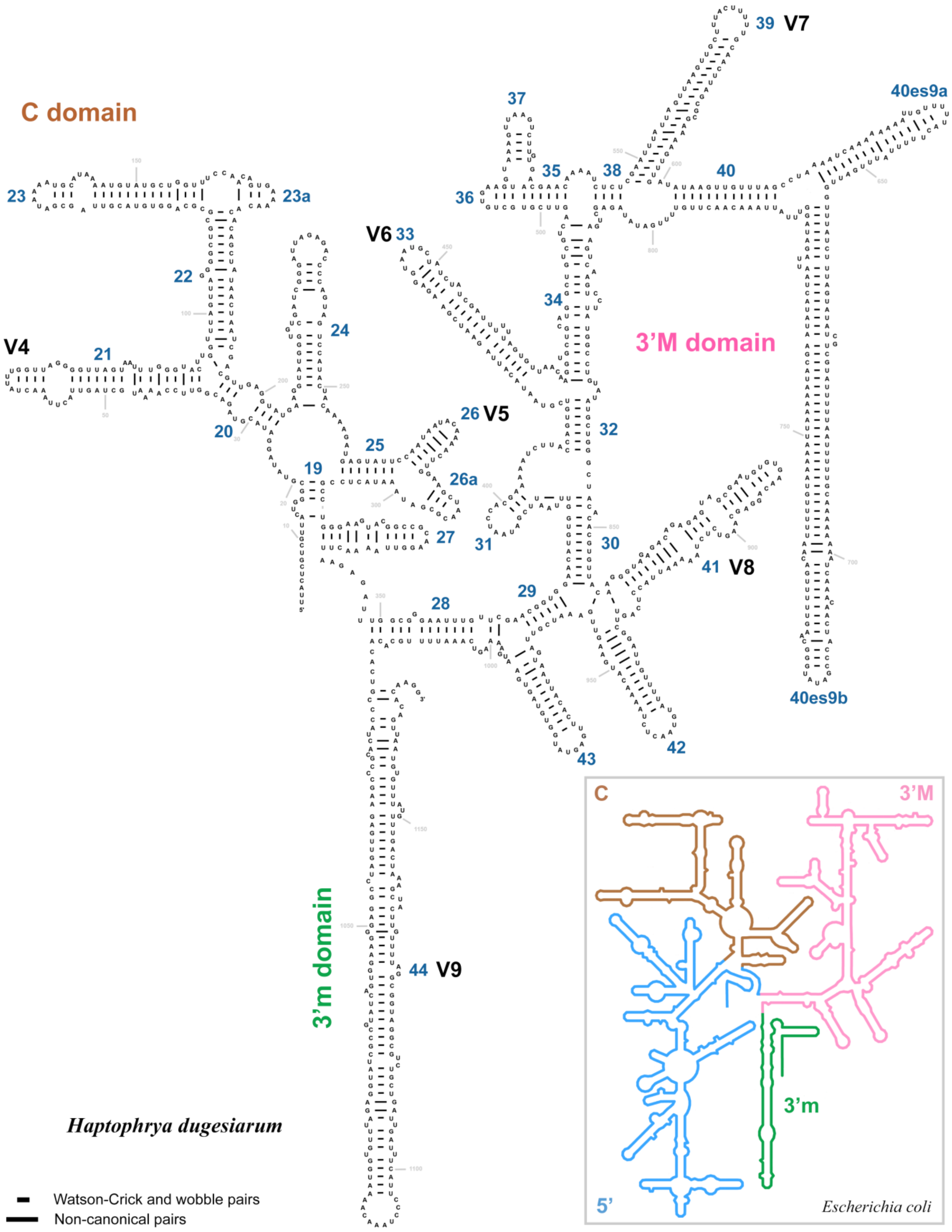


**SUPPLEMENTARY FIGURE S4 |** Secondary structure of the 16S rRNA molecule of *H. dugesiarum* nov. spec., based on models taking into account 3D structures. 16S secondary structure map of *Escherichia coli* (inset) is from http://apollo.chemistry.gatech.edu/RibosomeGallery (Petrov et al., 2014).

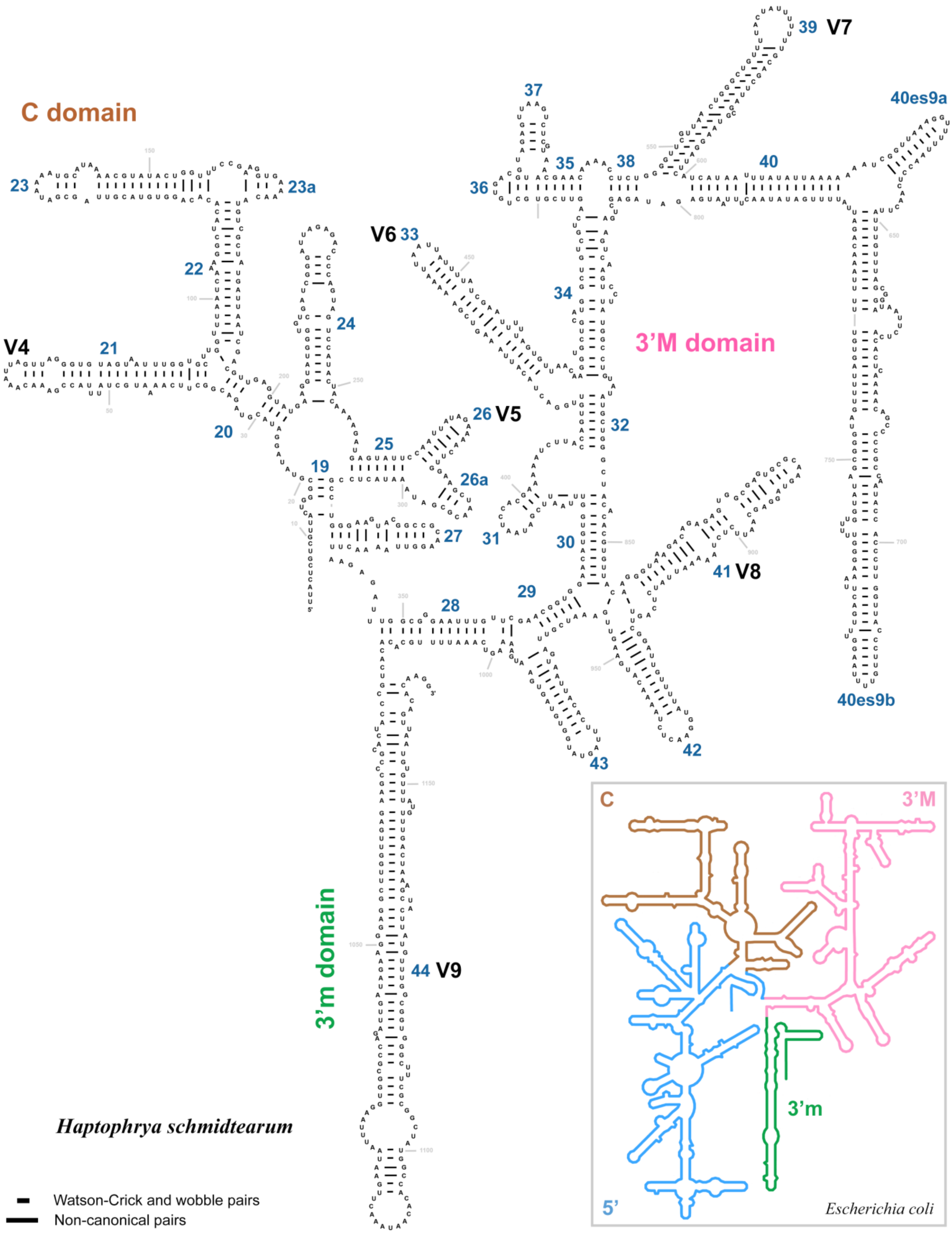


**SUPPLEMENTARY FIGURE S5 |** Secondary structure of the 16S rRNA molecule of *H. schmidtearum* nov. spec., based on models taking into account 3D structures. 16S secondary structure map of *Escherichia coli* (inset) is from http://apollo.chemistry.gatech.edu/RibosomeGallery (Petrov et al., 2014).

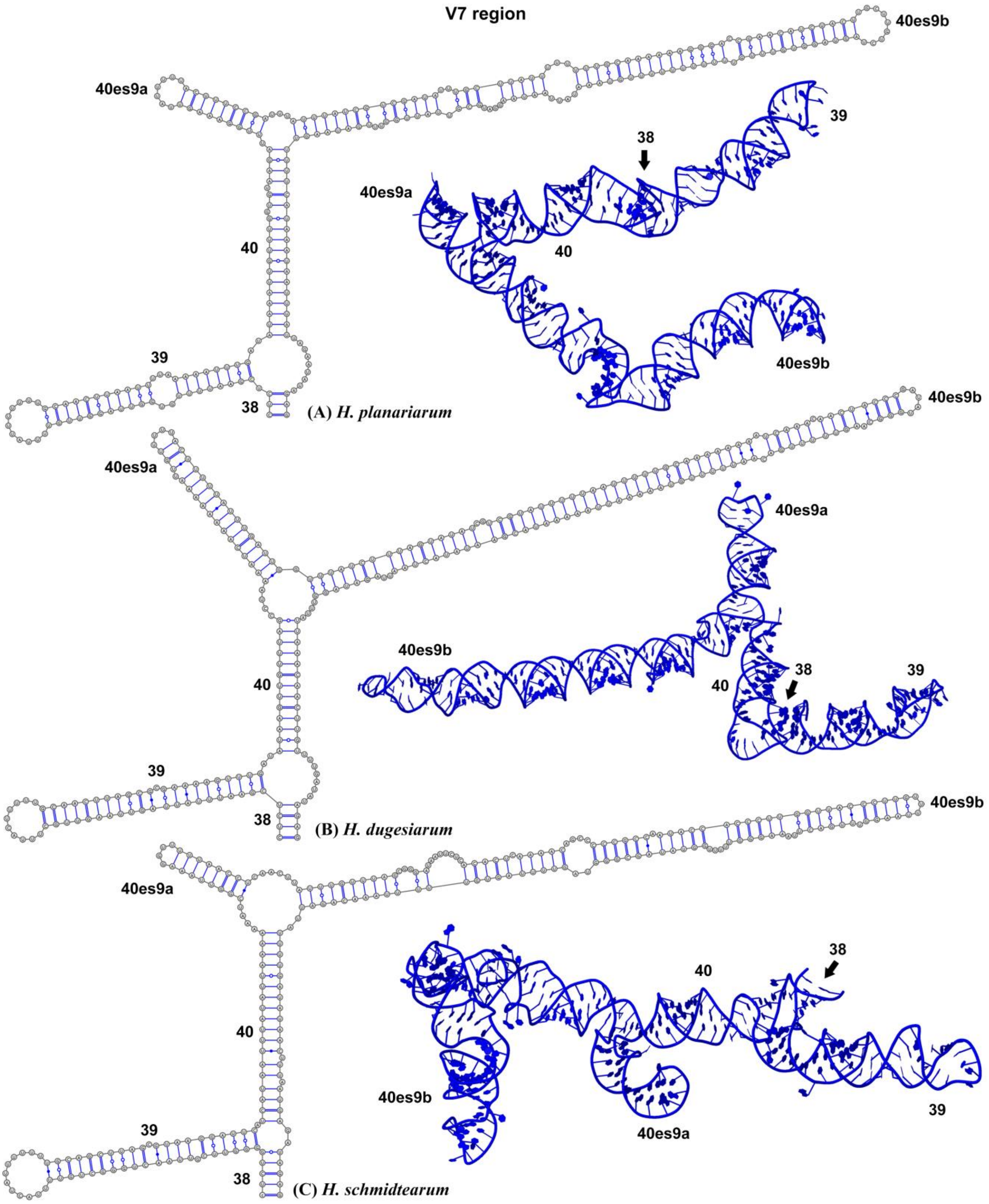


**SUPPLEMENTARY FIGURE S6 |** Secondary structure of the highly divergent V7 region of the 16S rRNA molecule of *H. planariarum* **(A)**, *H. dugesiarum* nov. spec. **(B)**, and *H. schmidtearum* nov. spec. **(C)**.

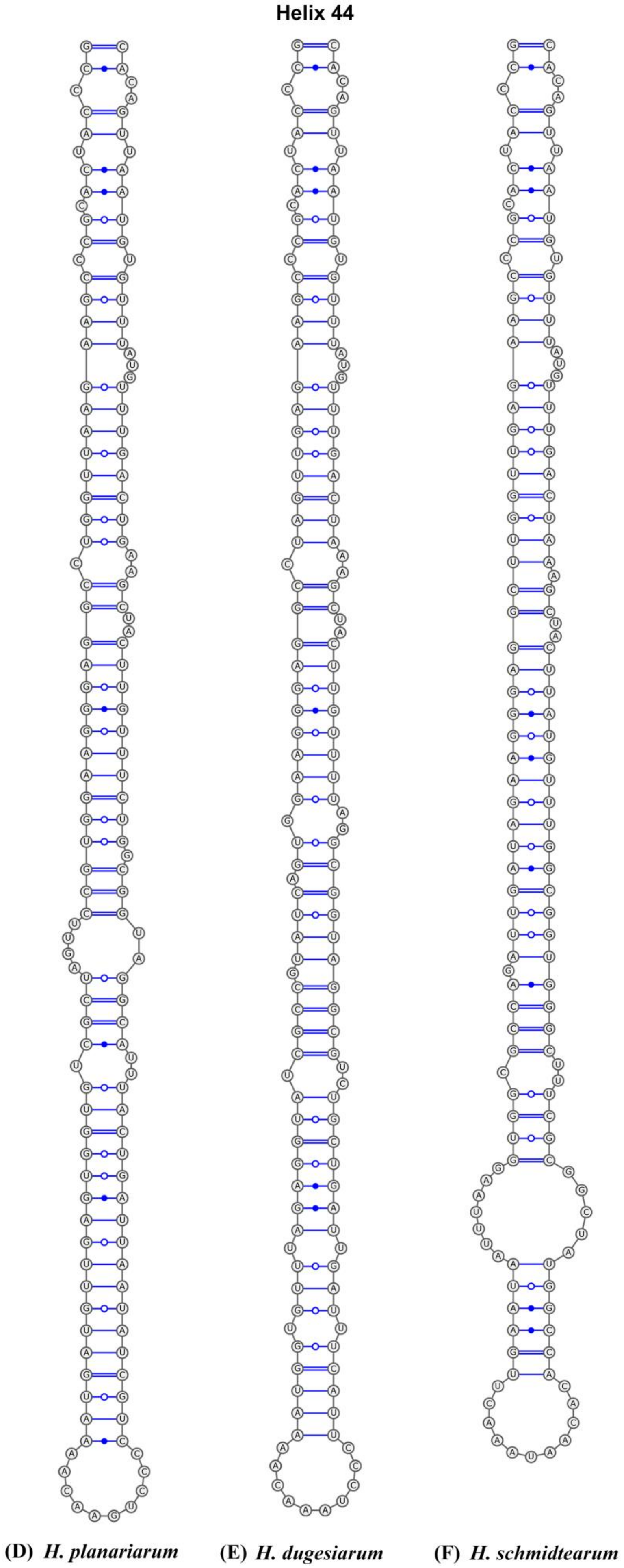


**SUPPLEMENTARY FIGURE S7 |** Secondary structure of the highly divergent V9 region of the 16S rRNA molecule of *H. planariarum* **(A)**, *H. dugesiarum* nov. spec. **(B)**, and *H. schmidtearum* nov. spec. **(C)**.

## 18S rRNA gene alignment

>Haptophrya_planariarum_KD_1_DG
AACCTGGTTGATCCTGCCAGTAGTCATATGCTTGTCTCAAAGATTAAGCCATGCATGTCTAAGTATAATAGTATACAGTAAAACTGCGA
ATGGCTCATTACAACAGTTATAGTTTATTTGATAATGGAAGTCTACATGGATAACCGTGGTAATTCTAGAGCTAATACATGCTGTTAAA
CCTAACTTTACGGAAGGGTTGTATTTATTAGATATTAAGCCAATATTCTTTCGGGATTATTGCGAGGAATCATAATAACTGATCGAATC
TCAAATTTGAGATAAATCATTCAAGTTTCTGCCCTATCAGCTTTCGATGGTAGTGTATTGGACTACCATGGCAGTCACGGGTAACGGAG
AATTAGGGTTCGGTTCCGGAGAGGGAGCCTGAGAAACGGCTACCACATCTAAGGAAGGCAGCAGGCGCGTAAATTACCCAATCCTGATT
CAGGGAGGTAGTGACAAGAAATAACAACTCGGGGATTTTTAATCTTACGGGATTGCAATGAGAACAATTTAAACCACTTAGCGAGGAAC
AATTGGAGGGCAAGTCTGGTGCCAGCAGCCGCGGTAATTCCAGCTCCAATAGCGTATATTAAAGTTGTTGCAGTTAAAAAGCTCGTAGT
TGAATTTCTGGCTTAGCTTAAACGCGGCTTCGGTCAGTTTAAGATAAGTCATCCGTCTGCAAACCATATTCGTCCTTCACGGGTTCGAT
TTGGGGAGTAGATTTTTTACTTTGAAAAAATTAGAGTGTTTCAGGCAGGTTATCGCCCGAATACATTAGCATGGAATAATGGAATAGGA
CTTTTGTCCATTTGGTTGGTTATTGGACATAAGTAATGATTAATAGGGACAGTTGGGGGCATTAGTATTTAATTGTCAGAGGTGAAATT
CTTGGATTTATTAAAGACTAACTTATGCGAAAGCATTTGCCAAGGATGTTTTCATTAATCAAGAACGAAAGTTAAGGGATCAAATACGA
TCAGATACCGTAGTAGTCTTAACTATAAACTATACCGACTCGGGATTGGATGGGTATATGCCCATTCAGCACCGTATGAGAAATCAAAG
TCTTTGGGTTCTGGGGGGAGTATGGTCGCAAGGCTGAAACTTAAAGGAATTGACGGAAGGGCACCACCAGGAGTGGAGCCTGCGGCTTA
ATTTGACTCAACACGGGGAAACTTACCAGGTCAAAACATGGGTGGGATTGACAGATTGAGAGCTCTTTCTTGATTCTATGGGTGGTGGT
GCATGGCCGTTCTTAGTTGGTGGAGTGATTTGTCTGGTTAATTCCGTTAACGAACGAGACCTTAACCTGCTAACTAGTCTTTTAGTATA
ATTACTAAAAGTACTTCTTAGAGGGACTATGCGACGAAACGCATGGAAGTTTGAGGCAATAACAGGTCTGTGATGCCCTTAGATGTCCT
GGGCCGCACGTGCGCTACAATGACACGTTCAACAAGTTTTTTCCTGGCCCGGAAGGGTACGGGTAATCTTTTTAATGCGTGTCGTGTTA
GGGATAGATCTTTGGAATTATAGATCTTGAACGAGGAATTCCTAGTAAGTGCAAGTCATCAACTTGTACTGATTACGTCCCTGCCCTTT
GTACACACCGCCCGTCGCTCCTACCGATTTCGAGTGATCCGGTGAACCTTCTGGACTAGGTCGTCCTTGAGACGGACTTGGGAAGTTAA
GTAAACCTTATCACTTAGAGGAAGGAGAAGTCGTAACAAGGTTTCCGTAGGTGAACCTGCAGAAGGATCA
>Haptophrya_planariarum_KD_2_DG
AACCTGGTTGATCCTGCCAGTAGTCATATGCTTGTCTCAAAGATTAAGCCATGCATGTCTAAGTATAATAGTATACAGTAAAACTGCGA
ATGGCTCATTACAACAGTTATAGTTTATTTGATAATGGAAGTCTACATGGATAACCGTGGTAATTCTAGAGCTAATACATGCTGTTAAA
CCTAACTTTACGGAAGGGTTGTATTTATTAGATATTAAGCCAATATTCTTTCGGGATTATTGCGAGGAATCATAATAACTGATCGAATC
TCAAATTTGAGATAAATCATTCAAGTTTCTGCCCTATCAGCTTTCGATGGTAGTGTATTGGACTACCATGGCAGTCACGGGTAACGGAG
AATTAGGGTTCGGTTCCGGAGAGGGAGCCTGAGAAACGGCTACCACATCTAAGGAAGGCAGCAGGCGCGTAAATTACCCAATCCTGATT
CAGGGAGGTAGTGACAAGAAATAACAACTCGGGGATTTTTAATCTTACGGGATTGCAATGAGAACAATTTAAACCACTTAGCGAGGAAC
AATTGGAGGGCAAGTCTGGTGCCAGCAGCCGCGGTAATTCCAGCTCCAATAGCGTATATTAAAGTTGTTGCAGTTAAAAAGCTCGTAGT
TGAATTTCTGGCTTAGCTTAAACGCGGCTTCGGTCAGTTTAAGATAAGTCATCCGTCTGCAAACCATATTCGTCCTTCACGGGTTCGAT
TTGGGGAGTAGATTTTTTACTTTGAAAAAATTAGAGTGTTTCAGGCAGGTTATCGCCCGAATACATTAGCATGGAATAATGGAATAGGA
CTTTTGTCCATTTGGTTGGTTATTGGACATAAGTAATGATTAATAGGGACAGTTGGGGGCATTAGTATTTAATTGTCAGAGGTGAAATT
CTTGGATTTATTAAAGACTAACTTATGCGAAAGCATTTGCCAAGGATGTTTTCATTAATCAAGAACGAAAGTTAAGGGATCAAATACGA
TCAGATACCGTAGTAGTCTTAACTATAAACTATACCGACTCGGGATTGGATGGGTATATGCCCATTCAGCACCGTATGAGAAATCAAAG
TCTTTGGGTTCTGGGGGGAGTATGGTCGCAAGGCTGAAACTTAAAGGAATTGACGGAAGGGCACCACCAGGAGTGGAGCCTGCGGCTTA
ATTTGACTCAACACGGGGAAACTTACCAGGTCAAAACATGGGTGGGATTGACAGATTGAGAGCTCTTTCTTGATTCTATGGGTGGTGGT
GCATGGCCGTTCTTAGTTGGTGGAGTGATTTGTCTGGTTAATTCCGTTAACGAACGAGACCTTAACCTGCTAACTAGTCTTTTAGTATA
ATTACTAAAAGTACTTCTTAGAGGGACTATGCGACGAAACGCATGGAAGTTTGAGGCAATAACAGGTCTGTGATGCCCTTAGATGTCCT
GGGCCGCACGTGCGCTACAATGACACGTTCAACAAGTTTTTTCCTGGCCCGGAAGGGTACGGGTAATCTTTTTAATGCGTGTCGTGTTA
GGGATAGATCTTTGGAATTATAGATCTTGAACGAGGAATTCCTAGTAAGTGCAAGTCATCAACTTGTACTGATTACGTCCCTGCCCTTT
GTACACACCGCCCGTCGCTCCTACCGATTTCGAGTGATCCGGTGAACCTTCTGGACTAGGTCGTCCTTGAGACGGACTTGGGAAGTTAA
GTAAACCTTATCACTTAGAGGAAGGAGAAGTCGTAACAAGGTTTCCGTAGGTGAACCTGCAGAAGGATCA
>Haptophrya_planariarum_KD_3_DG
AACCTGGTTGATCCTGCCAGTAGTCATATGCTTGTCTCAAAGATTAAGCCATGCATGTCTAAGTATAATAGTATACAGTAAAACTGCGA
ATGGCTCATTACAACAGTTATAGTTTATTTGATAATGGAAGTCTACATGGATAACCGTGGTAATTCTAGAGCTAATACATGCTGTTAAA
CCTAACTTTACGGAAGGGTTGTATTTATTAGATATTAAGCCAATATTCTTTCGGGATTATTGCGAGGAATCATAATAACTGATCGAATC
TCAAATTTGAGATAAATCATTCAAGTTTCTGCCCTATCAGCTTTCGATGGTAGTGTATTGGACTACCATGGCAGTCACGGGTAACGGAG
AATTAGGGTTCGGTTCCGGAGAGGGAGCCTGAGAAACGGCTACCACATCTAAGGAAGGCAGCAGGCGCGTAAATTACCCAATCCTGATT
CAGGGAGGTAGTGACAAGAAATAACAACTCGGGGATTTTTAATCTTACGGGATTGCAATGAGAACAATTTAAACCACTTAGCGAGGAAC
AATTGGAGGGCAAGTCTGGTGCCAGCAGCCGCGGTAATTCCAGCTCCAATAGCGTATATTAAAGTTGTTGCAGTTAAAAAGCTCGTAGT
TGAATTTCTGGCTTAGCTTAAACGCGGCTTCGGTCAGTTTAAGATAAGTCATCCGTCTGCAAACCATATTCGTCCTTCACGGGTTCGAT
TTGGGGAGTAGATTTTTTACTTTGAAAAAATTAGAGTGTTTCAGGCAGGTTATCGCCCGAATACATTAGCATGGAATAATGGAATAGGA
CTTTTGTCCATTTGGTTGGTTATTGGACATAAGTAATGATTAATAGGGACAGTTGGGGGCATTAGTATTTAATTGTCAGAGGTGAAATT
CTTGGATTTATTAAAGACTAACTTATGCGAAAGCATTTGCCAAGGATGTTTTCATTAATCAAGAACGAAAGTTAAGGGATCAAATACGA
TCAGATACCGTAGTAGTCTTAACTATAAACTATACCGACTCGGGATTGGATGGGTATATGCCCATTCAGCACCGTATGAGAAATCAAAG
TCTTTGGGTTCTGGGGGGAGTATGGTCGCAAGGCTGAAACTTAAAGGAATTGACGGAAGGGCACCACCAGGAGTGGAGCCTGCGGCTTA
ATTTGACTCAACACGGGGAAACTTACCAGGTCAAAACATGGGTGGGATTGACAGATTGAGAGCTCTTTCTTGATTCTATGGGTGGTGGT
GCATGGCCGTTCTTAGTTGGTGGAGTGATTTGTCTGGTTAATTCCGTTAACGAACGAGACCTTAACCTGCTAACTAGTCTTTTAGTATA
ATTACTAAAAGTACTTCTTAGAGGGACTATGCGACGAAACGCATGGAAGTTTGAGGCAATAACAGGTCTGTGATGCCCTTAGATGTCCT
GGGCCGCACGTGCGCTACAATGACACGTTCAACAAGTTTTTTCCTGGCCCGGAAGGGTACGGGTAATCTTTTTAATGCGTGTCGTGTTA
GGGATAGATCTTTGGAATTATAGATCTTGAACGAGGAATTCCTAGTAAGTGCAAGTCATCAACTTGTACTGATTACGTCCCTGCCCTTT
GTACACACCGCCCGTCGCTCCTACCGATTTCGAGTGATCCGGTGAACCTTCTGGACTAGGTCGTCCTTGAGACGGACTTGGGAAGTTAA
GTAAACCTTATCACTTAGAGGAAGGAGAAGTCGTAACAAGGTTTCCGTAGGTGAACCTGCAGAAGGATCA
>Haptophrya_planariarum_OL_4_DG
AACCTGGTTGATCCTGCCAGTAGTCATATGCTTGTCTCAAAGATTAAGCCATGCATGTCTAAGTATAATAGTATACAGTAAAACTGCGA
ATGGCTCATTACAACAGTTATAGTTTATTTGATAATGGAAGTCTACATGGATAACCGTGGTAATTCTAGAGCTAATACATGCTGTTAAA
CCTAACTTTACGGAAGGGTTGTATTTATTAGATATTAAGCCAATATTCTTTCGGGATTATTGCGAGGAATCATAATAACTGATCGAATC
TCAAATTTGAGATAAATCATTCAAGTTTCTGCCCTATCAGCTTTCGATGGTAGTGTATTGGACTACCATGGCAGTCACGGGTAACGGAG

AATTAGGGTTCGGTTCCGGAGAGGGAGCCTGAGAAACGGCTACCACATCTAAGGAAGGCAGCAGGCGCGTAAATTACCCAATCCTGATT
CAGGGAGGTAGTGACAAGAAATAACAACTCGGGGATTTTTAATCTTACGGGATTGCAATGAGAACAATTTAAACCACTTAGCGAGGAAC
AATTGGAGGGCAAGTCTGGTGCCAGCAGCCGCGGTAATTCCAGCTCCAATAGCGTATATTAAAGTTGTTGCAGTTAAAAAGCTCGTAGT
TGAATTTCTGGCTTAGCTTAAACGCGGCTTCGGTCAGTTTAAGATAAGTCATCCGTCTGCAAACCATATTCGTCCTTCACGGGTTCGAT
TTGGGGAGTAGATTTTTTACTTTGAAAAAATTAGAGTGTTTCAGGCAGGTTATCGCCCGAATACATTAGCATGGAATAATGGAATAGGA
CTTTTGTCCATTTGGTTGGTTATTGGACATAAGTAATGATTAATAGGGACAGTTGGGGGCATTAGTATTTAATTGTCAGAGGTGAAATT
CTTGGATTTATTAAAGACTAACTTATGCGAAAGCATTTGCCAAGGATGTTTTCATTAATCAAGAACGAAAGTTAAGGGATCAAATACGA
TCAGATACCGTAGTAGTCTTAACTATAAACTATACCGACTCGGGATTGGATGGGTATATGCCCATTCAGCACCGTATGAGAAATCAAAG
TCTTTGGGTTCTGGGGGGAGTATGGTCGCAAGGCTGAAACTTAAAGGAATTGACGGAAGGGCACCACCAGGAGTGGAGCCTGCGGCTTA
ATTTGACTCAACACGGGGAAACTTACCAGGTCAAAACATGGGTGGGATTGACAGATTGAGAGCTCTTTCTTGATTCTATGGGTGGTGGT
GCATGGCCGTTCTTAGTTGGTGGAGTGATTTGTCTGGTTAATTCCGTTAACGAACGAGACCTTAACCTGCTAACTAGTCTTTTAGTATA
ATTACTAAAAGTACTTCTTAGAGGGACTATGCGACGAAACGCATGGAAGTTTGAGGCAATAACAGGTCTGTGATGCCCTTAGATGTCCT
GGGCCGCACGTGCGCTACAATGACACGTTCAACAAGTTTTTTCCTGGCCCGGAAGGGTACGGGTAATCTTTTTAATGCGTGTCGTGTTA
GGGATAGATCTTTGGAATTATAGATCTTGAACGAGGAATTCCTAGTAAGTGCAAGTCATCAACTTGTACTGATTACGTCCCTGCCCTTT
GTACACACCGCCCGTCGCTCCTACCGATTTCGAGTGATCCGGTGAACCTTCTGGACTAGGTCGTCCTTGAGACGGACTTGGGAAGTTAA
GTAAACCTTATCACTTAGAGGAAGGAGAAGTCGTAACAAGGTTTCCGTAGGTGAACCTGCAGAAGGATCA
>Haptophrya_planariarum_OL_6_DG
AACCTGGTTGATCCTGCCAGTAGTCATATGCTTGTCTCAAAGATTAAGCCATGCATGTCTAAGTATAATAGTATACAGTAAAACTGCGA
ATGGCTCATTACAACAGTTATAGTTTATTTGATAATGGAAGTCTACATGGATAACCGTGGTAATTCTAGAGCTAATACATGCTGTTAAA
CCTAACTTTACGGAAGGGTTGTATTTATTAGATATTAAGCCAATATTCTTTCGGGATTATTGCGAGGAATCATAATAACTGATCGAATC
TCAAATTTGAGATAAATCATTCAAGTTTCTGCCCTATCAGCTTTCGATGGTAGTGTATTGGACTACCATGGCAGTCACGGGTAACGGAG
AATTAGGGTTCGGTTCCGGAGAGGGAGCCTGAGAAACGGCTACCACATCTAAGGAAGGCAGCAGGCGCGTAAATTACCCAATCCTGATT
CAGGGAGGTAGTGACAAGAAATAACAACTCGGGGATTTTTAATCTTACGGGATTGCAATGAGAACAATTTAAACCACTTAGCGAGGAAC
AATTGGAGGGCAAGTCTGGTGCCAGCAGCCGCGGTAATTCCAGCTCCAATAGCGTATATTAAAGTTGTTGCAGTTAAAAAGCTCGTAGT
TGAATTTCTGGCTTAGCTTAAACGCGGCTTCGGTCAGTTTAAGATAAGTCATCCGTCTGCAAACCATATTCGTCCTTCACGGGTTCGAT
TTGGGGAGTAGATTTTTTACTTTGAAAAAATTAGAGTGTTTCAGGCAGGTTATCGCCCGAATACATTAGCATGGAATAATGGAATAGGA
CTTTTGTCCATTTGGTTGGTTATTGGACATAAGTAATGATTAATAGGGACAGTTGGGGGCATTAGTATTTAATTGTCAGAGGTGAAATT
CTTGGATTTATTAAAGACTAACTTATGCGAAAGCATTTGCCAAGGATGTTTTCATTAATCAAGAACGAAAGTTAAGGGATCAAAGACGA
TCAGATACCGTAGTAGTCTTAACTATAAACTATACCGACTCGGGATTGGATGGGTATATGCCCATTCAGCACCGTATGAGAAATCAAAG
TCTTTGGGTTCTGGGGGGAGTATGGTCGCAAGGCTGAAACTTAAAGGAATTGACGGAAGGGCACCACCAGGAGTGGAGCCTGCGGCTTA
ATTTGACTCAACACGGGGAAACTTACCAGGTCAAAACATGGGTGGGATTGACAGATTGAGAGCTCTTTCTTGATTCTATGGGTGGTGGT
GCATGGCCGTTCTTAGTTGGTGGAGTGATTTGTCTGGTTAATTCCGTTAACGAACGAGACCTTAACCTGCTAACTAGTCTTTTAGTATA
ATTACTAAAAGTACTTCTTAGAGGGACTATGCGACGAAACGCATGGAAGTTTGAGGCAATAACAGGTCTGTGATGCCCTTAGATGTCCT
GGGCCGCACGTGCGCTACAATGACACGTTCAACAAGTTTTTTCCTGGCCCGGAAGGGTACGGGTAATCTTTTTAATGCGTGTCGTGTTA
GGGATAGATCTTTGGAATTATAGATCTTGAACGAGGAATTCCTAGTAAGTGCAAGTCATCAACTTGTACTGATTACGTCCCTGCCCTTT
GTACACACCGCCCGTCGCTCCTACCGATTTCGAGTGATCCGGTGAACCTTCTGGACTAGGTCGTCCTTGAGACGGACTTGGGAAGTTAA
GTAAACCTTATCACTTAGAGGAAGGAGAAGTCGTAACAAGGTTTCCGTAGGTGAACCTGCAGAAGGATCA
>Haptophrya_planariarum_OL_7_DG
AACCTGGTTGATCCTGCCAGTAGTCATATGCTTGTCTCAAAGATTAAGCCATGCATGTCTAAGTATAATAGTATACAGTAAAACTGCGA
ATGGCTCATTACAACAGTTATAGTTTATTTGATAATGGAAGTCTACATGGATAACCGTGGTAATTCTAGAGCTAATACATGCTGTTAAA
CCTAACTTTACGGAAGGGTTGTATTTATTAGATATTAAGCCAATATTCTTTCGGGATTATTGCGAGGAATCATAATAACTGATCGAATC
TCAAATTTGAGATAAATCATTCAAGTTTCTGCCCTATCAGCTTTCGATGGTAGTGTATTGGACTACCATGGCAGTCACGGGTAACGGAG
AATTAGGGTTCGGTTCCGGAGAGGGAGCCTGAGAAACGGCTACCACATCTAAGGAAGGCAGCAGGCGCGTAAATTACCCAATCCTGATT
CAGGGAGGTAGTGACAAGAAATAACAACTCGGGGATTTTTAATCTTACGGGATTGCAATGAGAACAATTTAAACCACTTAGCGAGGAAC
AATTGGAGGGCAAGTCTGGTGCCAGCAGCCGCGGTAATTCCAGCTCCAATAGCGTATATTAAAGTTGTTGCAGTTAAAAAGCTCGTAGT
TGAATTTCTGGCTTAGCTTAAACGCGGCTTCGGTCAGTTTAAGATAAGTCATCCGTCTGCAAACCATATTCGTCCTTCACGGGTTCGAT
TTGGGGAGTAGATTTTTTACTTTGAAAAAATTAGAGTGTTTCAGGCAGGTTATCGCCCGAATACATTAGCATGGAATAATGGAATAGGA
CTTTTGTCCATTTGGTTGGTTATTGGACATAAGTAATGATTAATAGGGACAGTTGGGGGCATTAGTATTTAATTGTCAGAGGTGAAATT
CTTGGATTTATTAAAGACTAACTTATGCGAAAGCATTTGCCAAGGATGTTTTCATTAATCAAGAACGAAAGTTAAGGGATCAAATACGA
TCAGATACCGTAGTAGTCTTAACTATAAACTATACCGACTCGGGATTGGATGGGTATATGCCCATTCAGCACCGTATGAGAAATCAAAG
TCTTTGGGTTCTGGGGGGAGTATGGTCGCAAGGCTGAAACTTAAAGGAATTGACGGAAGGGCACCACCAGGAGTGGAGCCTGCGGCTTA
ATTTGACTCAACACGGGGAAACTTACCAGGTCAAAACATGGGTGGGATTGACAGATTGAGAGCTCTTTCTTGATTCTATGGGTGGTGGT
GCATGGCCGTTCTTAGTTGGTGGAGTGATTTGTCTGGTTAATTCCGTTAACGAACGAGACCTTAACCTGCTAACTAGTCTTTTAGTATA
ATTACTAAAAGTACTTCTTAGAGGGACTATGCGACGAAACGCATGGAAGTTTGAGGCAATAACAGGTCTGTGATGCCCTTAGATGTCCT
GGGCCGCACGTGCGCTACAATGACACGTTCAACAAGTTTTTTCCTGGCCCGGAAGGGTACGGGTAATCTTTTTAATGCGTGTCGTGTTA
GGGATAGATCTTTGGAATTATAGATCTTGAACGAGGAATTCCTAGTAAGTGCAAGTCATCAACTTGTACTGATTACGTCCCTGCCCTTT
GTACACACCGCCCGTCGCTCCTACCGATTTCGAGTGATCCGGTGAACCTTCTGGACTAGGTCGTCCTTGAGACGGACTTGGGAAGTTAA
GTAAACCTTATCACTTAGAGGAAGGAGAAGTCGTAACAAGGTTTCCGTAGGTGAACCTGCAGAAGGATCA
>Haptophrya_planariarum_OL_8_DG
AACCTGGTTGATCCTGCCAGTAGTCATATGCTTGTCTCAAAGATTAAGCCATGCATGTCTAAGTATAATAGTATACAGTAAAACTGCGA
ATGGCTCATTACAACAGTTATAGTTTATTTGATAATGGAAGTCTACATGGATAACCGTGGTAATTCTAGAGCTAATACATGCTGTTAAA
CCTAACTTTACGGAAGGGTTGTATTTATTAGATATTAAGCCAATATTCTTTCGGGATTATTGCGAGGAATCATAATAACTGATCGAATC
TCAAATTTGAGATAAATCATTCAAGTTTCTGCCCTATCAGCTTTCGATGGTAGTGTATTGGACTACCATGGCAGTCACGGGTAACGGAG
AATTAGGGTTCGGTTCCGGAGAGGGAGCCTGAGAAACGGCTACCACATCTAAGGAAGGCAGCAGGCGCGTAAATTACCCAATCCTGATT
CAGGGAGGTAGTGACAAGAAATAACAACTCGGGGATTTTTAATCTTACGGGATTGCAATGAGAACAATTTAAACCACTTAGCGAGGAAC
AATTGGAGGGCAAGTCTGGTGCCAGCAGCCGCGGTAATTCCAGCTCCAATAGCGTATATTAAAGTTGTTGCAGTTAAAAAGCTCGTAGT
TGAATTTCTGGCTTAGCTTAAACGCGGCTTCGGTCAGTTTAAGATAAGTCATCCGTCTGCAAACCATATTCGTCCTTCACGGGTTCGAT
TTGGGGAGTAGATTTTTTACTTTGAAAAAATTAGAGTGTTTCAGGCAGGTTATCGCCCGAATACATTAGCATGGAATAATGGAATAGGA
CTTTTGTCCATTTGGTTGGTTATTGGACATAAGTAATGATTAATAGGGACAGTTGGGGGCATTAGTATTTAATTGTCAGAGGTGAAATT
CTTGGATTTATTAAAGACTAACTTATGCGAAAGCATTTGCCAAGGATGTTTTCATTAATCAAGAACGAAAGTTAAGGGATCAAAGACGA
TCAGATACCGTAGTAGTCTTAACTATAAACTATACCGACTCGGGATTGGATGGGTATATGCCCATTCAGCACCGTATGAGAAATCAAAG

TCTTTGGGTTCTGGGGGGAGTATGGTCGCAAGGCTGAAACTTAAAGGAATTGACGGAAGGGCACCACCAGGAGTGGAGCCTGCGGCTTA
ATTTGACTCAACACGGGGAAACTTACCAGGTCAAAACATGGGTGGGATTGACAGATTGAGAGCTCTTTCTTGATTCTATGGGTGGTGGT
GCATGGCCGTTCTTAGTTGGTGGAGTGATTTGTCTGGTTAATTCCGTTAACGAACGAGACCTTAACCTGCTAACTAGTCTTTTAGTATA
ATTACTAAAAGTACTTCTTAGAGGGACTATGCGACGAAACGCATGGAAGTTTGAGGCAATAACAGGTCTGTGATGCCCTTAGATGTCCT
GGGCCGCACGTGCGCTACAATGACACGTTCAACAAGTTTTTTCCTGGCCCGGAAGGGTACGGGTAATCTTTTTAATGCGTGTCGTGTTA
GGGATAGATCTTTGGAATTATAGATCTTGAACGAGGAATTCCTAGTAAGTGCAAGTCATCAACTTGTACTGATTACGTCCCTGCCCTTT
GTACACACCGCCCGTCGCTCCTACCGATTTCGAGTGATCCGGTGAACCTTCTGGACTAGGTCGTCCTTGAGACGGACTTGGGAAGTTAA
GTAAACCTTATCACTTAGAGGAAGGAGAAGTCGTAACAAGGTTTCCGTAGGTGAACCTGCAGAAGGATCA
>Haptophrya_planariarum_OL_9_DG
AACCTGGTTGATCCTGCCAGTAGTCATATGCTTGTCTCAAAGATTAAGCCATGCATGTCTAAGTATAATAGTATACAGTAAAACTGCGA
ATGGCTCATTACAACAGTTATAGTTTATTTGATAATGGAAGTCTACATGGATAACCGTGGTAATTCTAGAGCTAATACATGCTGTTAAA
CCTAACTTTACGGAAGGGTTGTATTTATTAGATATTAAGCCAATATTCTTTCGGGATTATTGCGAGGAATCATAATAACTGATCGAATC
TCAAATTTGAGATAAATCATTCAAGTTTCTGCCCTATCAGCTTTCGATGGTAGTGTATTGGACTACCATGGCAGTCACGGGTAACGGAG
AATTAGGGTTCGGTTCCGGAGAGGGAGCCTGAGAAACGGCTACCACATCTAAGGAAGGCAGCAGGCGCGTAAATTACCCAATCCTGATT
CAGGGAGGTAGTGACAAGAAATAACAACTCGGGGATTTTTAATCTTACGGGATTGCAATGAGAACAATTTAAACCACTTAGCGAGGAAC
AATTGGAGGGCAAGTCTGGTGCCAGCAGCCGCGGTAATTCCAGCTCCAATAGCGTATATTAAAGTTGTTGCAGTTAAAAAGCTCGTAGT
TGAATTTCTGGCTTAGCTTAAACGCGGCTTCGGTCAGTTTAAGATAAGTCATCCGTCTGCAAACCATATTCGTCCTTCACGGGTTCGAT
TTGGGGAGTAGATTTTTTACTTTGAAAAAATTAGAGTGTTTCAGGCAGGTTATCGCCCGAATACATTAGCATGGAATAATGGAATAGGA
CTTTTGTCCATTTGGTTGGTTATTGGACATAAGTAATGATTAATAGGGACAGTTGGGGGCATTAGTATTTAATTGTCAGAGGTGAAATT
CTTGGATTTATTAAAGACTAACTTATGCGAAAGCATTTGCCAAGGATGTTTTCATTAATCAAGAACGAAAGTTAAGGGATCAAAGACGA
TCAGATACCGTAGTAGTCTTAACTATAAACTATACCGACTCGGGATTGGATGGGTATATGCCCATTCAGCACCGTATGAGAAATCAAAG
TCTTTGGGTTCTGGGGGGAGTATGGTCGCAAGGCTGAAACTTAAAGGAATTGACGGAAGGGCACCACCAGGAGTGGAGCCTGCGGCTTA
ATTTGACTCAACACGGGGAAACTTACCAGGTCAAAACATGGGTGGGATTGACAGATTGAGAGCTCTTTCTTGATTCTATGGGTGGTGGT
GCATGGCCGTTCTTAGTTGGTGGAGTGATTTGTCTGGTTAATTCCGTTAACGAACGAGACCTTAACCTGCTAACTAGTCTTTTAGTATA
ATTACTAAAAGTACTTCTTAGAGGGACTATGCGACGAAACGCATGGAAGTTTGAGGCAATAACAGGTCTGTGATGCCCTTAGATGTCCT
GGGCCGCACGTGCGCTACAATGACACGTTCAACAAGTTTTTTCCTGGCCCGGAAGGGTACGGGTAATCTTTTTAATGCGTGTCGTGTTA
GGGATAGATCTTTGGAATTATAGATCTTGAACGAGGAATTCCTAGTAAGTGCAAGTCATCAACTTGTACTGATTACGTCCCTGCCCTTT
GTACACACCGCCCGTCGCTCCTACCGATTTCGAGTGATCCGGTGAACCTTCTGGACTAGGTCGTCCTTGAGACGGACTTGGGAAGTTAA
GTAAACCTTATCACTTAGAGGAAGGAGAAGTCGTAACAAGGTTTCCGTAGGTGAACCTGCAGAAGGATCA
>Haptophrya_planariarum_OL_11_DG
AACCTGGTTGATCCTGCCAGTAGTCATATGCTTGTCTCAAAGATTAAGCCATGCATGTCTAAGTATAATAGTATACAGTAAAACTGCGA
ATGGCTCATTACAACAGTTATAGTTTATTTGATAATGGAAGTCTACATGGATAACCGTGGTAATTCTAGAGCTAATACATGCTGTTAAA
CCTAACTTTACGGAAGGGTTGTATTTATTAGATATTAAGCCAATATTCTTTCGGGATTATTGCGAGGAATCATAATAACTGATCGAATC
TCAAATTTGAGATAAATCATTCAAGTTTCTGCCCTATCAGCTTTCGATGGTAGTGTATTGGACTACCATGGCAGTCACGGGTAACGGAG
AATTAGGGTTCGGTTCCGGAGAGGGAGCCTGAGAAACGGCTACCACATCTAAGGAAGGCAGCAGGCGCGTAAATTACCCAATCCTGATT
CAGGGAGGTAGTGACAAGAAATAACAACTCGGGGATTTTTAATCTTACGGGATTGCAATGAGAACAATTTAAACCACTTAGCGAGGAAC
AATTGGAGGGCAAGTCTGGTGCCAGCAGCCGCGGTAATTCCAGCTCCAATAGCGTATATTAAAGTTGTTGCAGTTAAAAAGCTCGTAGT
TGAATTTCTGGCTTAGCTTAAACGCGGCTTCGGTCAGTTTAAGATAAGTCATCCGTCTGCAAACCATATTCGTCCTTCACGGGTTCGAT
TTGGGGAGTAGATTTTTTACTTTGAAAAAATTAGAGTGTTTCAGGCAGGTTATCGCCCGAATACATTAGCATGGAATAATGGAATAGGA
CTTTTGTCCATTTGGTTGGTTATTGGACATAAGTAATGATTAATAGGGACAGTTGGGGGCATTAGTATTTAATTGTCAGAGGTGAAATT
CTTGGATTTATTAAAGACTAACTTATGCGAAAGCATTTGCCAAGGATGTTTTCATTAATCAAGAACGAAAGTTAAGGGATCAAAGACGA
TCAGATACCGTAGTAGTCTTAACTATAAACTATACCGACTCGGGATTGGATGGGTATATGCCCATTCAGCACCGTATGAGAAATCAAAG
TCTTTGGGTTCTGGGGGGAGTATGGTCGCAAGGCTGAAACTTAAAGGAATTGACGGAAGGGCACCACCAGGAGTGGAGCCTGCGGCTTA
ATTTGACTCAACACGGGGAAACTTACCAGGTCAAAACATGGGTGGGATTGACAGATTGAGAGCTCTTTCTTGATTCTATGGGTGGTGGT
GCATGGCCGTTCTTAGTTGGTGGAGTGATTTGTCTGGTTAATTCCGTTAACGAACGAGACCTTAACCTGCTAACTAGTCTTTTAGTATA
ATTACTAAAAGTACTTCTTAGAGGGACTATGCGACGAAACGCATGGAAGTTTGAGGCAATAACAGGTCTGTGATGCCCTTAGATGTCCT
GGGCCGCACGTGCGCTACAATGACACGTTCAACAAGTTTTTTCCTGGCCCGGAAGGGTACGGGTAATCTTTTTAATGCGTGTCGTGTTA
GGGATAGATCTTTGGAATTATAGATCTTGAACGAGGAATTCCTAGTAAGTGCAAGTCATCAACTTGTACTGATTACGTCCCTGCCCTTT
GTACACACCGCCCGTCGCTCCTACCGATTTCGAGTGATCCGGTGAACCTTCTGGACTAGGTCGTCCTTGAGACGGACTTGGGAAGTTAA
GTAAACCTTATCACTTAGAGGAAGGAGAAGTCGTAACAAGGTTTCCGTAGGTGAACCTGCAGAAGGATCA
>Haptophrya_planariarum_MI_12_DG
AACCTGGTTGATCCTGCCAGTAGTCATATGCTTGTCTCAAAGATTAAGCCATGCATGTCTAAGTATAATAGTATACAGTAAAACTGCGA
ATGGCTCATTACAACAGTTATAGTTTATTTGATAATGGAAGTCTACATGGATAACCGTGGTAATTCTAGAGCTAATACATGCTGTTAAA
CCTAACTTTACGGAAGGGTTGTATTTATTAGATATTAAGCCAATATTCTTTCGGGATTATTGCGAGGAATCATAATAACTGATCGAATC
TCAAATTTGAGATAAATCATTCAAGTTTCTGCCCTATCAGCTTTCGATGGTAGTGTATTGGACTACCATGGCAGTCACGGGTAACGGAG
AATTAGGGTTCGGTTCCGGAGAGGGAGCCTGAGAAACGGCTACCACATCTAAGGAAGGCAGCAGGCGCGTAAATTACCCAATCCTGATT
CAGGGAGGTAGTGACAAGAAATAACAACTCGGGGATTTTTAATCTTACGGGATTGCAATGAGAACAATTTAAACCACTTAGCGAGGAAC
AATTGGAGGGCAAGTCTGGTGCCAGCAGCCGCGGTAATTCCAGCTCCAATAGCGTATATTAAAGTTGTTGCAGTTAAAAAGCTCGTAGT
TGAATTTCTGGCTTAGCTTAAACGCGGCTTCGGTCAGTTTAAGATAAGTCATCCGTCTGCAAACCATATTCGTCCTTCACGGGTTCGAT
TTGGGGAGTAGATTTTTTACTTTGAAAAAATTAGAGTGTTTCAGGCAGGTTATCGCCCGAATACATTAGCATGGAATAATGGAATAGGA
CTTTTGTCCATTTGGTTGGTTATTGGACATAAGTAATGATTAATAGGGACAGTTGGGGGCATTAGTATTTAATTGTCAGAGGTGAAATT
CTTGGATTTATTAAAGACTAACTTATGCGAAAGCATTTGCCAAGGATGTTTTCATTAATCAAGAACGAAAGTTAAGGGATCAAAGACGA
TCAGATACCGTAGTAGTCTTAACTATAAACTATACCGACTCGGGATTGGATGGGTATATGCCCATTCAGCACCGTATGAGAAATCAAAG
TCTTTGGGTTCTGGGGGGAGTATGGTCGCAAGGCTGAAACTTAAAGGAATTGACGGAAGGGCACCACCAGGAGTGGAGCCTGCGGCTTA
ATTTGACTCAACACGGGGAAACTTACCAGGTCAAAACATGGGTGGGATTGACAGATTGAGAGCTCTTTCTTGATTCTATGGGTGGTGGT
GCATGGCCGTTCTTAGTTGGTGGAGTGATTTGTCTGGTTAATTCCGTTAACGAACGAGACCTTAACCTGCTAACTAGTCTTTTAGTATA
ATTACTAAAAGTACTTCTTAGAGGGACTATGCGACGAAACGCATGGAAGTTTGAGGCAATAACAGGTCTGTGATGCCCTTAGATGTCCT
GGGCCGCACGTGCGCTACAATGACACGTTCAACAAGTTTTTTCCTGGCCCGGAAGGGTACGGGTAATCTTTTTAATGCGTGTCGTGTTA
GGGATAGATCTTTGGAATTATAGATCTTGAACGAGGAATTCCTAGTAAGTGCAAGTCATCAACTTGTACTGATTACGTCCCTGCCCTTT
GTACACACCGCCCGTCGCTCCTACCGATTTCGAGTGATCCGGTGAACCTTCTGGACTAGGTCGTCCTTGAGACGGACTTGGGAAGTTAA
GTAAACCTTATCACTTAGAGGAAGGAGAAGTCGTAACAAGGTTTCCGTAGGTGAACCTGCAGAAGGATCA

>Haptophrya_planariarum_MI_16_DG
AACCTGGTTGATCCTGCCAGTAGTCATATGCTTGTCTCAAAGATTAAGCCATGCATGTCTAAGTATAATAGTATACAGTAAAACTGCGA
ATGGCTCATTACAACAGTTATAGTTTATTTGATAATGGAAGTCTACATGGATAACCGTGGTAATTCTAGAGCTAATACATGCTGTTAAA
CCTAACTTTACGGAAGGGTTGTATTTATTAGATATTAAGCCAATATTCTTTCGGGATTATTGCGAGGAATCATAATAACTGATCGAATC
TCAAATTTGAGATAAATCATTCAAGTTTCTGCCCTATCAGCTTTCGATGGTAGTGTATTGGACTACCATGGCAGTCACGGGTAACGGAG
AATTAGGGTTCGGTTCCGGAGAGGGAGCCTGAGAAACGGCTACCACATCTAAGGAAGGCAGCAGGCGCGTAAATTACCCAATCCTGATT
CAGGGAGGTAGTGACAAGAAATAACAACTCGGGGATTTTTAATCTTACGGGATTGCAATGAGAACAATTTAAACCACTTAGCGAGGAAC
AATTGGAGGGCAAGTCTGGTGCCAGCAGCCGCGGTAATTCCAGCTCCAATAGCGTATATTAAAGTTGTTGCAGTTAAAAAGCTCGTAGT
TGAATTTCTGGCTTAGCTTAAACGCGGCTTCGGTCAGTTTAAGATAAGTCATCCGTCTGCAAACCATATTCGTCCTTCACGGGTTCGAT
TTGGGGAGTAGATTTTTTACTTTGAAAAAATTAGAGTGTTTCAGGCAGGTTATCGCCCGAATACATTAGCATGGAATAATGGAATAGGA
CTTTTGTCCATTTGGTTGGTTATTGGACATAAGTAATGATTAATAGGGACAGTTGGGGGCATTAGTATTTAATTGTCAGAGGTGAAATT
CTTGGATTTATTAAAGACTAACTTATGCGAAAGCATTTGCCAAGGATGTTTTCATTAATCAAGAACGAAAGTTAAGGGATCAAAGACGA
TCAGATACCGTAGTAGTCTTAACTATAAACTATACCGACTCGGGATTGGATGGGTATATGCCCATTCAGCACCGTATGAGAAATCAAAG
TCTTTGGGTTCTGGGGGGAGTATGGTCGCAAGGCTGAAACTTAAAGGAATTGACGGAAGGGCACCACCAGGAGTGGAGCCTGCGGCTTA
ATTTGACTCAACACGGGGAAACTTACCAGGTCAAAACATGGGTGGGATTGACAGATTGAGAGCTCTTTCTTGATTCTATGGGTGGTGGT
GCATGGCCGTTCTTAGTTGGTGGAGTGATTTGTCTGGTTAATTCCGTTAACGAACGAGACCTTAACCTGCTAACTAGTCTTTTAGTATA
ATTACTAAAAGTACTTCTTAGAGGGACTATGCGACGAAACGCATGGAAGTTTGAGGCAATAACAGGTCTGTGATGCCCTTAGATGTCCT
GGGCCGCACGTGCGCTACAATGACACGTTCAACAAGTTTTTTCCTGGCCCGGAAGGGTACGGGTAATCTTTTTAATGCGTGTCGTGTTA
GGGATAGATCTTTGGAATTATAGATCTTGAACGAGGAATTCCTAGTAAGTGCAAGTCATCAACTTGTACTGATTACGTCCCTGCCCTTT
GTACACACCGCCCGTCGCTCCTACCGATTTCGAGTGATCCGGTGAACCTTCTGGACTAGGTCGTCCTTGAGACGGACTTGGGAAGTTAA
GTAAACCTTATCACTTAGAGGAAGGAGAAGTCGTAACAAGGTTTCCGTAGGTGAACCTGCAGAAGGATCA
>Haptophrya_planariarum_DL_19_DG
AACCTGGTTGATCCTGCCAGTAGTCATATGCTTGTCTCAAAGATTAAGCCATGCATGTCTAAGTATAATAGTATACAGTAAAACTGCGA
ATGGCTCATTACAACAGTTATAGTTTATTTGATAATGGAAGTCTACATGGATAACCGTGGTAATTCTAGAGCTAATACATGCTGTTAAA
CCTAACTTTACGGAAGGGTTGTATTTATTAGATATTAAGCCAATATTCTTTCGGGATTATTGCGAGGAATCATAATAACTGATCGAATC
TCAAATTTGAGATAAATCATTCAAGTTTCTGCCCTATCAGCTTTCGATGGTAGTGTATTGGACTACCATGGCAGTCACGGGTAACGGAG
AATTAGGGTTCGGTTCCGGAGAGGGAGCCTGAGAAACGGCTACCACATCTAAGGAAGGCAGCAGGCGCGTAAATTACCCAATCCTGATT
CAGGGAGGTAGTGACAAGAAATAACAACTCGGGGATTTTTAATCTTACGGGATTGCAATGAGAACAATTTAAACCACTTAGCGAGGAAC
AATTGGAGGGCAAGTCTGGTGCCAGCAGCCGCGGTAATTCCAGCTCCAATAGCGTATATTAAAGTTGTTGCAGTTAAAAAGCTCGTAGT
TGAATTTCTGGCTTAGCTTAAACGCGGCTTCGGTCAGTTTAAGATAAGTCATCCGTCTGCAAACCATATTCGTCCTTCACGGGTTCGAT
TTGGGGAGTAGATTTTTTACTTTGAAAAAATTAGAGTGTTTCAGGCAGGTTATCGCCCGAATACATTAGCATGGAATAATGGAATAGGA
CTTTTGTCCATTTGGTTGGTTATTGGACATAAGTAATGATTAATAGGGACAGTTGGGGGCATTAGTATTTAATTGTCAGAGGTGAAATT
CTTGGATTTATTAAAGACTAACTTATGCGAAAGCATTTGCCAAGGATGTTTTCATTAATCAAGAACGAAAGTTAAGGGATCAAAGACGA
TCAGATACCGTAGTAGTCTTAACTATAAACTATACCGACTCGGGATTGGATGGGTATATGCCCATTCAGCACCGTATGAGAAATCAAAG
TCTTTGGGTTCTGGGGGGAGTATGGTCGCAAGGCTGAAACTTAAAGGAATTGACGGAAGGGCACCACCAGGAGTGGAGCCTGCGGCTTA
ATTTGACTCAACACGGGGAAACTTACCAGGTCAAAACATGGGTGGGATTGACAGATTGAGAGCTCTTTCTTGATTCTATGGGTGGTGGT
GCATGGCCGTTCTTAGTTGGTGGAGTGATTTGTCTGGTTAATTCCGTTAACGAACGAGACCTTAACCTGCTAACTAGTCTTTTAGTATA
ATTACTAAAAGTACTTCTTAGAGGGACTATGCGACGAAACGCATGGAAGTTTGAGGCAATAACAGGTCTGTGATGCCCTTAGATGTCCT
GGGCCGCACGTGCGCTACAATGACACGTTCAACAAGTTTTTTCCTGGCCCGGAAGGGTACGGGTAATCTTTTTAATGCGTGTCGTGTTA
GGGATAGATCTTTGGAATTATAGATCTTGAACGAGGAATTCCTAGTAAGTGCAAGTCATCAACTTGTACTGATTACGTCCCTGCCCTTT
GTACACACCGCCCGTCGCTCCTACCGATTTCGAGTGATCCGGTGAACCTTCTGGACTAGGTCGTCCTTGAGACGGACTTGGGAAGTTAA
GTAAACCTTATCACTTAGAGGAAGGAGAAGTCGTAACAAGGTTTCCGTAGGTGAACCTGCAGAAGGATCA
>Haptophrya_planariarum_DL_20_DG
AACCTGGTTGATCCTGCCAGTAGTCATATGCTTGTCTCAAAGATTAAGCCATGCATGTCTAAGTATAATAGTATACAGTAAAACTGCGA
ATGGCTCATTACAACAGTTATAGTTTATTTGATAATGGAAGTCTACATGGATAACCGTGGTAATTCTAGAGCTAATACATGCTGTTAAA
CCTAACTTTACGGAAGGGTTGTATTTATTAGATATTAAGCCAATATTCTTTCGGGATTATTGCGAGGAATCATAATAACTGATCGAATC
TCAAATTTGAGATAAATCATTCAAGTTTCTGCCCTATCAGCTTTCGATGGTAGTGTATTGGACTACCATGGCAGTCACGGGTAACGGAG
AATTAGGGTTCGGTTCCGGAGAGGGAGCCTGAGAAACGGCTACCACATCTAAGGAAGGCAGCAGGCGCGTAAATTACCCAATCCTGATT
CAGGGAGGTAGTGACAAGAAATAACAACTCGGGGATTTTTAATCTTACGGGATTGCAATGAGAACAATTTAAACCACTTAGCGAGGAAC
AATTGGAGGGCAAGTCTGGTGCCAGCAGCCGCGGTAATTCCAGCTCCAATAGCGTATATTAAAGTTGTTGCAGTTAAAAAGCTCGTAGT
TGAATTTCTGGCTTAGCTTAAACGCGGCTTCGGTCAGTTTAAGATAAGTCATCCGTCTGCAAACCATATTCGTCCTTCACGGGTTCGAT
TTGGGGAGTAGATTTTTTACTTTGAAAAAATTAGAGTGTTTCAGGCAGGTTATCGCCCGAATACATTAGCATGGAATAATGGAATAGGA
CTTTTGTCCATTTGGTTGGTTATTGGACATAAGTAATGATTAATAGGGACAGTTGGGGGCATTAGTATTTAATTGTCAGAGGTGAAATT
CTTGGATTTATTAAAGACTAACTTATGCGAAAGCATTTGCCAAGGATGTTTTCATTAATCAAGAACGAAAGTTAAGGGATCAAAGACGA
TCAGATACCGTAGTAGTCTTAACTATAAACTATACCGACTCGGGATTGGATGGGTATATGCCCATTCAGCACCGTATGAGAAATCAAAG
TCTTTGGGTTCTGGGGGGAGTATGGTCGCAAGGCTGAAACTTAAAGGAATTGACGGAAGGGCACCACCAGGAGTGGAGCCTGCGGCTTA
ATTTGACTCAACACGGGGAAACTTACCAGGTCAAAACATGGGTGGGATTGACAGATTGAGAGCTCTTTCTTGATTCTATGGGTGGTGGT
GCATGGCCGTTCTTAGTTGGTGGAGTGATTTGTCTGGTTAATTCCGTTAACGAACGAGACCTTAACCTGCTAACTAGTCTTTTAGTATA
ATTACTAAAAGTACTTCTTAGAGGGACTATGCGACGAAACGCATGGAAGTTTGAGGCAATAACAGGTCTGTGATGCCCTTAGATGTCCT
GGGCCGCACGTGCGCTACAATGACACGTTCAACAAGTTTTTTCCTGGCCCGGAAGGGTACGGGTAATCTTTTTAATGCGTGTCGTGTTA
GGGATAGATCTTTGGAATTATAGATCTTGAACGAGGAATTCCTAGTAAGTGCAAGTCATCAACTTGTACTGATTACGTCCCTGCCCTTT
GTACACACCGCCCGTCGCTCCTACCGATTTCGAGTGATCCGGTGAACCTTCTGGACTAGGTCGTCCTTGAGACGGACTTGGGAAGTTAA
GTAAACCTTATCACTTAGAGGAAGGAGAAGTCGTAACAAGGTTTCCGTAGGTGAACCTGCAGAAGGATCA
>Haptophrya_planariarum_DL_21_DG
AACCTGGTTGATCCTGCCAGTAGTCATATGCTTGTCTCAAAGATTAAGCCATGCATGTCTAAGTATAATAGTATACAGTAAAACTGCGA
ATGGCTCATTACAACAGTTATAGTTTATTTGATAATGGAAGTCTACATGGATAACCGTGGTAATTCTAGAGCTAATACATGCTGTTAAA
CCTAACTTTACGGAAGGGTTGTATTTATTAGATATTAAGCCAATATTCTTTCGGGATTATTGCGAGGAATCATAATAACTGATCGAATC
TCAAATTTGAGATAAATCATTCAAGTTTCTGCCCTATCAGCTTTCGATGGTAGTGTATTGGACTACCATGGCAGTCACGGGTAACGGAG
AATTAGGGTTCGGTTCCGGAGAGGGAGCCTGAGAAACGGCTACCACATCTAAGGAAGGCAGCAGGCGCGTAAATTACCCAATCCTGATT
CAGGGAGGTAGTGACAAGAAATAACAACTCGGGGATTTTTAATCTTACGGGATTGCAATGAGAACAATTTAAACCACTTAGCGAGGAAC
AATTGGAGGGCAAGTCTGGTGCCAGCAGCCGCGGTAATTCCAGCTCCAATAGCGTATATTAAAGTTGTTGCAGTTAAAAAGCTCGTAGT

TGAATTTCTGGCTTAGCTTAAACGCGGCTTCGGTCAGTTTAAGATAAGTCATCCGTCTGCAAACCATATTCGTCCTTCACGGGTTCGAT
TTGGGGAGTAGATTTTTTACTTTGAAAAAATTAGAGTGTTTCAGGCAGGTTATCGCCCGAATACATTAGCATGGAATAATGGAATAGGA
CTTTTGTCCATTTGGTTGGTTATTGGACATAAGTAATGATTAATAGGGACAGTTGGGGGCATTAGTATTTAATTGTCAGAGGTGAAATT
CTTGGATTTATTAAAGACTAACTTATGCGAAAGCATTTGCCAAGGATGTTTTCATTAATCAAGAACGAAAGTTAAGGGATCAAAGACGA
TCAGATACCGTAGTAGTCTTAACTATAAACTATACCGACTCGGGATTGGATGGGTATATGCCCATTCAGCACCGTATGAGAAATCAAAG
TCTTTGGGTTCTGGGGGGAGTATGGTCGCAAGGCTGAAACTTAAAGGAATTGACGGAAGGGCACCACCAGGAGTGGAGCCTGCGGCTTA
ATTTGACTCAACACGGGGAAACTTACCAGGTCAAAACATGGGTGGGATTGACAGATTGAGAGCTCTTTCTTGATTCTATGGGTGGTGGT
GCATGGCCGTTCTTAGTTGGTGGAGTGATTTGTCTGGTTAATTCCGTTAACGAACGAGACCTTAACCTGCTAACTAGTCTTTTAGTATA
ATTACTAAAAGTACTTCTTAGAGGGACTATGCGACGAAACGCATGGAAGTTTGAGGCAATAACAGGTCTGTGATGCCCTTAGATGTCCT
GGGCCGCACGTGCGCTACAATGACACGTTCAACAAGTTTTTTCCTGGCCCGGAAGGGTACGGGTAATCTTTTTAATGCGTGTCGTGTTA
GGGATAGATCTTTGGAATTATAGATCTTGAACGAGGAATTCCTAGTAAGTGCAAGTCATCAACTTGTACTGATTACGTCCCTGCCCTTT
GTACACACCGCCCGTCGCTCCTACCGATTTCGAGTGATCCGGTGAACCTTCTGGACTAGGTCGTCCTTGAGACGGACTTGGGAAGTTAA
GTAAACCTTATCACTTAGAGGAAGGAGAAGTCGTAACAAGGTTTCCGTAGGTGAACCTGCAGAAGGATCA
>Haptophrya_planariarum_DL_22_DG
AACCTGGTTGATCCTGCCAGTAGTCATATGCTTGTCTCAAAGATTAAGCCATGCATGTCTAAGTATAATAGTATACAGTAAAACTGCGA
ATGGCTCATTACAACAGTTATAGTTTATTTGATAATGGAAGTCTACATGGATAACCGTGGTAATTCTAGAGCTAATACATGCTGTTAAA
CCTAACTTTACGGAAGGGTTGTATTTATTAGATATTAAGCCAATATTCTTTCGGGATTATTGCGAGGAATCATAATAACTGATCGAATC
TCAAATTTGAGATAAATCATTCAAGTTTCTGCCCTATCAGCTTTCGATGGTAGTGTATTGGACTACCATGGCAGTCACGGGTAACGGAG
AATTAGGGTTCGGTTCCGGAGAGGGAGCCTGAGAAACGGCTACCACATCTAAGGAAGGCAGCAGGCGCGTAAATTACCCAATCCTGATT
CAGGGAGGTAGTGACAAGAAATAACAACTCGGGGATTTTTAATCTTACGGGATTGCAATGAGAACAATTTAAACCACTTAGCGAGGAAC
AATTGGAGGGCAAGTCTGGTGCCAGCAGCCGCGGTAATTCCAGCTCCAATAGCGTATATTAAAGTTGTTGCAGTTAAAAAGCTCGTAGT
TGAATTTCTGGCTTAGCTTAAACGCGGCTTCGGTCAGTTTAAGATAAGTCATCCGTCTGCAAACCATATTCGTCCTTCACGGGTTCGAT
TTGGGGAGTAGATTTTTTACTTTGAAAAAATTAGAGTGTTTCAGGCAGGTTATCGCCCGAATACATTAGCATGGAATAATGGAATAGGA
CTTTTGTCCATTTGGTTGGTTATTGGACATAAGTAATGATTAATAGGGACAGTTGGGGGCATTAGTATTTAATTGTCAGAGGTGAAATT
CTTGGATTTATTAAAGACTAACTTATGCGAAAGCATTTGCCAAGGATGTTTTCATTAATCAAGAACGAAAGTTAAGGGATCAAAGACGA
TCAGATACCGTAGTAGTCTTAACTATAAACTATACCGACTCGGGATTGGATGGGTATATGCCCATTCAGCACCGTATGAGAAATCAAAG
TCTTTGGGTTCTGGGGGGAGTATGGTCGCAAGGCTGAAACTTAAAGGAATTGACGGAAGGGCACCACCAGGAGTGGAGCCTGCGGCTTA
ATTTGACTCAACACGGGGAAACTTACCAGGTCAAAACATGGGTGGGATTGACAGATTGAGAGCTCTTTCTTGATTCTATGGGTGGTGGT
GCATGGCCGTTCTTAGTTGGTGGAGTGATTTGTCTGGTTAATTCCGTTAACGAACGAGACCTTAACCTGCTAACTAGTCTTTTAGTATA
ATTACTAAAAGTACTTCTTAGAGGGACTATGCGACGAAACGCATGGAAGTTTGAGGCAATAACAGGTCTGTGATGCCCTTAGATGTCCT
GGGCCGCACGTGCGCTACAATGACACGTTCAACAAGTTTTTTCCTGGCCCGGAAGGGTACGGGTAATCTTTTTAATGCGTGTCGTGTTA
GGGATAGATCTTTGGAATTATAGATCTTGAACGAGGAATTCCTAGTAAGTGCAAGTCATCAACTTGTACTGATTACGTCCCTGCCCTTT
GTACACACCGCCCGTCGCTCCTACCGATTTCGAGTGATCCGGTGAACCTTCTGGACTAGGTCGTCCTTGAGACGGACTTGGGAAGTTAA
GTAAACCTTATCACTTAGAGGAAGGAGAAGTCGTAACAAGGTTTCCGTAGGTGAACCTGCAGAAGGATCA
>Haptophrya_planariarum_DL_23_DG
AACCTGGTTGATCCTGCCAGTAGTCATATGCTTGTCTCAAAGATTAAGCCATGCATGTCTAAGTATAATAGTATACAGTAAAACTGCGA
ATGGCTCATTACAACAGTTATAGTTTATTTGATAATGGAAGTCTACATGGATAACCGTGGTAATTCTAGAGCTAATACATGCTGTTAAA
CCTAACTTTACGGAAGGGTTGTATTTATTAGATATTAAGCCAATATTCTTTCGGGATTATTGCGAGGAATCATAATAACTGATCGAATC
TCAAATTTGAGATAAATCATTCAAGTTTCTGCCCTATCAGCTTTCGATGGTAGTGTATTGGACTACCATGGCAGTCACGGGTAACGGAG
AATTAGGGTTCGGTTCCGGAGAGGGAGCCTGAGAAACGGCTACCACATCTAAGGAAGGCAGCAGGCGCGTAAATTACCCAATCCTGATT
CAGGGAGGTAGTGACAAGAAATAACAACTCGGGGATTTTTAATCTTACGGGATTGCAATGAGAACAATTTAAACCACTTAGCGAGGAAC
AATTGGAGGGCAAGTCTGGTGCCAGCAGCCGCGGTAATTCCAGCTCCAATAGCGTATATTAAAGTTGTTGCAGTTAAAAAGCTCGTAGT
TGAATTTCTGGCTTAGCTTAAACGCGGCTTCGGTCAGTTTAAGATAAGTCATCCGTCTGCAAACCATATTCGTCCTTCACGGGTTCGAT
TTGGGGAGTAGATTTTTTACTTTGAAAAAATTAGAGTGTTTCAGGCAGGTTATCGCCCGAATACATTAGCATGGAATAATGGAATAGGA
CTTTTGTCCATTTGGTTGGTTATTGGACATAAGTAATGATTAATAGGGACAGTTGGGGGCATTAGTATTTAATTGTCAGAGGTGAAATT
CTTGGATTTATTAAAGACTAACTTATGCGAAAGCATTTGCCAAGGATGTTTTCATTAATCAAGAACGAAAGTTAAGGGATCAAAGACGA
TCAGATACCGTAGTAGTCTTAACTATAAACTATACCGACTCGGGATTGGATGGGTATATGCCCATTCAGCACCGTATGAGAAATCAAAG
TCTTTGGGTTCTGGGGGGAGTATGGTCGCAAGGCTGAAACTTAAAGGAATTGACGGAAGGGCACCACCAGGAGTGGAGCCTGCGGCTTA
ATTTGACTCAACACGGGGAAACTTACCAGGTCAAAACATGGGTGGGATTGACAGATTGAGAGCTCTTTCTTGATTCTATGGGTGGTGGT
GCATGGCCGTTCTTAGTTGGTGGAGTGATTTGTCTGGTTAATTCCGTTAACGAACGAGACCTTAACCTGCTAACTAGTCTTTTAGTATA
ATTACTAAAAGTACTTCTTAGAGGGACTATGCGACGAAACGCATGGAAGTTTGAGGCAATAACAGGTCTGTGATGCCCTTAGATGTCCT
GGGCCGCACGTGCGCTACAATGACACGTTCAACAAGTTTTTTCCTGGCCCGGAAGGGTACGGGTAATCTTTTTAATGCGTGTCGTGTTA
GGGATAGATCTTTGGAATTATAGATCTTGAACGAGGAATTCCTAGTAAGTGCAAGTCATCAACTTGTACTGATTACGTCCCTGCCCTTT
GTACACACCGCCCGTCGCTCCTACCGATTTCGAGTGATCCGGTGAACCTTCTGGACTAGGTCGTCCTTGAGACGGACTTGGGAAGTTAA
GTAAACCTTATCACTTAGAGGAAGGAGAAGTCGTAACAAGGTTTCCGTAGGTGAACCTGCAGAAGGATCA
>Haptophrya_planariarum_DL_24_DG
AACCTGGTTGATCCTGCCAGTAGTCATATGCTTGTCTCAAAGATTAAGCCATGCATGTCTAAGTATAATAGTATACAGTAAAACTGCGA
ATGGCTCATTACAACAGTTATAGTTTATTTGATAATGGAAGTCTACATGGATAACCGTGGTAATTCTAGAGCTAATACATGCTGTTAAA
CCTAACTTTACGGAAGGGTTGTATTTATTAGATATTAAGCCAATATTCTTTCGGGATTATTGCGAGGAATCATAATAACTGATCGAATC
TCAAATTTGAGATAAATCATTCAAGTTTCTGCCCTATCAGCTTTCGATGGTAGTGTATTGGACTACCATGGCAGTCACGGGTAACGGAG
AATTAGGGTTCGGTTCCGGAGAGGGAGCCTGAGAAACGGCTACCACATCTAAGGAAGGCAGCAGGCGCGTAAATTACCCAATCCTGATT
CAGGGAGGTAGTGACAAGAAATAACAACTCGGGGATTTTTAATCTTACGGGATTGCAATGAGAACAATTTAAACCACTTAGCGAGGAAC
AATTGGAGGGCAAGTCTGGTGCCAGCAGCCGCGGTAATTCCAGCTCCAATAGCGTATATTAAAGTTGTTGCAGTTAAAAAGCTCGTAGT
TGAATTTCTGGCTTAGCTTAAACGCGGCTTCGGTCAGTTTAAGATAAGTCATCCGTCTGCAAACCATATTCGTCCTTCACGGGTTCGAT
TTGGGGAGTAGATTTTTTACTTTGAAAAAATTAGAGTGTTTCAGGCAGGTTATCGCCCGAATACATTAGCATGGAATAATGGAATAGGA
CTTTTGTCCATTTGGTTGGTTATTGGACATAAGTAATGATTAATAGGGACAGTTGGGGGCATTAGTATTTAATTGTCAGAGGTGAAATT
CTTGGATTTATTAAAGACTAACTTATGCGAAAGCATTTGCCAAGGATGTTTTCATTAATCAAGAACGAAAGTTAAGGGATCAAAGACGA
TCAGATACCGTAGTAGTCTTAACTATAAACTATACCGACTCGGGATTGGATGGGTATATGCCCATTCAGCACCGTATGAGAAATCAAAG
TCTTTGGGTTCTGGGGGGAGTATGGTCGCAAGGCTGAAACTTAAAGGAATTGACGGAAGGGCACCACCAGGAGTGGAGCCTGCGGCTTA
ATTTGACTCAACACGGGGAAACTTACCAGGTCAAAACATGGGTGGGATTGACAGATTGAGAGCTCTTTCTTGATTCTATGGGTGGTGGT
GCATGGCCGTTCTTAGTTGGTGGAGTGATTTGTCTGGTTAATTCCGTTAACGAACGAGACCTTAACCTGCTAACTAGTCTTTTAGTATA

ATTACTAAAAGTACTTCTTAGAGGGACTATGCGACGAAACGCATGGAAGTTTGAGGCAATAACAGGTCTGTGATGCCCTTAGATGTCCT
GGGCCGCACGTGCGCTACAATGACACGTTCAACAAGTTTTTTCCTGGCCCGGAAGGGTACGGGTAATCTTTTTAATGCGTGTCGTGTTA
GGGATAGATCTTTGGAATTATAGATCTTGAACGAGGAATTCCTAGTAAGTGCAAGTCATCAACTTGTACTGATTACGTCCCTGCCCTTT
GTACACACCGCCCGTCGCTCCTACCGATTTCGAGTGATCCGGTGAACCTTCTGGACTAGGTCGTCCTTGAGACGGACTTGGGAAGTTAA
GTAAACCTTATCACTTAGAGGAAGGAGAAGTCGTAACAAGGTTTCCGTAGGTGAACCTGCAGAAGGATCA
>Haptophrya_planariarum_DL_25_DG
AACCTGGTTGATCCTGCCAGTAGTCATATGCTTGTCTCAAAGATTAAGCCATGCATGTCTAAGTATAATAGTATACAGTAAAACTGCGA
ATGGCTCATTACAACAGTTATAGTTTATTTGATAATGGAAGTCTACATGGATAACCGTGGTAATTCTAGAGCTAATACATGCTGTTAAA
CCTAACTTTACGGAAGGGTTGTATTTATTAGATATTAAGCCAATATTCTTTCGGGATTATTGCGAGGAATCATAATAACTGATCGAATC
TCAAATTTGAGATAAATCATTCAAGTTTCTGCCCTATCAGCTTTCGATGGTAGTGTATTGGACTACCATGGCAGTCACGGGTAACGGAG
AATTAGGGTTCGGTTCCGGAGAGGGAGCCTGAGAAACGGCTACCACATCTAAGGAAGGCAGCAGGCGCGTAAATTACCCAATCCTGATT
CAGGGAGGTAGTGACAAGAAATAACAACTCGGGGATTTTTAATCTTACGGGATTGCAATGAGAACAATTTAAACCACTTAGCGAGGAAC
AATTGGAGGGCAAGTCTGGTGCCAGCAGCCGCGGTAATTCCAGCTCCAATAGCGTATATTAAAGTTGTTGCAGTTAAAAAGCTCGTAGT
TGAATTTCTGGCTTAGCTTAAACGCGGCTTCGGTCAGTTTAAGATAAGTCATCCGTCTGCAAACCATATTCGTCCTTCACGGGTTCGAT
TTGGGGAGTAGATTTTTTACTTTGAAAAAATTAGAGTGTTTCAGGCAGGTTATCGCCCGAATACATTAGCATGGAATAATGGAATAGGA
CTTTTGTCCATTTGGTTGGTTATTGGACATAAGTAATGATTAATAGGGACAGTTGGGGGCATTAGTATTTAATTGTCAGAGGTGAAATT
CTTGGATTTATTAAAGACTAACTTATGCGAAAGCATTTGCCAAGGATGTTTTCATTAATCAAGAACGAAAGTTAAGGGATCAAAGACGA
TCAGATACCGTAGTAGTCTTAACTATAAACTATACCGACTCGGGATTGGATGGGTATATGCCCATTCAGCACCGTATGAGAAATCAAAG
TCTTTGGGTTCTGGGGGGAGTATGGTCGCAAGGCTGAAACTTAAAGGAATTGACGGAAGGGCACCACCAGGAGTGGAGCCTGCGGCTTA
ATTTGACTCAACACGGGGAAACTTACCAGGTCAAAACATGGGTGGGATTGACAGATTGAGAGCTCTTTCTTGATTCTATGGGTGGTGGT
GCATGGCCGTTCTTAGTTGGTGGAGTGATTTGTCTGGTTAATTCCGTTAACGAACGAGACCTTAACCTGCTAACTAGTCTTTTAGTATA
ATTACTAAAAGTACTTCTTAGAGGGACTATGCGACGAAACGCATGGAAGTTTGAGGCAATAACAGGTCTGTGATGCCCTTAGATGTCCT
GGGCCGCACGTGCGCTACAATGACACGTTCAACAAGTTTTTTCCTGGCCCGGAAGGGTACGGGTAATCTTTTTAATGCGTGTCGTGTTA
GGGATAGATCTTTGGAATTATAGATCTTGAACGAGGAATTCCTAGTAAGTGCAAGTCATCAACTTGTACTGATTACGTCCCTGCCCTTT
GTACACACCGCCCGTCGCTCCTACCGATTTCGAGTGATCCGGTGAACCTTCTGGACTAGGTCGTCCTTGAGACGGACTTGGGAAGTTAA
GTAAACCTTATCACTTAGAGGAAGGAGAAGTCGTAACAAGGTTTCCGTAGGTGAACCTGCAGAAGGATCA
>Haptophrya_planariarum_KD_26_DG
AACCTGGTTGATCCTGCCAGTAGTCATATGCTTGTCTCAAAGATTAAGCCATGCATGTCTAAGTATAATAGTATACAGTAAAACTGCGA
ATGGCTCATTACAACAGTTATAGTTTATTTGATAATGGAAGTCTACATGGATAACCGTGGTAATTCTAGAGCTAATACATGCTGTTAAA
CCTAACTTTACGGAAGGGTTGTATTTATTAGATATTAAGCCAATATTCTTTCGGGATTATTGCGAGGAATCATAATAACTGATCGAATC
TCAAATTTGAGATAAATCATTCAAGTTTCTGCCCTATCAGCTTTCGATGGTAGTGTATTGGACTACCATGGCAGTCACGGGTAACGGAG
AATTAGGGTTCGGTTCCGGAGAGGGAGCCTGAGAAACGGCTACCACATCTAAGGAAGGCAGCAGGCGCGTAAATTACCCAATCCTGATT
CAGGGAGGTAGTGACAAGAAATAACAACTCGGGGATTTTTAATCTTACGGGATTGCAATGAGAACAATTTAAACCACTTAGCGAGGAAC
AATTGGAGGGCAAGTCTGGTGCCAGCAGCCGCGGTAATTCCAGCTCCAATAGCGTATATTAAAGTTGTTGCAGTTAAAAAGCTCGTAGT
TGAATTTCTGGCTTAGCTTAAACGCGGCTTCGGTCAGTTTAAGATAAGTCATCCGTCTGCAAACCATATTCGTCCTTCACGGGTTCGAT
TTGGGGAGTAGATTTTTTACTTTGAAAAAATTAGAGTGTTTCAGGCAGGTTATCGCCCGAATACATTAGCATGGAATAATGGAATAGGA
CTTTTGTCCATTTGGTTGGTTATTGGACATAAGTAATGATTAATAGGGACAGTTGGGGGCATTAGTATTTAATTGTCAGAGGTGAAATT
CTTGGATTTATTAAAGACTAACTTATGCGAAAGCATTTGCCAAGGATGTTTTCATTAATCAAGAACGAAAGTTAAGGGATCAAAGACGA
TCAGATACCGTAGTAGTCTTAACTATAAACTATACCGACTCGGGATTGGATGGGTATATGCCCATTCAGCACCGTATGAGAAATCAAAG
TCTTTGGGTTCTGGGGGGAGTATGGTCGCAAGGCTGAAACTTAAAGGAATTGACGGAAGGGCACCACCAGGAGTGGAGCCTGCGGCTTA
ATTTGACTCAACACGGGGAAACTTACCAGGTCAAAACATGGGTGGGATTGACAGATTGAGAGCTCTTTCTTGATTCTATGGGTGGTGGT
GCATGGCCGTTCTTAGTTGGTGGAGTGATTTGTCTGGTTAATTCCGTTAACGAACGAGACCTTAACCTGCTAACTAGTCTTTTAGTATA
ATTACTAAAAGTACTTCTTAGAGGGACTATGCGACGAAACGCATGGAAGTTTGAGGCAATAACAGGTCTGTGATGCCCTTAGATGTCCT
GGGCCGCACGTGCGCTACAATGACACGTTCAACAAGTTTTTTCCTGGCCCGGAAGGGTACGGGTAATCTTTTTAATGCGTGTCGTGTTA
GGGATAGATCTTTGGAATTATAGATCTTGAACGAGGAATTCCTAGTAAGTGCAAGTCATCAACTTGTACTGATTACGTCCCTGCCCTTT
GTACACACCGCCCGTCGCTCCTACCGATTTCGAGTGATCCGGTGAACCTTCTGGACTAGGTCGTCCTTGAGACGGACTTGGGAAGTTAA
GTAAACCTTATCACTTAGAGGAAGGAGAAGTCGTAACAAGGTTTCCGTAGGTGAACCTGCAGAAGGATCA
>Haptophrya_planariarum_KD_27_DG
AACCTGGTTGATCCTGCCAGTAGTCATATGCTTGTCTCAAAGATTAAGCCATGCATGTCTAAGTATAATAGTATACAGTAAAACTGCGA
ATGGCTCATTACAACAGTTATAGTTTATTTGATAATGGAAGTCTACATGGATAACCGTGGTAATTCTAGAGCTAATACATGCTGTTAAA
CCTAACTTTACGGAAGGGTTGTATTTATTAGATATTAAGCCAATATTCTTTCGGGATTATTGCGAGGAATCATAATAACTGATCGAATC
TCAAATTTGAGATAAATCATTCAAGTTTCTGCCCTATCAGCTTTCGATGGTAGTGTATTGGACTACCATGGCAGTCACGGGTAACGGAG
AATTAGGGTTCGGTTCCGGAGAGGGAGCCTGAGAAACGGCTACCACATCTAAGGAAGGCAGCAGGCGCGTAAATTACCCAATCCTGATT
CAGGGAGGTAGTGACAAGAAATAACAACTCGGGGATTTTTAATCTTACGGGATTGCAATGAGAACAATTTAAACCACTTAGCGAGGAAC
AATTGGAGGGCAAGTCTGGTGCCAGCAGCCGCGGTAATTCCAGCTCCAATAGCGTATATTAAAGTTGTTGCAGTTAAAAAGCTCGTAGT
TGAATTTCTGGCTTAGCTTAAACGCGGCTTCGGTCAGTTTAAGATAAGTCATCCGTCTGCAAACCATATTCGTCCTTCACGGGTTCGAT
TTGGGGAGTAGATTTTTTACTTTGAAAAAATTAGAGTGTTTCAGGCAGGTTATCGCCCGAATACATTAGCATGGAATAATGGAATAGGA
CTTTTGTCCATTTGGTTGGTTATTGGACATAAGTAATGATTAATAGGGACAGTTGGGGGCATTAGTATTTAATTGTCAGAGGTGAAATT
CTTGGATTTATTAAAGACTAACTTATGCGAAAGCATTTGCCAAGGATGTTTTCATTAATCAAGAACGAAAGTTAAGGGATCAAAGACGA
TCAGATACCGTAGTAGTCTTAACTATAAACTATACCGACTCGGGATTGGATGGGTATATGCCCATTCAGCACCGTATGAGAAATCAAAG
TCTTTGGGTTCTGGGGGGAGTATGGTCGCAAGGCTGAAACTTAAAGGAATTGACGGAAGGGCACCACCAGGAGTGGAGCCTGCGGCTTA
ATTTGACTCAACACGGGGAAACTTACCAGGTCAAAACATGGGTGGGATTGACAGATTGAGAGCTCTTTCTTGATTCTATGGGTGGTGGT
GCATGGCCGTTCTTAGTTGGTGGAGTGATTTGTCTGGTTAATTCCGTTAACGAACGAGACCTTAACCTGCTAACTAGTCTTTTAGTATA
ATTACTAAAAGTACTTCTTAGAGGGACTATGCGACGAAACGCATGGAAGTTTGAGGCAATAACAGGTCTGTGATGCCCTTAGATGTCCT
GGGCCGCACGTGCGCTACAATGACACGTTCAACAAGTTTTTTCCTGGCCCGGAAGGGTACGGGTAATCTTTTTAATGCGTGTCGTGTTA
GGGATAGATCTTTGGAATTATAGATCTTGAACGAGGAATTCCTAGTAAGTGCAAGTCATCAACTTGTACTGATTACGTCCCTGCCCTTT
GTACACACCGCCCGTCGCTCCTACCGATTTCGAGTGATCCGGTGAACCTTCTGGACTAGGTCGTCCTTGAGACGGACTTGGGAAGTTAA
GTAAACCTTATCACTTAGAGGAAGGAGAAGTCGTAACAAGGTTTCCGTAGGTGAACCTGCAGAAGGATCA
>Haptophrya_planariarum_DL_28_DG
AACCTGGTTGATCCTGCCAGTAGTCATATGCTTGTCTCAAAGATTAAGCCATGCATGTCTAAGTATAATAGTATACAGTAAAACTGCGA
ATGGCTCATTACAACAGTTATAGTTTATTTGATAATGGAAGTCTACATGGATAACCGTGGTAATTCTAGAGCTAATACATGCTGTTAAA

CCTAACTTTACGGAAGGGTTGTATTTATTAGATATTAAGCCAATATTCTTTCGGGATTATTGCGAGGAATCATAATAACTGATCGAATC
TCAAATTTGAGATAAATCATTCAAGTTTCTGCCCTATCAGCTTTCGATGGTAGTGTATTGGACTACCATGGCAGTCACGGGTAACGGAG
AATTAGGGTTCGGTTCCGGAGAGGGAGCCTGAGAAACGGCTACCACATCTAAGGAAGGCAGCAGGCGCGTAAATTACCCAATCCTGATT
CAGGGAGGTAGTGACAAGAAATAACAACTCGGGGATTTTTAATCTTACGGGATTGCAATGAGAACAATTTAAACCACTTAGCGAGGAAC
AATTGGAGGGCAAGTCTGGTGCCAGCAGCCGCGGTAATTCCAGCTCCAATAGCGTATATTAAAGTTGTTGCAGTTAAAAAGCTCGTAGT
TGAATTTCTGGCTTAGCTTAAACGCGGCTTCGGTCAGTTTAAGATAAGTCATCCGTCTGCAAACCATATTCGTCCTTCACGGGTTCGAT
TTGGGGAGTAGATTTTTTACTTTGAAAAAATTAGAGTGTTTCAGGCAGGTTATCGCCCGAATACATTAGCATGGAATAATGGAATAGGA
CTTTTGTCCATTTGGTTGGTTATTGGACATAAGTAATGATTAATAGGGACAGTTGGGGGCATTAGTATTTAATTGTCAGAGGTGAAATT
CTTGGATTTATTAAAGACTAACTTATGCGAAAGCATTTGCCAAGGATGTTTTCATTAATCAAGAACGAAAGTTAAGGGATCAAAGACGA
TCAGATACCGTAGTAGTCTTAACTATAAACTATACCGACTCGGGATTGGATGGGTATATGCCCATTCAGCACCGTATGAGAAATCAAAG
TCTTTGGGTTCTGGGGGGAGTATGGTCGCAAGGCTGAAACTTAAAGGAATTGACGGAAGGGCACCACCAGGAGTGGAGCCTGCGGCTTA
ATTTGACTCAACACGGGGAAACTTACCAGGTCAAAACATGGGTGGGATTGACAGATTGAGAGCTCTTTCTTGATTCTATGGGTGGTGGT
GCATGGCCGTTCTTAGTTGGTGGAGTGATTTGTCTGGTTAATTCCGTTAACGAACGAGACCTTAACCTGCTAACTAGTCTTTTAGTATA
ATTACTAAAAGTACTTCTTAGAGGGACTATGCGACGAAACGCATGGAAGTTTGAGGCAATAACAGGTCTGTGATGCCCTTAGATGTCCT
GGGCCGCACGTGCGCTACAATGACACGTTCAACAAGTTTTTTCCTGGCCCGGAAGGGTACGGGTAATCTTTTTAATGCGTGTCGTGTTA
GGGATAGATCTTTGGAATTATAGATCTTGAACGAGGAATTCCTAGTAAGTGCAAGTCATCAACTTGTACTGATTACGTCCCTGCCCTTT
GTACACACCGCCCGTCGCTCCTACCGATTTCGAGTGATCCGGTGAACCTTCTGGACTAGGTCGTCCTTGAGACGGACTTGGGAAGTTAA
GTAAACCTTATCACTTAGAGGAAGGAGAAGTCGTAACAAGGTTTCCGTAGGTGAACCTGCAGAAGGATCA
>Haptophrya_planariarum_KD_29_DG
AACCTGGTTGATCCTGCCAGTAGTCATATGCTTGTCTCAAAGATTAAGCCATGCATGTCTAAGTATAATAGTATACAGTAAAACTGCGA
ATGGCTCATTACAACAGTTATAGTTTATTTGATAATGGAAGTCTACATGGATAACCGTGGTAATTCTAGAGCTAATACATGCTGTTAAA
CCTAACTTTACGGAAGGGTTGTATTTATTAGATATTAAGCCAATATTCTTTCGGGATTATTGCGAGGAATCATAATAACTGATCGAATC
TCAAATTTGAGATAAATCATTCAAGTTTCTGCCCTATCAGCTTTCGATGGTAGTGTATTGGACTACCATGGCAGTCACGGGTAACGGAG
AATTAGGGTTCGGTTCCGGAGAGGGAGCCTGAGAAACGGCTACCACATCTAAGGAAGGCAGCAGGCGCGTAAATTACCCAATCCTGATT
CAGGGAGGTAGTGACAAGAAATAACAACTCGGGGATTTTTAATCTTACGGGATTGCAATGAGAACAATTTAAACCACTTAGCGAGGAAC
AATTGGAGGGCAAGTCTGGTGCCAGCAGCCGCGGTAATTCCAGCTCCAATAGCGTATATTAAAGTTGTTGCAGTTAAAAAGCTCGTAGT
TGAATTTCTGGCTTAGCTTAAACGCGGCTTCGGTCAGTTTAAGATAAGTCATCCGTCTGCAAACCATATTCGTCCTTCACGGGTTCGAT
TTGGGGAGTAGATTTTTTACTTTGAAAAAATTAGAGTGTTTCAGGCAGGTTATCGCCCGAATACATTAGCATGGAATAATGGAATAGGA
CTTTTGTCCATTTGGTTGGTTATTGGACATAAGTAATGATTAATAGGGACAGTTGGGGGCATTAGTATTTAATTGTCAGAGGTGAAATT
CTTGGATTTATTAAAGACTAACTTATGCGAAAGCATTTGCCAAGGATGTTTTCATTAATCAAGAACGAAAGTTAAGGGATCAAAGACGA
TCAGATACCGTAGTAGTCTTAACTATAAACTATACCGACTCGGGATTGGATGGGTATATGCCCATTCAGCACCGTATGAGAAATCAAAG
TCTTTGGGTTCTGGGGGGAGTATGGTCGCAAGGCTGAAACTTAAAGGAATTGACGGAAGGGCACCACCAGGAGTGGAGCCTGCGGCTTA
ATTTGACTCAACACGGGGAAACTTACCAGGTCAAAACATGGGTGGGATTGACAGATTGAGAGCTCTTTCTTGATTCTATGGGTGGTGGT
GCATGGCCGTTCTTAGTTGGTGGAGTGATTTGTCTGGTTAATTCCGTTAACGAACGAGACCTTAACCTGCTAACTAGTCTTTTAGTATA
ATTACTAAAAGTACTTCTTAGAGGGACTATGCGACGAAACGCATGGAAGTTTGAGGCAATAACAGGTCTGTGATGCCCTTAGATGTCCT
GGGCCGCACGTGCGCTACAATGACACGTTCAACAAGTTTTTTCCTGGCCCGGAAGGGTACGGGTAATCTTTTTAATGCGTGTCGTGTTA
GGGATAGATCTTTGGAATTATAGATCTTGAACGAGGAATTCCTAGTAAGTGCAAGTCATCAACTTGTACTGATTACGTCCCTGCCCTTT
GTACACACCGCCCGTCGCTCCTACCGATTTCGAGTGATCCGGTGAACCTTCTGGACTAGGTCGTCCTTGAGACGGACTTGGGAAGTTAA
GTAAACCTTATCACTTAGAGGAAGGAGAAGTCGTAACAAGGTTTCCGTAGGTGAACCTGCAGAAGGATCA
>Haptophrya_planariarum_KDo_33_DG
AACCTGGTTGATCCTGCCAGTAGTCATATGCTTGTCTCAAAGATTAAGCCATGCATGTCTAAGTATAATAGTATACAGTAAAACTGCGA
ATGGCTCATTACAACAGTTATAGTTTATTTGATAATGGAAGTCTACATGGATAACCGTGGTAATTCTAGAGCTAATACATGCTGTTAAA
CCTAACTTTACGGAAGGGTTGTATTTATTAGATATTAAGCCAATATTCTTTCGGGATTATTGCGAGGAATCATAATAACTGATCGAATC
TCAAATTTGAGATAAATCATTCAAGTTTCTGCCCTATCAGCTTTCGATGGTAGTGTATTGGACTACCATGGCAGTCACGGGTAACGGAG
AATTAGGGTTCGGTTCCGGAGAGGGAGCCTGAGAAACGGCTACCACATCTAAGGAAGGCAGCAGGCGCGTAAATTACCCAATCCTGATT
CAGGGAGGTAGTGACAAGAAATAACAACTCGGGGATTTTTAATCTTACGGGATTGCAATGAGAACAATTTAAACCACTTAGCGAGGAAC
AATTGGAGGGCAAGTCTGGTGCCAGCAGCCGCGGTAATTCCAGCTCCAATAGCGTATATTAAAGTTGTTGCAGTTAAAAAGCTCGTAGT
TGAATTTCTGGCTTAGCTTAAACGCGGCTTCGGTCAGTTTAAGATAAGTCATCCGTCTGCAAACCATATTCGTCCTTCACGGGTTCGAT
TTGGGGAGTAGATTTTTTACTTTGAAAAAATTAGAGTGTTTCAGGCAGGTTATCGCCCGAATACATTAGCATGGAATAATGGAATAGGA
CTTTTGTCCATTTGGTTGGTTATTGGACATAAGTAATGATTAATAGGGACAGTTGGGGGCATTAGTATTTAATTGTCAGAGGTGAAATT
CTTGGATTTATTAAAGACTAACTTATGCGAAAGCATTTGCCAAGGATGTTTTCATTAATCAAGAACGAAAGTTAAGGGATCAAAGACGA
TCAGATACCGTAGTAGTCTTAACTATAAACTATACCGACTCGGGATTGGATGGGTATATGCCCATTCAGCACCGTATGAGAAATCAAAG
TCTTTGGGTTCTGGGGGGAGTATGGTCGCAAGGCTGAAACTTAAAGGAATTGACGGAAGGGCACCACCAGGAGTGGAGCCTGCGGCTTA
ATTTGACTCAACACGGGGAAACTTACCAGGTCAAAACATGGGTGGGATTGACAGATTGAGAGCTCTTTCTTGATTCTATGGGTGGTGGT
GCATGGCCGTTCTTAGTTGGTGGAGTGATTTGTCTGGTTAATTCCGTTAACGAACGAGACCTTAACCTGCTAACTAGTCTTTTAGTATA
ATTACTAAAAGTACTTCTTAGAGGGACTATGCGACGAAACGCATGGAAGTTTGAGGCAATAACAGGTCTGTGATGCCCTTAGATGTCCT
GGGCCGCACGTGCGCTACAATGACACGTTCAACAAGTTTTTTCCTGGCCCGGAAGGGTACGGGTAATCTTTTTAATGCGTGTCGTGTTA
GGGATAGATCTTTGGAATTATAGATCTTGAACGAGGAATTCCTAGTAAGTGCAAGTCATCAACTTGTACTGATTACGTCCCTGCCCTTT
GTACACACCGCCCGTCGCTCCTACCGATTTCGAGTGATCCGGTGAACCTTCTGGACTAGGTCGTCCTTGAGACGGACTTGGGAAGTTAA
GTAAACCTTATCACTTAGAGGAAGGAGAAGTCGTAACAAGGTTTCCGTAGGTGAACCTGCAGAAGGATCA
>Haptophrya_planariarum_MH_34_DG
AACCTGGTTGATCCTGCCAGTAGTCATATGCTTGTCTCAAAGATTAAGCCATGCATGTCTAAGTATAATAGTATACAGTAAAACTGCGA
ATGGCTCATTACAACAGTTATAGTTTATTTGATAATGGAAGTCTACATGGATAACCGTGGTAATTCTAGAGCTAATACATGCTGTTAAA
CCTAACTTTACGGAAGGGTTGTATTTATTAGATATTAAGCCAATATTCTTTCGGGATTATTGCGAGGAATCATAATAACTGATCGAATC
TCAAATTTGAGATAAATCATTCAAGTTTCTGCCCTATCAGCTTTCGATGGTAGTGTATTGGACTACCATGGCAGTCACGGGTAACGGAG
AATTAGGGTTCGGTTCCGGAGAGGGAGCCTGAGAAACGGCTACCACATCTAAGGAAGGCAGCAGGCGCGTAAATTACCCAATCCTGATT
CAGGGAGGTAGTGACAAGAAATAACAACTCGGGGATTTTTAATCTTACGGGATTGCAATGAGAACAATTTAAACCACTTAGCGAGGAAC
AATTGGAGGGCAAGTCTGGTGCCAGCAGCCGCGGTAATTCCAGCTCCAATAGCGTATATTAAAGTTGTTGCAGTTAAAAAGCTCGTAGT
TGAATTTCTGGCTTAGCTTAAACGCGGCTTCGGTCAGTTTAAGATAAGTCATCCGTCTGCAAACCATATTCGTCCTTCACGGGTTCGAT
TTGGGGAGTAGATTTTTTACTTTGAAAAAATTAGAGTGTTTCAGGCAGGTTATCGCCCGAATACATTAGCATGGAATAATGGAATAGGA
CTTTTGTCCATTTGGTTGGTTATTGGACATAAGTAATGATTAATAGGGACAGTTGGGGGCATTAGTATTTAATTGTCAGAGGTGAAATT

CTTGGATTTATTAAAGACTAACTTATGCGAAAGCATTTGCCAAGGATGTTTTCATTAATCAAGAACGAAAGTTAAGGGATCAAAGACGA
TCAGATACCGTAGTAGTCTTAACTATAAACTATACCGACTCGGGATTGGATGGGTATATGCCCATTCAGCACCGTATGAGAAATCAAAG
TCTTTGGGTTCTGGGGGGAGTATGGTCGCAAGGCTGAAACTTAAAGGAATTGACGGAAGGGCACCACCAGGAGTGGAGCCTGCGGCTTA
ATTTGACTCAACACGGGGAAACTTACCAGGTCAAAACATGGGTGGGATTGACAGATTGAGAGCTCTTTCTTGATTCTATGGGTGGTGGT
GCATGGCCGTTCTTAGTTGGTGGAGTGATTTGTCTGGTTAATTCCGTTAACGAACGAGACCTTAACCTGCTAACTAGTCTTTTAGTATA
ATTACTAAAAGTACTTCTTAGAGGGACTATGCGACGAAACGCATGGAAGTTTGAGGCAATAACAGGTCTGTGATGCCCTTAGATGTCCT
GGGCCGCACGTGCGCTACAATGACACGTTCAACAAGTTTTTTCCTGGCCCGGAAGGGTACGGGTAATCTTTTTAATGCGTGTCGTGTTA
GGGATAGATCTTTGGAATTATAGATCTTGAACGAGGAATTCCTAGTAAGTGCAAGTCATCAACTTGTACTGATTACGTCCCTGCCCTTT
GTACACACCGCCCGTCGCTCCTACCGATTTCGAGTGATCCGGTGAACCTTCTGGACTAGGTCGTCCTTGAGACGGACTTGGGAAGTTAA
GTAAACCTTATCACTTAGAGGAAGGAGAAGTCGTAACAAGGTTTCCGTAGGTGAACCTGCAGAAGGATCA
>Haptophrya_planariarum_MH_35_DG
AACCTGGTTGATCCTGCCAGTAGTCATATGCTTGTCTCAAAGATTAAGCCATGCATGTCTAAGTATAATAGTATACAGTAAAACTGCGA
ATGGCTCATTACAACAGTTATAGTTTATTTGATAATGGAAGTCTACATGGATAACCGTGGTAATTCTAGAGCTAATACATGCTGTTAAA
CCTAACTTTACGGAAGGGTTGTATTTATTAGATATTAAGCCAATATTCTTTCGGGATTATTGCGAGGAATCATAATAACTGATCGAATC
TCAAATTTGAGATAAATCATTCAAGTTTCTGCCCTATCAGCTTTCGATGGTAGTGTATTGGACTACCATGGCAGTCACGGGTAACGGAG
AATTAGGGTTCGGTTCCGGAGAGGGAGCCTGAGAAACGGCTACCACATCTAAGGAAGGCAGCAGGCGCGTAAATTACCCAATCCTGATT
CAGGGAGGTAGTGACAAGAAATAACAACTCGGGGATTTTTAATCTTACGGGATTGCAATGAGAACAATTTAAACCACTTAGCGAGGAAC
AATTGGAGGGCAAGTCTGGTGCCAGCAGCCGCGGTAATTCCAGCTCCAATAGCGTATATTAAAGTTGTTGCAGTTAAAAAGCTCGTAGT
TGAATTTCTGGCTTAGCTTAAACGCGGCTTCGGTCAGTTTAAGATAAGTCATCCGTCTGCAAACCATATTCGTCCTTCACGGGTTCGAT
TTGGGGAGTAGATTTTTTACTTTGAAAAAATTAGAGTGTTTCAGGCAGGTTATCGCCCGAATACATTAGCATGGAATAATGGAATAGGA
CTTTTGTCCATTTGGTTGGTTATTGGACATAAGTAATGATTAATAGGGACAGTTGGGGGCATTAGTATTTAATTGTCAGAGGTGAAATT
CTTGGATTTATTAAAGACTAACTTATGCGAAAGCATTTGCCAAGGATGTTTTCATTAATCAAGAACGAAAGTTAAGGGATCAAAGACGA
TCAGATACCGTAGTAGTCTTAACTATAAACTATACCGACTCGGGATTGGATGGGTATATGCCCATTCAGCACCGTATGAGAAATCAAAG
TCTTTGGGTTCTGGGGGGAGTATGGTCGCAAGGCTGAAACTTAAAGGAATTGACGGAAGGGCACCACCAGGAGTGGAGCCTGCGGCTTA
ATTTGACTCAACACGGGGAAACTTACCAGGTCAAAACATGGGTGGGATTGACAGATTGAGAGCTCTTTCTTGATTCTATGGGTGGTGGT
GCATGGCCGTTCTTAGTTGGTGGAGTGATTTGTCTGGTTAATTCCGTTAACGAACGAGACCTTAACCTGCTAACTAGTCTTTTAGTATA
ATTACTAAAAGTACTTCTTAGAGGGACTATGCGACGAAACGCATGGAAGTTTGAGGCAATAACAGGTCTGTGATGCCCTTAGATGTCCT
GGGCCGCACGTGCGCTACAATGACACGTTCAACAAGTTTTTTCCTGGCCCGGAAGGGTACGGGTAATCTTTTTAATGCGTGTCGTGTTA
GGGATAGATCTTTGGAATTATAGATCTTGAACGAGGAATTCCTAGTAAGTGCAAGTCATCAACTTGTACTGATTACGTCCCTGCCCTTT
GTACACACCGCCCGTCGCTCCTACCGATTTCGAGTGATCCGGTGAACCTTCTGGACTAGGTCGTCCTTGAGACGGACTTGGGAAGTTAA
GTAAACCTTATCACTTAGAGGAAGGAGAAGTCGTAACAAGGTTTCCGTAGGTGAACCTGCAGAAGGATCA
>Haptophrya_planariarum_MH_36_DG
AACCTGGTTGATCCTGCCAGTAGTCATATGCTTGTCTCAAAGATTAAGCCATGCATGTCTAAGTATAATAGTATACAGTAAAACTGCGA
ATGGCTCATTACAACAGTTATAGTTTATTTGATAATGGAAGTCTACATGGATAACCGTGGTAATTCTAGAGCTAATACATGCTGTTAAA
CCTAACTTTACGGAAGGGTTGTATTTATTAGATATTAAGCCAATATTCTTTCGGGATTATTGCGAGGAATCATAATAACTGATCGAATC
TCAAATTTGAGATAAATCATTCAAGTTTCTGCCCTATCAGCTTTCGATGGTAGTGTATTGGACTACCATGGCAGTCACGGGTAACGGAG
AATTAGGGTTCGGTTCCGGAGAGGGAGCCTGAGAAACGGCTACCACATCTAAGGAAGGCAGCAGGCGCGTAAATTACCCAATCCTGATT
CAGGGAGGTAGTGACAAGAAATAACAACTCGGGGATTTTTAATCTTACGGGATTGCAATGAGAACAATTTAAACCACTTAGCGAGGAAC
AATTGGAGGGCAAGTCTGGTGCCAGCAGCCGCGGTAATTCCAGCTCCAATAGCGTATATTAAAGTTGTTGCAGTTAAAAAGCTCGTAGT
TGAATTTCTGGCTTAGCTTAAACGCGGCTTCGGTCAGTTTAAGATAAGTCATCCGTCTGCAAACCATATTCGTCCTTCACGGGTTCGAT
TTGGGGAGTAGATTTTTTACTTTGAAAAAATTAGAGTGTTTCAGGCAGGTTATCGCCCGAATACATTAGCATGGAATAATGGAATAGGA
CTTTTGTCCATTTGGTTGGTTATTGGACATAAGTAATGATTAATAGGGACAGTTGGGGGCATTAGTATTTAATTGTCAGAGGTGAAATT
CTTGGATTTATTAAAGACTAACTTATGCGAAAGCATTTGCCAAGGATGTTTTCATTAATCAAGAACGAAAGTTAAGGGATCAAAGACGA
TCAGATACCGTAGTAGTCTTAACTATAAACTATACCGACTCGGGATTGGATGGGTATATGCCCATTCAGCACCGTATGAGAAATCAAAG
TCTTTGGGTTCTGGGGGGAGTATGGTCGCAAGGCTGAAACTTAAAGGAATTGACGGAAGGGCACCACCAGGAGTGGAGCCTGCGGCTTA
ATTTGACTCAACACGGGGAAACTTACCAGGTCAAAACATGGGTGGGATTGACAGATTGAGAGCTCTTTCTTGATTCTATGGGTGGTGGT
GCATGGCCGTTCTTAGTTGGTGGAGTGATTTGTCTGGTTAATTCCGTTAACGAACGAGACCTTAACCTGCTAACTAGTCTTTTAGTATA
ATTACTAAAAGTACTTCTTAGAGGGACTATGCGACGAAACGCATGGAAGTTTGAGGCAATAACAGGTCTGTGATGCCCTTAGATGTCCT
GGGCCGCACGTGCGCTACAATGACACGTTCAACAAGTTTTTTCCTGGCCCGGAAGGGTACGGGTAATCTTTTTAATGCGTGTCGTGTTA
GGGATAGATCTTTGGAATTATAGATCTTGAACGAGGAATTCCTAGTAAGTGCAAGTCATCAACTTGTACTGATTACGTCCCTGCCCTTT
GTACACACCGCCCGTCGCTCCTACCGATTTCGAGTGATCCGGTGAACCTTCTGGACTAGGTCGTCCTTGAGACGGACTTGGGAAGTTAA
GTAAACCTTATCACTTAGAGGAAGGAGAAGTCGTAACAAGGTTTCCGTAGGTGAACCTGCAGAAGGATCA
>Haptophrya_planariarum_MH_37_DG
AACCTGGTTGATCCTGCCAGTAGTCATATGCTTGTCTCAAAGATTAAGCCATGCATGTCTAAGTATAATAGTATACAGTAAAACTGCGA
ATGGCTCATTACAACAGTTATAGTTTATTTGATAATGGAAGTCTACATGGATAACCGTGGTAATTCTAGAGCTAATACATGCTGTTAAA
CCTAACTTTACGGAAGGGTTGTATTTATTAGATATTAAGCCAATATTCTTTCGGGATTATTGCGAGGAATCATAATAACTGATCGAATC
TCAAATTTGAGATAAATCATTCAAGTTTCTGCCCTATCAGCTTTCGATGGTAGTGTATTGGACTACCATGGCAGTCACGGGTAACGGAG
AATTAGGGTTCGGTTCCGGAGAGGGAGCCTGAGAAACGGCTACCACATCTAAGGAAGGCAGCAGGCGCGTAAATTACCCAATCCTGATT
CAGGGAGGTAGTGACAAGAAATAACAACTCGGGGATTTTTAATCTTACGGGATTGCAATGAGAACAATTTAAACCACTTAGCGAGGAAC
AATTGGAGGGCAAGTCTGGTGCCAGCAGCCGCGGTAATTCCAGCTCCAATAGCGTATATTAAAGTTGTTGCAGTTAAAAAGCTCGTAGT
TGAATTTCTGGCTTAGCTTAAACGCGGCTTCGGTCAGTTTAAGATAAGTCATCCGTCTGCAAACCATATTCGTCCTTCACGGGTTCGAT
TTGGGGAGTAGATTTTTTACTTTGAAAAAATTAGAGTGTTTCAGGCAGGTTATCGCCCGAATACATTAGCATGGAATAATGGAATAGGA
CTTTTGTCCATTTGGTTGGTTATTGGACATAAGTAATGATTAATAGGGACAGTTGGGGGCATTAGTATTTAATTGTCAGAGGTGAAATT
CTTGGATTTATTAAAGACTAACTTATGCGAAAGCATTTGCCAAGGATGTTTTCATTAATCAAGAACGAAAGTTAAGGGATCAAAGACGA
TCAGATACCGTAGTAGTCTTAACTATAAACTATACCGACTCGGGATTGGATGGGTATATGCCCATTCAGCACCGTATGAGAAATCAAAG
TCTTTGGGTTCTGGGGGGAGTATGGTCGCAAGGCTGAAACTTAAAGGAATTGACGGAAGGGCACCACCAGGAGTGGAGCCTGCGGCTTA
ATTTGACTCAACACGGGGAAACTTACCAGGTCAAAACATGGGTGGGATTGACAGATTGAGAGCTCTTTCTTGATTCTATGGGTGGTGGT
GCATGGCCGTTCTTAGTTGGTGGAGTGATTTGTCTGGTTAATTCCGTTAACGAACGAGACCTTAACCTGCTAACTAGTCTTTTAGTATA
ATTACTAAAAGTACTTCTTAGAGGGACTATGCGACGAAACGCATGGAAGTTTGAGGCAATAACAGGTCTGTGATGCCCTTAGATGTCCT
GGGCCGCACGTGCGCTACAATGACACGTTCAACAAGTTTTTTCCTGGCCCGGAAGGGTACGGGTAATCTTTTTAATGCGTGTCGTGTTA
GGGATAGATCTTTGGAATTATAGATCTTGAACGAGGAATTCCTAGTAAGTGCAAGTCATCAACTTGTACTGATTACGTCCCTGCCCTTT

GTACACACCGCCCGTCGCTCCTACCGATTTCGAGTGATCCGGTGAACCTTCTGGACTAGGTCGTCCTTGAGACGGACTTGGGAAGTTAA
GTAAACCTTATCACTTAGAGGAAGGAGAAGTCGTAACAAGGTTTCCGTAGGTGAACCTGCAGAAGGATCA
>Haptophrya_planariarum_RT_42_DG
AACCTGGTTGATCCTGCCAGTAGTCATATGCTTGTCTCAAAGATTAAGCCATGCATGTCTAAGTATAATAGTATACAGTAAAACTGCGA
ATGGCTCATTACAACAGTTATAGTTTATTTGATAATGGAAGTCTACATGGATAACCGTGGTAATTCTAGAGCTAATACATGCTGTTAAA
CCTAACTTTACGGAAGGGTTGTATTTATTAGATATTAAGCCAATATTCTTTCGGGATTATTGCGAGGAATCATAATAACTGATCGAATC
TCAAATTTGAGATAAATCATTCAAGTTTCTGCCCTATCAGCTTTCGATGGTAGTGTATTGGACTACCATGGCAGTCACGGGTAACGGAG
AATTAGGGTTCGGTTCCGGAGAGGGAGCCTGAGAAACGGCTACCACATCTAAGGAAGGCAGCAGGCGCGTAAATTACCCAATCCTGATT
CAGGGAGGTAGTGACAAGAAATAACAACTCGGGGATTTTTAATCTTACGGGATTGCAATGAGAACAATTTAAACCACTTAGCGAGGAAC
AATTGGAGGGCAAGTCTGGTGCCAGCAGCCGCGGTAATTCCAGCTCCAATAGCGTATATTAAAGTTGTTGCAGTTAAAAAGCTCGTAGT
TGAATTTCTGGCTTAGCTTAAACGCGGCTTCGGTCAGTTTAAGATAAGTCATCCGTCTGCAAACCATATTCGTCCTTCACGGGTTCGAT
TTGGGGAGTAGATTTTTTACTTTGAAAAAATTAGAGTGTTTCAGGCAGGTTATCGCCCGAATACATTAGCATGGAATAATGGAATAGGA
CTTTTGTCCATTTGGTTGGTTATTGGACATAAGTAATGATTAATAGGGACAGTTGGGGGCATTAGTATTTAATTGTCAGAGGTGAAATT
CTTGGATTTATTAAAGACTAACTTATGCGAAAGCATTTGCCAAGGATGTTTTCATTAATCAAGAACGAAAGTTAAGGGATCAAAGACGA
TCAGATACCGTAGTAGTCTTAACTATAAACTATACCGACTCGGGATTGGATGGGTATATGCCCATTCAGCACCGTATGAGAAATCAAAG
TCTTTGGGTTCTGGGGGGAGTATGGTCGCAAGGCTGAAACTTAAAGGAATTGACGGAAGGGCACCACCAGGAGTGGAGCCTGCGGCTTA
ATTTGACTCAACACGGGGAAACTTACCAGGTCAAAACATGGGTGGGATTGACAGATTGAGAGCTCTTTCTTGATTCTATGGGTGGTGGT
GCATGGCCGTTCTTAGTTGGTGGAGTGATTTGTCTGGTTAATTCCGTTAACGAACGAGACCTTAACCTGCTAACTAGTCTTTTAGTATA
ATTACTAAAAGTACTTCTTAGAGGGACTATGCGACGAAACGCATGGAAGTTTGAGGCAATAACAGGTCTGTGATGCCCTTAGATGTCCT
GGGCCGCACGTGCGCTACAATGACACGTTCAACAAGTTTTTTCCTGGCCCGGAAGGGTACGGGTAATCTTTTTAATGCGTGTCGTGTTA
GGGATAGATCTTTGGAATTATAGATCTTGAACGAGGAATTCCTAGTAAGTGCAAGTCATCAACTTGTACTGATTACGTCCCTGCCCTTT
GTACACACCGCCCGTCGCTCCTACCGATTTCGAGTGATCCGGTGAACCTTCTGGACTAGGTCGTCCTTGAGACGGACTTGGGAAGTTAA
GTAAACCTTATCACTTAGAGGAAGGAGAAGTCGTAACAAGGTTTCCGTAGGTGAACCTGCAGAAGGATCA
>Haptophrya_planariarum_RT_43_DG
AACCTGGTTGATCCTGCCAGTAGTCATATGCTTGTCTCAAAGATTAAGCCATGCATGTCTAAGTATAATAGTATACAGTAAAACTGCGA
ATGGCTCATTACAACAGTTATAGTTTATTTGATAATGGAAGTCTACATGGATAACCGTGGTAATTCTAGAGCTAATACATGCTGTTAAA
CCTAACTTTACGGAAGGGTTGTATTTATTAGATATTAAGCCAATATTCTTTCGGGATTATTGCGAGGAATCATAATAACTGATCGAATC
TCAAATTTGAGATAAATCATTCAAGTTTCTGCCCTATCAGCTTTCGATGGTAGTGTATTGGACTACCATGGCAGTCACGGGTAACGGAG
AATTAGGGTTCGGTTCCGGAGAGGGAGCCTGAGAAACGGCTACCACATCTAAGGAAGGCAGCAGGCGCGTAAATTACCCAATCCTGATT
CAGGGAGGTAGTGACAAGAAATAACAACTCGGGGATTTTTAATCTTACGGGATTGCAATGAGAACAATTTAAACCACTTAGCGAGGAAC
AATTGGAGGGCAAGTCTGGTGCCAGCAGCCGCGGTAATTCCAGCTCCAATAGCGTATATTAAAGTTGTTGCAGTTAAAAAGCTCGTAGT
TGAATTTCTGGCTTAGCTTAAACGCGGCTTCGGTCAGTTTAAGATAAGTCATCCGTCTGCAAACCATATTCGTCCTTCACGGGTTCGAT
TTGGGGAGTAGATTTTTTACTTTGAAAAAATTAGAGTGTTTCAGGCAGGTTATCGCCCGAATACATTAGCATGGAATAATGGAATAGGA
CTTTTGTCCATTTGGTTGGTTATTGGACATAAGTAATGATTAATAGGGACAGTTGGGGGCATTAGTATTTAATTGTCAGAGGTGAAATT
CTTGGATTTATTAAAGACTAACTTATGCGAAAGCATTTGCCAAGGATGTTTTCATTAATCAAGAACGAAAGTTAAGGGATCAAAGACGA
TCAGATACCGTAGTAGTCTTAACTATAAACTATACCGACTCGGGATTGGATGGGTATATGCCCATTCAGCACCGTATGAGAAATCAAAG
TCTTTGGGTTCTGGGGGGAGTATGGTCGCAAGGCTGAAACTTAAAGGAATTGACGGAAGGGCACCACCAGGAGTGGAGCCTGCGGCTTA
ATTTGACTCAACACGGGGAAACTTACCAGGTCAAAACATGGGTGGGATTGACAGATTGAGAGCTCTTTCTTGATTCTATGGGTGGTGGT
GCATGGCCGTTCTTAGTTGGTGGAGTGATTTGTCTGGTTAATTCCGTTAACGAACGAGACCTTAACCTGCTAACTAGTCTTTTAGTATA
ATTACTAAAAGTACTTCTTAGAGGGACTATGCGACGAAACGCATGGAAGTTTGAGGCAATAACAGGTCTGTGATGCCCTTAGATGTCCT
GGGCCGCACGTGCGCTACAATGACACGTTCAACAAGTTTTTTCCTGGCCCGGAAGGGTACGGGTAATCTTTTTAATGCGTGTCGTGTTA
GGGATAGATCTTTGGAATTATAGATCTTGAACGAGGAATTCCTAGTAAGTGCAAGTCATCAACTTGTACTGATTACGTCCCTGCCCTTT
GTACACACCGCCCGTCGCTCCTACCGATTTCGAGTGATCCGGTGAACCTTCTGGACTAGGTCGTCCTTGAGACGGACTTGGGAAGTTAA
GTAAACCTTATCACTTAGAGGAAGGAGAAGTCGTAACAAGGTTTCCGTAGGTGAACCTGCAGAAGGATCA
>Haptophrya_planariarum_RT_44_DG
AACCTGGTTGATCCTGCCAGTAGTCATATGCTTGTCTCAAAGATTAAGCCATGCATGTCTAAGTATAATAGTATACAGTAAAACTGCGA
ATGGCTCATTACAACAGTTATAGTTTATTTGATAATGGAAGTCTACATGGATAACCGTGGTAATTCTAGAGCTAATACATGCTGTTAAA
CCTAACTTTACGGAAGGGTTGTATTTATTAGATATTAAGCCAATATTCTTTCGGGATTATTGCGAGGAATCATAATAACTGATCGAATC
TCAAATTTGAGATAAATCATTCAAGTTTCTGCCCTATCAGCTTTCGATGGTAGTGTATTGGACTACCATGGCAGTCACGGGTAACGGAG
AATTAGGGTTCGGTTCCGGAGAGGGAGCCTGAGAAACGGCTACCACATCTAAGGAAGGCAGCAGGCGCGTAAATTACCCAATCCTGATT
CAGGGAGGTAGTGACAAGAAATAACAACTCGGGGATTTTTAATCTTACGGGATTGCAATGAGAACAATTTAAACCACTTAGCGAGGAAC
AATTGGAGGGCAAGTCTGGTGCCAGCAGCCGCGGTAATTCCAGCTCCAATAGCGTATATTAAAGTTGTTGCAGTTAAAAAGCTCGTAGT
TGAATTTCTGGCTTAGCTTAAACGCGGCTTCGGTCAGTTTAAGATAAGTCATCCGTCTGCAAACCATATTCGTCCTTCACGGGTTCGAT
TTGGGGAGTAGATTTTTTACTTTGAAAAAATTAGAGTGTTTCAGGCAGGTTATCGCCCGAATACATTAGCATGGAATAATGGAATAGGA
CTTTTGTCCATTTGGTTGGTTATTGGACATAAGTAATGATTAATAGGGACAGTTGGGGGCATTAGTATTTAATTGTCAGAGGTGAAATT
CTTGGATTTATTAAAGACTAACTTATGCGAAAGCATTTGCCAAGGATGTTTTCATTAATCAAGAACGAAAGTTAAGGGATCAAAGACGA
TCAGATACCGTAGTAGTCTTAACTATAAACTATACCGACTCGGGATTGGATGGGTATATGCCCATTCAGCACCGTATGAGAAATCAAAG
TCTTTGGGTTCTGGGGGGAGTATGGTCGCAAGGCTGAAACTTAAAGGAATTGACGGAAGGGCACCACCAGGAGTGGAGCCTGCGGCTTA
ATTTGACTCAACACGGGGAAACTTACCAGGTCAAAACATGGGTGGGATTGACAGATTGAGAGCTCTTTCTTGATTCTATGGGTGGTGGT
GCATGGCCGTTCTTAGTTGGTGGAGTGATTTGTCTGGTTAATTCCGTTAACGAACGAGACCTTAACCTGCTAACTAGTCTTTTAGTATA
ATTACTAAAAGTACTTCTTAGAGGGACTATGCGACGAAACGCATGGAAGTTTGAGGCAATAACAGGTCTGTGATGCCCTTAGATGTCCT
GGGCCGCACGTGCGCTACAATGACACGTTCAACAAGTTTTTTCCTGGCCCGGAAGGGTACGGGTAATCTTTTTAATGCGTGTCGTGTTA
GGGATAGATCTTTGGAATTATAGATCTTGAACGAGGAATTCCTAGTAAGTGCAAGTCATCAACTTGTACTGATTACGTCCCTGCCCTTT
GTACACACCGCCCGTCGCTCCTACCGATTTCGAGTGATCCGGTGAACCTTCTGGACTAGGTCGTCCTTGAGACGGACTTGGGAAGTTAA
GTAAACCTTATCACTTAGAGGAAGGAGAAGTCGTAACAAGGTTTCCGTAGGTGAACCTGCAGAAGGATCA
>Haptophrya_planariarum_RT_47_DG
AACCTGGTTGATCCTGCCAGTAGTCATATGCTTGTCTCAAAGATTAAGCCATGCATGTCTAAGTATAATAGTATACAGTAAAACTGCGA
ATGGCTCATTACAACAGTTATAGTTTATTTGATAATGGAAGTCTACATGGATAACCGTGGTAATTCTAGAGCTAATACATGCTGTTAAA
CCTAACTTTACGGAAGGGTTGTATTTATTAGATATTAAGCCAATATTCTTTCGGGATTATTGCGAGGAATCATAATAACTGATCGAATC
TCAAATTTGAGATAAATCATTCAAGTTTCTGCCCTATCAGCTTTCGATGGTAGTGTATTGGACTACCATGGCAGTCACGGGTAACGGAG
AATTAGGGTTCGGTTCCGGAGAGGGAGCCTGAGAAACGGCTACCACATCTAAGGAAGGCAGCAGGCGCGTAAATTACCCAATCCTGATT

CAGGGAGGTAGTGACAAGAAATAACAACTCGGGGATTTTTAATCTTACGGGATTGCAATGAGAACAATTTAAACCACTTAGCGAGGAAC
AATTGGAGGGCAAGTCTGGTGCCAGCAGCCGCGGTAATTCCAGCTCCAATAGCGTATATTAAAGTTGTTGCAGTTAAAAAGCTCGTAGT
TGAATTTCTGGCTTAGCTTAAACGCGGCTTCGGTCAGTTTAAGATAAGTCATCCGTCTGCAAACCATATTCGTCCTTCACGGGTTCGAT
TTGGGGAGTAGATTTTTTACTTTGAAAAAATTAGAGTGTTTCAGGCAGGTTATCGCCCGAATACATTAGCATGGAATAATGGAATAGGA
CTTTTGTCCATTTGGTTGGTTATTGGACATAAGTAATGATTAATAGGGACAGTTGGGGGCATTAGTATTTAATTGTCAGAGGTGAAATT
CTTGGATTTATTAAAGACTAACTTATGCGAAAGCATTTGCCAAGGATGTTTTCATTAATCAAGAACGAAAGTTAAGGGATCAAATACGA
TCAGATACCGTAGTAGTCTTAACTATAAACTATACCGACTCGGGATTGGATGGGTATATGCCCATTCAGCACCGTATGAGAAATCAAAG
TCTTTGGGTTCTGGGGGGAGTATGGTCGCAAGGCTGAAACTTAAAGGAATTGACGGAAGGGCACCACCAGGAGTGGAGCCTGCGGCTTA
ATTTGACTCAACACGGGGAAACTTACCAGGTCAAAACATGGGTGGGATTGACAGATTGAGAGCTCTTTCTTGATTCTATGGGTGGTGGT
GCATGGCCGTTCTTAGTTGGTGGAGTGATTTGTCTGGTTAATTCCGTTAACGAACGAGACCTTAACCTGCTAACTAGTCTTTTAGTATA
ATTACTAAAAGTACTTCTTAGAGGGACTATGCGACGAAACGCATGGAAGTTTGAGGCAATAACAGGTCTGTGATGCCCTTAGATGTCCT
GGGCCGCACGTGCGCTACAATGACACGTTCAACAAGTTTTTTCCTGGCCCGGAAGGGTACGGGTAATCTTTTTAATGCGTGTCGTGTTA
GGGATAGATCTTTGGAATTATAGATCTTGAACGAGGAATTCCTAGTAAGTGCAAGTCATCAACTTGTACTGATTACGTCCCTGCCCTTT
GTACACACCGCCCGTCGCTCCTACCGATTTCGAGTGATCCGGTGAACCTTCTGGACTAGGTCGTCCTTGAGACGGACTTGGGAAGTTAA
GTAAACCTTATCACTTAGAGGAAGGAGAAGTCGTAACAAGGTTTCCGTAGGTGAACCTGCAGAAGGATCA
>Haptophrya_planariarum_RT_48_DG
AACCTGGTTGATCCTGCCAGTAGTCATATGCTTGTCTCAAAGATTAAGCCATGCATGTCTAAGTATAATAGTATACAGTAAAACTGCGA
ATGGCTCATTACAACAGTTATAGTTTATTTGATAATGGAAGTCTACATGGATAACCGTGGTAATTCTAGAGCTAATACATGCTGTTAAA
CCTAACTTTACGGAAGGGTTGTATTTATTAGATATTAAGCCAATATTCTTTCGGGATTATTGCGAGGAATCATAATAACTGATCGAATC
TCAAATTTGAGATAAATCATTCAAGTTTCTGCCCTATCAGCTTTCGATGGTAGTGTATTGGACTACCATGGCAGTCACGGGTAACGGAG
AATTAGGGTTCGGTTCCGGAGAGGGAGCCTGAGAAACGGCTACCACATCTAAGGAAGGCAGCAGGCGCGTAAATTACCCAATCCTGATT
CAGGGAGGTAGTGACAAGAAATAACAACTCGGGGATTTTTAATCTTACGGGATTGCAATGAGAACAATTTAAACCACTTAGCGAGGAAC
AATTGGAGGGCAAGTCTGGTGCCAGCAGCCGCGGTAATTCCAGCTCCAATAGCGTATATTAAAGTTGTTGCAGTTAAAAAGCTCGTAGT
TGAATTTCTGGCTTAGCTTAAACGCGGCTTCGGTCAGTTTAAGATAAGTCATCCGTCTGCAAACCATATTCGTCCTTCACGGGTTCGAT
TTGGGGAGTAGATTTTTTACTTTGAAAAAATTAGAGTGTTTCAGGCAGGTTATCGCCCGAATACATTAGCATGGAATAATGGAATAGGA
CTTTTGTCCATTTGGTTGGTTATTGGACATAAGTAATGATTAATAGGGACAGTTGGGGGCATTAGTATTTAATTGTCAGAGGTGAAATT
CTTGGATTTATTAAAGACTAACTTATGCGAAAGCATTTGCCAAGGATGTTTTCATTAATCAAGAACGAAAGTTAAGGGATCAAAGACGA
TCAGATACCGTAGTAGTCTTAACTATAAACTATACCGACTCGGGATTGGATGGGTATATGCCCATTCAGCACCGTATGAGAAATCAAAG
TCTTTGGGTTCTGGGGGGAGTATGGTCGCAAGGCTGAAACTTAAAGGAATTGACGGAAGGGCACCACCAGGAGTGGAGCCTGCGGCTTA
ATTTGACTCAACACGGGGAAACTTACCAGGTCAAAACATGGGTGGGATTGACAGATTGAGAGCTCTTTCTTGATTCTATGGGTGGTGGT
GCATGGCCGTTCTTAGTTGGTGGAGTGATTTGTCTGGTTAATTCCGTTAACGAACGAGACCTTAACCTGCTAACTAGTCTTTTAGTATA
ATTACTAAAAGTACTTCTTAGAGGGACTATGCGACGAAACGCATGGAAGTTTGAGGCAATAACAGGTCTGTGATGCCCTTAGATGTCCT
GGGCCGCACGTGCGCTACAATGACACGTTCAACAAGTTTTTTCCTGGCCCGGAAGGGTACGGGTAATCTTTTTAATGCGTGTCGTGTTA
GGGATAGATCTTTGGAATTATAGATCTTGAACGAGGAATTCCTAGTAAGTGCAAGTCATCAACTTGTACTGATTACGTCCCTGCCCTTT
GTACACACCGCCCGTCGCTCCTACCGATTTCGAGTGATCCGGTGAACCTTCTGGACTAGGTCGTCCTTGAGACGGACTTGGGAAGTTAA
GTAAACCTTATCACTTAGAGGAAGGAGAAGTCGTAACAAGGTTTCCGTAGGTGAACCTGCAGAAGGATCA
>Haptophrya_planariarum_CA_50_DG
AACCTGGTTGATCCTGCCAGTAGTCATATGCTTGTCTCAAAGATTAAGCCATGCATGTCTAAGTATAATAGTATACAGTAAAACTGCGA
ATGGCTCATTACAACAGTTATAGTTTATTTGATAATGGAAGTCTACATGGATAACCGTGGTAATTCTAGAGCTAATACATGCTGTTAAA
CCTAACTTTACGGAAGGGTTGTATTTATTAGATATTAAGCCAATATTCTTTCGGGATTATTGCGAGGAATCATAATAACTGATCGAATC
TCAAATTTGAGATAAATCATTCAAGTTTCTGCCCTATCAGCTTTCGATGGTAGTGTATTGGACTACCATGGCAGTCACGGGTAACGGAG
AATTAGGGTTCGGTTCCGGAGAGGGAGCCTGAGAAACGGCTACCACATCTAAGGAAGGCAGCAGGCGCGTAAATTACCCAATCCTGATT
CAGGGAGGTAGTGACAAGAAATAACAACTCGGGGATTTTTAATCTTACGGGATTGCAATGAGAACAATTTAAACCACTTAGCGAGGAAC
AATTGGAGGGCAAGTCTGGTGCCAGCAGCCGCGGTAATTCCAGCTCCAATAGCGTATATTAAAGTTGTTGCAGTTAAAAAGCTCGTAGT
TGAATTTCTGGCTTAGCTTAAACGCGGCTTCGGTCAGTTTAAGATAAGTCATCCGTCTGCAAACCATATTCGTCCTTCACGGGTTCGAT
TTGGGGAGTAGATTTTTTACTTTGAAAAAATTAGAGTGTTTCAGGCAGGTTATCGCCCGAATACATTAGCATGGAATAATGGAATAGGA
CTTTTGTCCATTTGGTTGGTTATTGGACATAAGTAATGATTAATAGGGACAGTTGGGGGCATTAGTATTTAATTGTCAGAGGTGAAATT
CTTGGATTTATTAAAGACTAACTTATGCGAAAGCATTTGCCAAGGATGTTTTCATTAATCAAGAACGAAAGTTAAGGGATCAAAGACGA
TCAGATACCGTAGTAGTCTTAACTATAAACTATACCGACTCGGGATTGGATGGGTATATGCCCATTCAGCACCGTATGAGAAATCAAAG
TCTTTGGGTTCTGGGGGGAGTATGGTCGCAAGGCTGAAACTTAAAGGAATTGACGGAAGGGCACCACCAGGAGTGGAGCCTGCGGCTTA
ATTTGACTCAACACGGGGAAACTTACCAGGTCAAAACATGGGTGGGATTGACAGATTGAGAGCTCTTTCTTGATTCTATGGGTGGTGGT
GCATGGCCGTTCTTAGTTGGTGGAGTGATTTGTCTGGTTAATTCCGTTAACGAACGAGACCTTAACCTGCTAACTAGTCTTTTAGTATA
ATTACTAAAAGTACTTCTTAGAGGGACTATGCGACGAAACGCATGGAAGTTTGAGGCAATAACAGGTCTGTGATGCCCTTAGATGTCCT
GGGCCGCACGTGCGCTACAATGACACGTTCAACAAGTTTTTTCCTGGCCCGGAAGGGTACGGGTAATCTTTTTAATGCGTGTCGTGTTA
GGGATAGATCTTTGGAATTATAGATCTTGAACGAGGAATTCCTAGTAAGTGCAAGTCATCAACTTGTACTGATTACGTCCCTGCCCTTT
GTACACACCGCCCGTCGCTCCTACCGATTTCGAGTGATCCGGTGAACCTTCTGGACTAGGTCGTCCTTGAGACGGACTTGGGAAGTTAA
GTAAACCTTATCACTTAGAGGAAGGAGAAGTCGTAACAAGGTTTCCGTAGGTGAACCTGCAGAAGGATCA
>Haptophrya_planariarum_CA_51_DG
AACCTGGTTGATCCTGCCAGTAGTCATATGCTTGTCTCAAAGATTAAGCCATGCATGTCTAAGTATAATAGTATACAGTAAAACTGCGA
ATGGCTCATTACAACAGTTATAGTTTATTTGATAATGGAAGTCTACATGGATAACCGTGGTAATTCTAGAGCTAATACATGCTGTTAAA
CCTAACTTTACGGAAGGGTTGTATTTATTAGATATTAAGCCAATATTCTTTCGGGATTATTGCGAGGAATCATAATAACTGATCGAATC
TCAAATTTGAGATAAATCATTCAAGTTTCTGCCCTATCAGCTTTCGATGGTAGTGTATTGGACTACCATGGCAGTCACGGGTAACGGAG
AATTAGGGTTCGGTTCCGGAGAGGGAGCCTGAGAAACGGCTACCACATCTAAGGAAGGCAGCAGGCGCGTAAATTACCCAATCCTGATT
CAGGGAGGTAGTGACAAGAAATAACAACTCGGGGATTTTTAATCTTACGGGATTGCAATGAGAACAATTTAAACCACTTAGCGAGGAAC
AATTGGAGGGCAAGTCTGGTGCCAGCAGCCGCGGTAATTCCAGCTCCAATAGCGTATATTAAAGTTGTTGCAGTTAAAAAGCTCGTAGT
TGAATTTCTGGCTTAGCTTAAACGCGGCTTCGGTCAGTTTAAGATAAGTCATCCGTCTGCAAACCATATTCGTCCTTCACGGGTTCGAT
TTGGGGAGTAGATTTTTTACTTTGAAAAAATTAGAGTGTTTCAGGCAGGTTATCGCCCGAATACATTAGCATGGAATAATGGAATAGGA
CTTTTGTCCATTTGGTTGGTTATTGGACATAAGTAATGATTAATAGGGACAGTTGGGGGCATTAGTATTTAATTGTCAGAGGTGAAATT
CTTGGATTTATTAAAGACTAACTTATGCGAAAGCATTTGCCAAGGATGTTTTCATTAATCAAGAACGAAAGTTAAGGGATCAAAGACGA
TCAGATACCGTAGTAGTCTTAACTATAAACTATACCGACTCGGGATTGGATGGGTATATGCCCATTCAGCACCGTATGAGAAATCAAAG
TCTTTGGGTTCTGGGGGGAGTATGGTCGCAAGGCTGAAACTTAAAGGAATTGACGGAAGGGCACCACCAGGAGTGGAGCCTGCGGCTTA

ATTTGACTCAACACGGGGAAACTTACCAGGTCAAAACATGGGTGGGATTGACAGATTGAGAGCTCTTTCTTGATTCTATGGGTGGTGGT
GCATGGCCGTTCTTAGTTGGTGGAGTGATTTGTCTGGTTAATTCCGTTAACGAACGAGACCTTAACCTGCTAACTAGTCTTTTAGTATA
ATTACTAAAAGTACTTCTTAGAGGGACTATGCGACGAAACGCATGGAAGTTTGAGGCAATAACAGGTCTGTGATGCCCTTAGATGTCCT
GGGCCGCACGTGCGCTACAATGACACGTTCAACAAGTTTTTTCCTGGCCCGGAAGGGTACGGGTAATCTTTTTAATGCGTGTCGTGTTA
GGGATAGATCTTTGGAATTATAGATCTTGAACGAGGAATTCCTAGTAAGTGCAAGTCATCAACTTGTACTGATTACGTCCCTGCCCTTT
GTACACACCGCCCGTCGCTCCTACCGATTTCGAGTGATCCGGTGAACCTTCTGGACTAGGTCGTCCTTGAGACGGACTTGGGAAGTTAA
GTAAACCTTATCACTTAGAGGAAGGAGAAGTCGTAACAAGGTTTCCGTAGGTGAACCTGCAGAAGGATCA
>Haptophrya_planariarum_CA_52_DG
AACCTGGTTGATCCTGCCAGTAGTCATATGCTTGTCTCAAAGATTAAGCCATGCATGTCTAAGTATAATAGTATACAGTAAAACTGCGA
ATGGCTCATTACAACAGTTATAGTTTATTTGATAATGGAAGTCTACATGGATAACCGTGGTAATTCTAGAGCTAATACATGCTGTTAAA
CCTAACTTTACGGAAGGGTTGTATTTATTAGATATTAAGCCAATATTCTTTCGGGATTATTGCGAGGAATCATAATAACTGATCGAATC
TCAAATTTGAGATAAATCATTCAAGTTTCTGCCCTATCAGCTTTCGATGGTAGTGTATTGGACTACCATGGCAGTCACGGGTAACGGAG
AATTAGGGTTCGGTTCCGGAGAGGGAGCCTGAGAAACGGCTACCACATCTAAGGAAGGCAGCAGGCGCGTAAATTACCCAATCCTGATT
CAGGGAGGTAGTGACAAGAAATAACAACTCGGGGATTTTTAATCTTACGGGATTGCAATGAGAACAATTTAAACCACTTAGCGAGGAAC
AATTGGAGGGCAAGTCTGGTGCCAGCAGCCGCGGTAATTCCAGCTCCAATAGCGTATATTAAAGTTGTTGCAGTTAAAAAGCTCGTAGT
TGAATTTCTGGCTTAGCTTAAACGCGGCTTCGGTCAGTTTAAGATAAGTCATCCGTCTGCAAACCATATTCGTCCTTCACGGGTTCGAT
TTGGGGAGTAGATTTTTTACTTTGAAAAAATTAGAGTGTTTCAGGCAGGTTATCGCCCGAATACATTAGCATGGAATAATGGAATAGGA
CTTTTGTCCATTTGGTTGGTTATTGGACATAAGTAATGATTAATAGGGACAGTTGGGGGCATTAGTATTTAATTGTCAGAGGTGAAATT
CTTGGATTTATTAAAGACTAACTTATGCGAAAGCATTTGCCAAGGATGTTTTCATTAATCAAGAACGAAAGTTAAGGGATCAAAGACGA
TCAGATACCGTAGTAGTCTTAACTATAAACTATACCGACTCGGGATTGGATGGGTATATGCCCATTCAGCACCGTATGAGAAATCAAAG
TCTTTGGGTTCTGGGGGGAGTATGGTCGCAAGGCTGAAACTTAAAGGAATTGACGGAAGGGCACCACCAGGAGTGGAGCCTGCGGCTTA
ATTTGACTCAACACGGGGAAACTTACCAGGTCAAAACATGGGTGGGATTGACAGATTGAGAGCTCTTTCTTGATTCTATGGGTGGTGGT
GCATGGCCGTTCTTAGTTGGTGGAGTGATTTGTCTGGTTAATTCCGTTAACGAACGAGACCTTAACCTGCTAACTAGTCTTTTAGTATA
ATTACTAAAAGTACTTCTTAGAGGGACTATGCGACGAAACGCATGGAAGTTTGAGGCAATAACAGGTCTGTGATGCCCTTAGATGTCCT
GGGCCGCACGTGCGCTACAATGACACGTTCAACAAGTTTTTTCCTGGCCCGGAAGGGTACGGGTAATCTTTTTAATGCGTGTCGTGTTA
GGGATAGATCTTTGGAATTATAGATCTTGAACGAGGAATTCCTAGTAAGTGCAAGTCATCAACTTGTACTGATTACGTCCCTGCCCTTT
GTACACACCGCCCGTCGCTCCTACCGATTTCGAGTGATCCGGTGAACCTTCTGGACTAGGTCGTCCTTGAGACGGACTTGGGAAGTTAA
GTAAACCTTATCACTTAGAGGAAGGAGAAGTCGTAACAAGGTTTCCGTAGGTGAACCTGCAGAAGGATCA
>Haptophrya_planariarum_DL_54_DG
AACCTGGTTGATCCTGCCAGTAGTCATATGCTTGTCTCAAAGATTAAGCCATGCATGTCTAAGTATAATAGTATACAGTAAAACTGCGA
ATGGCTCATTACAACAGTTATAGTTTATTTGATAATGGAAGTCTACATGGATAACCGTGGTAATTCTAGAGCTAATACATGCTGTTAAA
CCTAACTTTACGGAAGGGTTGTATTTATTAGATATTAAGCCAATATTCTTTCGGGATTATTGCGAGGAATCATAATAACTGATCGAATC
TCAAATTTGAGATAAATCATTCAAGTTTCTGCCCTATCAGCTTTCGATGGTAGTGTATTGGACTACCATGGCAGTCACGGGTAACGGAG
AATTAGGGTTCGGTTCCGGAGAGGGAGCCTGAGAAACGGCTACCACATCTAAGGAAGGCAGCAGGCGCGTAAATTACCCAATCCTGATT
CAGGGAGGTAGTGACAAGAAATAACAACTCGGGGATTTTTAATCTTACGGGATTGCAATGAGAACAATTTAAACCACTTAGCGAGGAAC
AATTGGAGGGCAAGTCTGGTGCCAGCAGCCGCGGTAATTCCAGCTCCAATAGCGTATATTAAAGTTGTTGCAGTTAAAAAGCTCGTAGT
TGAATTTCTGGCTTAGCTTAAACGCGGCTTCGGTCAGTTTAAGATAAGTCATCCGTCTGCAAACCATATTCGTCCTTCACGGGTTCGAT
TTGGGGAGTAGATTTTTTACTTTGAAAAAATTAGAGTGTTTCAGGCAGGTTATCGCCCGAATACATTAGCATGGAATAATGGAATAGGA
CTTTTGTCCATTTGGTTGGTTATTGGACATAAGTAATGATTAATAGGGACAGTTGGGGGCATTAGTATTTAATTGTCAGAGGTGAAATT
CTTGGATTTATTAAAGACTAACTTATGCGAAAGCATTTGCCAAGGATGTTTTCATTAATCAAGAACGAAAGTTAAGGGATCAAAGACGA
TCAGATACCGTAGTAGTCTTAACTATAAACTATACCGACTCGGGATTGGATGGGTATATGCCCATTCAGCACCGTATGAGAAATCAAAG
TCTTTGGGTTCTGGGGGGAGTATGGTCGCAAGGCTGAAACTTAAAGGAATTGACGGAAGGGCACCACCAGGAGTGGAGCCTGCGGCTTA
ATTTGACTCAACACGGGGAAACTTACCAGGTCAAAACATGGGTGGGATTGACAGATTGAGAGCTCTTTCTTGATTCTATGGGTGGTGGT
GCATGGCCGTTCTTAGTTGGTGGAGTGATTTGTCTGGTTAATTCCGTTAACGAACGAGACCTTAACCTGCTAACTAGTCTTTTAGTATA
ATTACTAAAAGTACTTCTTAGAGGGACTATGCGACGAAACGCATGGAAGTTTGAGGCAATAACAGGTCTGTGATGCCCTTAGATGTCCT
GGGCCGCACGTGCGCTACAATGACACGTTCAACAAGTTTTTTCCTGGCCCGGAAGGGTACGGGTAATCTTTTTAATGCGTGTCGTGTTA
GGGATAGATCTTTGGAATTATAGATCTTGAACGAGGAATTCCTAGTAAGTGCAAGTCATCAACTTGTACTGATTACGTCCCTGCCCTTT
GTACACACCGCCCGTCGCTCCTACCGATTTCGAGTGATCCGGTGAACCTTCTGGACTAGGTCGTCCTTGAGACGGACTTGGGAAGTTAA
GTAAACCTTATCACTTAGAGGAAGGAGAAGTCGTAACAAGGTTTCCGTAGGTGAACCTGCAGAAGGATCA
>Haptophrya_planariarum_BB_85_DG
AACCTGGTTGATCCTGCCAGTAGTCATATGCTTGTCTCAAAGATTAAGCCATGCATGTCTAAGTATAATAGTATACAGTAAAACTGCGA
ATGGCTCATTACAACAGTTATAGTTTATTTGATAATGGAAGTCTACATGGATAACCGTGGTAATTCTAGAGCTAATACATGCTGTTAAA
CCTAACTTTACGGAAGGGTTGTATTTATTAGATATTAAGCCAATATTCTTTCGGGATTATTGCGAGGAATCATAATAACTGATCGAATC
TCAAATTTGAGATAAATCATTCAAGTTTCTGCCCTATCAGCTTTCGATGGTAGTGTATTGGACTACCATGGCAGTCACGGGTAACGGAG
AATTAGGGTTCGGTTCCGGAGAGGGAGCCTGAGAAACGGCTACCACATCTAAGGAAGGCAGCAGGCGCGTAAATTACCCAATCCTGATT
CAGGGAGGTAGTGACAAGAAATAACAACTCGGGGATTTTTAATCTTACGGGATTGCAATGAGAACAATTTAAACCACTTAGCGAGGAAC
AATTGGAGGGCAAGTCTGGTGCCAGCAGCCGCGGTAATTCCAGCTCCAATAGCGTATATTAAAGTTGTTGCAGTTAAAAAGCTCGTAGT
TGAATTTCTGGCTTAGCTTAAACGCGGCTTCGGTCAGTTTAAGATAAGTCATCCGTCTGCAAACCATATTCGTCCTTCACGGGTTCGAT
TTGGGGAGTAGATTTTTTACTTTGAAAAAATTAGAGTGTTTCAGGCAGGTTATCGCCCGAATACATTAGCATGGAATAATGGAATAGGA
CTTTTGTCCATTTGGTTGGTTATTGGACATAAGTAATGATTAATAGGGACAGTTGGGGGCATTAGTATTTAATTGTCAGAGGTGAAATT
CTTGGATTTATTAAAGACTAACTTATGCGAAAGCATTTGCCAAGGATGTTTTCATTAATCAAGAACGAAAGTTAAGGGATCAAATACGA
TCAGATACCGTAGTAGTCTTAACTATAAACTATACCGACTCGGGATTGGATGGGTATATGCCCATTCAGCACCGTATGAGAAATCAAAG
TCTTTGGGTTCTGGGGGGAGTATGGTCGCAAGGCTGAAACTTAAAGGAATTGACGGAAGGGCACCACCAGGAGTGGAGCCTGCGGCTTA
ATTTGACTCAACACGGGGAAACTTACCAGGTCAAAACATGGGTGGGATTGACAGATTGAGAGCTCTTTCTTGATTCTATGGGTGGTGGT
GCATGGCCGTTCTTAGTTGGTGGAGTGATTTGTCTGGTTAATTCCGTTAACGAACGAGACCTTAACCTGCTAACTAGTCTTTTAGTATA
ATTACTAAAAGTACTTCTTAGAGGGACTATGCGACGAAACGCATGGAAGTTTGAGGCAATAACAGGTCTGTGATGCCCTTAGATGTCCT
GGGCCGCACGTGCGCTACAATGACACGTTCAACAAGTTTTTTCCTGGCCCGGAAGGGTACGGGTAATCTTTTTAATGCGTGTCGTGTTA
GGGATAGATCTTTGGAATTATAGATCTTGAACGAGGAATTCCTAGTAAGTGCAAGTCATCAACTTGTACTGATTACGTCCCTGCCCTTT
GTACACACCGCCCGTCGCTCCTACCGATTTCGAGTGATCCGGTGAACCTTCTGGACTAGGTCGTCCTTGAGACGGACTTGGGAAGTTAA
GTAAACCTTATCACTTAGAGGAAGGAGAAGTCGTAACAAGGTTTCCGTAGGTGAACCTGCAGAAGGATCA

>Haptophrya_planariarum_BB_99_DG
AACCTGGTTGATCCTGCCAGTAGTCATATGCTTGTCTCAAAGATTAAGCCATGCATGTCTAAGTATAATAGTATACAGTAAAACTGCGA
ATGGCTCATTACAACAGTTATAGTTTATTTGATAATGGAAGTCTACATGGATAACCGTGGTAATTCTAGAGCTAATACATGCTGTTAAA
CCTAACTTTACGGAAGGGTTGTATTTATTAGATATTAAGCCAATATTCTTTCGGGATTATTGCGAGGAATCATAATAACTGATCGAATC
TCAAATTTGAGATAAATCATTCAAGTTTCTGCCCTATCAGCTTTCGATGGTAGTGTATTGGACTACCATGGCAGTCACGGGTAACGGAG
AATTAGGGTTCGGTTCCGGAGAGGGAGCCTGAGAAACGGCTACCACATCTAAGGAAGGCAGCAGGCGCGTAAATTACCCAATCCTGATT
CAGGGAGGTAGTGACAAGAAATAACAACTCGGGGATTTTTAATCTTACGGGATTGCAATGAGAACAATTTAAACCACTTAGCGAGGAAC
AATTGGAGGGCAAGTCTGGTGCCAGCAGCCGCGGTAATTCCAGCTCCAATAGCGTATATTAAAGTTGTTGCAGTTAAAAAGCTCGTAGT
TGAATTTCTGGCTTAGCTTAAACGCGGCTTCGGTCAGTTTAAGATAAGTCATCCGTCTGCAAACCATATTCGTCCTTCACGGGTTCGAT
TTGGGGAGTAGATTTTTTACTTTGAAAAAATTAGAGTGTTTCAGGCAGGTTATCGCCCGAATACATTAGCATGGAATAATGGAATAGGA
CTTTTGTCCATTTGGTTGGTTATTGGACATAAGTAATGATTAATAGGGACAGTTGGGGGCATTAGTATTTAATTGTCAGAGGTGAAATT
CTTGGATTTATTAAAGACTAACTTATGCGAAAGCATTTGCCAAGGATGTTTTCATTAATCAAGAACGAAAGTTAAGGGATCAAATACGA
TCAGATACCGTAGTAGTCTTAACTATAAACTATACCGACTCGGGATTGGATGGGTATATGCCCATTCAGCACCGTATGAGAAATCAAAG
TCTTTGGGTTCTGGGGGGAGTATGGTCGCAAGGCTGAAACTTAAAGGAATTGACGGAAGGGCACCACCAGGAGTGGAGCCTGCGGCTTA
ATTTGACTCAACACGGGGAAACTTACCAGGTCAAAACATGGGTGGGATTGACAGATTGAGAGCTCTTTCTTGATTCTATGGGTGGTGGT
GCATGGCCGTTCTTAGTTGGTGGAGTGATTTGTCTGGTTAATTCCGTTAACGAACGAGACCTTAACCTGCTAACTAGTCTTTTAGTATA
ATTACTAAAAGTACTTCTTAGAGGGACTATGCGACGAAACGCATGGAAGTTTGAGGCAATAACAGGTCTGTGATGCCCTTAGATGTCCT
GGGCCGCACGTGCGCTACAATGACACGTTCAACAAGTTTTTTCCTGGCCCGGAAGGGTACGGGTAATCTTTTTAATGCGTGTCGTGTTA
GGGATAGATCTTTGGAATTATAGATCTTGAACGAGGAATTCCTAGTAAGTGCAAGTCATCAACTTGTACTGATTACGTCCCTGCCCTTT
GTACACACCGCCCGTCGCTCCTACCGATTTCGAGTGATCCGGTGAACCTTCTGGACTAGGTCGTCCTTGAGACGGACTTGGGAAGTTAA
GTAAACCTTATCACTTAGAGGAAGGAGAAGTCGTAACAAGGTTTCCGTAGGTGAACCTGCAGAAGGATCA
>Haptophrya_planariarum_ST_132_DG
AACCTGGTTGATCCTGCCAGTAGTCATATGCTTGTCTCAAAGATTAAGCCATGCATGTCTAAGTATAATAGTATACAGTAAAACTGCGA
ATGGCTCATTACAACAGTTATAGTTTATTTGATAATGGAAGTCTACATGGATAACCGTGGTAATTCTAGAGCTAATACATGCTGTTAAA
CCTAACTTTACGGAAGGGTTGTATTTATTAGATATTAAGCCAATATTCTTTCGGGATTATTGCGAGGAATCATAATAACTGATCGAATC
TCAAATTTGAGATAAATCATTCAAGTTTCTGCCCTATCAGCTTTCGATGGTAGTGTATTGGACTACCATGGCAGTCACGGGTAACGGAG
AATTAGGGTTCGGTTCCGGAGAGGGAGCCTGAGAAACGGCTACCACATCTAAGGAAGGCAGCAGGCGCGTAAATTACCCAATCCTGATT
CAGGGAGGTAGTGACAAGAAATAACAACTCGGGGATTTTTAATCTTACGGGATTGCAATGAGAACAATTTAAACCACTTAGCGAGGAAC
AATTGGAGGGCAAGTCTGGTGCCAGCAGCCGCGGTAATTCCAGCTCCAATAGCGTATATTAAAGTTGTTGCAGTTAAAAAGCTCGTAGT
TGAATTTCTGGCTTAGCTTAAACGCGGCTTCGGTCAGTTTAAGATAAGTCATCCGTCTGCAAACCATATTCGTCCTTCACGGGTTCGAT
TTGGGGAGTAGATTTTTTACTTTGAAAAAATTAGAGTGTTTCAGGCAGGTTATCGCCCGAATACATTAGCATGGAATAATGGAATAGGA
CTTTTGTCCATTTGGTTGGTTATTGGACATAAGTAATGATTAATAGGGACAGTTGGGGGCATTAGTATTTAATTGTCAGAGGTGAAATT
CTTGGATTTATTAAAGACTAACTTATGCGAAAGCATTTGCCAAGGATGTTTTCATTAATCAAGAACGAAAGTTAAGGGATCAAAGACGA
TCAGATACCGTAGTAGTCTTAACTATAAACTATACCGACTCGGGATTGGATGGGTATATGCCCATTCAGCACCGTATGAGAAATCAAAG
TCTTTGGGTTCTGGGGGGAGTATGGTCGCAAGGCTGAAACTTAAAGGAATTGACGGAAGGGCACCACCAGGAGTGGAGCCTGCGGCTTA
ATTTGACTCAACACGGGGAAACTTACCAGGTCAAAACATGGGTGGGATTGACAGATTGAGAGCTCTTTCTTGATTCTATGGGTGGTGGT
GCATGGCCGTTCTTAGTTGGTGGAGTGATTTGTCTGGTTAATTCCGTTAACGAACGAGACCTTAACCTGCTAACTAGTCTTTTAGTATA
ATTACTAAAAGTACTTCTTAGAGGGACTATGCGACGAAACGCATGGAAGTTTGAGGCAATAACAGGTCTGTGATGCCCTTAGATGTCCT
GGGCCGCACGTGCGCTACAATGACACGTTCAACAAGTTTTTTCCTGGCCCGGAAGGGTACGGGTAATCTTTTTAATGCGTGTCGTGTTA
GGGATAGATCTTTGGAATTATAGATCTTGAACGAGGAATTCCTAGTAAGTGCAAGTCATCAACTTGTACTGATTACGTCCCTGCCCTTT
GTACACACCGCCCGTCGCTCCTACCGATTTCGAGTGATCCGGTGAACCTTCTGGACTAGGTCGTCCTTGAGACGGACTTGGGAAGTTAA
GTAAACCTTATCACTTAGAGGAAGGAGAAGTCGTAACAAGGTTTCCGTAGGTGAACCTGCAGAAGGATCA
>Haptophrya_planariarum_BY_139_DG
AACCTGGTTGATCCTGCCAGTAGTCATATGCTTGTCTCAAAGATTAAGCCATGCATGTCTAAGTATAATAGTATACAGTAAAACTGCGA
ATGGCTCATTACAACAGTTATAGTTTATTTGATAATGGAAGTCTACATGGATAACCGTGGTAATTCTAGAGCTAATACATGCTGTTAAA
CCTAACTTTACGGAAGGGTTGTATTTATTAGATATTAAGCCAATATTCTTTCGGGATTATTGCGAGGAATCATAATAACTGATCGAATC
TCAAATTTGAGATAAATCATTCAAGTTTCTGCCCTATCAGCTTTCGATGGTAGTGTATTGGACTACCATGGCAGTCACGGGTAACGGAG
AATTAGGGTTCGGTTCCGGAGAGGGAGCCTGAGAAACGGCTACCACATCTAAGGAAGGCAGCAGGCGCGTAAATTACCCAATCCTGATT
CAGGGAGGTAGTGACAAGAAATAACAACTCGGGGATTTTTAATCTTACGGGATTGCAATGAGAACAATTTAAACCACTTAGCGAGGAAC
AATTGGAGGGCAAGTCTGGTGCCAGCAGCCGCGGTAATTCCAGCTCCAATAGCGTATATTAAAGTTGTTGCAGTTAAAAAGCTCGTAGT
TGAATTTCTGGCTTAGCTTAAACGCGGCTTCGGTCAGTTTAAGATAAGTCATCCGTCTGCAAACCATATTCGTCCTTCACGGGTTCGAT
TTGGGGAGTAGATTTTTTACTTTGAAAAAATTAGAGTGTTTCAGGCAGGTTATCGCCCGAATACATTAGCATGGAATAATGGAATAGGA
CTTTTGTCCATTTGGTTGGTTATTGGACATAAGTAATGATTAATAGGGACAGTTGGGGGCATTAGTATTTAATTGTCAGAGGTGAAATT
CTTGGATTTATTAAAGACTAACTTATGCGAAAGCATTTGCCAAGGATGTTTTCATTAATCAAGAACGAAAGTTAAGGGATCAAAGACGA
TCAGATACCGTAGTAGTCTTAACTATAAACTATACCGACTCGGGATTGGATGGGTATATGCCCATTCAGCACCGTATGAGAAATCAAAG
TCTTTGGGTTCTGGGGGGAGTATGGTCGCAAGGCTGAAACTTAAAGGAATTGACGGAAGGGCACCACCAGGAGTGGAGCCTGCGGCTTA
ATTTGACTCAACACGGGGAAACTTACCAGGTCAAAACATGGGTGGGATTGACAGATTGAGAGCTCTTTCTTGATTCTATGGGTGGTGGT
GCATGGCCGTTCTTAGTTGGTGGAGTGATTTGTCTGGTTAATTCCGTTAACGAACGAGACCTTAACCTGCTAACTAGTCTTTTAGTATA
ATTACTAAAAGTACTTCTTAGAGGGACTATGCGACGAAACGCATGGAAGTTTGAGGCAATAACAGGTCTGTGATGCCCTTAGATGTCCT
GGGCCGCACGTGCGCTACAATGACACGTTCAACAAGTTTTTTCCTGGCCCGGAAGGGTACGGGTAATCTTTTTAATGCGTGTCGTGTTA
GGGATAGATCTTTGGAATTATAGATCTTGAACGAGGAATTCCTAGTAAGTGCAAGTCATCAACTTGTACTGATTACGTCCCTGCCCTTT
GTACACACCGCCCGTCGCTCCTACCGATTTCGAGTGATCCGGTGAACCTTCTGGACTAGGTCGTCCTTGAGACGGACTTGGGAAGTTAA
GTAAACCTTATCACTTAGAGGAAGGAGAAGTCGTAACAAGGTTTCCGTAGGTGAACCTGCAGAAGGATCA
>Haptophrya_planariarum_BY_149_DG
AACCTGGTTGATCCTGCCAGTAGTCATATGCTTGTCTCAAAGATTAAGCCATGCATGTCTAAGTATAATAGTATACAGTAAAACTGCGA
ATGGCTCATTACAACAGTTATAGTTTATTTGATAATGGAAGTCTACATGGATAACCGTGGTAATTCTAGAGCTAATACATGCTGTTAAA
CCTAACTTTACGGAAGGGTTGTATTTATTAGATATTAAGCCAATATTCTTTCGGGATTATTGCGAGGAATCATAATAACTGATCGAATC
TCAAATTTGAGATAAATCATTCAAGTTTCTGCCCTATCAGCTTTCGATGGTAGTGTATTGGACTACCATGGCAGTCACGGGTAACGGAG
AATTAGGGTTCGGTTCCGGAGAGGGAGCCTGAGAAACGGCTACCACATCTAAGGAAGGCAGCAGGCGCGTAAATTACCCAATCCTGATT
CAGGGAGGTAGTGACAAGAAATAACAACTCGGGGATTTTTAATCTTACGGGATTGCAATGAGAACAATTTAAACCACTTAGCGAGGAAC
AATTGGAGGGCAAGTCTGGTGCCAGCAGCCGCGGTAATTCCAGCTCCAATAGCGTATATTAAAGTTGTTGCAGTTAAAAAGCTCGTAGT

TGAATTTCTGGCTTAGCTTAAACGCGGCTTCGGTCAGTTTAAGATAAGTCATCCGTCTGCAAACCATATTCGTCCTTCACGGGTTCGAT
TTGGGGAGTAGATTTTTTACTTTGAAAAAATTAGAGTGTTTCAGGCAGGTTATCGCCCGAATACATTAGCATGGAATAATGGAATAGGA
CTTTTGTCCATTTGGTTGGTTATTGGACATAAGTAATGATTAATAGGGACAGTTGGGGGCATTAGTATTTAATTGTCAGAGGTGAAATT
CTTGGATTTATTAAAGACTAACTTATGCGAAAGCATTTGCCAAGGATGTTTTCATTAATCAAGAACGAAAGTTAAGGGATCAAATACGA
TCAGATACCGTAGTAGTCTTAACTATAAACTATACCGACTCGGGATTGGATGGGTATATGCCCATTCAGCACCGTATGAGAAATCAAAG
TCTTTGGGTTCTGGGGGGAGTATGGTCGCAAGGCTGAAACTTAAAGGAATTGACGGAAGGGCACCACCAGGAGTGGAGCCTGCGGCTTA
ATTTGACTCAACACGGGGAAACTTACCAGGTCAAAACATGGGTGGGATTGACAGATTGAGAGCTCTTTCTTGATTCTATGGGTGGTGGT
GCATGGCCGTTCTTAGTTGGTGGAGTGATTTGTCTGGTTAATTCCGTTAACGAACGAGACCTTAACCTGCTAACTAGTCTTTTAGTATA
ATTACTAAAAGTACTTCTTAGAGGGACTATGCGACGAAACGCATGGAAGTTTGAGGCAATAACAGGTCTGTGATGCCCTTAGATGTCCT
GGGCCGCACGTGCGCTACAATGACACGTTCAACAAGTTTTTTCCTGGCCCGGAAGGGTACGGGTAATCTTTTTAATGCGTGTCGTGTTA
GGGATAGATCTTTGGAATTATAGATCTTGAACGAGGAATTCCTAGTAAGTGCAAGTCATCAACTTGTACTGATTACGTCCCTGCCCTTT
GTACACACCGCCCGTCGCTCCTACCGATTTCGAGTGATCCGGTGAACCTTCTGGACTAGGTCGTCCTTGAGACGGACTTGGGAAGTTAA
GTAAACCTTATCACTTAGAGGAAGGAGAAGTCGTAACAAGGTTTCCGTAGGTGAACCTGCAGAAGGATCA
>Haptophrya_dugesiarum_BB_78_DG
AACCTGGTTGATCCTGCCAGTAGTCATATGCTTGTCTCAAAGATTAAGCCATGCATGTCTAAGTATAATAGTATACAGTAAAACTGCGA
ATGGCTCATTACAACAGTTATAGTTTATTTGATAATGGAAGTCTACATGGATAACCGTGGTAATTCTAGAGCTAATACATGCTGTTAAA
CCTAACTTTACGGAAGGGTTGTATTTATTAGATATTAAGCCAATATTCTTTCGGGATTATTGCGAGGAATCATAATAACTGATCGAATC
TCAAATTTGAGATAAATCATTCAAGTTTCTGCCCTATCAGCTTTCGATGGTAGTGTATTGGACTACCATGGCAGTCACGGGTAACGGAG
AATTAGGGTTCGGTTCCGGAGAGGGAGCCTGAGAAACGGCTACCACATCTAAGGAAGGCAGCAGGCGCGTAAATTACCCAATCCTGATT
CAGGGAGGTAGTGACAAGAAATAACAACTCGGGGATTTTTTATCTTACGGGATTGCAATGAGAACAATTTAAACCACTTAGCGAGGAAC
AATTGGAGGGCAAGTCTGGTGCCAGCAGCCGCGGTAATTCCAGCTCCAATAGCGTATATTAAAGTTGTTGCAGTTAAAAAGCTCGTAGT
TGAATTTCTGGCTTAGCTTAAACGCGGCTTCGGTCAGTTTAAGATAAGTCATCCGTCTGCAAACCATATTCGTCCTTCACGGGTTCGAT
TTGGGGAGTAGATTTTTTACTTTGAAAAAATTAGAGTGTTTCAGGCAGGTTATCGCCCGAATACATTAGCATGGAATAATGGAATAGGA
CTTTTGTCCATTTGGTTGGTTATTGGACATAAGTAATGATTAATAGGGACAGTTGGGGGCATTAGTATTTAATTGTCAGAGGTGAAATT
CTTGGATTTATTAAAGACTAACTTATGCGAAAGCATTTGCCAAGGATGTTTTCATTAATCAAGAACGAAAGTTAAGGGATCAAAGACGA
TCAGATACCGTAGTAGTCTTAACTATAAACTATACCGACTCGGGATTGGATGGGTATATGCCCATTCAGCACCGTATGAGAAATCAAAG
TCTTTGGGTTCTGGGGGGAGTATGGTCGCAAGGCTGAAACTTAAAGGAATTGACGGAAGGGCACCACCAGGAGTGGAGCCTGCGGCTTA
ATTTGACTCAACACGGGGAAACTTACCAGGTCAAAACATGGGTGGGATTGACAGATTGAGAGCTCTTTCTTGATTCTATGGGTGGTGGT
GCATGGCCGTTCTTAGTTGGTGGAGTGATTTGTCTGGTTAATTCCGTTAACGAACGAGACCTTAACCTGCTAAATAGTCTTTTAGTATA
ATTACTAAAAGTACTTCTTAGAGGGACTATGCGACGAAACGCATGGAAGTTTGAGGCAATAACAGGTCTGTGATGCCCTTAGATGTCCT
GGGCCGCACGTGCGCTACAATGACACGTTCAACAAGTTTTTTCCTGGCCCGGAAGGGTACGGGTAATCTTTTTAATGCGTGTCGTGTTA
GGGATAGATCTTTGGAATTATAGATCTTGAACGAGGAATTCCTAGTAAGTGCAAGTCATCAACTTGTACTGATTACGTCCCTGCCCTTT
GTACACACCGCCCGTCGCTCCTACCGATTTCGAGTGATCCGGTGAACCTTCTGGACTAGGTCGTCCTTGAGACGGACTTGGGAAGTTAA
GTAAACCTTATCACTTAGAGGAAGGAGAAGTCGTAACAAGGTTTCCGTAGGTGAACCTGCAGAAGGATCA
>Haptophrya_dugesiarum_BB_79_DG
AACCTGGTTGATCCTGCCAGTAGTCATATGCTTGTCTCAAAGATTAAGCCATGCATGTCTAAGTATAATAGTATACAGTAAAACTGCGA
ATGGCTCATTACAACAGTTATAGTTTATTTGATAATGGAAGTCTACATGGATAACCGTGGTAATTCTAGAGCTAATACATGCTGTTAAA
CCTAACTTTACGGAAGGGTTGTATTTATTAGATATTAAGCCAATATTCTTTCGGGATTATTGCGAGGAATCATAATAACTGATCGAATC
TCAAATTTGAGATAAATCATTCAAGTTTCTGCCCTATCAGCTTTCGATGGTAGTGTATTGGACTACCATGGCAGTCACGGGTAACGGAG
AATTAGGGTTCGGTTCCGGAGAGGGAGCCTGAGAAACGGCTACCACATCTAAGGAAGGCAGCAGGCGCGTAAATTACCCAATCCTGATT
CAGGGAGGTAGTGACAAGAAATAACAACTCGGGGATTTTTTATCTTACGGGATTGCAATGAGAACAATTTAAACCACTTAGCGAGGAAC
AATTGGAGGGCAAGTCTGGTGCCAGCAGCCGCGGTAATTCCAGCTCCAATAGCGTATATTAAAGTTGTTGCAGTTAAAAAGCTCGTAGT
TGAATTTCTGGCTTAGCTTAAACGCGGCTTCGGTCAGTTTAAGATAAGTCATCCGTCTGCAAACCATATTCGTCCTTCACGGGTTCGAT
TTGGGGAGTAGATTTTTTACTTTGAAAAAATTAGAGTGTTTCAGGCAGGTTATCGCCCGAATACATTAGCATGGAATAATGGAATAGGA
CTTTTGTCCATTTGGTTGGTTATTGGACATAAGTAATGATTAATAGGGACAGTTGGGGGCATTAGTATTTAATTGTCAGAGGTGAAATT
CTTGGATTTATTAAAGACTAACTTATGCGAAAGCATTTGCCAAGGATGTTTTCATTAATCAAGAACGAAAGTTAAGGGATCAAATACGA
TCAGATACCGTAGTAGTCTTAACTATAAACTATACCGACTCGGGATTGGATGGGTATATGCCCATTCAGCACCGTATGAGAAATCAAAG
TCTTTGGGTTCTGGGGGGAGTATGGTCGCAAGGCTGAAACTTAAAGGAATTGACGGAAGGGCACCACCAGGAGTGGAGCCTGCGGCTTA
ATTTGACTCAACACGGGGAAACTTACCAGGTCAAAACATGGGTGGGATTGACAGATTGAGAGCTCTTTCTTGATTCTATGGGTGGTGGT
GCATGGCCGTTCTTAGTTGGTGGAGTGATTTGTCTGGTTAATTCCGTTAACGAACGAGACCTTAACCTGCTAAATAGTCTTTTAGTATA
ATTACTAAAAGTACTTCTTAGAGGGACTATGCGACGAAACGCATGGAAGTTTGAGGCAATAACAGGTCTGTGATGCCCTTAGATGTCCT
GGGCCGCACGTGCGCTACAATGACACGTTCAACAAGTTTTTTCCTGGCCCGGAAGGGTACGGGTAATCTTTTTAATGCGTGTCGTGTTA
GGGATAGATCTTTGGAATTATAGATCTTGAACGAGGAATTCCTAGTAAGTGCAAGTCATCAACTTGTACTGATTACGTCCCTGCCCTTT
GTACACACCGCCCGTCGCTCCTACCGATTTCGAGTGATCCGGTGAACCTTCTGGACTAGGTCGTCCTTGAGACGGACTTGGGAAGTTAA
GTAAACCTTATCACTTAGAGGAAGGAGAAGTCGTAACAAGGTTTCCGTAGGTGAACCTGCAGAAGGATCA
>Haptophrya_dugesiarum_BB_87_DG
AACCTGGTTGATCCTGCCAGTAGTCATATGCTTGTCTCAAAGATTAAGCCATGCATGTCTAAGTATAATAGTATACAGTAAAACTGCGA
ATGGCTCATTACAACAGTTATAGTTTATTTGATAATGGAAGTCTACATGGATAACCGTGGTAATTCTAGAGCTAATACATGCTGTTAAA
CCTAACTTTACGGAAGGGTTGTATTTATTAGATATTAAGCCAATATTCTTTCGGGATTATTGCGAGGAATCATAATAACTGATCGAATC
TCAAATTTGAGATAAATCATTCAAGTTTCTGCCCTATCAGCTTTCGATGGTAGTGTATTGGACTACCATGGCAGTCACGGGTAACGGAG
AATTAGGGTTCGGTTCCGGAGAGGGAGCCTGAGAAACGGCTACCACATCTAAGGAAGGCAGCAGGCGCGTAAATTACCCAATCCTGATT
CAGGGAGGTAGTGACAAGAAATAACAACTCGGGGATTTTTTATCTTACGGGATTGCAATGAGAACAATTTAAACCACTTAGCGAGGAAC
AATTGGAGGGCAAGTCTGGTGCCAGCAGCCGCGGTAATTCCAGCTCCAATAGCGTATATTAAAGTTGTTGCAGTTAAAAAGCTCGTAGT
TGAATTTCTGGCTTAGCTTAAACGCGGCTTCGGTCAGTTTAAGATAAGTCATCCGTCTGCAAACCATATTCGTCCTTCACGGGTTCGAT
TTGGGGAGTAGATTTTTTACTTTGAAAAAATTAGAGTGTTTCAGGCAGGTTATCGCCCGAATACATTAGCATGGAATAATGGAATAGGA
CTTTTGTCCATTTGGTTGGTTATTGGACATAAGTAATGATTAATAGGGACAGTTGGGGGCATTAGTATTTAATTGTCAGAGGTGAAATT
CTTGGATTTATTAAAGACTAACTTATGCGAAAGCATTTGCCAAGGATGTTTTCATTAATCAAGAACGAAAGTTAAGGGATCAAAGACGA
TCAGATACCGTAGTAGTCTTAACTATAAACTATACCGACTCGGGATTGGATGGGTATATGCCCATTCAGCACCGTATGAGAAATCAAAG
TCTTTGGGTTCTGGGGGGAGTATGGTCGCAAGGCTGAAACTTAAAGGAATTGACGGAAGGGCACCACCAGGAGTGGAGCCTGCGGCTTA
ATTTGACTCAACACGGGGAAACTTACCAGGTCAAAACATGGGTGGGATTGACAGATTGAGAGCTCTTTCTTGATTCTATGGGTGGTGGT
GCATGGCCGTTCTTAGTTGGTGGAGTGATTTGTCTGGTTAATTCCGTTAACGAACGAGACCTTAACCTGCTAAATAGTCTTTTAGTATA

ATTACTAAAAGTACTTCTTAGAGGGACTATGCGACGAAACGCATGGAAGTTTGAGGCAATAACAGGTCTGTGATGCCCTTAGATGTCCT
GGGCCGCACGTGCGCTACAATGACACGTTCAACAAGTTTTTTCCTGGCCCGGAAGGGTACGGGTAATCTTTTTAATGCGTGTCGTGTTA
GGGATAGATCTTTGGAATTATAGATCTTGAACGAGGAATTCCTAGTAAGTGCAAGTCATCAACTTGTACTGATTACGTCCCTGCCCTTT
GTACACACCGCCCGTCGCTCCTACCGATTTCGAGTGATCCGGTGAACCTTCTGGACTAGGTCGTCCTTGAGACGGACTTGGGAAGTTAA
GTAAACCTTATCACTTAGAGGAAGGAGAAGTCGTAACAAGGTTTCCGTAGGTGAACCTGCAGAAGGATCA
>Haptophrya_dugesiarum_BB_98_DG
AACCTGGTTGATCCTGCCAGTAGTCATATGCTTGTCTCAAAGATTAAGCCATGCATGTCTAAGTATAATAGTATACAGTAAAACTGCGA
ATGGCTCATTACAACAGTTATAGTTTATTTGATAATGGAAGTCTACATGGATAACCGTGGTAATTCTAGAGCTAATACATGCTGTTAAA
CCTAACTTTACGGAAGGGTTGTATTTATTAGATATTAAGCCAATATTCTTTCGGGATTATTGCGAGGAATCATAATAACTGATCGAATC
TCAAATTTGAGATAAATCATTCAAGTTTCTGCCCTATCAGCTTTCGATGGTAGTGTATTGGACTACCATGGCAGTCACGGGTAACGGAG
AATTAGGGTTCGGTTCCGGAGAGGGAGCCTGAGAAACGGCTACCACATCTAAGGAAGGCAGCAGGCGCGTAAATTACCCAATCCTGATT
CAGGGAGGTAGTGACAAGAAATAACAACTCGGGGATTTTTTATCTTACGGGATTGCAATGAGAACAATTTAAACCACTTAGCGAGGAAC
AATTGGAGGGCAAGTCTGGTGCCAGCAGCCGCGGTAATTCCAGCTCCAATAGCGTATATTAAAGTTGTTGCAGTTAAAAAGCTCGTAGT
TGAATTTCTGGCTTAGCTTAAACGCGGCTTCGGTCAGTTTAAGATAAGTCATCCGTCTGCAAACCATATTCGTCCTTCACGGGTTCGAT
TTGGGGAGTAGATTTTTTACTTTGAAAAAATTAGAGTGTTTCAGGCAGGTTATCGCCCGAATACATTAGCATGGAATAATGGAATAGGA
CTTTTGTCCATTTGGTTGGTTATTGGACATAAGTAATGATTAATAGGGACAGTTGGGGGCATTAGTATTTAATTGTCAGAGGTGAAATT
CTTGGATTTATTAAAGACTAACTTATGCGAAAGCATTTGCCAAGGATGTTTTCATTAATCAAGAACGAAAGTTAAGGGATCAAATACGA
TCAGATACCGTAGTAGTCTTAACTATAAACTATACCGACTCGGGATTGGATGGGTATATGCCCATTCAGCACCGTATGAGAAATCAAAG
TCTTTGGGTTCTGGGGGGAGTATGGTCGCAAGGCTGAAACTTAAAGGAATTGACGGAAGGGCACCACCAGGAGTGGAGCCTGCGGCTTA
ATTTGACTCAACACGGGGAAACTTACCAGGTCAAAACATGGGTGGGATTGACAGATTGAGAGCTCTTTCTTGATTCTATGGGTGGTGGT
GCATGGCCGTTCTTAGTTGGTGGAGTGATTTGTCTGGTTAATTCCGTTAACGAACGAGACCTTAACCTGCTAAATAGTCTTTTAGTATA
ATTACTAAAAGTACTTCTTAGAGGGACTATGCGACGAAACGCATGGAAGTTTGAGGCAATAACAGGTCTGTGATGCCCTTAGATGTCCT
GGGCCGCACGTGCGCTACAATGACACGTTCAACAAGTTTTTTCCTGGCCCGGAAGGGTACGGGTAATCTTTTTAATGCGTGTCGTGTTA
GGGATAGATCTTTGGAATTATAGATCTTGAACGAGGAATTCCTAGTAAGTGCAAGTCATCAACTTGTACTGATTACGTCCCTGCCCTTT
GTACACACCGCCCGTCGCTCCTACCGATTTCGAGTGATCCGGTGAACCTTCTGGACTAGGTCGTCCTTGAGACGGACTTGGGAAGTTAA
GTAAACCTTATCACTTAGAGGAAGGAGAAGTCGTAACAAGGTTTCCGTAGGTGAACCTGCAGAAGGATCA
>Haptophrya_dugesiarum_BY_142_DG
AACCTGGTTGATCCTGCCAGTAGTCATATGCTTGTCTCAAAGATTAAGCCATGCATGTCTAAGTATAATAGTATACAGTAAAACTGCGA
ATGGCTCATTACAACAGTTATAGTTTATTTGATAATGGAAGTCTACATGGATAACCGTGGTAATTCTAGAGCTAATACATGCTGTTAAA
CCTAACTTTACGGAAGGGTTGTATTTATTAGATATTAAGCCAATATTCTTTCGGGATTATTGCGAGGAATCATAATAACTGATCGAATC
TCAAATTTGAGATAAATCATTCAAGTTTCTGCCCTATCAGCTTTCGATGGTAGTGTATTGGACTACCATGGCAGTCACGGGTAACGGAG
AATTAGGGTTCGGTTCCGGAGAGGGAGCCTGAGAAACGGCTACCACATCTAAGGAAGGCAGCAGGCGCGTAAATTACCCAATCCTGATT
CAGGGAGGTAGTGACAAGAAATAACAACTCGGGGATTTTTTATCTTACGGGATTGCAATGAGAACAATTTAAACCACTTAGCGAGGAAC
AATTGGAGGGCAAGTCTGGTGCCAGCAGCCGCGGTAATTCCAGCTCCAATAGCGTATATTAAAGTTGTTGCAGTTAAAAAGCTCGTAGT
TGAATTTCTGGCTTAGCTTAAACGCGGCTTCGGTCAGTTTAAGATAAGTCATCCGTCTGCAAACCATATTCGTCCTTCACGGGTTCGAT
TTGGGGAGTAGATTTTTTACTTTGAAAAAATTAGAGTGTTTCAGGCAGGTTATCGCCCGAATACATTAGCATGGAATAATGGAATAGGA
CTTTTGTCCATTTGGTTGGTTATTGGACATAAGTAATGATTAATAGGGACAGTTGGGGGCATTAGTATTTAATTGTCAGAGGTGAAATT
CTTGGATTTATTAAAGACTAACTTATGCGAAAGCATTTGCCAAGGATGTTTTCATTAATCAAGAACGAAAGTTAAGGGATCAAATACGA
TCAGATACCGTAGTAGTCTTAACTATAAACTATACCGACTCGGGATTGGATGGGTATATGCCCATTCAGCACCGTATGAGAAATCAAAG
TCTTTGGGTTCTGGGGGGAGTATGGTCGCAAGGCTGAAACTTAAAGGAATTGACGGAAGGGCACCACCAGGAGTGGAGCCTGCGGCTTA
ATTTGACTCAACACGGGGAAACTTACCAGGTCAAAACATGGGTGGGATTGACAGATTGAGAGCTCTTTCTTGATTCTATGGGTGGTGGT
GCATGGCCGTTCTTAGTTGGTGGAGTGATTTGTCTGGTTAATTCCGTTAACGAACGAGACCTTAACCTGCTAAATAGTCTTTTAGTATA
ATTACTAAAAGTACTTCTTAGAGGGACTATGCGACGAAACGCATGGAAGTTTGAGGCAATAACAGGTCTGTGATGCCCTTAGATGTCCT
GGGCCGCACGTGCGCTACAATGACACGTTCAACAAGTTTTTTCCTGGCCCGGAAGGGTACGGGTAATCTTTTTAATGCGTGTCGTGTTA
GGGATAGATCTTTGGAATTATAGATCTTGAACGAGGAATTCCTAGTAAGTGCAAGTCATCAACTTGTACTGATTACGTCCCTGCCCTTT
GTACACACCGCCCGTCGCTCCTACCGATTTCGAGTGATCCGGTGAACCTTCTGGACTAGGTCGTCCTTGAGACGGACTTGGGAAGTTAA
GTAAACCTTATCACTTAGAGGAAGGAGAAGTCGTAACAAGGTTTCCGTAGGTGAACCTGCAGAAGGATCA
>Hplan_110_JurJaz_SchPol
AACCTGGTTGATCCTGCCAGTAGTCATATGCTTGTCTCAAAGATTAAGCCATGCATGTCTAAGTATAATAGTATACAGTAAAACTGCGA
ATGGCTCATTACAACAGTTATAGTTTATTTGATAATGGAAGTCTACATGGATAACCGTGGTAATTCTAGAGCTAATACATGCTGTTAAA
CCTAACTTTACGGAAGGGTTGTATTTATTAGATATTAAGCCAATATTCTTTCGGGATTATTGCGAGGAATCATAATAACTGATCGAATC
TCAAATTTGAGATAAATCATTCAAGTTTCTGCCCTATCAGCTTTCGATGGTAGTGTATTGGACTACCATGGCAGTCACGGGTAACGGAG
AATTAGGGTTCGGTTCCGGAGAGGGAGCCTGAGAAACGGCTACCACATCTAAGGAAGGCAGCAGGCGCGTAAATTACCCAATCCTGATT
CAGGGAGGTAGTGACAAGAAATAACAACTCGGGGATTTTTAATCTTACGGGATTGCAATGAGAACAATTTAAACCACTTAGCGAGGAAC
AATTGGAGGGCAAGTCTGGTGCCAGCAGCCGCGGTAATTCCAGCTCCAATAGCGTATATTAAAGTTGTTGCAGTTAAAAAGCTCGTAGT
TGAATTTCTGGCTTAATTTAATCGCGGCTTCGGTCAGGTTAAAATAAGTCATCCGTTTGCAAACCATTTTCGTCCTTCACAGGTTCGAT
TTGGGGAGTAAACCTTTTACTTTGAAAAAATTAGAGTGTTTCAGGCAGGTTATAGCCCGAATACATTAGCATGGAATAATGGAATAGGA
CTTTTGTCCATTTGGTTGGTTATTGGACATAAGTAATGATTAATAGGGACAGTTGGGGGCATTAGTATTTAATTGTCAGAGGTGAAATT
CTTGGATTTATTAAAGACTAACTTATGCGAAAGCATTTGCCAAGGATGTTTTCATTAATCAAGAACGAAAGTTAAGGGATCAAATACGA
TCAGATACCGTAGTAGTCTTAACTATAAACTATACCGACTCGGGATTGGATGGGTATATGCCCATTCAGCACCGTATGAGAAATCAAAG
TCTTTGGGTTCTGGGGGGAGTATGGTCGCAAGGCTGAAACTTAAAGGAATTGACGGAAGGGCACCACCAGGAGTGGAGCCTGCGGCTTA
ATTTGACTCAACACGGGGAAACTTACCAGGTCAAAACATGGGTGGGATTGACAGATTGAGAGCTCTTTCTTGATTCTATGGGTGGTGGT
GCATGGCCGTTCTTAGTTGGTGGAGTGATTTGTCTGGTTAATTCCGTTAACGAACGAGACCTTAACCTGCTAACTAGTCTTTTAGTATA
-TTACTAAAAGTATTTCTTAGAGGGACTATGCGATGAAACGCATGGAAGTTTGAGGCAATAACAGGTCTGTGATGCCCTTAGATGTCCT
GGGCCGCACGTGCGCTACAATGACACGTTCAACAAGTTATTTCCTGGCCCGGAAGGGTACGGGTAATCTTTTTAATGCGTGTCGTGTTA
GGGATAGATCTTTGGAATTATAGATCTTGAACGAGGAATTCCTAGTAAGTGCAAGTCATCAACTTGTACTGATTACGTCCCTGCCCTTT
GTACACACCGCCCGTCGCTCCTACCGATTTCGAGTGATCCGGTGAACCTTCTGGACTGGGTCGTCCTTGAGACGGACTTGGGAAGTTAA
GTAAACCTTATCACTTAGAGGAAGGAGAAGTCGTAACAAGGTTTCCGTAGGTGAACCTGCAGAAGGATCA
>Haptophrya_schmidtearum_JJ_117_SP
AACCTGGTTGATCCTGCCAGTAGTCATATGCTTGTCTCAAAGATTAAGCCATGCATGTCTAAGTATAATAGTATACAGTAAAACTGCGA
ATGGCTCATTACAACAGTTATAGTTTATTTGATAATGGAAGTCTACATGGATAACCGTGGTAATTCTAGAGCTAATACATGCTGTTAAA

CCTAACTTTACGGAAGGGTTGTATTTATTAGATATTAAGCCAATATTCTTTCGGGATTATTGCGAGGAATCATAATAACTGATCGAATC
TCAAATTTGAGATAAATCATTCAAGTTTCTGCCCTATCAGCTTTCGATGGTAGTGTATTGGACTACCATGGCAGTCACGGGTAACGGAG
AATTAGGGTTCGGTTCCGGAGAGGGAGCCTGAGAAACGGCTACCACATCTAAGGAAGGCAGCAGGCGCGTAAATTACCCAATCCTGATT
CAGGGAGGTAGTGACAAGAAATAACAACTCGGGGATTTTTAATCTTACGGGATTGCAATGAGAACAATTTAAACCACTTAGCGAGGAAC
AATTGGAGGGCAAGTCTGGTGCCAGCAGCCGCGGTAATTCCAGCTCCAATAGCGTATATTAAAGTTGTTGCAGTTAAAAAGCTCGTAGT
TGAATTTCTGGCTTAATTTAATCGCGGCTTCGGTCAGGTTAAAATAAGTCATCCGTTTGCAAACCATTTTCGTCCTTCACAGGTTCGAT
TTGGGGAGTAAACCTTTTACTTTGAAAAAATTAGAGTGTTTCAGGCAGGTTATAGCCCGAATACATTAGCATGGAATAATGGAATAGGA
CTTTTGTCCATTTGGTTGGTTATTGGACATAAGTAATGATTAATAGGGACAGTTGGGGGCATTAGTATTTAATTGTCAGAGGTGAAATT
CTTGGATTTATTAAAGACTAACTTATGCGAAAGCATTTGCCAAGGATGTTTTCATTAATCAAGAACGAAAGTTAAGGGATCAAATACGA
TCAGATACCGTAGTAGTCTTAACTATAAACTATACCGACTCGGGATTGGATGGGTATATGCCCATTCAGCACCGTATGAGAAATCAAAG
TCTTTGGGTTCTGGGGGGAGTATGGTCGCAAGGCTGAAACTTAAAGGAATTGACGGAAGGGCACCACCAGGAGTGGAGCCTGCGGCTTA
ATTTGACTCAACACGGGGAAACTTACCAGGTCAAAACATGGGTGGGATTGACAGATTGAGAGCTCTTTCTTGATTCTATGGGTGGTGGT
GCATGGCCGTTCTTAGTTGGTGGAGTGATTTGTCTGGTTAATTCCGTTAACGAACGAGACCTTAACCTGCTAACTAGTCTTTTAGTATA
-TTACTAAAAGTATTTCTTAGAGGGACTATGCGATGAAACGCATGGAAGTTTGAGGCAATAACAGGTCTGTGATGCCCTTAGATGTCCT
GGGCCGCACGTGCGCTACAATGACACGTTCAACAAGTTATTTCCTGGCCCGGAAGGGTACGGGTAATCTTTTTAATGCGTGTCGTGTTA
GGGATAGATCTTTGGAATTATAGATCTTGAACGAGGAATTCCTAGTAAGTGCAAGTCATCAACTTGTACTGATTACGTCCCTGCCCTTT
GTACACACCGCCCGTCGCTCCTACCGATTTCGAGTGATCCGGTGAACCTTCTGGACTGGGTCGTCCTTGAGACGGACTTGGGAAGTTAA
GTAAACCTTATCACTTAGAGGAAGGAGAAGTCGTAACAAGGTTTCCGTAGGTGAACCTGCAGAAGGATCA

## 16S rRNA gene alignment

```
>Haptophrya_planariarum_KD_1_DG
TTACTCGTCGTTACTGGGCGTATAGGATACGTAGATGGTTCTAAACATGCTA--T-TTACTG-AAACGATTTTGTTAGGGGGTGGTAAT
TTAGGTACTTGGTTTAGTTAAGGGCGGCACACAGGTGTATGTTAGCGATAAAATGCAGAAAATATATGCTGGTTTCCGAAAGTGAAAAC
ATGCCGCTATAACTAATCGACATTGAGGTATGAAGGTATGGGTGTCGATCGGGATTAGAGACCCCAGTAGTCCATACTACAAAAGATGA
GTATTCCAATAGCACAAA-CTTGGAAGCTAACGCGATAAATACTCCGCCTGGGGAGTACGGCCGCAAGGTTAAAACTTAAGAGATTTGG
CGGGAATTTGTTCGAACGGTGGAACATGTGGTTTAATTCGATAATCCACGAAAAATCTTACCAGTGTTGGAATACACTAGAATCGAAGG
AGTAACGTCATAGATCGATTTTTTAGTGTTAACACGGTGTTGCATGGCTGTCGTCAGTTCGTGCTGTGAAGTGTAGAGTTAAGTCTTAT
AAACGAACAAAATCTCTCGAATTT-TGTGGTTAGGTTGT-TT--TATTTTTTTGCAACTTAATTTTAAAAATTTGAT-ATAAATTGT-T
TGGTG--T-AAGAT--TTGGAAAGT-TA-TTTTTACTTTCC----GATTTATTTATCGTC---G-ATA--T-T-T-GC-T--TATTT-T
TTTTGCA-AAAAAGGGAC-CGAACATCTAC--TTGCTTGTCTAGTAGGTGTTTGTTTTTTTTTTTGTAAATATTA-ATAAGGAT-GCGT
-TATTGGTTTGATA-AGTTTTTCATAA-ATAATTTATTAGAATATAGAGCTGAAGTCAAGTCCTTATGGCCTGTATATGCTGGGCTACA
CACGTGTTACAAGGGTAGAGACAGAGAGTAGCAAGTGTGCGAACAGGAGCAAGCCTCTAAAAATTACCTTAGTTCGGATTGTTTTATGT
AACTCTAAAGCATGAAGTTGAAATCGTTAGTAATTACACTTGAGTATGGTGTAGTGAATGAAAAGTCAAATTTTGCACACACTGCCCAT
CACGCCCGAAGAATTGGTCCGGAGGGAAGGT-G-CCTTGATCGCTGTGGTG-AGTTGT-AGTAA-AACAAGTCCCCTGCTATAATTAGT
CA-TTTACGGATGGCGGTCTTTGTTCATCGAAGTCAGTTTGTATTTGTGTAATTGACACAAGG
>Haptophrya_planariarum_KD_2_DG
TTACTCGTCGTTACTGGGCGTATAGGATACGTAGATGGTTCTAAACATGCTA--T-TTACTG-AAACGATTTTGTTAGGGGGTGGTAAT
TTAGGTACTTGGTTTAGTTAAGGGCGGCACACAGGTGTATGTTAGCGATAAAATGCAGAAAATATATGCTGGTTTCCGAAAGTGAAAAC
ATGCCGCTATAACTAATCGACATTGAGGTATGAAGGTATGGGTGTCGATCGGGATTAGAGACCCCAGTAGTCCATACTACAAAAGATGA
GTATTCCAATAGCACAAA-CTTGGAAGCTAACGCGATAAATACTCCGCCTGGGGAGTACGGCCGCAAGGTTAAAACTTAAGAGATTTGG
CGGGAATTTGTTCGAACGGTGGAACATGTGGTTTAATTCGATAATCCACGAAAAATCTTACCAGTGTTGGAATACACTAGAATCGAAGG
AGTAACGTCATAGATCGATTTTTTAGTGTTAACACGGTGTTGCATGGCTGTCGTCAGTTCGTGCTGTGAAGTGTAGAGTTAAGTCTTAT
AAACGAACAAAATCTCTCGAATTT-TGTGGTTAGGTTGT-TT--TATTTTTTTGCAACTTAATTTTAAAAATTTGAT-ATAAATTGT-T
TGGTG--T-AAGAT--TTGGAAAGT-TA-TTTTTACTTTCC----GATTTATTTATCGTC---G-ATA--T-T-T-GC-T--TATTT-T
TTTTGCA-AAAAAGGGAC-CGAACATCTAC--TTGCTTGTCTAGTAGGTGTTTGTTTTTTTTTTTGTAAATATTA-ATAAGGAT-GCGT
-TATTGGTTTGATA-AGTTTTTCATAA-ATAATTTATTAGAATATAGAGCTGAAGTCAAGTCCTTATGGCCTGTATATGCTGGGCTACA
CACGTGTTACAAGGGTAGAGACAGAGAGTAGCAAGTGTGCGAACAGGAGCAAGCCTCTAAAAATTACCTTAGTTCGGATTGTTTTATGT
AACTCTAAAGCATGAAGTTGAAATCGTTAGTAATTACACTTGAGTATGGTGTAGTGAATGAAAAGTCAAATTTTGCACACACTGCCCAT
CACGCCCGAAGAATTGGTCCGGAGGGAAGGT-G-CCTTGATCGCTGTGGTG-AGTTGT-AGTAA-AACAAGTCCCCTGCTATAATTAGT
CA-TTTACGGATGGCGGTCTTTGTTCATCGAAGTCAGTTTGTATTTGTGTAATTGACACAAGG
>Haptophrya_planariarum_KD_3_DG
TTACTCGTCGTTACTGGGCGTATAGGATACGTAGATGGTTCTAAACATGCTA--T-TTACTG-AAACGATTTTGTTAGGGGGTGGTAAT
TTAGGTACTTGGTTTAGTTAAGGGCGGCACACAGGTGTATGTTAGCGATAAAATGCAGAAAATATATGCTGGTTTCCGAAAGTGAAAAC
ATGCCGCTATAACTAATCGACATTGAGGTATGAAGGTATGGGTGTCGATCGGGATTAGAGACCCCAGTAGTCCATACTACAAAAGATGA
GTATTCCAATAGCACAAA-CTTGGAAGCTAACGCGATAAATACTCCGCCTGGGGAGTACGGCCGCAAGGTTAAAACTTAAGAGATTTGG
CGGGAATTTGTTCGAACGGTGGAACATGTGGTTTAATTCGATAATCCACGAAAAATCTTACCAGTGTTGGAATACACTAGAATCGAAGG
AGTAACGTCATAGATCGATTTTTTAGTGTTAACACGGTGTTGCATGGCTGTCGTCAGTTCGTGCTGTGAAGTGTAGAGTTAAGTCTTAT
AAACGAACAAAATCTCTCGAATTT-TGTGGTTAGGTTGT-TT--TATTTTTTTGCAACTTAATTTTAAAAATTTGAT-ATAAATTGT-T
TGGTG--T-AAGAT--TTGGAAAGT-TA-TTTTTACTTTCC----GATTTATTTATCGTC---G-ATA--T-T-T-GC-T--TATTT-T
TTTTGCA-AAAAAGGGAC-CGAACATCTAC--TTGCTTGTCTAGTAGGTGTTTGTTTTTTTTTTTGTAAATATTA-ATAAGGAT-GCGT
-TATTGGTTTGATA-AGTTTTTCATAA-ATAATTTATTAGAATATAGAGCTGAAGTCAAGTCCTTATGGCCTGTATATGCTGGGCTACA
CACGTGTTACAAGGGTAGAGACAGAGAGTAGCAAGTGTGCGAACAGGAGCAAGCCTCTAAAAATTACCTTAGTTCGGATTGTTTTATGT
AACTCTAAAGCATGAAGTTGAAATCGTTAGTAATTACACTTGAGTATGGTGTAGTGAATGAAAAGTCAAATTTTGCACACACTGCCCAT
CACGCCCGAAGAATTGGTCCGGAGGGAAGGT-G-CCTTGATCGCTGTGGTG-AGTTGT-AGTAA-AACAAGTCCCCTGCTATAATTAGT
CA-TTTACGGATGGCGGTCTTTGTTCATCGAAGTCAGTTTGTATTTGTGTAATTGACACAAGG
>Haptophrya_planariarum_OL_4_DG
TTACTCGTCGTTACTGGGCGTATAGGATACGTAGATGGTTCTAAACATGCTA--T-TTACTG-AAACGATTTTGTTAGGGGGTGGTAAT
TTAGGTACTTGGTTTAGTTAAGGGCGGCACACAGGTGTATGTTAGCGATAAAATGCAGAAAATATATGCTGGTTTCCGAAAGTGAAAAC
ATGCCGCTATAACTAATCGACATTGAGGTATGAAGGTATGGGTGTCGATCGGGATTAGAGACCCCAGTAGTCCATACTACAAAAGATGA
GTATTCCAATAGCACAAA-CTTGGAAGCTAACGCGATAAATACTCCGCCTGGGGAGTACGGCCGCAAGGTTAAAACTTAAGAGATTTGG
CGGGAATTTGTTCGAACGGTGGAACATGTGGTTTAATTCGATAATCCACGAAAAATCTTACCAGTGTTGGAATACACTAGAATCGAAGG
AGTAACGTCATAGATCGATTTTTTAGTGTTAACACGGTGTTGCATGGCTGTCGTCAGTTCGTGCTGTGAAGTGTAGAGTTAAGTCTTAT
AAACGAACAAAATCTCTCGAATTT-TGTGGTTAGGTTGT-TT--TATTTTTTTGCAACTTAATTTTAAAAATTTGAT-ATAAATTGT-T
TGGTG--T-AAGAT--TTGGAAAGT-TA-TTTTTACTTTCC----GATTTATTTATCGTC---G-ATA--T-T-T-GC-T--TATTT-T
TTTTGCA-AAAAAGGGAC-CGAACATCTAC--TTGCTTGTCTAGTAGGTGTTTGTTTTTTTTTTTGTAAATATTA-ATAAGGAT-GCGT
-TATTGGTTTGATA-AGTTTTTCATAA-ATAATTTATTAGAATATAGAGCTGAAGTCAAGTCCTTATGGCCTGTATATGCTGGGCTACA
CACGTGTTACAAGGGTAGAGACAGAGAGTAGCAAGTGTGCGAACAGGAGCAAGCCTCTAAAAATTACCTTAGTTCGGATTGTTTTATGT
AACTCTAAAGCATGAAGTTGAAATCGTTAGTAATTACACTTGAGTATGGTGTAGTGAATGAAAAGTCAAATTTTGCACACACTGCCCAT
CACGCCCGAAGAATTGGTCCGGAGGGAAGGT-G-CCTTGATCGCTGTGGTG-AGTTGT-AGTAA-AACAAGTCCCCTGCTATAATTAGT
CA-TTTACGGATGGCGGTCTTTGTTCATCGAAGTCAGTTTGTATTTGTGTAATTGACACAAGG
>Haptophrya_planariarum_OL_6_DG
TTACTCGTCGTTACTGGGCGTATAGGATACGTAGATGGTTCTAAACATGCTA--T-TTACTG-AAACGATTTTGTTAGGGGGTGGTAAT
TTAGGTACTTGGTTTAGTTAAGGGCGGCACACAGGTGTATGTTAGCGATAAAATGCAGAAAATATATGCTGGTTTCCGAAAGTGAAAAC
ATGCCGCTATAACTAATCGACATTGAGGTATGAAGGTATGGGTGTCGATCGGGATTAGAGACCCCAGTAGTCCATACTACAAAAGATGA
GTATTCCAATAGCACAAA-CTTGGAAGCTAACGCGATAAATACTCCGCCTGGGGAGTACGGCCGCAAGGTTAAAACTTAAGAGATTTGG
CGGGAATTTGTTCGAACGGTGGAACATGTGGTTTAATTCGATAATCCACGAAAAATCTTACCAGTGTTGGAATACACTAGAATCGAAGG
AGTAACGTCATAGATCGATTTTTTAGTGTTAACACGGTGTTGCATGGCTGTCGTCAGTTCGTGCTGTGAAGTGTAGAGTTAAGTCTTAT
AAACGAACAAAATCTCTCGAATTT-TGTGGTTAGGTTGT-TT--TATTTTTTTGCAACTTAATTTTAAAAATTTGAT-ATAAATTGT-T
TGGTG--T-AAGAT--TTGGAAAGT-TA-TTTTTACTTTCC----GATTTATTTATCGTC---G-ATA--T-T-T-GC-T--TATTT-T
```

TTTTGCA-AAAAAGGGAC-CGAACATCTAC--TTGCTTGTCTAGTAGGTGTTTGTTTTTTTTTTTGTAAATATTA-ATAAGGAT-GCGT
-TATTGGTTTGATA-AGTTTTTCATAA-ATAATTTATTAGAATATAGAGCTGAAGTCAAGTCCTTATGGCCTGTATATGCTGGGCTACA
CACGTGTTACAAGGGTAGAGACAGAGAGTAGCAAGTGTGCGAACAGGAGCAAGCCTCTAAAAATTACCTTAGTTCGGATTGTTTTATGT
AACTCTAAAGCATGAAGTTGAAATCGTTAGTAATTACACTTGAGTATGGTGTAGTGAATGAAAAGTCAAATTTTGCACACACTGCCCAT
CACGCCCGAAGAATTGGTCCGGAGGGAAGGT-G-CCTTGATCGCTGTGGTG-AGTTGT-AGTAA-AACAAGTCCCCTGCTATAATTAGT
CA-TTTACGGATGGCGGTCTTTGTTCATCGAAGTCAGTTTGTATTTGTGTAATTGACACAAGG
>Haptophrya_planariarum_OL_7_DG
TTACTCGTCGTTACTGGGCGTATAGGATACGTAGATGGTTCTAAACATGCTA--T-TTACTG-AAACGATTTTGTTAGGGGGTGGTAAT
TTAGGTACTTGGTTTAGTTAAGGGCGGCACACAGGTGTATGTTAGCGATAAAATGCAGAAAATATATGCTGGTTTCCGAAAGTGAAAAC
ATGCCGCTATAACTAATCGACATTGAGGTATGAAGGTATGGGTGTCGATCGGGATTAGAGACCCCAGTAGTCCATACTACAAAAGATGA
GTATTCCAATAGCACAAA-CTTGGAAGCTAACGCGATAAATACTCCGCCTGGGGAGTACGGCCGCAAGGTTAAAACTTAAGAGATTTGG
CGGGAATTTGTTCGAACGGTGGAACATGTGGTTTAATTCGATAATCCACGAAAAATCTTACCAGTGTTGGAATACACTAGAATCGAAGG
AGTAACGTCATAGATCGATTTTTTAGTGTTAACACGGTGTTGCATGGCTGTCGTCAGTTCGTGCTGTGAAGTGTAGAGTTAAGTCTTAT
AAACGAACAAAATCTCTCGAATTT-TGTGGTTAGGTTGT-TT--TATTTTTTTGCAACTTAATTTTAAAAATTTGAT-ATAAATTGT-T
TGGTG--T-AAGAT--TTGGAAAGT-TA-TTTTTACTTTCC----GATTTATTTATCGTC---G-ATA--T-T-T-GC-T--TATTT-T
TTTTGCA-AAAAAGGGAC-CGAACATCTAC--TTGCTTGTCTAGTAGGTGTTTGTTTTTTTTTTTGTAAATATTA-ATAAGGAT-GCGT
-TATTGGTTTGATA-AGTTTTTCATAA-ATAATTTATTAGAATATAGAGCTGAAGTCAAGTCCTTATGGCCTGTATATGCTGGGCTACA
CACGTGTTACAAGGGTAGAGACAGAGAGTAGCAAGTGTGCGAACAGGAGCAAGCCTCTAAAAATTACCTTAGTTCGGATTGTTTTATGT
AACTCTAAAGCATGAAGTTGAAATCGTTAGTAATTACACTTGAGTATGGTGTAGTGAATGAAAAGTCAAATTTTGCACACACTGCCCAT
CACGCCCGAAGAATTGGTCCGGAGGGAAGGT-G-CCTTGATCGCTGTGGTG-AGTTGT-AGTAA-AACAAGTCCCCTGCTATAATTAGT
CA-TTTACGGATGGCGGTCTTTGTTCATCGAAGTCAGTTTGTATTTGTGTAATTGACACAAGG
>Haptophrya_planariarum_OL_8_DG
TTACTCGTCGTTACTGGGCGTATAGGATACGTAGATGGTTCTAAACATGCTA--T-TTACTG-AAACGATTTTGTTAGGGGGTGGTAAT
TTAGGTACTTGGTTTAGTTAAGGGCGGCACACAGGTGTATGTTAGCGATAAAATGCAGAAAATATATGCTGGTTTCCGAAAGTGAAAAC
ATGCCGCTATAACTAATCGACATTGAGGTATGAAGGTATGGGTGTCGATCGGGATTAGAGACCCCAGTAGTCCATACTACAAAAGATGA
GTATTCCAATAGCACAAA-CTTGGAAGCTAACGCGATAAATACTCCGCCTGGGGAGTACGGCCGCAAGGTTAAAACTTAAGAGATTTGG
CGGGAATTTGTTCGAACGGTGGAACATGTGGTTTAATTCGATAATCCACGAAAAATCTTACCAGTGTTGGAATACACTAGAATCGAAGG
AGTAACGTCATAGATCGATTTTTTAGTGTTAACACGGTGTTGCATGGCTGTCGTCAGTTCGTGCTGTGAAGTGTAGAGTTAAGTCTTAT
AAACGAACAAAATCTCTCGAATTT-TGTGGTTAGGTTGT-TT--TATTTTTTTGCAACTTAATTTTAAAAATTTGAT-ATAAATTGT-T
TGGTG--T-AAGAT--TTGGAAAGT-TA-TTTTTACTTTCC----GATTTATTTATCGTC---G-ATA--T-T-T-GC-T--TATTT-T
TTTTGCA-AAAAAGGGAC-CGAACATCTAC--TTGCTTGTCTAGTAGGTGTTTGTTTTTTTTTTTGTAAATATTA-ATAAGGAT-GCGT
-TATTGGTTTGATA-AGTTTTTCATAA-ATAATTTATTAGAATATAGAGCTGAAGTCAAGTCCTTATGGCCTGTATATGCTGGGCTACA
CACGTGTTACAAGGGTAGAGACAGAGAGTAGCAAGTGTGCGAACAGGAGCAAGCCTCTAAAAATTACCTTAGTTCGGATTGTTTTATGT
AACTCTAAAGCATGAAGTTGAAATCGTTAGTAATTACACTTGAGTATGGTGTAGTGAATGAAAAGTCAAATTTTGCACACACTGCCCAT
CACGCCCGAAGAATTGGTCCGGAGGGAAGGT-G-CCTTGATCGCTGTGGTG-AGTTGT-AGTAA-AACAAGTCCCCTGCTATAATTAGT
CA-TTTACGGATGGCGGTCTTTGTTCATCGAAGTCAGTTTGTATTTGTGTAATTGACACAAGG
>Haptophrya_planariarum_OL_9_DG
TTACTCGTCGTTACTGGGCGTATAGGATACGTAGATGGTTCTAAACATGCTA--T-TTACTG-AAACGATTTTGTTAGGGGGTGGTAAT
TTAGGTACTTGGTTTAGTTAAGGGCGGCACACAGGTGTATGTTAGCGATAAAATGCAGAAAATATATGCTGGTTTCCGAAAGTGAAAAC
ATGCCGCTATAACTAATCGACATTGAGGTATGAAGGTATGGGTGTCGATCGGGATTAGAGACCCCAGTAGTCCATACTACAAAAGATGA
GTATTCCAATAGCACAAA-CTTGGAAGCTAACGCGATAAATACTCCGCCTGGGGAGTACGGCCGCAAGGTTAAAACTTAAGAGATTTGG
CGGGAATTTGTTCGAACGGTGGAACATGTGGTTTAATTCGATAATCCACGAAAAATCTTACCAGTGTTGGAATACACTAGAATCGAAGG
AGTAACGTCATAGATCGATTTTTTAGTGTTAACACGGTGTTGCATGGCTGTCGTCAGTTCGTGCTGTGAAGTGTAGAGTTAAGTCTTAT
AAACGAACAAAATCTCTCGAATTT-TGTGGTTAGGTTGT-TT--TATTTTTTTGCAACTTAATTTTAAAAATTTGAT-ATAAATTGT-T
TGGTG--T-AAGAT--TTGGAAAGT-TA-TTTTTACTTTCC----GATTTATTTATCGTC---G-ATA--T-T-T-GC-T--TATTT-T
TTTTGCA-AAAAAGGGAC-CGAACATCTAC--TTGCTTGTCTAGTAGGTGTTTGTTTTTTTTTTTGTAAATATTA-ATAAGGAT-GCGT
-TATTGGTTTGATA-AGTTTTTCATAA-ATAATTTATTAGAATATAGAGCTGAAGTCAAGTCCTTATGGCCTGTATATGCTGGGCTACA
CACGTGTTACAAGGGTAGAGACAGAGAGTAGCAAGTGTGCGAACAGGAGCAAGCCTCTAAAAATTACCTTAGTTCGGATTGTTTTATGT
AACTCTAAAGCATGAAGTTGAAATCGTTAGTAATTACACTTGAGTATGGTGTAGTGAATGAAAAGTCAAATTTTGCACACACTGCCCAT
CACGCCCGAAGAATTGGTCCGGAGGGAAGGT-G-CCTTGATCGCTGTGGTG-AGTTGT-AGTAA-AACAAGTCCCCTGCTATAATTAGT
CA-TTTACGGATGGCGGTCTTTGTTCATCGAAGTCAGTTTGTATTTGTGTAATTGACACAAGG
>Haptophrya_planariarum_OL_11_DG
TTACTCGTCGTTACTGGGCGTATAGGATACGTAGATGGTTCTAAACATGCTA--T-TTACTG-AAACGATTTTGTTAGGGGGTGGTAAT
TTAGGTACTTGGTTTAGTTAAGGGCGGCACACAGGTGTATGTTAGCGATAAAATGCAGAAAATATATGCTGGTTTCCGAAAGTGAAAAC
ATGCCGCTATAACTAATCGACATTGAGGTATGAAGGTATGGGTGTCGATCGGGATTAGAGACCCCAGTAGTCCATACTACAAAAGATGA
GTATTCCAATAGCACAAA-CTTGGAAGCTAACGCGATAAATACTCCGCCTGGGGAGTACGGCCGCAAGGTTAAAACTTAAGAGATTTGG
CGGGAATTTGTTCGAACGGTGGAACATGTGGTTTAATTCGATAATCCACGAAAAATCTTACCAGTGTTGGAATACACTAGAATCGAAGG
AGTAACGTCATAGATCGATTTTTTAGTGTTAACACGGTGTTGCATGGCTGTCGTCAGTTCGTGCTGTGAAGTGTAGAGTTAAGTCTTAT
AAACGAACAAAATCTCTCGAATTT-TGTGGTTAGGTTGT-TT--TATTTTTTTGCAACTTAATTTTAAAAATTTGAT-ATAAATTGT-T
TGGTG--T-AAGAT--TTGGAAAGT-TA-TTTTTACTTTCC----GATTTATTTATCGTC---G-ATA--T-T-T-GC-T--TATTT-T
TTTTGCA-AAAAAGGGAC-CGAACATCTAC--TTGCTTGTCTAGTAGGTGTTTGTTTTTTTTTTTGTAAATATTA-ATAAGGAT-GCGT
-TATTGGTTTGATA-AGTTTTTCATAA-ATAATTTATTAGAATATAGAGCTGAAGTCAAGTCCTTATGGCCTGTATATGCTGGGCTACA
CACGTGTTACAAGGGTAGAGACAGAGAGTAGCAAGTGTGCGAACAGGAGCAAGCCTCTAAAAATTACCTTAGTTCGGATTGTTTTATGT
AACTCTAAAGCATGAAGTTGAAATCGTTAGTAATTACACTTGAGTATGGTGTAGTGAATGAAAAGTCAAATTTTGCACACACTGCCCAT
CACGCCCGAAGAATTGGTCCGGAGGGAAGGT-G-CCTTGATCGCTGTGGTG-AGTTGT-AGTAA-AACAAGTCCCCTGCTATAATTAGT
CA-TTTACGGATGGCGGTCTTTGTTCATCGAAGTCAGTTTGTATTTGTGTAATTGACACAAGG
>Haptophrya_planariarum_MI_12_DG
TTACTCGTCGTTACTGGGCGTATAGGATACGTAGATGGTTCTAAACATGCTA--T-TTACTG-AAACGATTTTGTTAGGGGGTGGTAAT
TTAGGTACTTGGTTTAGTTAAGGGCGGCACACAGGTGTATGTTAGCGATAAAATGCAGAAAATATATGCTGGTTTCCGAAAGTGAAAAC
ATGCCGCTATAACTAATCGACATTGAGGTATGAAGGTATGGGTGTCGATCGGGATTAGAGACCCCAGTAGTCCATACTACAAAAGATGA
GTATTCCAATAGCACAAA-CTTGGAAGCTAACGCGATAAATACTCCGCCTGGGGAGTACGGCCGCAAGGTTAAAACTTAAGAGATTTGG

CGGGAATTTGTTCGAACGGTGGAACATGTGGTTTAATTCGATAATCCACGAAAAATCTTACCAGTGTTGGAATACACTAGAATCGAAGG
AGTAACGTCATAGATCGATTTTTTAGTGTTAACACGGTGTTGCATGGCTGTCGTCAGTTCGTGCTGTGAAGTGTAGAGTTAAGTCTTAT
AAACGAACAAAATCTCTCGAATTT-TGTGGTTAGGTTGT-TT--TATTTTTTTTGCAACTTAATTTTAAAAATTTGAT-ATAAATTGT-T
TGGTG--T-AAGAT--TTGGAAAGT-TA-TTTTTACTTTCC----GATTTATTTATCGTC---G-ATA--T-T-T-GC-T--TATTT-T
TTTTGCA-AAAAAGGGAC-CGAACATCTAC--TTGCTTGTCTAGTAGGTGTTTGTTTTTTTTTTTGTAAATATTA-ATAAGGAT-GCGT
-TATTGGTTTGATA-AGTTTTTCATAA-ATAATTTATTAGAATATAGAGCTGAAGTCAAGTCCTTATGGCCTGTATATGCTGGGCTACA
CACGTGTTACAAGGGTAGAGACAGAGAGTAGCAAGTGTGCGAACAGGAGCAAGCCTCTAAAAATTACCTTAGTTCGGATTGTTTTATGT
AACTCTAAAGCATGAAGTTGAAATCGTTAGTAATTACACTTGAGTATGGTGTAGTGAATGAAAAGTCAAATTTTGCACACACTGCCCAT
CACGCCCGAAGAATTGGTCCGGAGGGAAGGT-G-CCTTGATCGCTGTGGTG-AGTTGT-AGTAA-AACAAGTCCCCTGCTATAATTAGT
CA-TTTACGGATGGCGGTCTTTGTTCATCGAAGTCAGTTTGTATTTGTGTAATTGACACAAGG
>Haptophrya_planariarum_MI_16_DG
TTACTCGTCGTTACTGGGCGTATAGGATACGTAGATGGTTCTAAACATGCTA--T-TTACTG-AAACGATTTTGTTAGGGGGTGGTAAT
TTAGGTACTTGGTTTAGTTAAGGGCGGCACACAGGTGTATGTTAGCGATAAAATGCAGAAAATATATGCTGGTTTCCGAAAGTGAAAAC
ATGCCGCTATAACTAATCGACATTGAGGTATGAAGGTATGGGTGTCGATCGGGATTAGAGACCCCAGTAGTCCATACTACAAAAGATGA
GTATTCCAATAGCACAAA-CTTGGAAGCTAACGCGATAAATACTCCGCCTGGGGAGTACGGCCGCAAGGTTAAAACTTAAGAGATTTGG
CGGGAATTTGTTCGAACGGTGGAACATGTGGTTTAATTCGATAATCCACGAAAAATCTTACCAGTGTTGGAATACACTAGAATCGAAGG
AGTAACGTCATAGATCGATTTTTTAGTGTTAACACGGTGTTGCATGGCTGTCGTCAGTTCGTGCTGTGAAGTGTAGAGTTAAGTCTTAT
AAACGAACAAAATCTCTCGAATTT-TGTGGTTAGGTTGT-TT--TATTTTTTTTGCAACTTAATTTTAAAAATTTGAT-ATAAATTGT-T
TGGTG--T-AAGAT--TTGGAAAGT-TA-TTTTTACTTTCC----GATTTATTTATCGTC---G-ATA--T-T-T-GC-T--TATTT-T
TTTTGCA-AAAAAGGGAC-CGAACATCTAC--TTGCTTGTCTAGTAGGTGTTTGTTTTTTTTTTTGTAAATATTA-ATAAGGAT-GCGT
-TATTGGTTTGATA-AGTTTTTCATAA-ATAATTTATTAGAATATAGAGCTGAAGTCAAGTCCTTATGGCCTGTATATGCTGGGCTACA
CACGTGTTACAAGGGTAGAGACAGAGAGTAGCAAGTGTGCGAACAGGAGCAAGCCTCTAAAAATTACCTTAGTTCGGATTGTTTTATGT
AACTCTAAAGCATGAAGTTGAAATCGTTAGTAATTACACTTGAGTATGGTGTAGTGAATGAAAAGTCAAATTTTGCACACACTGCCCAT
CACGCCCGAAGAATTGGTCCGGAGGGAAGGT-G-CCTTGATCGCTGTGGTG-AGTTGT-AGTAA-AACAAGTCCCCTGCTATAATTAGT
CA-TTTACGGATGGCGGTCTTTGTTCATCGAAGTCAGTTTGTATTTGTGTAATTGACACAAGG
>Haptophrya_planariarum_DL_19_DG
TTACTCGTCGTTACTGGGCGTATAGGATACGTAGATGGTTCTAAACATGCTA--T-TTACTG-AAACGATTTTGTTAGGGGGTGGTAAT
TTAGGTACTTGGTTTAGTTAAGGGCGGCACACAGGTGTATGTTAGCGATAAAATGCAGAAAATATATGCTGGTTTCCGAAAGTGAAAAC
ATGCCGCTATAACTAATCGACATTGAGGTATGAAGGTATGGGTGTCGATCGGGATTAGAGACCCCAGTAGTCCATACTACAAAAGATGA
GTATTCCAATAGCACAAA-CTTGGAAGCTAACGCGATAAATACTCCGCCTGGGGAGTACGGCCGCAAGGTTAAAACTTAAGAGATTTGG
CGGGAATTTGTTCGAACGGTGGAACATGTGGTTTAATTCGATAATCCACGAAAAATCTTACCAGTGTTGGAATACACTAGAATCGAAGG
AGTAACGTCATAGATCGATTTTTTAGTGTTAACACGGTGTTGCATGGCTGTCGTCAGTTCGTGCTGTGAAGTGTAGAGTTAAGTCTTAT
AAACGAACAAAATCTCTCGAATTT-TGTGGTTAGGTTGT-TT--TATTTTTTTTGCAACTTAATTTTAAAAATTTGAT-ATAAATTGT-T
TGGTG--T-AAGAT--TTGGAAAGT-TA-TTTTTACTTTCC----GATTTATTTATCGTC---G-ATA--T-T-T-GC-T--TATTT-T
TTTTGCA-AAAAAGGGAC-CGAACATCTAC--TTGCTTGTCTAGTAGGTGTTTGTTTTTTTTTTTGTAAATATTA-ATAAGGAT-GCGT
-TATTGGTTTGATA-AGTTTTTCATAA-ATAATTTATTAGAATATAGAGCTGAAGTCAAGTCCTTATGGCCTGTATATGCTGGGCTACA
CACGTGTTACAAGGGTAGAGACAGAGAGTAGCAAGTGTGCGAACAGGAGCAAGCCTCTAAAAATTACCTTAGTTCGGATTGTTTTATGT
AACTCTAAAGCATGAAGTTGAAATCGTTAGTAATTACACTTGAGTATGGTGTAGTGAATGAAAAGTCAAATTTTGCACACACTGCCCAT
CACGCCCGAAGAATTGGTCCGGAGGGAAGGT-G-CCTTGATCGCTGTGGTG-AGTTGT-AGTAA-AACAAGTCCCCTGCTATAATTAGT
CA-TTTACGGATGGCGGTCTTTGTTCATCGAAGTCAGTTTGTATTTGTGTAATTGACACAAGG
>Haptophrya_planariarum_DL_20_DG
TTACTCGTCGTTACTGGGCGTATAGGATACGTAGATGGTTCTAAACATGCTA--T-TTACTG-AAACGATTTTGTTAGGGGGTGGTAAT
TTAGGTACTTGGTTTAGTTAAGGGCGGCACACAGGTGTATGTTAGCGATAAAATGCAGAAAATATATGCTGGTTTCCGAAAGTGAAAAC
ATGCCGCTATAACTAATCGACATTGAGGTATGAAGGTATGGGTGTCGATCGGGATTAGAGACCCCAGTAGTCCATACTACAAAAGATGA
GTATTCCAATAGCACAAA-CTTGGAAGCTAACGCGATAAATACTCCGCCTGGGGAGTACGGCCGCAAGGTTAAAACTTAAGAGATTTGG
CGGGAATTTGTTCGAACGGTGGAACATGTGGTTTAATTCGATAATCCACGAAAAATCTTACCAGTGTTGGAATACACTAGAATCGAAGG
AGTAACGTCATAGATCGATTTTTTAGTGTTAACACGGTGTTGCATGGCTGTCGTCAGTTCGTGCTGTGAAGTGTAGAGTTAAGTCTTAT
AAACGAACAAAATCTCTCGAATTT-TGTGGTTAGGTTGT-TT--TATTTTTTTTGCAACTTAATTTTAAAAATTTGAT-ATAAATTGT-T
TGGTG--T-AAGAT--TTGGAAAGT-TA-TTTTTACTTTCC----GATTTATTTATCGTC---G-ATA--T-T-T-GC-T--TATTT-T
TTTTGCA-AAAAAGGGAC-CGAACATCTAC--TTGCTTGTCTAGTAGGTGTTTGTTTTTTTTTTTGTAAATATTA-ATAAGGAT-GCGT
-TATTGGTTTGATA-AGTTTTTCATAA-ATAATTTATTAGAATATAGAGCTGAAGTCAAGTCCTTATGGCCTGTATATGCTGGGCTACA
CACGTGTTACAAGGGTAGAGACAGAGAGTAGCAAGTGTGCGAACAGGAGCAAGCCTCTAAAAATTACCTTAGTTCGGATTGTTTTATGT
AACTCTAAAGCATGAAGTTGAAATCGTTAGTAATTACACTTGAGTATGGTGTAGTGAATGAAAAGTCAAATTTTGCACACACTGCCCAT
CACGCCCGAAGAATTGGTCCGGAGGGAAGGT-G-CCTTGATCGCTGTGGTG-AGTTGT-AGTAA-AACAAGTCCCCTGCTATAATTAGT
CA-TTTACGGATGGCGGTCTTTGTTCATCGAAGTCAGTTTGTATTTGTGTAATTGACACAAGG
>Haptophrya_planariarum_DL_21_DG
TTACTCGTCGTTACTGGGCGTATAGGATACGTAGATGGTTCTAAACATGCTA--T-TTACTG-AAACGATTTTGTTAGGGGGTGGTAAT
TTAGGTACTTGGTTTAGTTAAGGGCGGCACACAGGTGTATGTTAGCGATAAAATGCAGAAAATATATGCTGGTTTCCGAAAGTGAAAAC
ATGCCGCTATAACTAATCGACATTGAGGTATGAAGGTATGGGTGTCGATCGGGATTAGAGACCCCAGTAGTCCATACTACAAAAGATGA
GTATTCCAATAGCACAAA-CTTGGAAGCTAACGCGATAAATACTCCGCCTGGGGAGTACGGCCGCAAGGTTAAAACTTAAGAGATTTGG
CGGGAATTTGTTCGAACGGTGGAACATGTGGTTTAATTCGATAATCCACGAAAAATCTTACCAGTGTTGGAATACACTAGAATCGAAGG
AGTAACGTCATAGATCGATTTTTTAGTGTTAACACGGTGTTGCATGGCTGTCGTCAGTTCGTGCTGTGAAGTGTAGAGTTAAGTCTTAT
AAACGAACAAAATCTCTCGAATTT-TGTGGTTAGGTTGT-TT--TATTTTTTTTGCAACTTAATTTTAAAAATTTGAT-ATAAATTGT-T
TGGTG--T-AAGAT--TTGGAAAGT-TA-TTTTTACTTTCC----GATTTATTTATCGTC---G-ATA--T-T-T-GC-T--TATTT-T
TTTTGCA-AAAAAGGGAC-CGAACATCTAC--TTGCTTGTCTAGTAGGTGTTTGTTTTTTTTTTTGTAAATATTA-ATAAGGAT-GCGT
-TATTGGTTTGATA-AGTTTTTCATAA-ATAATTTATTAGAATATAGAGCTGAAGTCAAGTCCTTATGGCCTGTATATGCTGGGCTACA
CACGTGTTACAAGGGTAGAGACAGAGAGTAGCAAGTGTGCGAACAGGAGCAAGCCTCTAAAAATTACCTTAGTTCGGATTGTTTTATGT
AACTCTAAAGCATGAAGTTGAAATCGTTAGTAATTACACTTGAGTATGGTGTAGTGAATGAAAAGTCAAATTTTGCACACACTGCCCAT
CACGCCCGAAGAATTGGTCCGGAGGGAAGGT-G-CCTTGATCGCTGTGGTG-AGTTGT-AGTAA-AACAAGTCCCCTGCTATAATTAGT
CA-TTTACGGATGGCGGTCTTTGTTCATCGAAGTCAGTTTGTATTTGTGTAATTGACACAAGG

>Haptophrya_planariarum_DL_22_DG
TTACTCGTCGTTACTGGGCGTATAGGATACGTAGATGGTTCTAAACATGCTA--T-TTACTG-AAACGATTTTGTTAGGGGGTGGTAAT
TTAGGTACTTGGTTTAGTTAAGGGCGGCACACAGGTGTATGTTAGCGATAAAATGCAGAAAATATATGCTGGTTTCCGAAAGTGAAAAC
ATGCCGCTATAACTAATCGACATTGAGGTATGAAGGTATGGGTGTCGATCGGGATTAGAGACCCCAGTAGTCCATACTACAAAAGATGA
GTATTCCAATAGCACAAA-CTTGGAAGCTAACGCGATAAATACTCCGCCTGGGGAGTACGGCCGCAAGGTTAAAACTTAAGAGATTTGG
CGGGAATTTGTTCGAACGGTGGAACATGTGGTTTAATTCGATAATCCACGAAAAATCTTACCAGTGTTGGAATACACTAGAATCGAAGG
AGTAACGTCATAGATCGATTTTTTAGTGTTAACACGGTGTTGCATGGCTGTCGTCAGTTCGTGCTGTGAAGTGTAGAGTTAAGTCTTAT
AAACGAACAAAATCTCTCGAATTT-TGTGGTTAGGTTGT-TT--TATTTTTTTGCAACTTAATTTTAAAAATTTGAT-ATAAATTGT-T
TGGTG--T-AAGAT--TTGGAAAGT-TA-TTTTTACTTTCC----GATTTATTTATCGTC---G-ATA--T-T-T-GC-T--TATTT-T
TTTTGCA-AAAAAGGGAC-CGAACATCTAC--TTGCTTGTCTAGTAGGTGTTTGTTTTTTTTTTTGTAAATATTA-ATAAGGAT-GCGT
-TATTGGTTTGATA-AGTTTTTCATAA-ATAATTTATTAGAATATAGAGCTGAAGTCAAGTCCTTATGGCCTGTATATGCTGGGCTACA
CACGTGTTACAAGGGTAGAGACAGAGAGTAGCAAGTGTGCGAACAGGAGCAAGCCTCTAAAAATTACCTTAGTTCGGATTGTTTTATGT
AACTCTAAAGCATGAAGTTGAAATCGTTAGTAATTACACTTGAGTATGGTGTAGTGAATGAAAAGTCAAATTTTGCACACACTGCCCAT
CACGCCCGAAGAATTGGTCCGGAGGGAAGGT-G-CCTTGATCGCTGTGGTG-AGTTGT-AGTAA-AACAAGTCCCCTGCTATAATTAGT
CA-TTTACGGATGGCGGTCTTTGTTCATCGAAGTCAGTTTGTATTTGTGTAATTGACACAAGG
>Haptophrya_planariarum_DL_23_DG
TTACTCGTCGTTACTGGGCGTATAGGATACGTAGATGGTTCTAAACATGCTA--T-TTACTG-AAACGATTTTGTTAGGGGGTGGTAAT
TTAGGTACTTGGTTTAGTTAAGGGCGGCACACAGGTGTATGTTAGCGATAAAATGCAGAAAATATATGCTGGTTTCCGAAAGTGAAAAC
ATGCCGCTATAACTAATCGACATTGAGGTATGAAGGTATGGGTGTCGATCGGGATTAGAGACCCCAGTAGTCCATACTACAAAAGATGA
GTATTCCAATAGCACAAA-CTTGGAAGCTAACGCGATAAATACTCCGCCTGGGGAGTACGGCCGCAAGGTTAAAACTTAAGAGATTTGG
CGGGAATTTGTTCGAACGGTGGAACATGTGGTTTAATTCGATAATCCACGAAAAATCTTACCAGTGTTGGAATACACTAGAATCGAAGG
AGTAACGTCATAGATCGATTTTTTAGTGTTAACACGGTGTTGCATGGCTGTCGTCAGTTCGTGCTGTGAAGTGTAGAGTTAAGTCTTAT
AAACGAACAAAATCTCTCGAATTT-TGTGGTTAGGTTGT-TT--TATTTTTTTGCAACTTAATTTTAAAAATTTGAT-ATAAATTGT-T
TGGTG--T-AAGAT--TTGGAAAGT-TA-TTTTTACTTTCC----GATTTATTTATCGTC---G-ATA--T-T-T-GC-T--TATTT-T
TTTTGCA-AAAAAGGGAC-CGAACATCTAC--TTGCTTGTCTAGTAGGTGTTTGTTTTTTTTTTTGTAAATATTA-ATAAGGAT-GCGT
-TATTGGTTTGATA-AGTTTTTCATAA-ATAATTTATTAGAATATAGAGCTGAAGTCAAGTCCTTATGGCCTGTATATGCTGGGCTACA
CACGTGTTACAAGGGTAGAGACAGAGAGTAGCAAGTGTGCGAACAGGAGCAAGCCTCTAAAAATTACCTTAGTTCGGATTGTTTTATGT
AACTCTAAAGCATGAAGTTGAAATCGTTAGTAATTACACTTGAGTATGGTGTAGTGAATGAAAAGTCAAATTTTGCACACACTGCCCAT
CACGCCCGAAGAATTGGTCCGGAGGGAAGGT-G-CCTTGATCGCTGTGGTG-AGTTGT-AGTAA-AACAAGTCCCCTGCTATAATTAGT
CA-TTTACGGATGGCGGTCTTTGTTCATCGAAGTCAGTTTGTATTTGTGTAATTGACACAAGG
>Haptophrya_planariarum_DL_24_DG
TTACTCGTCGTTACTGGGCGTATAGGATACGTAGATGGTTCTAAACATGCTA--T-TTACTG-AAACGATTTTGTTAGGGGGTGGTAAT
TTAGGTACTTGGTTTAGTTAAGGGCGGCACACAGGTGTATGTTAGCGATAAAATGCAGAAAATATATGCTGGTTTCCGAAAGTGAAAAC
ATGCCGCTATAACTAATCGACATTGAGGTATGAAGGTATGGGTGTCGATCGGGATTAGAGACCCCAGTAGTCCATACTACAAAAGATGA
GTATTCCAATAGCACAAA-CTTGGAAGCTAACGCGATAAATACTCCGCCTGGGGAGTACGGCCGCAAGGTTAAAACTTAAGAGATTTGG
CGGGAATTTGTTCGAACGGTGGAACATGTGGTTTAATTCGATAATCCACGAAAAATCTTACCAGTGTTGGAATACACTAGAATCGAAGG
AGTAACGTCATAGATCGATTTTTTAGTGTTAACACGGTGTTGCATGGCTGTCGTCAGTTCGTGCTGTGAAGTGTAGAGTTAAGTCTTAT
AAACGAACAAAATCTCTCGAATTT-TGTGGTTAGGTTGT-TT--TATTTTTTTGCAACTTAATTTTAAAAATTTGAT-ATAAATTGT-T
TGGTG--T-AAGAT--TTGGAAAGT-TA-TTTTTACTTTCC----GATTTATTTATCGTC---G-ATA--T-T-T-GC-T--TATTT-T
TTTTGCA-AAAAAGGGAC-CGAACATCTAC--TTGCTTGTCTAGTAGGTGTTTGTTTTTTTTTTTGTAAATATTA-ATAAGGAT-GCGT
-TATTGGTTTGATA-AGTTTTTCATAA-ATAATTTATTAGAATATAGAGCTGAAGTCAAGTCCTTATGGCCTGTATATGCTGGGCTACA
CACGTGTTACAAGGGTAGAGACAGAGAGTAGCAAGTGTGCGAACAGGAGCAAGCCTCTAAAAATTACCTTAGTTCGGATTGTTTTATGT
AACTCTAAAGCATGAAGTTGAAATCGTTAGTAATTACACTTGAGTATGGTGTAGTGAATGAAAAGTCAAATTTTGCACACACTGCCCAT
CACGCCCGAAGAATTGGTCCGGAGGGAAGGT-G-CCTTGATCGCTGTGGTG-AGTTGT-AGTAA-AACAAGTCCCCTGCTATAATTAGT
CA-TTTACGGATGGCGGTCTTTGTTCATCGAAGTCAGTTTGTATTTGTGTAATTGACACAAGG
>Haptophrya_planariarum_DL_25_DG
TTACTCGTCGTTACTGGGCGTATAGGATACGTAGATGGTTCTAAACATGCTA--T-TTACTG-AAACGATTTTGTTAGGGGGTGGTAAT
TTAGGTACTTGGTTTAGTTAAGGGCGGCACACAGGTGTATGTTAGCGATAAAATGCAGAAAATATATGCTGGTTTCCGAAAGTGAAAAC
ATGCCGCTATAACTAATCGACATTGAGGTATGAAGGTATGGGTGTCGATCGGGATTAGAGACCCCAGTAGTCCATACTACAAAAGATGA
GTATTCCAATAGCACAAA-CTTGGAAGCTAACGCGATAAATACTCCGCCTGGGGAGTACGGCCGCAAGGTTAAAACTTAAGAGATTTGG
CGGGAATTTGTTCGAACGGTGGAACATGTGGTTTAATTCGATAATCCACGAAAAATCTTACCAGTGTTGGAATACACTAGAATCGAAGG
AGTAACGTCATAGATCGATTTTTTAGTGTTAACACGGTGTTGCATGGCTGTCGTCAGTTCGTGCTGTGAAGTGTAGAGTTAAGTCTTAT
AAACGAACAAAATCTCTCGAATTT-TGTGGTTAGGTTGT-TT--TATTTTTTTGCAACTTAATTTTAAAAATTTGAT-ATAAATTGT-T
TGGTG--T-AAGAT--TTGGAAAGT-TA-TTTTTACTTTCC----GATTTATTTATCGTC---G-ATA--T-T-T-GC-T--TATTT-T
TTTTGCA-AAAAAGGGAC-CGAACATCTAC--TTGCTTGTCTAGTAGGTGTTTGTTTTTTTTTTTGTAAATATTA-ATAAGGAT-GCGT
-TATTGGTTTGATA-AGTTTTTCATAA-ATAATTTATTAGAATATAGAGCTGAAGTCAAGTCCTTATGGCCTGTATATGCTGGGCTACA
CACGTGTTACAAGGGTAGAGACAGAGAGTAGCAAGTGTGCGAACAGGAGCAAGCCTCTAAAAATTACCTTAGTTCGGATTGTTTTATGT
AACTCTAAAGCATGAAGTTGAAATCGTTAGTAATTACACTTGAGTATGGTGTAGTGAATGAAAAGTCAAATTTTGCACACACTGCCCAT
CACGCCCGAAGAATTGGTCCGGAGGGAAGGT-G-CCTTGATCGCTGTGGTG-AGTTGT-AGTAA-AACAAGTCCCCTGCTATAATTAGT
CA-TTTACGGATGGCGGTCTTTGTTCATCGAAGTCAGTTTGTATTTGTGTAATTGACACAAGG
>Haptophrya_planariarum_KD_26_DG
TTACTCGTCGTTACTGGGCGTATAGGATACGTAGATGGTTCTAAACATGCTA--T-TTACTG-AAACGATTTTGTTAGGGGGTGGTAAT
TTAGGTACTTGGTTTAGTTAAGGGCGGCACACAGGTGTATGTTAGCGATAAAATGCAGAAAATATATGCTGGTTTCCGAAAGTGAAAAC
ATGCCGCTATAACTAATCGACATTGAGGTATGAAGGTATGGGTGTCGATCGGGATTAGAGACCCCAGTAGTCCATACTACAAAAGATGA
GTATTCCAATAGCACAAA-CTTGGAAGCTAACGCGATAAATACTCCGCCTGGGGAGTACGGCCGCAAGGTTAAAACTTAAGAGATTTGG
CGGGAATTTGTTCGAACGGTGGAACATGTGGTTTAATTCGATAATCCACGAAAAATCTTACCAGTGTTGGAATACACTAGAATCGAAGG
AGTAACGTCATAGATCGATTTTTTAGTGTTAACACGGTGTTGCATGGCTGTCGTCAGTTCGTGCTGTGAAGTGTAGAGTTAAGTCTTAT
AAACGAACAAAATCTCTCGAATTT-TGTGGTTAGGTTGT-TT--TATTTTTTTGCAACTTAATTTTAAAAATTTGAT-ATAAATTGT-T
TGGTG--T-AAGAT--TTGGAAAGT-TA-TTTTTACTTTCC----GATTTATTTATCGTC---G-ATA--T-T-T-GC-T--TATTT-T
TTTTGCA-AAAAAGGGAC-CGAACATCTAC--TTGCTTGTCTAGTAGGTGTTTGTTTTTTTTTTTGTAAATATTA-ATAAGGAT-GCGT
-TATTGGTTTGATA-AGTTTTTCATAA-ATAATTTATTAGAATATAGAGCTGAAGTCAAGTCCTTATGGCCTGTATATGCTGGGCTACA

CACGTGTTACAAGGGTAGAGACAGAGAGTAGCAAGTGTGCGAACAGGAGCAAGCCTCTAAAAATTACCTTAGTTCGGATTGTTTTATGT
AACTCTAAAGCATGAAGTTGAAATCGTTAGTAATTACACTTGAGTATGGTGTAGTGAATGAAAAGTCAAATTTTGCACACACTGCCCAT
CACGCCCGAAGAATTGGTCCGGAGGGAAGGT-G-CCTTGATCGCTGTGGTG-AGTTGT-AGTAA-AACAAGTCCCCTGCTATAATTAGT
CA-TTTACGGATGGCGGTCTTTGTTCATCGAAGTCAGTTTGTATTTGTGTAATTGACACAAGG
>Haptophrya_planariarum_KD_27_DG
TTACTCGTCGTTACTGGGCGTATAGGATACGTAGATGGTTCTAAACATGCTA--T-TTACTG-AAACGATTTTGTTAGGGGGTGGTAAT
TTAGGTACTTGGTTTAGTTAAGGGCGGCACACAGGTGTATGTTAGCGATAAAATGCAGAAAATATATGCTGGTTTCCGAAAGTGAAAAC
ATGCCGCTATAACTAATCGACATTGAGGTATGAAGGTATGGGTGTCGATCGGGATTAGAGACCCCAGTAGTCCATACTACAAAAGATGA
GTATTCCAATAGCACAAA-CTTGGAAGCTAACGCGATAAATACTCCGCCTGGGGAGTACGGCCGCAAGGTTAAAACTTAAGAGATTTGG
CGGGAATTTGTTCGAACGGTGGAACATGTGGTTTAATTCGATAATCCACGAAAAATCTTACCAGTGTTGGAATACACTAGAATCGAAGG
AGTAACGTCATAGATCGATTTTTTAGTGTTAACACGGTGTTGCATGGCTGTCGTCAGTTCGTGCTGTGAAGTGTAGAGTTAAGTCTTAT
AAACGAACAAAATCTCTCGAATTT-TGTGGTTAGGTTGT-TT--TATTTTTTTGCAACTTAATTTTAAAAATTTGAT-ATAAATTGT-T
TGGTG--T-AAGAT--TTGGAAAGT-TA-TTTTTACTTTCC----GATTTATTTATCGTC---G-ATA--T-T-T-GC-T--TATTT-T
TTTTGCA-AAAAAGGGAC-CGAACATCTAC--TTGCTTGTCTAGTAGGTGTTTGTTTTTTTTTTTGTAAATATTA-ATAAGGAT-GCGT
-TATTGGTTTGATA-AGTTTTTCATAA-ATAATTTATTAGAATATAGAGCTGAAGTCAAGTCCTTATGGCCTGTATATGCTGGGCTACA
CACGTGTTACAAGGGTAGAGACAGAGAGTAGCAAGTGTGCGAACAGGAGCAAGCCTCTAAAAATTACCTTAGTTCGGATTGTTTTATGT
AACTCTAAAGCATGAAGTTGAAATCGTTAGTAATTACACTTGAGTATGGTGTAGTGAATGAAAAGTCAAATTTTGCACACACTGCCCAT
CACGCCCGAAGAATTGGTCCGGAGGGAAGGT-G-CCTTGATCGCTGTGGTG-AGTTGT-AGTAA-AACAAGTCCCCTGCTATAATTAGT
CA-TTTACGGATGGCGGTCTTTGTTCATCGAAGTCAGTTTGTATTTGTGTAATTGACACAAGG
>Haptophrya_planariarum_DL_28_DG
TTACTCGTCGTTACTGGGCGTATAGGATACGTAGATGGTTCTAAACATGCTA--T-TTACTG-AAACGATTTTGTTAGGGGGTGGTAAT
TTAGGTACTTGGTTTAGTTAAGGGCGGCACACAGGTGTATGTTAGCGATAAAATGCAGAAAATATATGCTGGTTTCCGAAAGTGAAAAC
ATGCCGCTATAACTAATCGACATTGAGGTATGAAGGTATGGGTGTCGATCGGGATTAGAGACCCCAGTAGTCCATACTACAAAAGATGA
GTATTCCAATAGCACAAA-CTTGGAAGCTAACGCGATAAATACTCCGCCTGGGGAGTACGGCCGCAAGGTTAAAACTTAAGAGATTTGG
CGGGAATTTGTTCGAACGGTGGAACATGTGGTTTAATTCGATAATCCACGAAAAATCTTACCAGTGTTGGAATACACTAGAATCGAAGG
AGTAACGTCATAGATCGATTTTTTAGTGTTAACACGGTGTTGCATGGCTGTCGTCAGTTCGTGCTGTGAAGTGTAGAGTTAAGTCTTAT
AAACGAACAAAATCTCTCGAATTT-TGTGGTTAGGTTGT-TT--TATTTTTTTGCAACTTAATTTTAAAAATTTGAT-ATAAATTGT-T
TGGTG--T-AAGAT--TTGGAAAGT-TA-TTTTTACTTTCC----GATTTATTTATCGTC---G-ATA--T-T-T-GC-T--TATTT-T
TTTTGCA-AAAAAGGGAC-CGAACATCTAC--TTGCTTGTCTAGTAGGTGTTTGTTTTTTTTTTTGTAAATATTA-ATAAGGAT-GCGT
-TATTGGTTTGATA-AGTTTTTCATAA-ATAATTTATTAGAATATAGAGCTGAAGTCAAGTCCTTATGGCCTGTATATGCTGGGCTACA
CACGTGTTACAAGGGTAGAGACAGAGAGTAGCAAGTGTGCGAACAGGAGCAAGCCTCTAAAAATTACCTTAGTTCGGATTGTTTTATGT
AACTCTAAAGCATGAAGTTGAAATCGTTAGTAATTACACTTGAGTATGGTGTAGTGAATGAAAAGTCAAATTTTGCACACACTGCCCAT
CACGCCCGAAGAATTGGTCCGGAGGGAAGGT-G-CCTTGATCGCTGTGGTG-AGTTGT-AGTAA-AACAAGTCCCCTGCTATAATTAGT
CA-TTTACGGATGGCGGTCTTTGTTCATCGAAGTCAGTTTGTATTTGTGTAATTGACACAAGG
>Haptophrya_planariarum_KD_29_DG
TTACTCGTCGTTACTGGGCGTATAGGATACGTAGATGGTTCTAAACATGCTA--T-TTACTG-AAACGATTTTGTTAGGGGGTGGTAAT
TTAGGTACTTGGTTTAGTTAAGGGCGGCACACAGGTGTATGTTAGCGATAAAATGCAGAAAATATATGCTGGTTTCCGAAAGTGAAAAC
ATGCCGCTATAACTAATCGACATTGAGGTATGAAGGTATGGGTGTCGATCGGGATTAGAGACCCCAGTAGTCCATACTACAAAAGATGA
GTATTCCAATAGCACAAA-CTTGGAAGCTAACGCGATAAATACTCCGCCTGGGGAGTACGGCCGCAAGGTTAAAACTTAAGAGATTTGG
CGGGAATTTGTTCGAACGGTGGAACATGTGGTTTAATTCGATAATCCACGAAAAATCTTACCAGTGTTGGAATACACTAGAATCGAAGG
AGTAACGTCATAGATCGATTTTTTAGTGTTAACACGGTGTTGCATGGCTGTCGTCAGTTCGTGCTGTGAAGTGTAGAGTTAAGTCTTAT
AAACGAACAAAATCTCTCGAATTT-TGTGGTTAGGTTGT-TT--TATTTTTTTGCAACTTAATTTTAAAAATTTGAT-ATAAATTGT-T
TGGTG--T-AAGAT--TTGGAAAGT-TA-TTTTTACTTTCC----GATTTATTTATCGTC---G-ATA--T-T-T-GC-T--TATTT-T
TTTTGCA-AAAAAGGGAC-CGAACATCTAC--TTGCTTGTCTAGTAGGTGTTTGTTTTTTTTTTTGTAAATATTA-ATAAGGAT-GCGT
-TATTGGTTTGATA-AGTTTTTCATAA-ATAATTTATTAGAATATAGAGCTGAAGTCAAGTCCTTATGGCCTGTATATGCTGGGCTACA
CACGTGTTACAAGGGTAGAGACAGAGAGTAGCAAGTGTGCGAACAGGAGCAAGCCTCTAAAAATTACCTTAGTTCGGATTGTTTTATGT
AACTCTAAAGCATGAAGTTGAAATCGTTAGTAATTACACTTGAGTATGGTGTAGTGAATGAAAAGTCAAATTTTGCACACACTGCCCAT
CACGCCCGAAGAATTGGTCCGGAGGGAAGGT-G-CCTTGATCGCTGTGGTG-AGTTGT-AGTAA-AACAAGTCCCCTGCTATAATTAGT
CA-TTTACGGATGGCGGTCTTTGTTCATCGAAGTCAGTTTGTATTTGTGTAATTGACACAAGG
>Haptophrya_planariarum_KDo_33_DG
TTACTCGTCGTTACTGGGCGTATAGGATACGTAGATGGTTCTAAACATGCTA--T-TTACTG-AAACGATTTTGTTAGGGGGTGGTAAT
TTAGGTACTTGGTTTAGTTAAGGGCGGCACACAGGTGTATGTTAGCGATAAAATGCAGAAAATATATGCTGGTTTCCGAAAGTGAAAAC
ATGCCGCTATAACTAATCGACATTGAGGTATGAAGGTATGGGTGTCGATCGGGATTAGAGACCCCAGTAGTCCATACTACAAAAGATGA
GTATTCCAATAGCACAAA-CTTGGAAGCTAACGCGATAAATACTCCGCCTGGGGAGTACGGCCGCAAGGTTAAAACTTAAGAGATTTGG
CGGGAATTTGTTCGAACGGTGGAACATGTGGTTTAATTCGATAATCCACGAAAAATCTTACCAGTGTTGGAATACACTAGAATCGAAGG
AGTAACGTCATAGATCGATTTTTTAGTGTTAACACGGTGTTGCATGGCTGTCGTCAGTTCGTGCTGTGAAGTGTAGAGTTAAGTCTTAT
AAACGAACAAAATCTCTCGAATTT-TGTGGTTAGGTTGT-TT--TATTTTTTTGCAACTTAATTTTAAAAATTTGAT-ATAAATTGT-T
TGGTG--T-AAGAT--TTGGAAAGT-TA-TTTTTACTTTCC----GATTTATTTATCGTC---G-ATA--T-T-T-GC-T--TATTT-T
TTTTGCA-AAAAAGGGAC-CGAACATCTAC--TTGCTTGTCTAGTAGGTGTTTGTTTTTTTTTTTGTAAATATTA-ATAAGGAT-GCGT
-TATTGGTTTGATA-AGTTTTTCATAA-ATAATTTATTAGAATATAGAGCTGAAGTCAAGTCCTTATGGCCTGTATATGCTGGGCTACA
CACGTGTTACAAGGGTAGAGACAGAGAGTAGCAAGTGTGCGAACAGGAGCAAGCCTCTAAAAATTACCTTAGTTCGGATTGTTTTATGT
AACTCTAAAGCATGAAGTTGAAATCGTTAGTAATTACACTTGAGTATGGTGTAGTGAATGAAAAGTCAAATTTTGCACACACTGCCCAT
CACGCCCGAAGAATTGGTCCGGAGGGAAGGT-G-CCTTGATCGCTGTGGTG-AGTTGT-AGTAA-AACAAGTCCCCTGCTATAATTAGT
CA-TTTACGGATGGCGGTCTTTGTTCATCGAAGTCAGTTTGTATTTGTGTAATTGACACAAGG
>Haptophrya_planariarum_MH_34_DG
TTACTCGTCGTTACTGGGCGTATAGGATACGTAGATGGTTCTAAACATGCTA--T-TTACTG-AAACGATTTTGTTAGGGGGTGGTAAT
TTAGGTACTTGGTTTAGTTAAGGGCGGCACACAGGTGTATGTTAGCGATAAAATGCAGAAAATATATGCTGGTTTCCGAAAGTGAAAAC
ATGCCGCTATAACTAATCGACATTGAGGTATGAAGGTATGGGTGTCGATCGGGATTAGAGACCCCAGTAGTCCATACTACAAAAGATGA
GTATTCCAATAGCACAAA-CTTGGAAGCTAACGCGATAAATACTCCGCCTGGGGAGTACGGCCGCAAGGTTAAAACTTAAGAGATTTGG
CGGGAATTTGTTCGAACGGTGGAACATGTGGTTTAATTCGATAATCCACGAAAAATCTTACCAGTGTTGGAATACACTAGAATCGAAGG
AGTAACGTCATAGATCGATTTTTTAGTGTTAACACGGTGTTGCATGGCTGTCGTCAGTTCGTGCTGTGAAGTGTAGAGTTAAGTCTTAT

AAACGAACAAAATCTCTCGAATTT-TGTGGTTAGGTTGT-TT--TATTTTTTTGCAACTTAATTTTAAAAATTTGAT-ATAAATTGT-T
TGGTG--T-AAGAT--TTGGAAAGT-TA-TTTTTACTTTCC----GATTTATTTATCGTC---G-ATA--T-T-T-GC-T--TATTT-T
TTTTGCA-AAAAAGGGAC-CGAACATCTAC--TTGCTTGTCTAGTAGGTGTTTGTTTTTTTTTTTGTAAATATTA-ATAAGGAT-GCGT
-TATTGGTTTGATA-AGTTTTTCATAA-ATAATTTATTAGAATATAGAGCTGAAGTCAAGTCCTTATGGCCTGTATATGCTGGGCTACA
CACGTGTTACAAGGGTAGAGACAGAGAGTAGCAAGTGTGCGAACAGGAGCAAGCCTCTAAAAATTACCTTAGTTCGGATTGTTTTATGT
AACTCTAAAGCATGAAGTTGAAATCGTTAGTAATTACACTTGAGTATGGTGTAGTGAATGAAAAGTCAAATTTTGCACACACTGCCCAT
CACGCCCGAAGAATTGGTCCGGAGGGAAGGT-G-CCTTGATCGCTGTGGTG-AGTTGT-AGTAA-AACAAGTCCCCTGCTATAATTAGT
CA-TTTACGGATGGCGGTCTTTGTTCATCGAAGTCAGTTTGTATTTGTGTAATTGACACAAGG
>Haptophrya_planariarum_MH_35_DG
TTACTCGTCGTTACTGGGCGTATAGGATACGTAGATGGTTCTAAACATGCTA--T-TTACTG-AAACGATTTTGTTAGGGGGTGGTAAT
TTAGGTACTTGGTTTAGTTAAGGGCGGCACACAGGTGTATGTTAGCGATAAAATGCAGAAAATATATGCTGGTTTCCGAAAGTGAAAAC
ATGCCGCTATAACTAATCGACATTGAGGTATGAAGGTATGGGTGTCGATCGGGATTAGAGACCCCAGTAGTCCATACTACAAAAGATGA
GTATTCCAATAGCACAAA-CTTGGAAGCTAACGCGATAAATACTCCGCCTGGGGAGTACGGCCGCAAGGTTAAAACTTAAGAGATTTGG
CGGGAATTTGTTCGAACGGTGGAACATGTGGTTTAATTCGATAATCCACGAAAAATCTTACCAGTGTTGGAATACACTAGAATCGAAGG
AGTAACGTCATAGATCGATTTTTTAGTGTTAACACGGTGTTGCATGGCTGTCGTCAGTTCGTGCTGTGAAGTGTAGAGTTAAGTCTTAT
AAACGAACAAAATCTCTCGAATTT-TGTGGTTAGGTTGT-TT--TATTTTTTTGCAACTTAATTTTAAAAATTTGAT-ATAAATTGT-T
TGGTG--T-AAGAT--TTGGAAAGT-TA-TTTTTACTTTCC----GATTTATTTATCGTC---G-ATA--T-T-T-GC-T--TATTT-T
TTTTGCA-AAAAAGGGAC-CGAACATCTAC--TTGCTTGTCTAGTAGGTGTTTGTTTTTTTTTTTGTAAATATTA-ATAAGGAT-GCGT
-TATTGGTTTGATA-AGTTTTTCATAA-ATAATTTATTAGAATATAGAGCTGAAGTCAAGTCCTTATGGCCTGTATATGCTGGGCTACA
CACGTGTTACAAGGGTAGAGACAGAGAGTAGCAAGTGTGCGAACAGGAGCAAGCCTCTAAAAATTACCTTAGTTCGGATTGTTTTATGT
AACTCTAAAGCATGAAGTTGAAATCGTTAGTAATTACACTTGAGTATGGTGTAGTGAATGAAAAGTCAAATTTTGCACACACTGCCCAT
CACGCCCGAAGAATTGGTCCGGAGGGAAGGT-G-CCTTGATCGCTGTGGTG-AGTTGT-AGTAA-AACAAGTCCCCTGCTATAATTAGT
CA-TTTACGGATGGCGGTCTTTGTTCATCGAAGTCAGTTTGTATTTGTGTAATTGACACAAGG
>Haptophrya_planariarum_MH_36_DG
TTACTCGTCGTTACTGGGCGTATAGGATACGTAGATGGTTCTAAACATGCTA--T-TTACTG-AAACGATTTTGTTAGGGGGTGGTAAT
TTAGGTACTTGGTTTAGTTAAGGGCGGCACACAGGTGTATGTTAGCGATAAAATGCAGAAAATATATGCTGGTTTCCGAAAGTGAAAAC
ATGCCGCTATAACTAATCGACATTGAGGTATGAAGGTATGGGTGTCGATCGGGATTAGAGACCCCAGTAGTCCATACTACAAAAGATGA
GTATTCCAATAGCACAAA-CTTGGAAGCTAACGCGATAAATACTCCGCCTGGGGAGTACGGCCGCAAGGTTAAAACTTAAGAGATTTGG
CGGGAATTTGTTCGAACGGTGGAACATGTGGTTTAATTCGATAATCCACGAAAAATCTTACCAGTGTTGGAATACACTAGAATCGAAGG
AGTAACGTCATAGATCGATTTTTTAGTGTTAACACGGTGTTGCATGGCTGTCGTCAGTTCGTGCTGTGAAGTGTAGAGTTAAGTCTTAT
AAACGAACAAAATCTCTCGAATTT-TGTGGTTAGGTTGT-TT--TATTTTTTTGCAACTTAATTTTAAAAATTTGAT-ATAAATTGT-T
TGGTG--T-AAGAT--TTGGAAAGT-TA-TTTTTACTTTCC----GATTTATTTATCGTC---G-ATA--T-T-T-GC-T--TATTT-T
TTTTGCA-AAAAAGGGAC-CGAACATCTAC--TTGCTTGTCTAGTAGGTGTTTGTTTTTTTTTTTGTAAATATTA-ATAAGGAT-GCGT
-TATTGGTTTGATA-AGTTTTTCATAA-ATAATTTATTAGAATATAGAGCTGAAGTCAAGTCCTTATGGCCTGTATATGCTGGGCTACA
CACGTGTTACAAGGGTAGAGACAGAGAGTAGCAAGTGTGCGAACAGGAGCAAGCCTCTAAAAATTACCTTAGTTCGGATTGTTTTATGT
AACTCTAAAGCATGAAGTTGAAATCGTTAGTAATTACACTTGAGTATGGTGTAGTGAATGAAAAGTCAAATTTTGCACACACTGCCCAT
CACGCCCGAAGAATTGGTCCGGAGGGAAGGT-G-CCTTGATCGCTGTGGTG-AGTTGT-AGTAA-AACAAGTCCCCTGCTATAATTAGT
CA-TTTACGGATGGCGGTCTTTGTTCATCGAAGTCAGTTTGTATTTGTGTAATTGACACAAGG
>Haptophrya_planariarum_MH_37_DG
TTACTCGTCGTTACTGGGCGTATAGGATACGTAGATGGTTCTAAACATGCTA--T-TTACTG-AAACGATTTTGTTAGGGGGTGGTAAT
TTAGGTACTTGGTTTAGTTAAGGGCGGCACACAGGTGTATGTTAGCGATAAAATGCAGAAAATATATGCTGGTTTCCGAAAGTGAAAAC
ATGCCGCTATAACTAATCGACATTGAGGTATGAAGGTATGGGTGTCGATCGGGATTAGAGACCCCAGTAGTCCATACTACAAAAGATGA
GTATTCCAATAGCACAAA-CTTGGAAGCTAACGCGATAAATACTCCGCCTGGGGAGTACGGCCGCAAGGTTAAAACTTAAGAGATTTGG
CGGGAATTTGTTCGAACGGTGGAACATGTGGTTTAATTCGATAATCCACGAAAAATCTTACCAGTGTTGGAATACACTAGAATCGAAGG
AGTAACGTCATAGATCGATTTTTTAGTGTTAACACGGTGTTGCATGGCTGTCGTCAGTTCGTGCTGTGAAGTGTAGAGTTAAGTCTTAT
AAACGAACAAAATCTCTCGAATTT-TGTGGTTAGGTTGT-TT--TATTTTTTTGCAACTTAATTTTAAAAATTTGAT-ATAAATTGT-T
TGGTG--T-AAGAT--TTGGAAAGT-TA-TTTTTACTTTCC----GATTTATTTATCGTC---G-ATA--T-T-T-GC-T--TATTT-T
TTTTGCA-AAAAAGGGAC-CGAACATCTAC--TTGCTTGTCTAGTAGGTGTTTGTTTTTTTTTTTGTAAATATTA-ATAAGGAT-GCGT
-TATTGGTTTGATA-AGTTTTTCATAA-ATAATTTATTAGAATATAGAGCTGAAGTCAAGTCCTTATGGCCTGTATATGCTGGGCTACA
CACGTGTTACAAGGGTAGAGACAGAGAGTAGCAAGTGTGCGAACAGGAGCAAGCCTCTAAAAATTACCTTAGTTCGGATTGTTTTATGT
AACTCTAAAGCATGAAGTTGAAATCGTTAGTAATTACACTTGAGTATGGTGTAGTGAATGAAAAGTCAAATTTTGCACACACTGCCCAT
CACGCCCGAAGAATTGGTCCGGAGGGAAGGT-G-CCTTGATCGCTGTGGTG-AGTTGT-AGTAA-AACAAGTCCCCTGCTATAATTAGT
CA-TTTACGGATGGCGGTCTTTGTTCATCGAAGTCAGTTTGTATTTGTGTAATTGACACAAGG
>Haptophrya_planariarum_RT_42_DG
TTACTCGTCGTTACTGGGCGTATAGGATACGTAGATGGTTCTAAACATGCTA--T-TTACTG-AAACGATTTTGTTAGGGGGTGGTAAT
TTAGGTACTTGGTTTAGTTAAGGGCGGCACACAGGTGTATGTTAGCGATAAAATGCAGAAAATATATGCTGGTTTCCGAAAGTGAAAAC
ATGCCGCTATAACTAATCGACATTGAGGTATGAAGGTATGGGTGTCGATCGGGATTAGAGACCCCAGTAGTCCATACTACAAAAGATGA
GTATTCCAATAGCACAAA-CTTGGAAGCTAACGCGATAAATACTCCGCCTGGGGAGTACGGCCGCAAGGTTAAAACTTAAGAGATTTGG
CGGGAATTTGTTCGAACGGTGGAACATGTGGTTTAATTCGATAATCCACGAAAAATCTTACCAGTGTTGGAATACACTAGAATCGAAGG
AGTAACGTCATAGATCGATTTTTTAGTGTTAACACGGTGTTGCATGGCTGTCGTCAGTTCGTGCTGTGAAGTGTAGAGTTAAGTCTTAT
AAACGAACAAAATCTCTCGAATTT-TGTGGTTAGGTTGT-TT--TATTTTTTTGCAACTTAATTTTAAAAATTTGAT-ATAAATTGT-T
TGGTG--T-AAGAT--TTGGAAAGT-TA-TTTTTACTTTCC----GATTTATTTATCGTC---G-ATA--T-T-T-GC-T--TATTT-T
TTTTGCA-AAAAAGGGAC-CGAACATCTAC--TTGCTTGTCTAGTAGGTGTTTGTTTTTTTTTTTGTAAATATTA-ATAAGGAT-GCGT
-TATTGGTTTGATA-AGTTTTTCATAA-ATAATTTATTAGAATATAGAGCTGAAGTCAAGTCCTTATGGCCTGTATATGCTGGGCTACA
CACGTGTTACAAGGGTAGAGACAGAGAGTAGCAAGTGTGCGAACAGGAGCAAGCCTCTAAAAATTACCTTAGTTCGGATTGTTTTATGT
AACTCTAAAGCATGAAGTTGAAATCGTTAGTAATTACACTTGAGTATGGTGTAGTGAATGAAAAGTCAAATTTTGCACACACTGCCCAT
CACGCCCGAAGAATTGGTCCGGAGGGAAGGT-G-CCTTGATCGCTGTGGTG-AGTTGT-AGTAA-AACAAGTCCCCTGCTATAATTAGT
CA-TTTACGGATGGCGGTCTTTGTTCATCGAAGTCAGTTTGTATTTGTGTAATTGACACAAGG
>Haptophrya_planariarum_RT_43_DG
TTACTCGTCGTTACTGGGCGTATAGGATACGTAGATGGTTCTAAACATGCTA--T-TTACTG-AAACGATTTTGTTAGGGGGTGGTAAT
TTAGGTACTTGGTTTAGTTAAGGGCGGCACACAGGTGTATGTTAGCGATAAAATGCAGAAAATATATGCTGGTTTCCGAAAGTGAAAAC

ATGCCGCTATAACTAATCGACATTGAGGTATGAAGGTATGGGTGTCGATCGGGATTAGAGACCCCAGTAGTCCATACTACAAAAGATGA
GTATTCCAATAGCACAAA-CTTGGAAGCTAACGCGATAAATACTCCGCCTGGGGAGTACGGCCGCAAGGTTAAAACTTAAGAGATTTGG
CGGGAATTTGTTCGAACGGTGGAACATGTGGTTTAATTCGATAATCCACGAAAAATCTTACCAGTGTTGGAATACACTAGAATCGAAGG
AGTAACGTCATAGATCGATTTTTTAGTGTTAACACGGTGTTGCATGGCTGTCGTCAGTTCGTGCTGTGAAGTGTAGAGTTAAGTCTTAT
AAACGAACAAAATCTCTCGAATTT-TGTGGTTAGGTTGT-TT--TATTTTTTTGCAACTTAATTTTAAAAATTTGAT-ATAAATTGT-T
TGGTG--T-AAGAT--TTGGAAAGT-TA-TTTTTACTTTCC----GATTTATTTATCGTC---G-ATA--T-T-T-GC-T--TATTT-T
TTTTGCA-AAAAAGGGAC-CGAACATCTAC--TTGCTTGTCTAGTAGGTGTTTGTTTTTTTTTTTGTAAATATTA-ATAAGGAT-GCGT
-TATTGGTTTGATA-AGTTTTTCATAA-ATAATTTATTAGAATATAGAGCTGAAGTCAAGTCCTTATGGCCTGTATATGCTGGGCTACA
CACGTGTTACAAGGGTAGAGACAGAGAGTAGCAAGTGTGCGAACAGGAGCAAGCCTCTAAAAATTACCTTAGTTCGGATTGTTTTATGT
AACTCTAAAGCATGAAGTTGAAATCGTTAGTAATTACACTTGAGTATGGTGTAGTGAATGAAAAGTCAAATTTTGCACACACTGCCCAT
CACGCCCGAAGAATTGGTCCGGAGGGAAGGT-G-CCTTGATCGCTGTGGTG-AGTTGT-AGTAA-AACAAGTCCCCTGCTATAATTAGT
CA-TTTACGGATGGCGGTCTTTGTTCATCGAAGTCAGTTTGTATTTGTGTAATTGACACAAGG
>Haptophrya_planariarum_RT_44_DG
TTACTCGTCGTTACTGGGCGTATAGGATACGTAGATGGTTCTAAACATGCTA--T-TTACTG-AAACGATTTTGTTAGGGGGTGGTAAT
TTAGGTACTTGGTTTAGTTAAGGGCGGCACACAGGTGTATGTTAGCGATAAAATGCAGAAAATATATGCTGGTTTCCGAAAGTGAAAAC
ATGCCGCTATAACTAATCGACATTGAGGTATGAAGGTATGGGTGTCGATCGGGATTAGAGACCCCAGTAGTCCATACTACAAAAGATGA
GTATTCCAATAGCACAAA-CTTGGAAGCTAACGCGATAAATACTCCGCCTGGGGAGTACGGCCGCAAGGTTAAAACTTAAGAGATTTGG
CGGGAATTTGTTCGAACGGTGGAACATGTGGTTTAATTCGATAATCCACGAAAAATCTTACCAGTGTTGGAATACACTAGAATCGAAGG
AGTAACGTCATAGATCGATTTTTTAGTGTTAACACGGTGTTGCATGGCTGTCGTCAGTTCGTGCTGTGAAGTGTAGAGTTAAGTCTTAT
AAACGAACAAAATCTCTCGAATTT-TGTGGTTAGGTTGT-TT--TATTTTTTTGCAACTTAATTTTAAAAATTTGAT-ATAAATTGT-T
TGGTG--T-AAGAT--TTGGAAAGT-TA-TTTTTACTTTCC----GATTTATTTATCGTC---G-ATA--T-T-T-GC-T--TATTT-T
TTTTGCA-AAAAAGGGAC-CGAACATCTAC--TTGCTTGTCTAGTAGGTGTTTGTTTTTTTTTTTGTAAATATTA-ATAAGGAT-GCGT
-TATTGGTTTGATA-AGTTTTTCATAA-ATAATTTATTAGAATATAGAGCTGAAGTCAAGTCCTTATGGCCTGTATATGCTGGGCTACA
CACGTGTTACAAGGGTAGAGACAGAGAGTAGCAAGTGTGCGAACAGGAGCAAGCCTCTAAAAATTACCTTAGTTCGGATTGTTTTATGT
AACTCTAAAGCATGAAGTTGAAATCGTTAGTAATTACACTTGAGTATGGTGTAGTGAATGAAAAGTCAAATTTTGCACACACTGCCCAT
CACGCCCGAAGAATTGGTCCGGAGGGAAGGT-G-CCTTGATCGCTGTGGTG-AGTTGT-AGTAA-AACAAGTCCCCTGCTATAATTAGT
CA-TTTACGGATGGCGGTCTTTGTTCATCGAAGTCAGTTTGTATTTGTGTAATTGACACAAGG
>Haptophrya_planariarum_RT_47_DG
TTACTCGTCGTTACTGGGCGTATAGGATACGTAGATGGTTCTAAACATGCTA--T-TTACTG-AAACGATTTTGTTAGGGGGTGGTAAT
TTAGGTACTTGGTTTAGTTAAGGGCGGCACACAGGTGTATGTTAGCGATAAAATGCAGAAAATATATGCTGGTTTCCGAAAGTGAAAAC
ATGCCGCTATAACTAATCGACATTGAGGTATGAAGGTATGGGTGTCGATCGGGATTAGAGACCCCAGTAGTCCATACTACAAAAGATGA
GTATTCCAATAGCACAAA-CTTGGAAGCTAACGCGATAAATACTCCGCCTGGGGAGTACGGCCGCAAGGTTAAAACTTAAGAGATTTGG
CGGGAATTTGTTCGAACGGTGGAACATGTGGTTTAATTCGATAATCCACGAAAAATCTTACCAGTGTTGGAATACACTAGAATCGAAGG
AGTAACGTCATAGATCGATTTTTTAGTGTTAACACGGTGTTGCATGGCTGTCGTCAGTTCGTGCTGTGAAGTGTAGAGTTAAGTCTTAT
AAACGAACAAAATCTCTCGAATTT-TGTGGTTAGGTTGT-TT--TATTTTTTTGCAACTTAATTTTAAAAATTTGAT-ATAAATTGT-T
TGGTG--T-AAGAT--TTGGAAAGT-TA-TTTTTACTTTCC----GATTTATTTATCGTC---G-ATA--T-T-T-GC-T--TATTT-T
TTTTGCA-AAAAAGGGAC-CGAACATCTAC--TTGCTTGTCTAGTAGGTGTTTGTTTTTTTTTTTGTAAATATTA-ATAAGGAT-GCGT
-TATTGGTTTGATA-AGTTTTTCATAA-ATAATTTATTAGAATATAGAGCTGAAGTCAAGTCCTTATGGCCTGTATATGCTGGGCTACA
CACGTGTTACAAGGGTAGAGACAGAGAGTAGCAAGTGTGCGAACAGGAGCAAGCCTCTAAAAATTACCTTAGTTCGGATTGTTTTATGT
AACTCTAAAGCATGAAGTTGAAATCGTTAGTAATTACACTTGAGTATGGTGTAGTGAATGAAAAGTCAAATTTTGCACACACTGCCCAT
CACGCCCGAAGAATTGGTCCGGAGGGAAGGT-G-CCTTGATCGCTGTGGTG-AGTTGT-AGTAA-AACAAGTCCCCTGCTATAATTAGT
CA-TTTACGGATGGCGGTCTTTGTTCATCGAAGTCAGTTTGTATTTGTGTAATTGACACAAGG
>Haptophrya_planariarum_RT_48_DG
TTACTCGTCGTTACTGGGCGTATAGGATACGTAGATGGTTCTAAACATGCTA--T-TTACTG-AAACGATTTTGTTAGGGGGTGGTAAT
TTAGGTACTTGGTTTAGTTAAGGGCGGCACACAGGTGTATGTTAGCGATAAAATGCAGAAAATATATGCTGGTTTCCGAAAGTGAAAAC
ATGCCGCTATAACTAATCGACATTGAGGTATGAAGGTATGGGTGTCGATCGGGATTAGAGACCCCAGTAGTCCATACTACAAAAGATGA
GTATTCCAATAGCACAAA-CTTGGAAGCTAACGCGATAAATACTCCGCCTGGGGAGTACGGCCGCAAGGTTAAAACTTAAGAGATTTGG
CGGGAATTTGTTCGAACGGTGGAACATGTGGTTTAATTCGATAATCCACGAAAAATCTTACCAGTGTTGGAATACACTAGAATCGAAGG
AGTAACGTCATAGATCGATTTTTTAGTGTTAACACGGTGTTGCATGGCTGTCGTCAGTTCGTGCTGTGAAGTGTAGAGTTAAGTCTTAT
AAACGAACAAAATCTCTCGAATTT-TGTGGTTAGGTTGT-TT--TATTTTTTTGCAACTTAATTTTAAAAATTTGAT-ATAAATTGT-T
TGGTG--T-AAGAT--TTGGAAAGT-TA-TTTTTACTTTCC----GATTTATTTATCGTC---G-ATA--T-T-T-GC-T--TATTT-T
TTTTGCA-AAAAAGGGAC-CGAACATCTAC--TTGCTTGTCTAGTAGGTGTTTGTTTTTTTTTTTGTAAATATTA-ATAAGGAT-GCGT
-TATTGGTTTGATA-AGTTTTTCATAA-ATAATTTATTAGAATATAGAGCTGAAGTCAAGTCCTTATGGCCTGTATATGCTGGGCTACA
CACGTGTTACAAGGGTAGAGACAGAGAGTAGCAAGTGTGCGAACAGGAGCAAGCCTCTAAAAATTACCTTAGTTCGGATTGTTTTATGT
AACTCTAAAGCATGAAGTTGAAATCGTTAGTAATTACACTTGAGTATGGTGTAGTGAATGAAAAGTCAAATTTTGCACACACTGCCCAT
CACGCCCGAAGAATTGGTCCGGAGGGAAGGT-G-CCTTGATCGCTGTGGTG-AGTTGT-AGTAA-AACAAGTCCCCTGCTATAATTAGT
CA-TTTACGGATGGCGGTCTTTGTTCATCGAAGTCAGTTTGTATTTGTGTAATTGACACAAGG
>Haptophrya_planariarum_CA_50_DG
TTACTCGTCGTTACTGGGCGTATAGGATACGTAGATGGTTCTAAACATGCTA--T-TTACTG-AAACGATTTTGTTAGGGGGTGGTAAT
TTAGGTACTTGGTTTAGTTAAGGGCGGCACACAGGTGTATGTTAGCGATAAAATGCAGAAAATATATGCTGGTTTCCGAAAGTGAAAAC
ATGCCGCTATAACTAATCGACATTGAGGTATGAAGGTATGGGTGTCGATCGGGATTAGAGACCCCAGTAGTCCATACTACAAAAGATGA
GTATTCCAATAGCACAAA-CTTGGAAGCTAACGCGATAAATACTCCGCCTGGGGAGTACGGCCGCAAGGTTAAAACTTAAGAGATTTGG
CGGGAATTTGTTCGAACGGTGGAACATGTGGTTTAATTCGATAATCCACGAAAAATCTTACCAGTGTTGGAATACACTAGAATCGAAGG
AGTAACGTCATAGATCGATTTTTTAGTGTTAACACGGTGTTGCATGGCTGTCGTCAGTTCGTGCTGTGAAGTGTAGAGTTAAGTCTTAT
AAACGAACAAAATCTCTCGAATTT-TGTGGTTAGGTTGT-TT--TATTTTTTTGCAACTTAATTTTAAAAATTTGAT-ATAAATTGT-T
TGGTG--T-AAGAT--TTGGAAAGT-TA-TTTTTACTTTCC----GATTTATTTATCGTC---G-ATA--T-T-T-GC-T--TATTT-T
TTTTGCA-AAAAAGGGAC-CGAACATCTAC--TTGCTTGTCTAGTAGGTGTTTGTTTTTTTTTTTGTAAATATTA-ATAAGGAT-GCGT
-TATTGGTTTGATA-AGTTTTTCATAA-ATAATTTATTAGAATATAGAGCTGAAGTCAAGTCCTTATGGCCTGTATATGCTGGGCTACA
CACGTGTTACAAGGGTAGAGACAGAGAGTAGCAAGTGTGCGAACAGGAGCAAGCCTCTAAAAATTACCTTAGTTCGGATTGTTTTATGT
AACTCTAAAGCATGAAGTTGAAATCGTTAGTAATTACACTTGAGTATGGTGTAGTGAATGAAAAGTCAAATTTTGCACACACTGCCCAT
CACGCCCGAAGAATTGGTCCGGAGGGAAGGT-G-CCTTGATCGCTGTGGTG-AGTTGT-AGTAA-AACAAGTCCCCTGCTATAATTAGT

CA-TTTACGGATGGCGGTCTTTGTTCATCGAAGTCAGTTTGTATTTGTGTAATTGACACAAGG
>Haptophrya_planariarum_CA_51_DG
TTACTCGTCGTTACTGGGCGTATAGGATACGTAGATGGTTCTAAACATGCTA--T-TTACTG-AAACGATTTTGTTAGGGGGTGGTAAT
TTAGGTACTTGGTTTAGTTAAGGGCGGCACACAGGTGTATGTTAGCGATAAAATGCAGAAAATATATGCTGGTTTCCGAAAGTGAAAAC
ATGCCGCTATAACTAATCGACATTGAGGTATGAAGGTATGGGTGTCGATCGGGATTAGAGACCCCAGTAGTCCATACTACAAAAGATGA
GTATTCCAATAGCACAAA-CTTGGAAGCTAACGCGATAAATACTCCGCCTGGGGAGTACGGCCGCAAGGTTAAAACTTAAGAGATTTGG
CGGGAATTTGTTCGAACGGTGGAACATGTGGTTTAATTCGATAATCCACGAAAAATCTTACCAGTGTTGGAATACACTAGAATCGAAGG
AGTAACGTCATAGATCGATTTTTTAGTGTTAACACGGTGTTGCATGGCTGTCGTCAGTTCGTGCTGTGAAGTGTAGAGTTAAGTCTTAT
AAACGAACAAAATCTCTCGAATTT-TGTGGTTAGGTTGT-TT--TATTTTTTTGCAACTTAATTTTAAAAATTTGAT-ATAAATTGT-T
TGGTG--T-AAGAT--TTGGAAAGT-TA-TTTTTACTTTCC----GATTTATTTATCGTC---G-ATA--T-T-T-GC-T--TATTT-T
TTTTGCA-AAAAAGGGAC-CGAACATCTAC--TTGCTTGTCTAGTAGGTGTTTGTTTTTTTTTTTGTAAATATTA-ATAAGGAT-GCGT
-TATTGGTTTGATA-AGTTTTTCATAA-ATAATTTATTAGAATATAGAGCTGAAGTCAAGTCCTTATGGCCTGTATATGCTGGGCTACA
CACGTGTTACAAGGGTAGAGACAGAGAGTAGCAAGTGTGCGAACAGGAGCAAGCCTCTAAAAATTACCTTAGTTCGGATTGTTTTATGT
AACTCTAAAGCATGAAGTTGAAATCGTTAGTAATTACACTTGAGTATGGTGTAGTGAATGAAAAGTCAAATTTTGCACACACTGCCCAT
CACGCCCGAAGAATTGGTCCGGAGGGAAGGT-G-CCTTGATCGCTGTGGTG-AGTTGT-AGTAA-AACAAGTCCCCTGCTATAATTAGT
CA-TTTACGGATGGCGGTCTTTGTTCATCGAAGTCAGTTTGTATTTGTGTAATTGACACAAGG
>Haptophrya_planariarum_CA_52_DG
TTACTCGTCGTTACTGGGCGTATAGGATACGTAGATGGTTCTAAACATGCTA--T-TTACTG-AAACGATTTTGTTAGGGGGTGGTAAT
TTAGGTACTTGGTTTAGTTAAGGGCGGCACACAGGTGTATGTTAGCGATAAAATGCAGAAAATATATGCTGGTTTCCGAAAGTGAAAAC
ATGCCGCTATAACTAATCGACATTGAGGTATGAAGGTATGGGTGTCGATCGGGATTAGAGACCCCAGTAGTCCATACTACAAAAGATGA
GTATTCCAATAGCACAAA-CTTGGAAGCTAACGCGATAAATACTCCGCCTGGGGAGTACGGCCGCAAGGTTAAAACTTAAGAGATTTGG
CGGGAATTTGTTCGAACGGTGGAACATGTGGTTTAATTCGATAATCCACGAAAAATCTTACCAGTGTTGGAATACACTAGAATCGAAGG
AGTAACGTCATAGATCGATTTTTTAGTGTTAACACGGTGTTGCATGGCTGTCGTCAGTTCGTGCTGTGAAGTGTAGAGTTAAGTCTTAT
AAACGAACAAAATCTCTCGAATTT-TGTGGTTAGGTTGT-TT--TATTTTTTTGCAACTTAATTTTAAAAATTTGAT-ATAAATTGT-T
TGGTG--T-AAGAT--TTGGAAAGT-TA-TTTTTACTTTCC----GATTTATTTATCGTC---G-ATA--T-T-T-GC-T--TATTT-T
TTTTGCA-AAAAAGGGAC-CGAACATCTAC--TTGCTTGTCTAGTAGGTGTTTGTTTTTTTTTTTGTAAATATTA-ATAAGGAT-GCGT
-TATTGGTTTGATA-AGTTTTTCATAA-ATAATTTATTAGAATATAGAGCTGAAGTCAAGTCCTTATGGCCTGTATATGCTGGGCTACA
CACGTGTTACAAGGGTAGAGACAGAGAGTAGCAAGTGTGCGAACAGGAGCAAGCCTCTAAAAATTACCTTAGTTCGGATTGTTTTATGT
AACTCTAAAGCATGAAGTTGAAATCGTTAGTAATTACACTTGAGTATGGTGTAGTGAATGAAAAGTCAAATTTTGCACACACTGCCCAT
CACGCCCGAAGAATTGGTCCGGAGGGAAGGT-G-CCTTGATCGCTGTGGTG-AGTTGT-AGTAA-AACAAGTCCCCTGCTATAATTAGT
CA-TTTACGGATGGCGGTCTTTGTTCATCGAAGTCAGTTTGTATTTGTGTAATTGACACAAGG
>Haptophrya_planariarum_DL_54_DG
TTACTCGTCGTTACTGGGCGTATAGGATACGTAGATGGTTCTAAACATGCTA--T-TTACTG-AAACGATTTTGTTAGGGGGTGGTAAT
TTAGGTACTTGGTTTAGTTAAGGGCGGCACACAGGTGTATGTTAGCGATAAAATGCAGAAAATATATGCTGGTTTCCGAAAGTGAAAAC
ATGCCGCTATAACTAATCGACATTGAGGTATGAAGGTATGGGTGTCGATCGGGATTAGAGACCCCAGTAGTCCATACTACAAAAGATGA
GTATTCCAATAGCACAAA-CTTGGAAGCTAACGCGATAAATACTCCGCCTGGGGAGTACGGCCGCAAGGTTAAAACTTAAGAGATTTGG
CGGGAATTTGTTCGAACGGTGGAACATGTGGTTTAATTCGATAATCCACGAAAAATCTTACCAGTGTTGGAATACACTAGAATCGAAGG
AGTAACGTCATAGATCGATTTTTTAGTGTTAACACGGTGTTGCATGGCTGTCGTCAGTTCGTGCTGTGAAGTGTAGAGTTAAGTCTTAT
AAACGAACAAAATCTCTCGAATTT-TGTGGTTAGGTTGT-TT--TATTTTTTTGCAACTTAATTTTAAAAATTTGAT-ATAAATTGT-T
TGGTG--T-AAGAT--TTGGAAAGT-TA-TTTTTACTTTCC----GATTTATTTATCGTC---G-ATA--T-T-T-GC-T--TATTT-T
TTTTGCA-AAAAAGGGAC-CGAACATCTAC--TTGCTTGTCTAGTAGGTGTTTGTTTTTTTTTTTGTAAATATTA-ATAAGGAT-GCGT
-TATTGGTTTGATA-AGTTTTTCATAA-ATAATTTATTAGAATATAGAGCTGAAGTCAAGTCCTTATGGCCTGTATATGCTGGGCTACA
CACGTGTTACAAGGGTAGAGACAGAGAGTAGCAAGTGTGCGAACAGGAGCAAGCCTCTAAAAATTACCTTAGTTCGGATTGTTTTATGT
AACTCTAAAGCATGAAGTTGAAATCGTTAGTAATTACACTTGAGTATGGTGTAGTGAATGAAAAGTCAAATTTTGCACACACTGCCCAT
CACGCCCGAAGAATTGGTCCGGAGGGAAGGT-G-CCTTGATCGCTGTGGTG-AGTTGT-AGTAA-AACAAGTCCCCTGCTATAATTAGT
CA-TTTACGGATGGCGGTCTTTGTTCATCGAAGTCAGTTTGTATTTGTGTAATTGACACAAGG
>Haptophrya_planariarum_BB_85_DG
TTACTCGTCGTTACTGGGCGTATAGGATACGTAGATGGTTCTAAACATGCTA--T-TTACTG-AAACGATTTTGTTAGGGGGTGGTAAT
TTAGGTACTTGGTTTAGTTAAGGGCGGCACACAGGTGTATGTTAGCGATAAAATGCAGAAAATATATGCTGGTTTCCGAAAGTGAAAAC
ATGCCGCTATAACTAATCGACATTGAGGTATGAAGGTATGGGTGTCGATCGGGATTAGAGACCCCAGTAGTCCATACTACAAAAGATGA
GTATTCCAATAGCACAAA-CTTGGAAGCTAACGCGATAAATACTCCGCCTGGGGAGTACGGCCGCAAGGTTAAAACTTAAGAGATTTGG
CGGGAATTTGTTCGAACGGTGGAACATGTGGTTTAATTCGATAATCCACGAAAAATCTTACCAGTGTTGGAATACACTAGAATCGAAGG
AGTAACGTCATAGATCGATTTTTTAGTGTTAACACGGTGTTGCATGGCTGTCGTCAGTTCGTGCTGTGAAGTGTAGAGTTAAGTCTTAT
AAACGAACAAAATCTCTCGAATTT-TGTGGTTAGGTTGT-TT--TATTTTTTTGCAACTTAATTTTAAAAATTTGAT-ATAAATTGT-T
TGGTG--T-AAGAT--TTGGAAAGT-TA-TTTTTACTTTCC----GATTTATTTATCGTC---G-ATA--T-T-T-GC-T--TATTT-T
TTTTGCA-AAAAAGGGAC-CGAACATCTAC--TTGCTTGTCTAGTAGGTGTTTGTTTTTTTTTTTGTAAATATTA-ATAAGGAT-GCGT
-TATTGGTTTGATA-AGTTTTTCATAA-ATAATTTATTAGAATATAGAGCTGAAGTCAAGTCCTTATGGCCTGTATATGCTGGGCTACA
CACGTGTTACAAGGGTAGAGACAGAGAGTAGCAAGTGTGCGAACAGGAGCAAGCCTCTAAAAATTACCTTAGTTCGGATTGTTTTATGT
AACTCTAAAGCATGAAGTTGAAATCGTTAGTAATTACACTTGAGTATGGTGTAGTGAATGAAAAGTCAAATTTTGCACACACTGCCCAT
CACGCCCGAAGAATTGGTCCGGAGGGAAGGT-G-CCTTGATCGCTGTGGTG-AGTTGT-AGTAA-AACAAGTCCCCTGCTATAATTAGT
CA-TTTACGGATGGCGGTCTTTGTTCATCGAAGTCAGTTTGTATTTGTGTAATTGACACAAGG
>Haptophrya_planariarum_BB_99_DG
TTACTCGTCGTTACTGGGCGTATAGGATACGTAGATGGTTCTAAACATGCTA--T-TTACTG-AAACGATTTTGTTAGGGGGTGGTAAT
TTAGGTACTTGGTTTAGTTAAGGGCGGCACACAGGTGTATGTTAGCGATAAAATGCAGAAAATATATGCTGGTTTCCGAAAGTGAAAAC
ATGCCGCTATAACTAATCGACATTGAGGTATGAAGGTATGGGTGTCGATCGGGATTAGAGACCCCAGTAGTCCATACTACAAAAGATGA
GTATTCCAATAGCACAAA-CTTGGAAGCTAACGCGATAAATACTCCGCCTGGGGAGTACGGCCGCAAGGTTAAAACTTAAGAGATTTGG
CGGGAATTTGTTCGAACGGTGGAACATGTGGTTTAATTCGATAATCCACGAAAAATCTTACCAGTGTTGGAATACACTAGAATCGAAGG
AGTAACGTCATAGATCGATTTTTTAGTGTTAACACGGTGTTGCATGGCTGTCGTCAGTTCGTGCTGTGAAGTGTAGAGTTAAGTCTTAT
AAACGAACAAAATCTCTCGAATTT-TGTGGTTAGGTTGT-TT--TATTTTTTTGCAACTTAATTTTAAAAATTTGAT-ATAAATTGT-T
TGGTG--T-AAGAT--TTGGAAAGT-TA-TTTTTACTTTCC----GATTTATTTATCGTC---G-ATA--T-T-T-GC-T--TATTT-T
TTTTGCA-AAAAAGGGAC-CGAACATCTAC--TTGCTTGTCTAGTAGGTGTTTGTTTTTTTTTTTGTAAATATTA-ATAAGGAT-GCGT

-TATTGGTTTGATA-AGTTTTTCATAA-ATAATTTATTAGAATATAGAGCTGAAGTCAAGTCCTTATGGCCTGTATATGCTGGGCTACA
CACGTGTTACAAGGGTAGAGACAGAGAGTAGCAAGTGTGCGAACAGGAGCAAGCCTCTAAAAATTACCTTAGTTCGGATTGTTTTATGT
AACTCTAAAGCATGAAGTTGAAATCGTTAGTAATTACACTTGAGTATGGTGTAGTGAATGAAAAGTCAAATTTTGCACACACTGCCCAT
CACGCCCGAAGAATTGGTCCGGAGGGAAGGT-G-CCTTGATCGCTGTGGTG-AGTTGT-AGTAA-AACAAGTCCCCTGCTATAATTAGT
CA-TTTACGGATGGCGGTCTTTGTTCATCGAAGTCAGTTTGTATTTGTGTAATTGACACAAGG
>Haptophrya_planariarum_ST_132_DG
TTACTCGTCGTTACTGGGCGTATAGGATACGTAGATGGTTCTAAACATGCTA--T-TTACTG-AAACGATTTTGTTAGGGGGTGGTAAT
TTAGGTACTTGGTTTAGTTAAGGGCGGCACACAGGTGTATGTTAGCGATAAAATGCAGAAAATATATGCTGGTTTCCGAAAGTGAAAAC
ATGCCGCTATAACTAATCGACATTGAGGTATGAAGGTATGGGTGTCGATCGGGATTAGAGACCCCAGTAGTCCATACTACAAAAGATGA
GTATTCCAATAGCACAAA-CTTGGAAGCTAACGCGATAAATACTCCGCCTGGGGAGTACGGCCGCAAGGTTAAAACTTAAGAGATTTGG
CGGGAATTTGTTCGAACGGTGGAACATGTGGTTTAATTCGATAATCCACGAAAAATCTTACCAGTGTTGGAATACACTAGAATCGAAGG
AGTAACGTCATAGATCGATTTTTTAGTGTTAACACGGTGTTGCATGGCTGTCGTCAGTTCGTGCTGTGAAGTGTAGAGTTAAGTCTTAT
AAACGAACAAAATCTCTCGAATTT-TGTGGTTAGGTTGT-TT--TATTTTTTTGCAACTTAATTTTAAAAATTTGAT-ATAAATTGT-T
TGGTG--T-AAGAT--TTGGAAAGT-TA-TTTTTACTTTCC----GATTTATTTATCGTC---G-ATA--T-T-T-GC-T--TATTT-T
TTTTGCA-AAAAAGGGAC-CGAACATCTAC--TTGCTTGTCTAGTAGGTGTTTGTTTTTTTTTTTGTAAATATTA-ATAAGGAT-GCGT
-TATTGGTTTGATA-AGTTTTTCATAA-ATAATTTATTAGAATATAGAGCTGAAGTCAAGTCCTTATGGCCTGTATATGCTGGGCTACA
CACGTGTTACAAGGGTAGAGACAGAGAGTAGCAAGTGTGCGAACAGGAGCAAGCCTCTAAAAATTACCTTAGTTCGGATTGTTTTATGT
AACTCTAAAGCATGAAGTTGAAATCGTTAGTAATTACACTTGAGTATGGTGTAGTGAATGAAAAGTCAAATTTTGCACACACTGCCCAT
CACGCCCGAAGAATTGGTCCGGAGGGAAGGT-G-CCTTGATCGCTGTGGTG-AGTTGT-AGTAA-AACAAGTCCCCTGCTATAATTAGT
CA-TTTACGGATGGCGGTCTTTGTTCATCGAAGTCAGTTTGTATTTGTGTAATTGACACAAGG
>Haptophrya_planariarum_BY_139_DG
TTACTCGTCGTTACTGGGCGTATAGGATACGTAGATGGTTCTAAACATGCTA--T-TTACTG-AAACGATTTTGTTAGGGGGTGGTAAT
TTAGGTACTTGGTTTAGTTAAGGGCGGCACACAGGTGTATGTTAGCGATAAAATGCAGAAAATATATGCTGGTTTCCGAAAGTGAAAAC
ATGCCGCTATAACTAATCGACATTGAGGTATGAAGGTATGGGTGTCGATCGGGATTAGAGACCCCAGTAGTCCATACTACAAAAGATGA
GTATTCCAATAGCACAAA-CTTGGAAGCTAACGCGATAAATACTCCGCCTGGGGAGTACGGCCGCAAGGTTAAAACTTAAGAGATTTGG
CGGGAATTTGTTCGAACGGTGGAACATGTGGTTTAATTCGATAATCCACGAAAAATCTTACCAGTGTTGGAATACACTAGAATCGAAGG
AGTAACGTCATAGATCGATTTTTTAGTGTTAACACGGTGTTGCATGGCTGTCGTCAGTTCGTGCTGTGAAGTGTAGAGTTAAGTCTTAT
AAACGAACAAAATCTCTCGAATTT-TGTGGTTAGGTTGT-TT--TATTTTTTTGCAACTTAATTTTAAAAATTTGAT-ATAAATTGT-T
TGGTG--T-AAGAT--TTGGAAAGT-TA-TTTTTACTTTCC----GATTTATTTATCGTC---G-ATA--T-T-T-GC-T--TATTT-T
TTTTGCA-AAAAAGGGAC-CGAACATCTAC--TTGCTTGTCTAGTAGGTGTTTGTTTTTTTTTTTGTAAATATTA-ATAAGGAT-GCGT
-TATTGGTTTGATA-AGTTTTTCATAA-ATAATTTATTAGAATATAGAGCTGAAGTCAAGTCCTTATGGCCTGTATATGCTGGGCTACA
CACGTGTTACAAGGGTAGAGACAGAGAGTAGCAAGTGTGCGAACAGGAGCAAGCCTCTAAAAATTACCTTAGTTCGGATTGTTTTATGT
AACTCTAAAGCATGAAGTTGAAATCGTTAGTAATTACACTTGAGTATGGTGTAGTGAATGAAAAGTCAAATTTTGCACACACTGCCCAT
CACGCCCGAAGAATTGGTCCGGAGGGAAGGT-G-CCTTGATCGCTGTGGTG-AGTTGT-AGTAA-AACAAGTCCCCTGCTATAATTAGT
CA-TTTACGGATGGCGGTCTTTGTTCATCGAAGTCAGTTTGTATTTGTGTAATTGACACAAGG
>Haptophrya_planariarum_BY_149_DG
TTACTCGTCGTTACTGGGCGTATAGGATACGTAGATGGTTCTAAACATGCTA--T-TTACTG-AAACGATTTTGTTAGGGGGTGGTAAT
TTAGGTACTTGGTTTAGTTAAGGGCGGCACACAGGTGTATGTTAGCGATAAAATGCAGAAAATATATGCTGGTTTCCGAAAGTGAAAAC
ATGCCGCTATAACTAATCGACATTGAGGTATGAAGGTATGGGTGTCGATCGGGATTAGAGACCCCAGTAGTCCATACTACAAAAGATGA
GTATTCCAATAGCACAAA-CTTGGAAGCTAACGCGATAAATACTCCGCCTGGGGAGTACGGCCGCAAGGTTAAAACTTAAGAGATTTGG
CGGGAATTTGTTCGAACGGTGGAACATGTGGTTTAATTCGATAATCCACGAAAAATCTTACCAGTGTTGGAATACACTAGAATCGAAGG
AGTAACGTCATAGATCGATTTTTTAGTGTTAACACGGTGTTGCATGGCTGTCGTCAGTTCGTGCTGTGAAGTGTAGAGTTAAGTCTTAT
AAACGAACAAAATCTCTCGAATTT-TGTGGTTAGGTTGT-TT--TATTTTTTTGCAACTTAATTTTAAAAATTTGAT-ATAAATTGT-T
TGGTG--T-AAGAT--TTGGAAAGT-TA-TTTTTACTTTCC----GATTTATTTATCGTC---G-ATA--T-T-T-GC-T--TATTT-T
TTTTGCA-AAAAAGGGAC-CGAACATCTAC--TTGCTTGTCTAGTAGGTGTTTGTTTTTTTTTTTGTAAATATTA-ATAAGGAT-GCGT
-TATTGGTTTGATA-AGTTTTTCATAA-ATAATTTATTAGAATATAGAGCTGAAGTCAAGTCCTTATGGCCTGTATATGCTGGGCTACA
CACGTGTTACAAGGGTAGAGACAGAGAGTAGCAAGTGTGCGAACAGGAGCAAGCCTCTAAAAATTACCTTAGTTCGGATTGTTTTATGT
AACTCTAAAGCATGAAGTTGAAATCGTTAGTAATTACACTTGAGTATGGTGTAGTGAATGAAAAGTCAAATTTTGCACACACTGCCCAT
CACGCCCGAAGAATTGGTCCGGAGGGAAGGT-G-CCTTGATCGCTGTGGTG-AGTTGT-AGTAA-AACAAGTCCCCTGCTATAATTAGT
CA-TTTACGGATGGCGGTCTTTGTTCATCGAAGTCAGTTTGTATTTGTGTAATTGACACAAGG
>Haptophrya_dugesiarum_BB_78_DG
TTACTCGTCGTTACTGGGCGTATAGGATACGTAGACGGTTCCAAA-ATGCTA--G-TTACTTAAA-CTATTTGGTTAGGGGTTAGTAAT
TTGGGTACTTGGTTTAGTTAGGGGCTGCGCGCAGGTGTACGTTAGCGATAAAATGCATAAAATGTATGCTGGTTTCCAACAGTGAAAAC
ACACAGCTATAACTAATCGACGTTGAGGTATGAAGGTATGGGTGTCGATCGGGATTAGAGACCCCAGTAGTCCATACTACAAAAGATGA
GTATTCCAATAATACAAAACTTGGAAGCTAACGCGATAAATACTCCGCCTGGGAAGTACGGCCGCAAGGTTAAAACTTAAGAGATTTGG
CGGGAATTTGTTCGAACGGTGGAACATGTGGTTTAATTCGATAATCCACGAAAAATCTTACCAGTGTCGTAATACATTAGAATCGAAGA
GGTAACGCTATCTATCGATTTTTTAGTGTTAACAAGGTGCTGCATGGCTGTCGTCAGTTCGTGCTGTGAAGTGTAAAGTTAAGTCTTGT
GAACGAACAAAATCTCTCGAATTTAT-TAGTTAAGTTGC-TT--TACTTTTT-GCAACTTAGCGCGAAAAGTTTGAT-ATAAGTTGT-T
TA--GCCTAAA-A---TCAAAAAAAATTGTTTTTACTTTTTATTTGATTGGTTTATCTTT--AGTATA-TA-T-TCGTAT--TTTTAAT
TTTTGCA-AAAAA---AATCAAACAACTAC--CCG-ATAT-GGGCAAGTTTTTG--ACAATTTTTGTAAAAATTA-AAAAT-AC-GAAT
ATACTAATTAGATA-GGTTTAT--TAA-ACAACTTGTTTTGATATAGAGCTGAAGTCAAGTCCTTATGGCCTGTATATGCTGGGCTACA
CACGTGTTACAAGGGTAGAGACAGAGAGTAGCGAATGTGTGAACAGGAGCAAGTCTCTAAAAATTACCTCAGTTCGGATTGTTTTATGT
AACTCTAAAACATGAAGTTGAAATCGTTAGTAATTACACTTGAGTATGGTGTAGTGAATGAAAAGTCAAATTTTGCACACACTGCCCAT
CACGCCCGAAGAGTTGATCCGGAGGGAAGGT-GACTATG-CCGCTATGGAG-ATTTGT-GGTAA-AACAAATCCCTTACTTTAGTTAGT
CG-TCTGCGGATGGCGGATTTTGTTCATCGAAATCAGTTTGTATTTGTGTAATTGACACAAGG
>Haptophrya_dugesiarum_BB_79_DG
TTACTCGTCGTTACTGGGCGTATAGGATACGTAGACGGTTCCAAA-ATGCTA--G-TTACTTAAA-CTATTTGGTTAGGGGTTAGTAAT
TTGGGTACTTGGTTTAGTTAGGGGCTGCGCGCAGGTGTACGTTAGCGATAAAATGCATAAAATGTATGCTGGTTTCCAACAGTGAAAAC
ACACAGCTATAACTAATCGACGTTGAGGTATGAAGGTATGGGTGTCGATCGGGATTAGAGACCCCAGTAGTCCATACTACAAAAGATGA
GTATTCCAATAATACAAAACTTGGAAGCTAACGCGATAAATACTCCGCCTGGGAAGTACGGCCGCAAGGTTAAAACTTAAGAGATTTGG
CGGGAATTTGTTCGAACGGTGGAACATGTGGTTTAATTCGATAATCCACGAAAAATCTTACCAGTGTCGTAATACATTAGAATCGAAGA

GGTAACGCTATCTATCGATTTTTTAGTGTTAACAAGGTGCTGCATGGCTGTCGTCAGTTCGTGCTGTGAAGTGTAAAGTTAAGTCTTGT
GAACGAACAAAATCTCTCGAATTTAT-TAGTTAAGTTGC-TT--TACTTTTT-GCAACTTAGCGCGAAAAGTTTGAT-ATAAGTTGT-T
TA--GCCTAAA-A---TCAAAAAAAATTGTTTTTACTTTTTATTTGATTGGTTTATCTTT--AGTATA-TA-T-TCGTAT--TTTTAAT
TTTTGCA-AAAAA---AATCAAACAACTAC--CCG-ATAT-GGGCAAGTTTTTG--ACAATTTTTGTAAAAATTA-AAAAT-AC-GAAT
ATACTAATTAGATA-GGTTTAT--TAA-ACAACTTGTTTTGATATAGAGCTGAAGTCAAGTCCTTATGGCCTGTATATGCTGGGCTACA
CACGTGTTACAAGGGTAGAGACAGAGAGTAGCGAATGTGTGAACAGGAGCAAGTCTCTAAAAATTACCTCAGTTCGGATTGTTTTATGT
AACTCTAAAACATGAAGTTGAAATCGTTAGTAATTACACTTGAGTATGGTGTAGTGAATGAAAAGTCAAATTTTGCACACACTGCCCAT
CACGCCCGAAGAGTTGATCCGGAGGGAAGGT-GACTATG-CCGCTATGGAG-ATTTGT-GGTAA-AACAAATCCCTTACTTTAGTTAGT
CG-TCTGCGGATGGCGGATTTTGTTCATCGAAATCAGTTTGTATTTGTGTAATTGACACAAGG
>Haptophrya_dugesiarum_BB_87_DG
TTACTCGTCGTTACTGGGCGTATAGGATACGTAGACGGTTCCAAA-ATGCTA--G-TTACTTAAA-CTATTTGGTTAGGGGTTAGTAAT
TTGGGTACTTGGTTTAGTTAGGGGCTGCGCGCAGGTGTACGTTAGCGATAAAATGCATAAAATGTATGCTGGTTTCCAACAGTGAAAAC
ACACAGCTATAACTAATCGACGTTGAGGTATGAAGGTATGGGTGTCGATCGGGATTAGAGACCCCAGTAGTCCATACTACAAAAGATGA
GTATTCCAATAATACAAAACTTGGAAGCTAACGCGATAAATACTCCGCCTGGGAAGTACGGCCGCAAGGTTAAAACTTAAGAGATTTGG
CGGGAATTTGTTCGAACGGTGGAACATGTGGTTTAATTCGATAATCCACGAAAAATCTTACCAGTGTCGTAATACATTAGAATCGAAGA
GGTAACGCTATCTATCGATTTTTTAGTGTTAACAAGGTGCTGCATGGCTGTCGTCAGTTCGTGCTGTGAAGTGTAAAGTTAAGTCTTGT
GAACGAACAAAATCTCTCGAATTTAT-TAGTTAAGTTGC-TT--TACTTTTT-GCAACTTAGCGCGAAAAGTTTGAT-ATAAGTTGT-T
TA--GCCTAAA-A---TCAAAAAAAATTGTTTTTACTTTTTATTTGATTGGTTTATCTTT--AGTATA-TA-T-TCGTAT--TTTTAAT
TTTTGCA-AAAAA---AATCAAACAACTAC--CCG-ATAT-GGGCAAGTTTTTG--ACAATTTTTGTAAAAATTA-AAAAT-AC-GAAT
ATACTAATTAGATA-GGTTTAT--TAA-ACAACTTGTTTTGATATAGAGCTGAAGTCAAGTCCTTATGGCCTGTATATGCTGGGCTACA
CACGTGTTACAAGGGTAGAGACAGAGAGTAGCGAATGTGTGAACAGGAGCAAGTCTCTAAAAATTACCTCAGTTCGGATTGTTTTATGT
AACTCTAAAACATGAAGTTGAAATCGTTAGTAATTACACTTGAGTATGGTGTAGTGAATGAAAAGTCAAATTTTGCACACACTGCCCAT
CACGCCCGAAGAGTTGATCCGGAGGGAAGGT-GACTATG-CCGCTATGGAG-ATTTGT-GGTAA-AACAAATCCCTTACTTTAGTTAGT
CG-TCTGCGGATGGCGGATTTTGTTCATCGAAATCAGTTTGTATTTGTGTAATTGACACAAGG
>Haptophrya_dugesiarum_BB_98_DG
TTACTCGTCGTTACTGGGCGTATAGGATACGTAGACGGTTCCAAA-ATGCTA--G-TTACTTAAA-CTATTTGGTTAGGGGTTAGTAAT
TTGGGTACTTGGTTTAGTTAGGGGCTGCGCGCAGGTGTACGTTAGCGATAAAATGCATAAAATGTATGCTGGTTTCCAACAGTGAAAAC
ACACAGCTATAACTAATCGACGTTGAGGTATGAAGGTATGGGTGTCGATCGGGATTAGAGACCCCAGTAGTCCATACTACAAAAGATGA
GTATTCCAATAATACAAAACTTGGAAGCTAACGCGATAAATACTCCGCCTGGGAAGTACGGCCGCAAGGTTAAAACTTAAGAGATTTGG
CGGGAATTTGTTCGAACGGTGGAACATGTGGTTTAATTCGATAATCCACGAAAAATCTTACCAGTGTCGTAATACATTAGAATCGAAGA
GGTAACGCTATCTATCGATTTTTTAGTGTTAACAAGGTGCTGCATGGCTGTCGTCAGTTCGTGCTGTGAAGTGTAAAGTTAAGTCTTGT
GAACGAACAAAATCTCTCGAATTTAT-TAGTTAAGTTGC-TT--TACTTTTT-GCAACTTAGCGCGAAAAGTTTGAT-ATAAGTTGT-T
TA--GCCTAAA-A---TCAAAAAAAATTGTTTTTACTTTTTATTTGATTGGTTTATCTTT--AGTATA-TA-T-TCGTAT--TTTTAAT
TTTTGCA-AAAAA---AATCAAACAACTAC--CCG-ATAT-GGGCAAGTTTTTG--ACAATTTTTGTAAAAATTA-AAAAT-AC-GAAT
ATACTAATTAGATA-GGTTTAT--TAA-ACAACTTGTTTTGATATAGAGCTGAAGTCAAGTCCTTATGGCCTGTATATGCTGGGCTACA
CACGTGTTACAAGGGTAGAGACAGAGAGTAGCGAATGTGTGAACAGGAGCAAGTCTCTAAAAATTACCTCAGTTCGGATTGTTTTATGT
AACTCTAAAACATGAAGTTGAAATCGTTAGTAATTACACTTGAGTATGGTGTAGTGAATGAAAAGTCAAATTTTGCACACACTGCCCAT
CACGCCCGAAGAGTTGATCCGGAGGGAAGGT-GACTATG-CCGCTATGGAG-ATTTGT-GGTAA-AACAAATCCCTTACTTTAGTTAGT
CG-TCTGCGGATGGCGGATTTTGTTCATCGAAATCAGTTTGTATTTGTGTAATTGACACAAGG
>Haptophrya_dugesiarum_BY_142_DG
TTACTCGTCGTTACTGGGCGTATAGGATACGTAGACGGTTCCAAA-ATGCTA--G-TTACTTAAA-CTATTTGGTTAGGGGTTAGTAAT
TTGGGTACTTGGTTTAGTTAGGGGCTGCGCGCAGGTGTACGTTAGCGATAAAATGCATAAAATGTATGCTGGTTTCCAACAGTGAAAAC
ACACAGCTATAACTAATCGACGTTGAGGTATGAAGGTATGGGTGTCGATCGGGATTAGAGACCCCAGTAGTCCATACTACAAAAGATGA
GTATTCCAATAATACAAAACTTGGAAGCTAACGCGATAAATACTCCGCCTGGGAAGTACGGCCGCAAGGTTAAAACTTAAGAGATTTGG
CGGGAATTTGTTCGAACGGTGGAACATGTGGTTTAATTCGATAATCCACGAAAAATCTTACCAGTGTCGTAATACATTAGAATCGAAGA
GGTAATGCTATCTATCGATTTTTTAGTGTTAACAAGGTGCTGCATGGCTGTCGTCAGTTCGTGCTGTGAAGTGTAAAGTTAAGTCTTGT
GAACGAACAAAATCTCTCGAATTTAT-TAGTTAAGTTGC-TT--TACTTTTT-GCAACTTAGCGCGAAAAGTTTGAT-ATAAGTTGT-T
TA--GCCTAAA-A---TCAAAAAAAATTGTTTTTACTTTTTATTTGATTGGTTTATCTTT--AGTATA-TC-G-TCGTAT--TTTTAAT
TTTTGCA-AAAAA---AATCAAACAACTAC--CCG-ATAT-GGGCAAGTTTTTG--ACAATTTTTGTAAAAATTA-AAAAT-AC-GAAT
ATACTAATTAGATA-GGTTTAT--TAA-ACAACTTGTTTTGATATAGAGCTGAAGTCAAGTCCTTATGGCCTGTATATGCTGGGCTACA
CACGTGTTACAAGGGTAGAGACAGAGAGTAGCGAATGTGTGAACAGGAGCAAGTCTCTAAAAATTACCTCAGTTCGGATTGTTTTATGT
AACTCTAAAACATGAAGTTGAAATCGTTAGTAATTACACTTGAGTATGGTGTAGTGAATGAAAAGTCAAATTTTGCACACACTGCCCAT
CACGCCCGAAGAGTTGATCCGGAGGGAAGGT-GACTATG-CCGCTATGGAG-ATTTGT-GGTAA-AACAAATCCCTTACTTTAGTTAGT
CG-TCTGCGGATGGCGGATTTTGTTCATCGAAATCAGTTTGTATTTGTGTAATTGACACAAGG
>Haptophrya_schmidtearum_JJ_110_SP
TTACTCGTCGTTACTGGGCGTATAGGATACGTAGACGGCTTCAAA-ATGCTATTTACCGA---AA-CAAATTAGTTAGGGTGTAGTAAT
TT-GGTGCTTGGTTTAATCAAAGGCTACACACAGGTGTACGTTAGCGATAAAATGCATAAAACGTATACTGGTTTCCGAAGGTGAAAAC
ATGTCGCTATGATTAATCGACGTTGAGGTATGAAGGTATGGGTGTCGATCGGGATTAGAGACCCCAGTAGTCCATACTACAAAAGATGA
GTATTCCAATTATAGAAAACTTGGAAGCTAACGCGATAAATACTCCGCCTGGGAAGTACGGCCGCAAGGTTAAAACTTAAGAGATTTGG
CGGGAATTTGTTCGAACGGTGGAACATGTGGTTTAATTCGATAATCCACGAAAAATCTTACCAGTGTTGGACTACATTAAAGTCGAAAA
ATTAAT-TATTTTATCGAATTTTTAGTGTTAACAAGGTGCTGCATGGCTGTCGTCAGTTCGTGCTGTGACGTGTAGAGTTAAGTCTCGT
AAACGAACAAAACCTCTGGGGTTCGT-TAACTGGGCTGTTTTACTATTTTTTTGCAGCTTAG--CGTAAGGATTCATCATAATTTATAT
TA-------AA-AAAATCGGTTAAA-GG-TTTTTAACCTC-----AACTTATTTGTTTAGCGGG-ATAATTCTCACAC-TCAAAACAGC
CCCCGCCAATACC---A--CCCTTGGTTACCCTTG-TT-T----TAAG-GTTTG--ACTTAAAGGGTTTTTGGTATAGCGG-GTAGTTT
-TAGTGTTTTTAAACAGATTATTTTGATATAACTT-TAATGAGATAGAGCTGAAGTCAAGTCCTTATGGCCTGTATATGCTGGGCTACA
CACGTGTTACAAGGGTAAAGACAGAGAGTGGCGAGTGCGCAAGTAGGAGCAATTCTCTAAAAATTATCTCAGTTCGGATTGTTTTATGG
AACTCTAAAACATGAAGTTGAAATCGTTAGTAATTACACTTTAGTATGGTGTAGTGAATGAAAAGTCAAATTTTGCACACACTGCCCAT
CACGCCCGAAGAGTTGGTTCGGAGGGAAGATAG-TTA-GACCG-C--GGTGGAATT-TAA-TAAGTTCAAATAACACACCGGTATC-GG
CGCTTT-CGGGTGGCGGTTTGTATACATCGAAATCAGTTTGTATTTGTGTAATTGACACAAGG
>Haptophrya_schmidtearum_JJ_117_SP
TTACTCGTCGTTACTGGGCGTATAGGATACGTAGACGGCTTCAAA-ATGCTATTTACCGA---AA-CAAATTAGTTAGGGTGTAGTAAT

TT-GGTGCTTGGTTTAATCAAAGGCTACACACAGGTGTACGTTAGCGATAAAATGCATAAAACGTATACTGGTTTCCGAAGGTGAAAAC
ATGTCGCTATGATTAATCGACGTTGAGGTATGAAGGTATGGGTGTCGATCGGGATTAGAGACCCCAGTAGTCCATACTACAAAAGATGA
GTATTCCAATTATAGAAAACTTGGAAGCTAACGCGATAAATACTCCGCCTGGGAAGTACGGCCGCAAGGTTAAAACTTAAGAGATTTGG
CGGGAATTTGTTCGAACGGTGGAACATGTGGTTTAATTCGATAATCCACGAAAAATCTTACCAGTGTTGGACTACATTAAAGTCGAAAA
ATTAAT-TATTTTATCGAATTTTTAGTGTTAACAAGGTGCTGCATGGCTGTCGTCAGTTCGTGCTGTGACGTGTAGAGTTAAGTCTCGT
AAACGAACAAAACCTCTGGGGTTCGT-TAACTGGGCTGTTTTACTATTTTTTTGCAGCTTAG--CGTAAGGATTCATCATAATTTATAT
TA-------AA-AAAATCGGTTAAA-GG-TTTTTAACCTC-----AACTTATTTGTTTAGCGGG-ATAATTCTCACAC-TCAAAACAGC
CCCCGCCAATACC---A--CCCTTGGTTACCCTTG-TT-T----TAAG-GTTTG--ACTTAAAGGGTTTTTGGTATAGCGG-GTAGTTT
-TAGTGTTTTTAAACAGATTATTTTGATATAACTT-TAATGAGATAGAGCTGAAGTCAAGTCCTTATGGCCTGTATATGCTGGGCTACA
CACGTGTTACAAGGGTAAAGACAGAGAGTGGCGAGTGCGCAAGTAGGAGCAATTCTCTAAAAATTATCTCAGTTCGGATTGTTTTATGG
AACTCTAAAACATGAAGTTGAAATCGTTAGTAATTACACTTTAGTATGGTGTAGTGAATGAAAAGTCAAATTTTGCACACACTGCCCAT
CACGCCCGAAGAGTTGGTTCGGAGGGAAGATAG-TTA-GACCG-C--GGTGGAATT-TAA-TAAGTTCAAATAACACACCGGTATC-GG
CGCTTT-CGGGTGGCGGTTTGTATACATCGAAATCAGTTTGTATTTGTGTAATTGACACAAGG

## REFERENCES


Bessho, Y., Ohama, T., and Osawa, S. (1992). Planarian mitochondria I. Heterogeneity of cytochrome *c* oxidase subunit I gene sequences in the freshwater planarian, *Dugesia japonica*. *J. Mol. Evol.* 34, 324–330.

Lynn, D. H., and Strüder-Kypke, M. C. (2006). Species of *Tetrahymena* identical by small subunit rRNA gene sequences are discriminated by mitochondrial cytochrome *c* oxidase I gene sequences. *J. Eukaryot. Microbiol.* 53, 385–387. doi: 10.1111/j.1550-7408.2006.00116.x.

Medlin, L., Elwood, H. J., Stickel, S., and Sogin, M. L. (1988). The characterization of enzymatically amplified eukaryotic 16S-like rRNA-coding regions. *Gene* 71, 491–499. doi: 10.1016/0378-1119(88)90066-2

Miao, M., Warren, A., Song, W., Wang, S., Shang, H., and Chen, Z. (2008). Analysis of the internal transcribed spacer 2 (ITS2) region of scuticociliates and related taxa (Ciliophora, Oligohymenophorea) to infer their evolution and phylogeny. *Protist* 159, 519–533. doi: 10.1016/j.protis.2008.05.002

Pawlowski, J. (2000). Introduction to the molecular systematics of foraminifera. *Micropaleontology* 46 (Suppl. 1), 1–12.

Petrov, A. S., Bernier, C. R., Gulen, B., Waterbury, C. C., Hershkovits, E., Hsiao, C., et al. (2014). Secondary structures of rRNAs from all three domains of life. *PLoS One* 9:e88222. doi: 10.1371/journal.pone.0088222

Rataj, M., and Vďačný, P. (2020). Multi-gene phylogeny of *Tetrahymena* refreshed with three new histophagous species invading freshwater planarians. *Parasitol. Res*. 119, 1523–1545. doi: 10.1007/s00436-020-06628-0

Strüder-Kypke, M. C., and Lynn, D. H. (2010). Comparative analysis of the mitochondrial cytochrome c oxidase subunit I (COI) gene in ciliates (Alveolata, Ciliophora) and evaluation of its suitability as a biodiversity marker. *System. Biodivers.* 8, 131–148. doi: 10.1080/14772000903507744

van Hoek, A. H. A. M., Akhmanova, A. S., Huynen, M. A., and Hackstein, J. H. P. (2000). A mitochondrial ancestry of the hydrogenosomes of *Nyctotherus ovalis*. *Mol. Biol. Evol.* 17, 202–206. doi: 10.1093/oxfordjournals.molbev.a026234

Vďačný, P., Bourland, W. A., Orsi, W., Epstein, S. S., and Foissner, W. (2011). Phylogeny and classification of the Litostomatea (Protista, Ciliophora), with emphasis on free-living taxa and the 18S rRNA gene. *Mol. Phylogenet. Evol.* 59, 510–522. doi: 10.1016/j.ympev.2011.02.016

Wang, S., Zhao, Y., Du, Y., and Tang, F. (2019). Morphological redescription and molecular identification of *Trichodina reticulata* Hirschmann & Partsch, 1955 (Ciliophora, Mobilida, Trichodinidae) with the supplemental new data of SSU rDNA and ITS-5.8S rDNA. *J. Eukaryot. Microbiol.* 66, 447–459. doi: 10.1111/jeu.12689

White, T.J., Bruns, T.D., Lee, S., and Taylor, J. (1990). Amplification and direct sequencing of fungal ribosomal RNA genes for phylogenetics. In: Innis, M.A., Gelfand, D.H., Sninsky, J.J., and White, T.J. (eds), PCR protocols, a guide to methods and applications. San Diego, CA: Academic Press, pp. 315–322.
